# Roadmap on computational methods in optical imaging and holography [invited]

**DOI:** 10.1007/s00340-024-08280-3

**Published:** 2024-08-29

**Authors:** Joseph Rosen, Simon Alford, Blake Allan, Vijayakumar Anand, Shlomi Arnon, Francis Gracy Arockiaraj, Jonathan Art, Bijie Bai, Ganesh M. Balasubramaniam, Tobias Birnbaum, Nandan S. Bisht, David Blinder, Liangcai Cao, Qian Chen, Ziyang Chen, Vishesh Dubey, Karen Egiazarian, Mert Ercan, Andrew Forbes, G. Gopakumar, Yunhui Gao, Sylvain Gigan, Paweł Gocłowski, Shivasubramanian Gopinath, Alon Greenbaum, Ryoichi Horisaki, Daniel Ierodiaconou, Saulius Juodkazis, Tanushree Karmakar, Vladimir Katkovnik, Svetlana N. Khonina, Peter Kner, Vladislav Kravets, Ravi Kumar, Yingming Lai, Chen Li, Jiaji Li, Shaoheng Li, Yuzhu Li, Jinyang Liang, Gokul Manavalan, Aditya Chandra Mandal, Manisha Manisha, Christopher Mann, Marcin J. Marzejon, Chané Moodley, Junko Morikawa, Inbarasan Muniraj, Donatas Narbutis, Soon Hock Ng, Fazilah Nothlawala, Jeonghun Oh, Aydogan Ozcan, YongKeun Park, Alexey P. Porfirev, Mariana Potcoava, Shashi Prabhakar, Jixiong Pu, Mani Ratnam Rai, Mikołaj Rogalski, Meguya Ryu, Sakshi Choudhary, Gangi Reddy Salla, Peter Schelkens, Sarp Feykun Şener, Igor Shevkunov, Tomoyoshi Shimobaba, Rakesh K. Singh, Ravindra P. Singh, Adrian Stern, Jiasong Sun, Shun Zhou, Chao Zuo, Zack Zurawski, Tatsuki Tahara, Vipin Tiwari, Maciej Trusiak, R. V. Vinu, Sergey G. Volotovskiy, Hasan Yılmaz, Hilton Barbosa De Aguiar, Balpreet S. Ahluwalia, Azeem Ahmad

**Affiliations:** 1 School of Electrical and Computer Engineering, Ben-Gurion University of the Negev, 8410501 Beer-Sheva, Israel; 2 Institute of Physics, University of Tartu, W. Ostwaldi 1, 50411 Tartu, Estonia; 3 Laboratoire Kastler Brossel, Centre National de la Recherche Scientifique (CNRS) UMR 8552, Sorbonne Universite ´, Ecole Normale Supe ´rieure-Paris Sciences et Lettres (PSL) Research University, Collège de France, 24 rue Lhomond, 75005 Paris, France; 4 Department of Physics and Technology, UiT The Arctic University of Norway, 9037 Tromsø, Norway; 5 Department of Anatomy and Cell Biology, University of Illinois at Chicago, 808 South Wood Street, Chicago, IL 60612 USA; 6 Faculty of Science Engineering and Built Environment, Deakin University, Princes Highway, Warrnambool, VIC 3280 Australia; 7 Optical Sciences Center and ARC Training Centre in Surface Engineering for Advanced Materials (SEAM), School of Science, Computing and Engineering Technologies, Optical Sciences Center, Swinburne University of Technology, Hawthorn, Melbourne, VIC 3122 Australia; 8 Electrical and Computer Engineering Department, Bioengineering Department, California NanoSystems Institute, University of California, Los Angeles (UCLA), Los Angeles, CA USA; 9 Department of Electronics and Informatics (ETRO), Vrije Universiteit Brussel VUB), Pleinlaan 2, 1050 Brussel, Belgium; 10 Swave BV, Gaston Geenslaan 2, 3001 Leuven, Belgium; 11 Applied Optics and Spectroscopy Laboratory, Department of Physics, Soban Singh Jeena University Campus Almora, Almora, Uttarakhand 263601 India; 12 IMEC, Kapeldreef 75, 3001 Leuven, Belgium; 13 Graduate School of Engineering, Chiba University, 1-33 Yayoi-cho, Inage-ku, Chiba, Chiba Japan; 14 Department of Precision Instruments, Tsinghua University, Beijing, 100084 China; 15 Jiangsu Key Laboratory of Spectral Imaging and Intelligent Sense, Nanjing, 210094 Jiangsu China; 16 Fujian Provincial Key Laboratory of Light Propagation and Transformation, College of Information Science and Engineering, Huaqiao University, Xiamen, 361021 Fujian China; 17 Computational Imaging Group, Faculty of Information Technology and Communication Sciences, Tampere University, 33100 Tampere, Finland; 18 Institute of Materials Science and Nanotechnology, National Nanotechnology Research Center (UNAM), Bilkent University, 06800 Ankara, Turkey; 19 Department of Physics, Bilkent University, 06800 Ankara, Turkey; 20 School of Physics, University of the Witwatersrand, Johannesburg, South Africa; 21 Department of Computer Science and Engineering, Amrita School of Computing, Amrita Vishwa Vidyapeetham, Amritapuri, Vallikavu, Kerala India; 22 Department of Biomedical Engineering, North Carolina State University and University of North Carolina at Chapel Hill, Raleigh, NC 27695 USA; 23 Comparative Medicine Institute, North Carolina State University, Raleigh, NC 27695 USA; 24 Bioinformatics Research Center, North Carolina State University, Raleigh, NC 27695 USA; 25 Graduate School of Information Science and Technology, The University of Tokyo, 7-3-1 Hongo, Bunkyo-ku, Tokyo, 113-8656 Japan; 26 World Research Hub Initiative (WRHI), Tokyo Institute of Technology, 2-12-1, Ookayama, Tokyo, 152-8550 Japan; 27 Laboratory of Information Photonics and Optical Metrology, Department of Physics, Indian Institute of Technology (Banaras Hindu University), Varanasi, Uttar Pradesh 221005 India; 28 IPSI RAS-Branch of the FSRC “Crystallography and Photonics” RAS, 443001 Samara, Russia; 29 Samara National Research University, 443086 Samara, Russia; 30 School of Electrical and Computer Engineering, University of Georgia, Athens, GA 30602 USA; 31 Department of Physics, SRM University – AP, Amaravati, Andhra Pradesh 522502 India; 32 Laboratory of Applied Computational Imaging, Centre Énergie Matériaux Télécommunications, Institut National de la Recherche Scientifique, Université du Québec, Varennes, QC J3X1Pd7 Canada; 33 Smart Computational Imaging Laboratory (SCILab), School of Electronic and Optical Engineering, Nanjing University of Science and Technology, Nanjing, 210094 Jiangsu China; 34 Smart Computational Imaging Research Institute (SCIRI), Nanjing, 210019 Jiangsu China; 35 Department of Applied Physics and Materials Science, Northern Arizona University, Flagstaff, AZ 86011 USA; 36 Center for Materials Interfaces in Research and Development, Northern Arizona University, Flagstaff, AZ 86011 USA; 37 Institute of Micromechanics and Photonics, Warsaw University of Technology, 8 Sw. A. Boboli St., 02-525 Warsaw, Poland; 38 LiFE Lab, Department of Electronics and Communication Engineering, Alliance School of Applied Engineering, Alliance University, Bangalore, Karnataka 562106 India; 39 Institute of Theoretical Physics and Astronomy, Faculty of Physics, Vilnius University, Sauletekio 9, 10222 Vilnius, Lithuania; 40 Department of Physics, Korea Advanced Institute of Science and Technology (KAIST), Daejeon, 34141 South Korea; 41 KAIST Institute for Health Science and Technology, KAIST, Daejeon, 34141 South Korea; 42 Tomocube Inc., Daejeon, 34051 South Korea; 43 Quantum Science and Technology Laboratory, Physical Research Laboratory, Navrangpura, Ahmedabad, 380009 India; 44 Research Institute for Material and Chemical Measurement, National Metrology Institute of Japan (AIST), 1-1-1 Umezono, Tsukuba, 305-8563 Japan; 45 Department Chemical Engineering, Ben-Gurion University of the Negev, 8410501 Beer-Shiva, Israel; 46 Applied Electromagnetic Research Center, Radio Research Institute, National Institute of Information and Communications Technology (NICT), 4-2-1 Nukuikitamachi, Koganei, Tokyo 184-8795 Japan

## Abstract

Computational methods have been established as cornerstones in optical imaging and holography in recent years. Every year, the dependence of optical imaging and holography on computational methods is increasing significantly to the extent that optical methods and components are being completely and efficiently replaced with computational methods at low cost. This roadmap reviews the current scenario in four major areas namely incoherent digital holography, quantitative phase imaging, imaging through scattering layers, and super-resolution imaging. In addition to registering the perspectives of the modern-day architects of the above research areas, the roadmap also reports some of the latest studies on the topic. Computational codes and pseudocodes are presented for computational methods in a plug-and-play fashion for readers to not only read and understand but also practice the latest algorithms with their data. We believe that this roadmap will be a valuable tool for analyzing the current trends in computational methods to predict and prepare the future of computational methods in optical imaging and holography.

**Supplementary Information:**

The online version contains supplementary material available at 10.1007/s00340-024-08280-3.

## Introduction (Joseph Rosen and Vijayakumar Anand)

Light is a powerful means that enables imprinting and recording of the characteristics of objects in real-time on a rewritable mold. The different properties of light, such as intensity and phase distributions, polarization and spectrum allow us to sense the reflectivity and thickness distributions and the birefringence and spectral absorption characteristics of objects [1]. When light interacts with an object, the different characteristics of the object are imprinted on those of light, and the goal is to measure the changes in the characteristics of light after the interaction with high accuracy, at a low cost, with fewer resources and in a short time. In the past, the abovementioned measurements involved only optical techniques and optical and recording elements [2–10]. However, the invention of charge-coupled devices, computers, and associated computational techniques revolutionized light-based measurement technologies by sharing the responsibilities between optics and computation. The phenomenal work of several researchers resulted in the gradual introduction of computational methods to imaging and holography [11–15]. This optical-computational association gradually reached several milestones in imaging technology in the following stages. The first imaging approaches were free of computational methods and completely relied on optical elements and recording media. The introduction of computational methods to imaging and holography shifted the full dependency on optics to both partial ones between optics and computation. Today, the field of imaging relies significantly more on computations than on optical elements, with some techniques even free of optical elements [16–20]. With the development of deep learning methods, new possibilities in imaging technology have arisen [20]. The entire imaging process in imaging systems that comprises many optical elements, if broken down into individual steps, reveals several closely knitted computational methods and processes with very few optical methods and processes.

The above evolution leads to an important question: what is the next step in this evolutionary process? This question is not direct or easy to answer. To answer this question, it is necessary to review the current state-of-the-art imaging technologies used in all associated sub-fields, such as computational imaging, quantitative phase imaging, quantum imaging, incoherent imaging, imaging through scattering layers, deep learning and polarization imaging. This roadmap is a collection of some of the widely used computational techniques that assist, improve, and replace optical counterparts in today’s imaging technologies. Unlike other roadmaps, this roadmap focuses on computational methods. The roadmap comprises computational techniques developed by some of the leading research groups that include prominent researchers and architects of modern-day computational imaging technologies. In the past, even today, the goal has been to measure the characteristics of an object, such as intensity, phase, and polarization, using light as a real-time mold, but better and faster, with fewer resources and at a low cost. Although it is impossible to cover the entire domain of imaging technology, this roadmap aims to provide insight into some of the latest computational techniques used in advanced imaging technologies. Mini summaries of the computational optical techniques with associated supplementary materials as computational codes are presented in the subsequent sections.

## Incoherent digital holography with phase-shifting interferometry (Tatsuki Tahara)

### Background

Digital holography (DH) [21–25] is a technique used to record an interference fringe image of the light wave diffracted from an object, termed hologram, and to reconstruct a three-dimensional (3D) image of the object. A laser light source is generally adopted to obtain interference fringes with high visibility. However, a digital hologram of daily-use light is obtained by exploiting incoherent digital holography [26–30]. Using incoherent digital holography (IDH), a speckleless holographic 3D image of the object is obtained. Single-pixel holographic fluorescence microscopy [31], lensless 3D imaging [32], the improvement of the point spread function (PSF) in the in-plane direction [33], and full-color 3D imaging with sunlight [34] were experimentally demonstrated. In IDH, phase-shifting interferometry (PSI) [35] and a common-path in-line configuration are frequently adopted to obtain a clear holographic 3D image of the object and robustness against external vibrations. I introduce IDH techniques using PSI in this section.

### Methodology

**Fig. 1:**
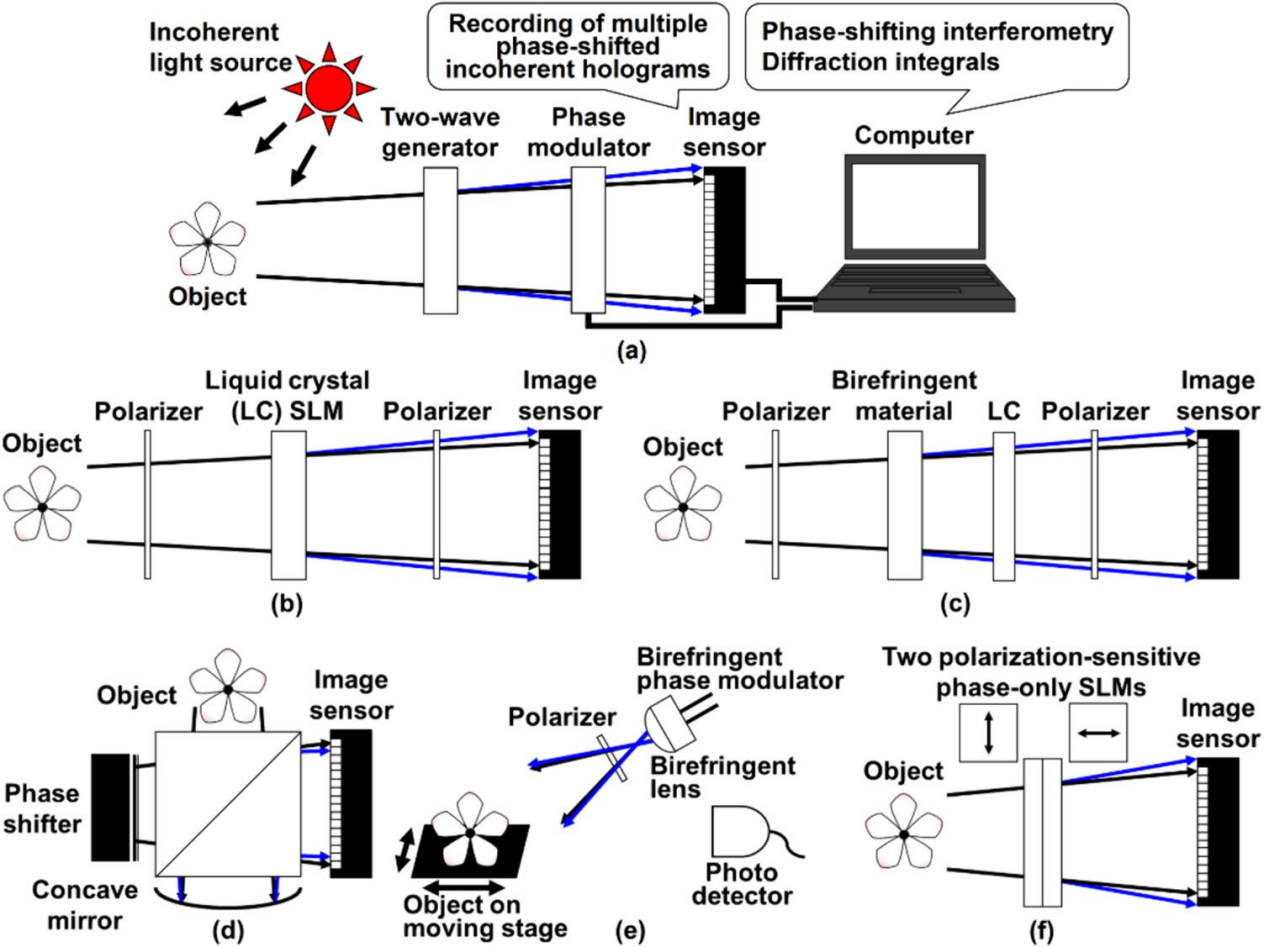
Phase-shifting incoherent digital holography (PS-IDH). **a** Schematic. **b** FINCH-type, **c** conoscopic-holography-type, **d** two-arm-interferometer-type, **e** optical-scanning-holography-type, and **f** TPP-SLMs-type optical setups

Figure 1 illustrates the schematic of the phase-shifting IDH (PS-IDH) and configurations of frequently adopted optical systems. An object wave generated with spatially incoherent light is diffracted from an object. In IDH, self-interference phenomenon is applied to generate an incoherent hologram from spatially incoherent light. Optical elements for generating two object waves whose wavefront curvature radii are different are set to obtain a self-interference hologram as shown in Fig. 1a. A phase modulator is set to shift the phase of one of the object waves, and an image sensor records multiple phase-shifted incoherent digital holograms. The complex amplitude distribution in the hologram is retrieved by PSI, and the 3D information of the object is reconstructed by calculating diffraction integrals to the complex amplitude distribution. In PS-IDH, optical setups of the Fresnel incoherent correlation holography (FINCH) type [26, 28, 36], conoscopic holography type [37, 38], two-arm interferometer type [34], optical scanning holography type [39–41], and two polarization-sensitive phase-only spatial light modulators (TPP-SLMs) type [42, 43] have been proposed. In a FINCH-type optical setup shown in Fig. 1b, a liquid crystal (LC) SLM works as both a two-wave generator and a phase modulator for obtaining self-interference phase-shifted incoherent holograms. In FINCH, phase-shifted Fresnel phase-lens patterns are displayed, and phase-shifted incoherent holograms are recorded. Polarizers are frequently set to improve the visibility of interference fringes. In a conoscopic-holography-type optical setup shown in Fig. 1c, instead of an SLM, a solid birefringent material such as calcite is adopted as a polarimetric two-wave generator. In comparison to that of Fig. 1b, the setup suppresses multi-order diffraction waves when a wide-wavelength-bandwidth light wave illuminates the setup although the size of the setup is enlarged. As another way, IDH is implemented with a classical two-arm interferometer shown in Fig. 1d and the wavefront curvature radius of one of the two object waves is changed by a concave mirror. Robustness against external vibrations is a current research objective. Figure 1e is a setup adopting optical scanning holography [21, 24, 27] and PSI. Phase shifts are introduced using a phase shifter such as an SLM [40, 41] before illuminating an object and phase-shifted Gabor zone plate pattern is illuminated to an object as a structured light. An object is moved along the in-plane direction and a photo detector records a sequence of temporally changed intensity values by introducing phase shifts. The structured light pattern relates the depth position of an object and detected intensity values, and information in the in-plane direction is obtained through optical scanning. Spatially incoherent phase-shifted holograms are numerically generated from the intensity values. The number of pixels and recording speed are dependent on the optical scanning. As described above, PS-IDH systems generally require a polarization filter and/or a half mirror. TPP-SLMs-type optical setup shown in Fig. 1f does not require these optical elements and is constructed to improve the light-use efficiency. Each SLM displays the phase distribution containing two spherical waves with different wavefront curvature radii based on space-division multiplexing, which is termed spatial multiplexing [36]. Phase shifts are introduced to one of the two spherical waves to conduct PSI. By introducing the same phase distributions and phase shifts for respective SLMs, self-interference phase-shifted incoherent holograms are generated. Phase-shifted incoherent holograms for respective polarization directions are formed and multiplexed on the image sensor plane. PS-IDH is implemented when the same phase shifts are introduced for respective polarization directions. It is noted that both 3D and polarization information is simultaneously obtained without a polarization filter by introducing different phase shifts for respective polarization directions and exploiting a holographic multiplexing scheme [42, 43]. Single-shot phase shifting (SSPS) [44–46] and the computational coherent superposition (CCS) scheme [47–49] are combined with these optical setups when conducting single-shot measurement and multidimensional imaging with holographic multiplexing, respectively. CCS is a multidimension-multiplexed PSI technique, and multiple physical quantities such as multiple wavelengths [47–49], multiple wavelength bands [50], and state of polarization [42, 43] are selectively extracted by the signal processing based on PSI. The detailed explanations for PS-IDH with SSPS and CCS are shown in refs. [29, 30].

### Results

**Fig. 2:**
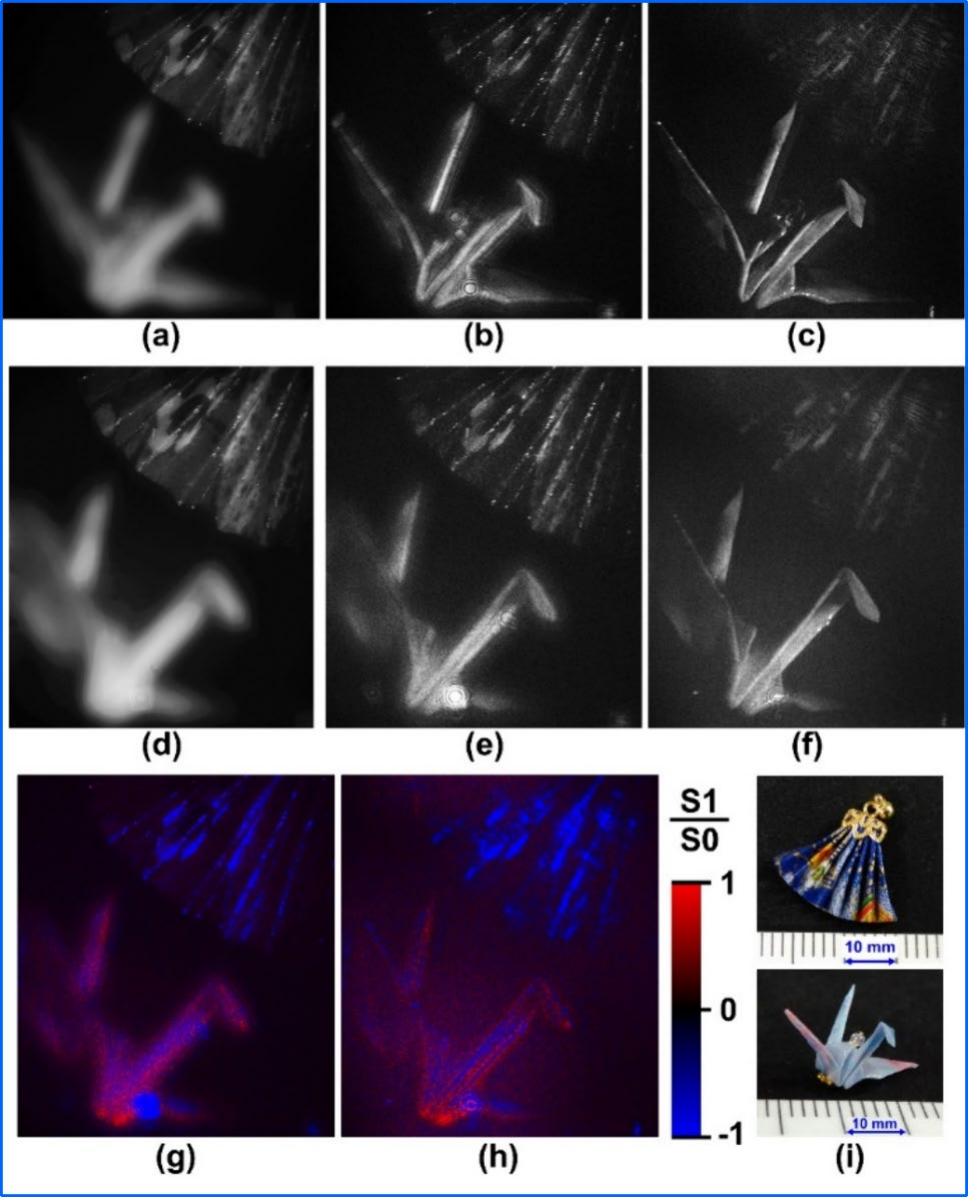
Experimental results of **a**–**c** PS-IDH and **d**–**h** P^4^IDH. **a** One of the phase-shifted holograms. Reconstructed images numerically focused on **b** origami fan and **c** origami crane. **d **One of the polarization-multiplexed phase-shifted holograms. Reconstructed intensity images numerically focused on **e** origami fan and **f** origami crane. Reconstructed polarimetric images numerically focused on **g** origami fan and **h** origami crane. Blue and red colors in **g** and **h** mean that the normalized Stokes parameter S1/S0 is minus and plus according to the scale bar, respectively. The exposure times per recording of a phase-shifted hologram were 100 ms in PS-IDH and 50 ms in P^4^IDH. **i** Photographs of the objects to show these sizes

The TPP-SLMs-type optical setup shown in refs. [42, 43] was constructed for experimental demonstrations. PS-IDH with TPP-SLMs is the IDH system with neither linear polarizers nor half mirrors, and a high light-use efficiency is achieved. Experiments on PS-IDH and filter-free polarimetric incoherent holography, termed polarization-filterless polarization-sensitive polarization-multiplexed phase-shifting IDH (P^4^IDH), which is the combination of PS-IDH and CCS, were carried out. Two objects, an origami fan and an origami crane, were set at different depths, and a polarization film was placed in front of the origami fan. The depth difference was 140 mm. The transmission axis of the film was the vertical direction. In this experiment, I set high-resolution LCoS-SLMs [51] to display the phase distribution of two spatially multiplexed spherical waves whose focal lengths are 850 mm and infinity. Four holograms in the experiment of PS-IDH and seven holograms in the experiment of P4IDH were obtained with blue LEDs (Thorlabs, LED4D201) whose nominal wavelength and full width at half maximum were 455 nm and 18 nm, respectively. The phase shifts in the horizontal and vertical polarizations of the object wave (θ1, θ2) were (0, 0), (π/2, π/2), (π, π), and (3π/2, 3π/2) in the experiment of PS-IDH and (3π/2, 0), (π, 0), (π/2, 0), (0, 0), (0, π/2), (0, π), and (0, 3π/2) in the experiment of P^4^IDH, respectively. The magnification set by four lenses in the constructed N-shaped self-interference interferometer [42, 43] was 0.5 and the field of view for an image hologram in length was 2.66 cm. Figure 2 shows the experimental results. The results indicate that clear 3D image information was reconstructed by PS-IDH and both 3D information and polarization information on the reflective 3D objects were successfully reconstructed without the use of any polarization filter by exploiting P^4^IDH. Depth information is obtained in the numerical refocusing, and quantitative depth-sensing capability is shown. The images of the normalized Stokes parameter S1/S0 shown in Fig. 2g and h describe quantitative imaging capability of polarimetric information.

### Conclusion and future perspectives

Both IDH and PSI are long-established 3D measurement techniques. Single-shot 3D imaging [52–54] and multidimensional imaging such as multiwavelength-multiplexed 3D imaging [55, 56], high-speed 3D motion-picture imaging [57], and filter-free polarimetric holographic 3D imaging [42, 43] have been experimentally demonstrated, merging SSPS and CCS into PS-IDH. Although single-shot 3D imaging with IDH has also been demonstrated by off-axis configurations [26, 58–60], an in-line configuration is frequently adopted in IDH, considering low temporal coherency of daily-use light. Research studies toward filter-free multidimensional motion-picture IDH and real-time measurements, the improvement of specifications such as light-use efficiency and 3D resolution, and developments of promising applications listed in many publications [26–30, 42, 43] are listed as future perspectives. The C-codes for generating the multiplexed Fresnel phase lens and phase shifting are given in supplementary materials S1 and S2 respectively.

## Transport of amplitude into phase using Gerchberg-Saxton algorithm for design of pure phase multifunctional diffractive optical elements (Shivasubramanian Gopinath, Joseph Rosen and Vijayakumar Anand)

### Background

Multiplexing multiple phase-only diffractive optical functions into a single high-efficiency multifunctional diffractive optical element (DOE) is essential for many applications, such as holography, imaging, and augmented and mixed reality applications [61–66]. When multiple phase functions are combined as $\sum_{k}exp\left(j{\Phi }_{k}\right)$, the resulting function is a complex function requiring both phase and amplitude modulations to achieve the expected result. However, most of the available modulators, either phase-only or amplitude-only, make the realization of multifunctional diffractive elements challenging. Advanced phase mask design methods and computational optical methods are needed to implement multifunctional DOEs. One of the widely used methods is the random multiplexing (RM) method, where multiple binary random matrices are designed such that the binary states of any mask are mutually exclusive to one another. One unique binary random matrix is assigned to every diffractive function and then summed [36]. This RM approach allows the combination of more than two diffractive functions in a single phase-only DOE [67]. However, the disadvantages of the RM include scattering noise and low light throughput. The polarization multiplexing (PM) method encodes different diffractive functions to orthogonal polarizations, and consequently, multiplexing more than two functions in a single phase only DOE [68] is impossible. Compared to RM, PM has a higher signal-to-noise ratio (SNR) but relatively lower light throughput due to the loss of light at polarizers. In this section, we present a recently developed computational algorithm called Transport of Amplitude into Phase using the Gerchberg Saxton Algorithm (TAP-GSA) for designing multifunctional pure phase DOEs [69].

### Methodology

**Fig. 3:**
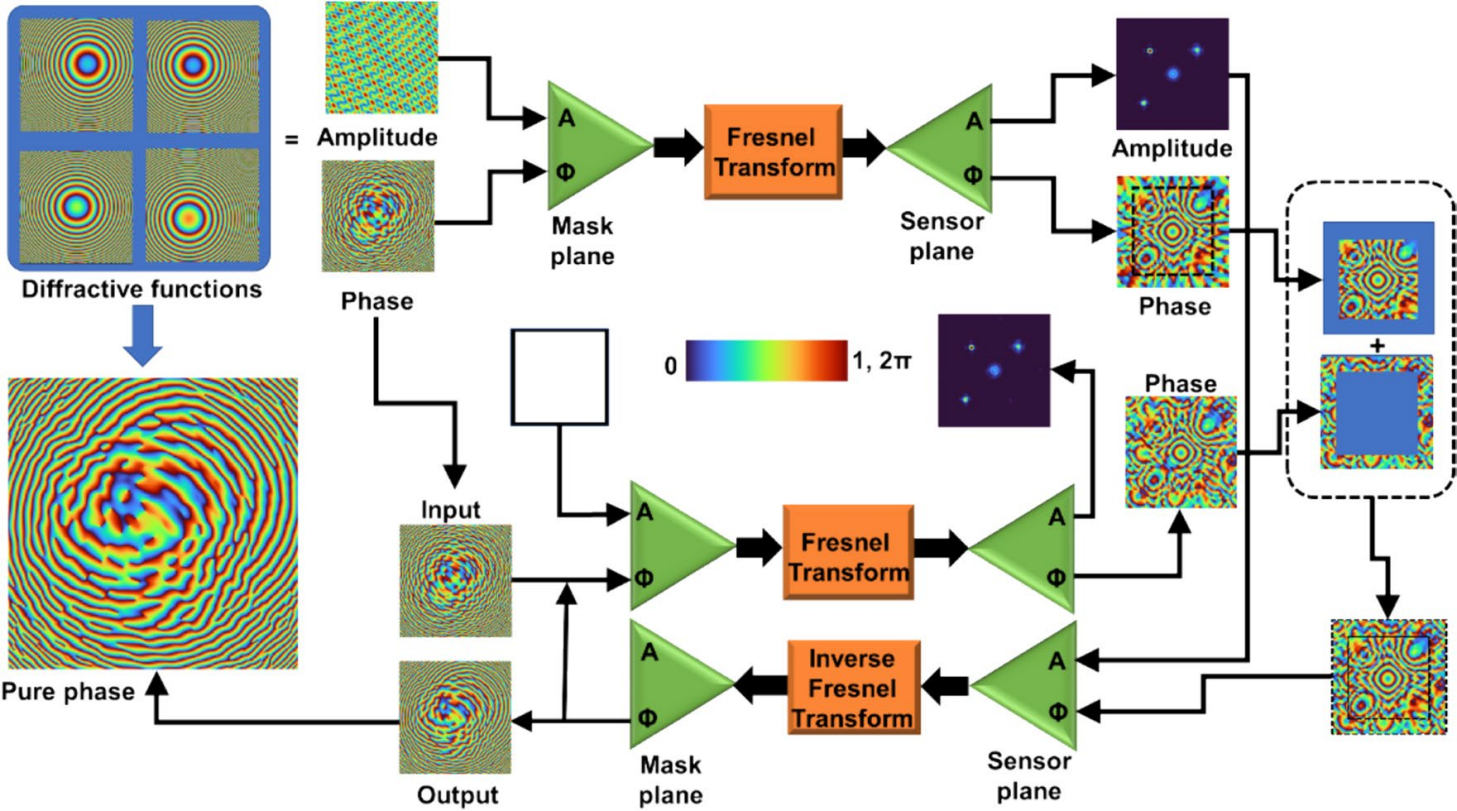
Schematic of TAP-GSA demonstrated here for multiplexing four diffractive lenses with different focal lengths

A schematic of the TAP-GSA is shown in Fig. 3. The TAP-GSA consists of two steps. In the first step, the functions of the DOEs are summed as follows ${C}_{1}=\sum_{k}exp\left(j{\Phi }_{k}\right)$, where ${C}_{1}$ is a complex function at the mask plane. The complex function ${C}_{1}$ is propagated to a plane of interest via Fresnel propagation to obtain the complex function ${C}_{2}=Fr\left({C}_{1}\right)$, where *Fr* is the Fresnel transform operator. After the first step, the following functions are extracted: $Arg({C}_{1})$, $Arg({C}_{2})$ and $\left|{C}_{2}\right|$. Next, the GSA begins with a complex amplitude ${C}_{3}=exp\left[j\cdot Arg({C}_{1})\right]$ at the mask plane, and ${C}_{3}$ is propagated to the sensor plane using the Fresnel transform. At the sensor plane, the magnitude of the resulting complex function ${C}_{4}$ is replaced by $\left|{C}_{2}\right|$, and its phase is partly replaced by $Arg({C}_{2})$. The ratio of the number of pixels replaced by $Arg\left({C}_{2}\right)$ to the total number of pixels is given as the degrees of freedom (DoF). The resulting complex function ${C}_{5}$ is backpropagated to the mask plane by an inverse Fresnel transform, and the phase is carried out while the amplitude is replaced by a uniform matrix of ones. This process is iterated until a nonchanging phase matrix is obtained in the mask plane.

In FINCH or IDH, it is necessary to create two different object beams for every object point. In the first versions of FINCH, the generation of two object beams was achieved using a randomly multiplexed diffractive lens, where two diffractive lenses with two different focal lengths are spatially and randomly multiplexed [36]. Spatial random multiplexing results in scattering noise, resulting in a low SNR. Polarization multiplexing was then developed by polarizing the input object beam along 45° of the active axis of a birefringent device, resulting in the generation of two different object beams with orthogonal polarizations at the birefringent device [33]. A second polarizer was mounted before the image sensor at 45° with respect to the active axis of the birefringent device to cause self-interference. As the SNR improved in polarization multiplexing, the light throughput decreased. TAP-GSA was implemented to design phase masks for FINCH [36].

### Results

**Fig. 4:**
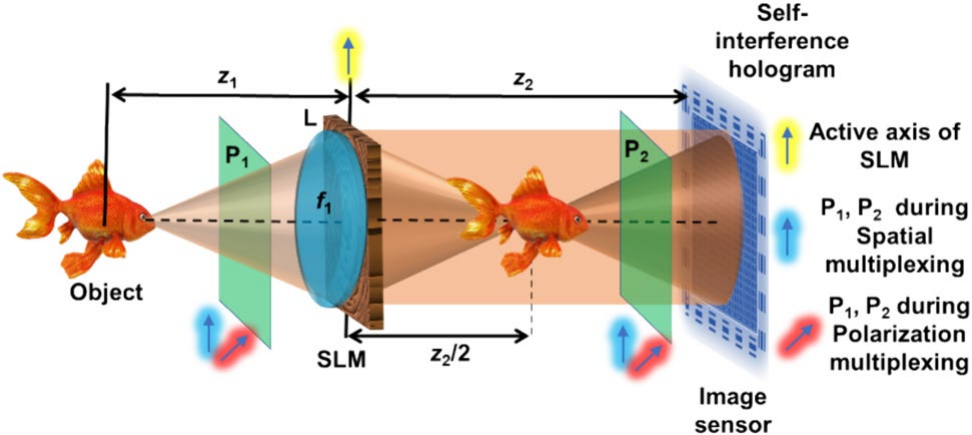
Optical configuration of FINCH

**Fig. 5:**
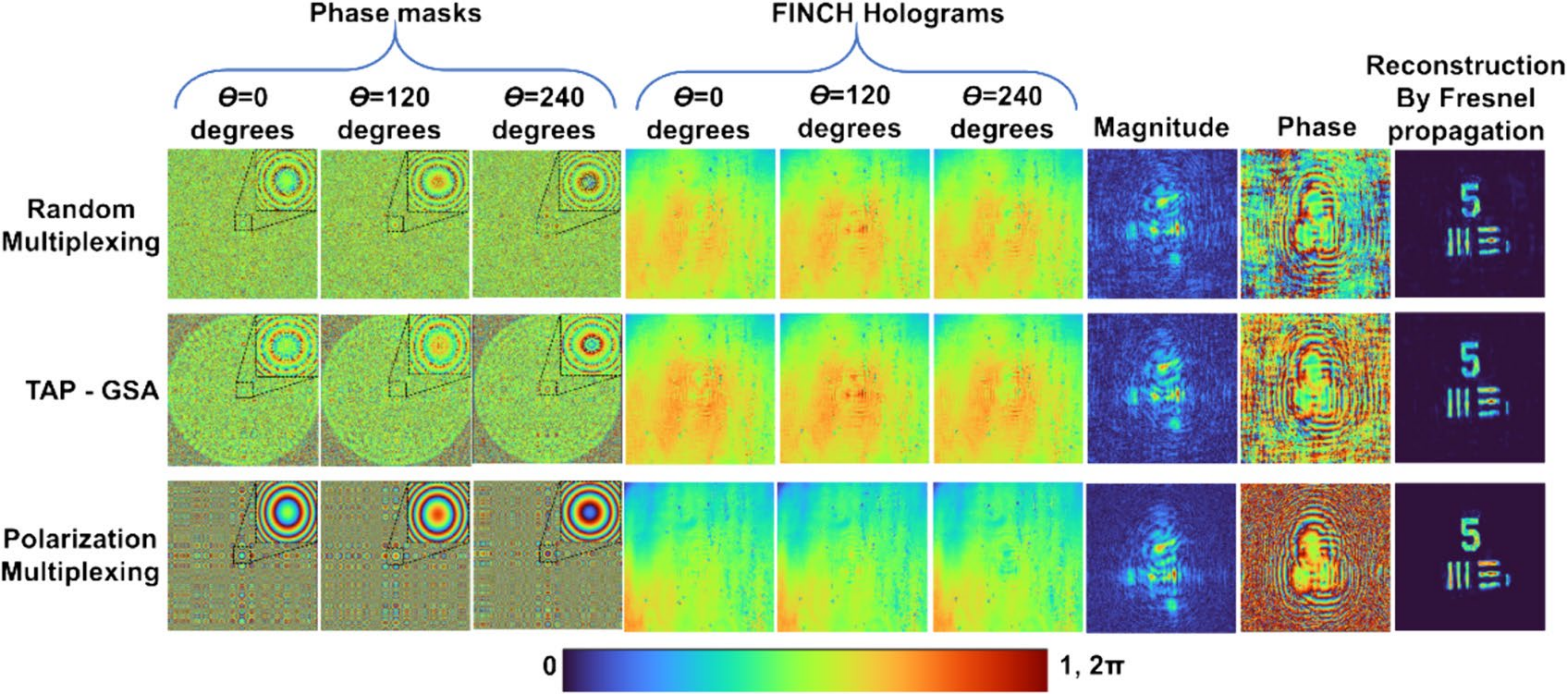
Phase masks and FINCH holograms of the USAF target for *θ* = 0, 120, and 240 degrees, magnitude and phase of the complex hologram and reconstruction result by Fresnel propagation. Rows 1, 2, and 3 are the results for the RM, TAP-GSA, and polarization multiplexing, respectively

The optical configuration of FINCH is shown in Fig. 4. Light from an incoherently illuminated object is collected and collimated by lens L with a focal length of *f*_1_ at a distance of *z*_1_. The collimated light is modulated by a spatial light modulator (SLM) on which dual diffractive lenses with focal lengths *f*_2_ = ∞ and *f*_3_ = *z*_2_/2 are displayed, and the holograms are recorded by an image sensor located at a distance of *z*_2_ from the SLM. The light from every object point is split into two waves that self-interfere to obtain the FINCH hologram. Two polarizers are used one before and one after the SLM for implementing one at a time by the same setup, FINCH with spatial multiplexing using RM, TAP-GSA, at one moment, and polarization multiplexing methods at the other. For RM and TAP-GSA, the multiplexed lenses are displayed with P1 and P2 oriented along the active axis of the SLM. For the PM, P1 and P2 are oriented at 45^o^ with respect to the active axis of the SLM, and a single diffractive lens is displayed on the SLM. The experiment was carried out with a high-power LED (Thorlabs, 940 mW, λ = 660 nm and Δλ = 20 nm), SLM (Thorlabs Exulus HD2, 1920 × 1200 pixels, pixel size = 8 μm) and image sensor (Zelux CS165MU/M 1.6 MP monochrome CMOS camera, 1440 × 1080 pixels with pixel size ~ 3.5 µm) with distances z_1_ = 5 cm and z_2_ = 17.8 cm. The images of the phase masks, FINCH holograms for the three-phase shifts θ = 0, 120, and 240 degrees, magnitude and phase of the complex hologram, and reconstruction results obtained by Fresnel propagation for the RM, TAP-GSA, and PM are shown in Fig. 5. The average background noise of RM, TAP-GSA, and PM are 3.27 × 10^–3^, 2.32 × 10^–3^, and 0.41 × 10^–3^, respectively. The exposure times needed to achieve the same signal level in the image sensor for RM, TAP-GSA, and PM were 440, 384, and 861 ms, respectively. Comparing all three approaches, TAP-GSA has better light throughput than both RM and PM and has an SNR better than that of RM and close to that of PM.

### Conclusion and future perspectives

The useful computational algorithm TAP-GSA was developed to combine multiple phase functions into a single phase-only function. The algorithm has been demonstrated on FINCH to improve both the SNR and the light throughput. We believe that the developed algorithm will benefit many research areas, such as beam shaping, optical trapping, holography and augmented reality. The MATLAB code with comments is provided in the supplementary materials S3.

## PSF engineering for Fresnel incoherent correlation holography (Francis Gracy Arockiaraj, Saulius Juodkazis and Vijayakumar Anand)

### Background

In the previous sections, FINCH was implemented based on the principles of IDH with self-interference, three to four camera shots with phase-shifting and reconstruction by back propagation of the complex hologram. FINCH is a linear, shift-invariant system and therefore FINCH can also be implemented based on the principles of coded aperture imaging (CAI). The FINCH hologram for an object is formed by the summation of shifted FINCH point responses. Therefore, if the point spread hologram (*I*_*PSH*_) library is recorded at different depths, then they can be used as reconstruction functions of FINCH object hologram (*I*_*OH*_) at those depths [70–77]. This FINCH as CAI replaced the multiple camera recordings by a one-time calibration procedure involving the recording of the *I*_*PSH*_ library. However, this approach of FINCH as CAI has the challenges associated with CAI. One of the challenges in the implementation is that the lateral resolution in CAI is governed by the size of the pinhole used for recording the *I*_*PSH*_ instead of the numerical aperture (NA) [70–79]. It is possible to record the *I*_*PSH*_ with a pinhole with a smaller diameter that is close to the lateral resolution limit governed by the NA. But with a smaller aperture, there is lesser number of photons and increased noise. In this section, we present a recently developed PSH engineering technique that allows to improve the reconstructions of FINCH as CAI [80].

### Methodology

**Fig. 6:**
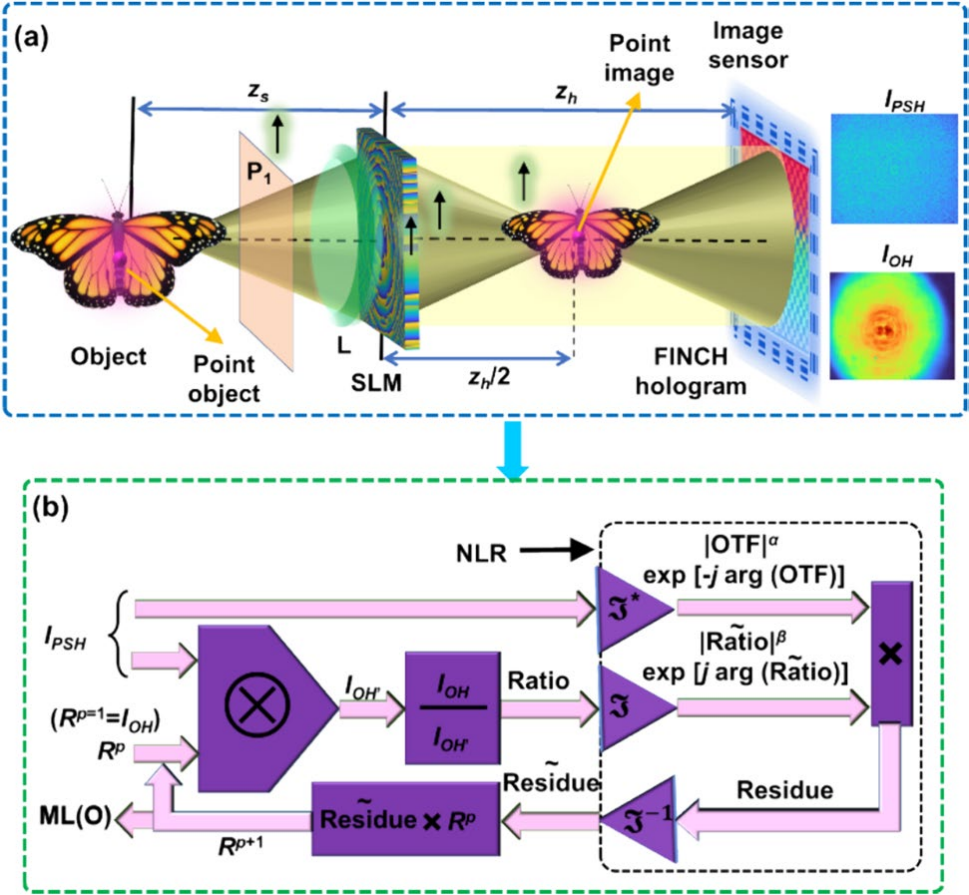
**a** Optical configuration of FINCH as CAI. **b** Schematic of LRRA

The optical configuration of FINCH as CAI using Lucy-Richardson-Rosen algorithm (LRRA) is shown in Fig. 6a. The light from the object point is split into two beams differently modulated using phase masks created from the TAP-GSA displayed on the SLM, and the two beams are then interfered to form a self-interference hologram. The *I*_*PSH*_ and *I*_*OH*_ holograms are required to reconstruct object information using the LRRA. In the PSH engineering technique, the *I*_*PSH*_ is recorded using a pinhole that can allow sufficient number of photons to record a hologram with minimum detector noise in the first step. In the next step, the ideal PSH *I*_*IPSH*_ for a single point is synthesized from the *I*_*PSH*_ recorded for the large pinhole and direct image of the pinhole using LRRA. The engineered PSH *I*_*IPSH*_ is given as $I_{IPSH} = I_{PSH}\circledast_{p}^{\alpha ,\beta }\, I_{D}$, $\circledast_{p}^{\alpha ,\beta }$ is the LRRA operator and ${I}_{D}$ is the direct image of the pinhole. The LRRA operator consists of three parameters *α*, *β* and *p* which are the powers of the magnitudes of the spectrum of matrices and the number of iterations respectively as shown in Fig. 6b. The synthesized *I*_*IPSH*_ and *I*_*OH*_ are used for reconstructing the object information in the final step as $I_{R} = I_{{IPSH}} \circledast_{n}^{{\alpha ,\beta }} \,I_{{OH}}$. With *I*_*IPSH*_ and recorded *I*_*OH*_, the object is reconstructed with an improved resolution and signal to noise ratio (SNR).

**Fig. 7:**
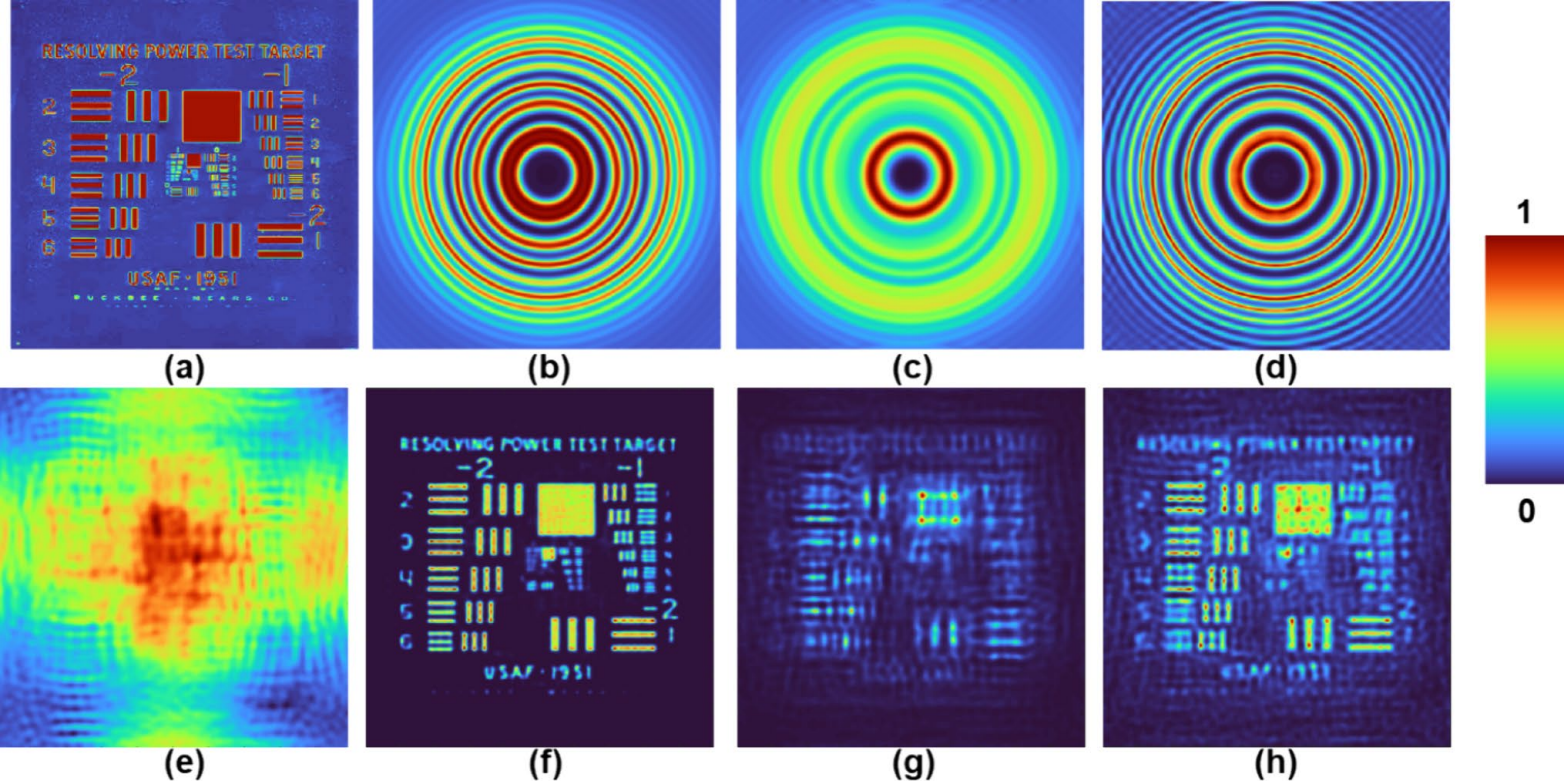
Simulation results of FINCH as CAI. **a** Test object, **b** simulated ideal *I*_*PSH*_, **c** *I*_*PSH*_ simulated with a point object 2.5 times that of NA defined lateral resolution, **d** engineered *I*_*IPSH*_, **e** *I*_*OH*_. Reconstruction results for **f** ideal *I*_*PSH*_ **g** *I*_*PSH*_ simulated with a point object 2.5 times that of NA defined lateral resolution and **h** engineered *I*_*IPSH*_

A simulation study of FINCH as CAI was carried out and the results are shown in Fig. 7. The simulation was carried out in MATLAB. An image of USAF 1951 (Fig. 7a) was used as a test object for the simulation studies. The I_PSH_ for a point object with a size equivalent to the lateral resolution and a point object with 2.5 times larger than the point object are shown in Fig. 7b and c respectively. The I_IPSH_ synthesized from Fig. 7c and the direct image of the pinhole using LRRA is shown in Fig. 7d. The object hologram I_OH_ is shown in Fig. 7e. The reconstruction results using Fig. 7b–d are shown in Fig. 7f–h respectively. As seen from the results, PSH engineering approach has more information and better SNR than the results obtained using the PSH recorded using a large pinhole.

## Results

**Fig. 8:**
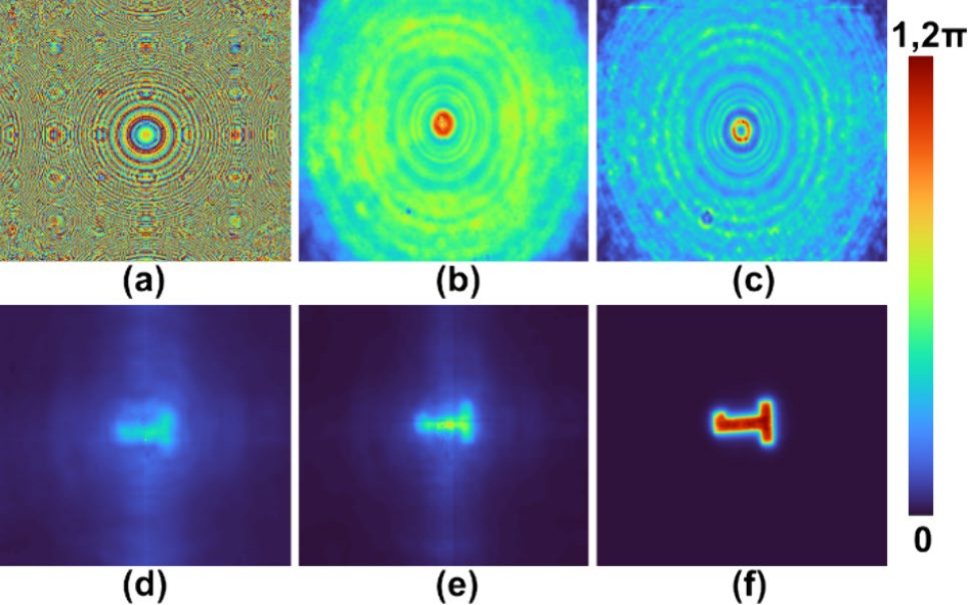
Experimental results of FINCH as CAI. **a** FINCH phase mask for DoF 98%, **b** recorded *I*_*PSH*_ for 50 μm, **c** Engineered *I*_*PSH*_, **d** reconstruction result of (**b**), **e** reconstruction result of (**c**), **f** direct imaging result

An optical experiment similar to Sect. 3 was carried out but instead of three camera shots, a single camera shot for a pinhole with a diameter of 50 µm and a USAF object digit ‘1’ from Group 5 were recorded. The images of the phase mask designed using TAP-GSA with a 98% DoF, recorded *I*_*PSH*_ and engineered *I*_*IPSH*_ are shown in Fig. 8a–c respectively. The reconstruction results using LRRA for IPSH and engineered *I*_*IPSH*_ for α = 0.4, β = 1 and p = 10 are shown in Fig. 8d and e respectively. The direct imaging result of the USAF object is shown in Fig. 8f. From the results shown in Fig. 8d–f, it can be seen that the result of PSH engineering has better SNR and more object information compared to the result obtained for a PSH recorded using a large pinhole.

### Conclusion and future perspectives

The lateral resolution of all imaging systems is governed by the NA of the system. However, in CAI, there is a secondary resolution limit given by the size of the pinhole that is used to record the PSF. This secondary resolution is usually lower than the NA defined lateral resolution. When FINCH is implemented as CAI, the above limitation ruins one of the most important advantages of FINCH which is the super lateral resolution. A PSH engineering method has been developed to shift the resolution limit of CAI back to the limit defined by the NA. A recently developed algorithm LRRA was used for this demonstration. However, the developed PSH engineering method can also work with other reconstruction methods such as non-linear reconstruction [81], Weiner deconvolution [82] and other advanced non-linear deconvolution methods [83]. While the PSH engineering approach improved the reconstruction results, advanced reconstruction methods are needed to minimize the differences in SNR between reconstructions of ideal PSH and synthesized ideal PSH. The PSH engineering method is not limited to FINCH as CAI but can be applied to many CAI methods [84]. The MATLAB code for implementing the PSH engineering method using LRRA is given in the supplementary section S4.

## Single molecule localization from self-interference digital holography (Shaoheng Li and Peter Kner)

### Background

Single Molecule Localization Microscopy (SMLM) has emerged as a powerful technique for breaking the diffraction limit in optical microscopy, enabling the precise localization—typically to less than 20 nm—of individual fluorescent molecules within biological samples [85]. However, the maximum depth of field for 3D-SMLM so far is still limited to a few microns. Self-interference digital holography (SIDH) can reconstruct images over an extended axial range [26]. We have proposed combining SIDH with SMLM to perform 3D super-resolution imaging with nanometer precision over a large axial range without mechanical refocusing. Previous work from our group has experimentally demonstrated localization of fluorescent microspheres using SIDH from only a few thousand photons [86–88]. SIDH produces a complex hologram from which the full 3D image of the emitter can be recreated. By determining the center of this 3D image, the emitter can be localized. Here, we describe the algorithm for localizing emitters from the SIDH data.

### Methodology

Three raw images of one or a few emitters are collected with an added phase shifts of 120° introduced between the two arms of the interferometer. The PSH is then calculated using the standard formula which eliminates the background and twin image [36]. The PSF can then be calculated from the PSH by convolution with the kernel, $\text{exp}\left(j\pi {\rho }^{2}/\lambda {z}_{r}\right)$, where ${z}_{r}$ is the reconstruction distance of the emitter image. By reconstructing 2D images as ${z}_{r}$ is varied, a 3D image stack can be created. Reconstruction of the in-focus PSF requires knowledge of the emitter axial location. Therefore, to reconstruct and localize an arbitrary emitter, a coarse axial search must first be done by varying ${z}_{r}$. The PSF is located by finding the approximate intensity maximum over the z-stack [86]; the axial step should be chosen less than the PSF axial width. Then, the 3D PSF of the emitter can be reconstructed with a finer axial step—100 nm for our experiments. For other 3D SMLM techniques, the axial localization is determined by PSF shape or by comparing two different PSF images [89, 90]. Because SIDH provides access to the full 3D PSF, the center of emission can be localized in all 3 dimensions using the same approach. The 3D centroid can be calculated, or maximum likelihood estimation can be used to determine the center of a three-dimensional Gaussian approximation to the PSF [91]. Here, we localize the center of the PSF by performing two-dimensional curve-fitting. 2D xy and yz slices are cut through the maximum intensity pixel and Gaussian fits are performed. The curve-fits yield the center of the Gaussian, $\left({x}_{c}, {y}_{c},{z}_{c}\right)$, the size of the Gaussian, $\left({\sigma }_{x},{\sigma }_{y},{\sigma }_{z}\right)$, and the total signal.

### Results

**Fig. 9:**
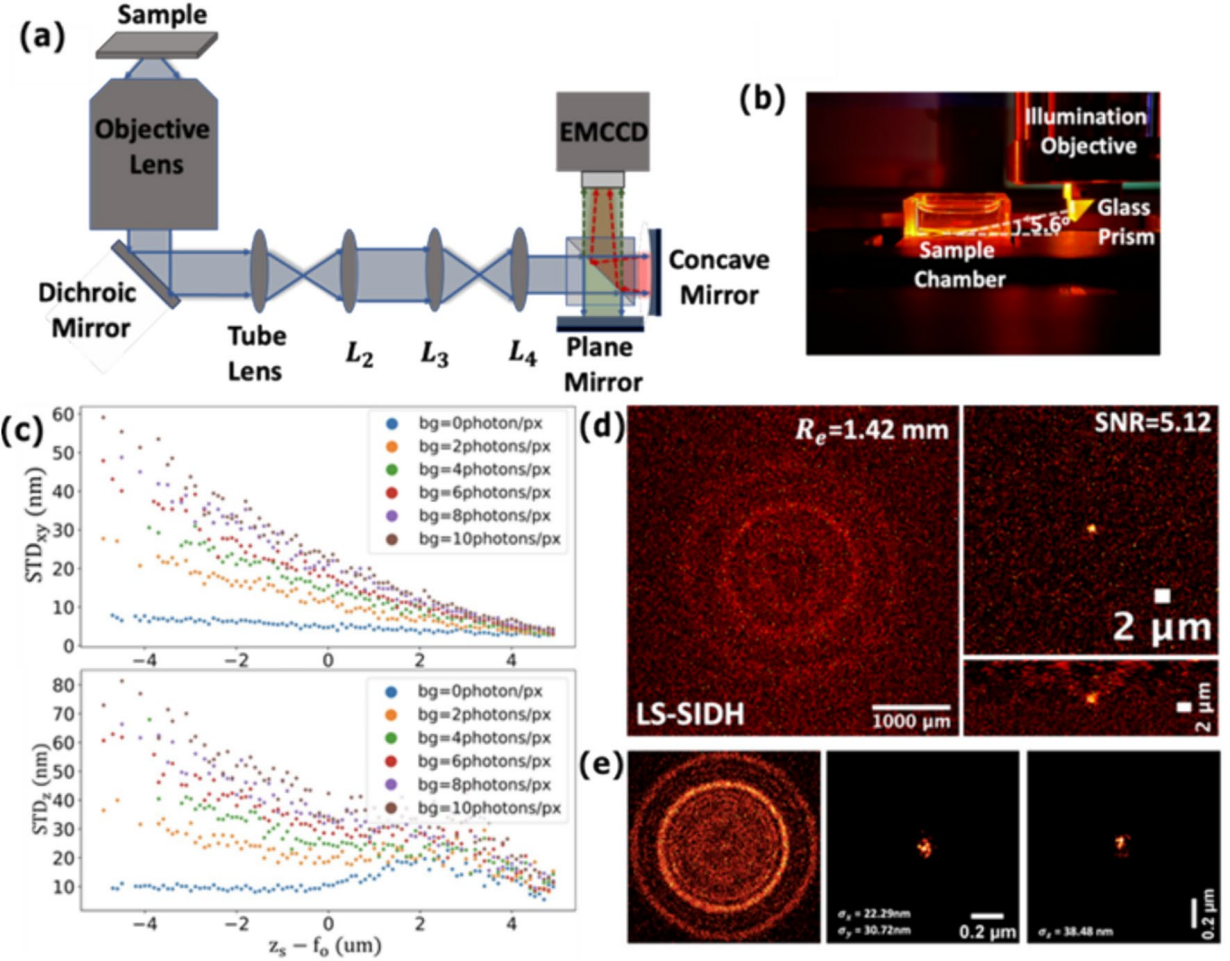
**a** Detailed schematic of the imaging path of the optimized SIDH setup with a Michelson interferometer. **b** The custom designed sample chamber for the tilted light-sheet (LS) illumination pathway. **c** Simulation results of lateral (top) and axial (bottom) localization precision of the optimized SIDH setup with the different background noise levels across a 10 µm imaging range. d The hologram of a 40 nm microsphere imaged with light-sheet illumination (left). Lateral (top) and axial (bottom) views of the image reconstructed by back-propagating the hologram. The SNR was calculated as the ratio of mean signal to the standard deviation of the background. **e** The PSH of a 100 nm microsphere (left). Scatter plots of the localizations in the xy-plane (middle) and yz-plane (right) of images reconstructed by back-propagating the hologram

Results are shown in Fig. 9. Figure 9a shows a schematic of the optical setup. Figure 9b shows the light-sheet illumination which is used to reduce background. The hologram is created by a Mach–Zehnder interferometer consisting of one plane mirror and one concave mirror (f = 300 mm, Edmund Optics). The plane mirror is mounted on a piezoelectric translation stage (Thorlabs NFL5DP20) to create the phase shifts necessary for reconstruction. The objective lens is an oil immersion lens (Olympus PlanApoN 60x, 1.42 NA), and the camera is an EMCCD camera (Andor Ixon-897 Life). The focal length of the tube lens is 180 mm. The focal length of ${L}_{2}$ is set to ${f}_{2}$ = 120 mm. The focal lengths of the relay lenses ${L}_{3}$ and ${L}_{4}$ are ${f}_{3}$ = 200 mm and ${f}_{4}$ = 100 mm, respectively. The distance from the interferometer to the camera is set to 100 mm. Figure 9b shows the light-sheet illumination path of SIDH, which is used to reduce background noise [88]. The excitation laser beams are initially expanded and then shaped using a cylindrical lens with a focal length of 200 mm (not shown). They are then introduced into the illumination objective and subsequently reflected by the glass prism. As the excitation lasers enter the imaging chamber, the incident angle of the tilted light-sheet is approximately 5.6°. The light sheet beam waist at the sample is 3.4 µm. A more detailed description of the optical system can be found in our earlier work [86–88].

Figure 9c shows results of simulations of the localization precision over a 10 µm axial range. With no background, the localization precision is better than 10 nm in the lateral plane, and better than 30 nm in the axial direction. In Fig. 9d, the results of imaging a 40 nm microsphere emitting ~ 2120 photons are shown. The PSH is shown on the left, and the resulting PSF is shown on the right. As can be seen, even with only a couple thousand photons, a SNR of 5 can be achieved demonstrating that the PSF is bright enough to be localized. In Fig. 9e, the results of imaging a 100 nm microsphere emitting ~ 8400 photons are shown. The microsphere was imaged and localized 50 times. A representative PSH is shown on the left, and scatter plots of the localizations in lateral and axial planes are shown on the right. The standard deviation of the localizations was $\sigma_{x} = 22 {\text{nm}}$, $\sigma_{y} = 30 {\text{nm}}$, and $\sigma_{z} = 38 {\text{nm}}$. As can be seen from Fig. 9c, the localization precision is sensitive to the level of background, and we estimate the background level in Fig. 9e to be 13 photons/pixel.

### Conclusion and future perspectives

We have demonstrated a straightforward algorithm for the localization of point sources from SIDH images. With low background, SMLM-SIDH can achieve better than 10 nm precision in all three dimensions over an axial range greater than 10 µm. In future work, we will optimize the reconstruction process by extracting the fluorophore position directly from the hologram without explicitly reconstructing the PSF. It should also be possible to capture only one hologram and then discard the twin-image based on image analysis. Future work will also include incorporating aberration correction into the reconstruction process. Single fluorophores emit several hundred to several thousand photons, and we plan to demonstrate localization of single fluorophores. The Python codes for SMLM-SIDH are given in supplementary materials S5 and GitHub [92].

## Deep learning-based illumination and detection correction in light-sheet microscopy (Mani Ratnam Rai, Chen Li and Alon Greenbaum)

### Background

**Fig. 10:**
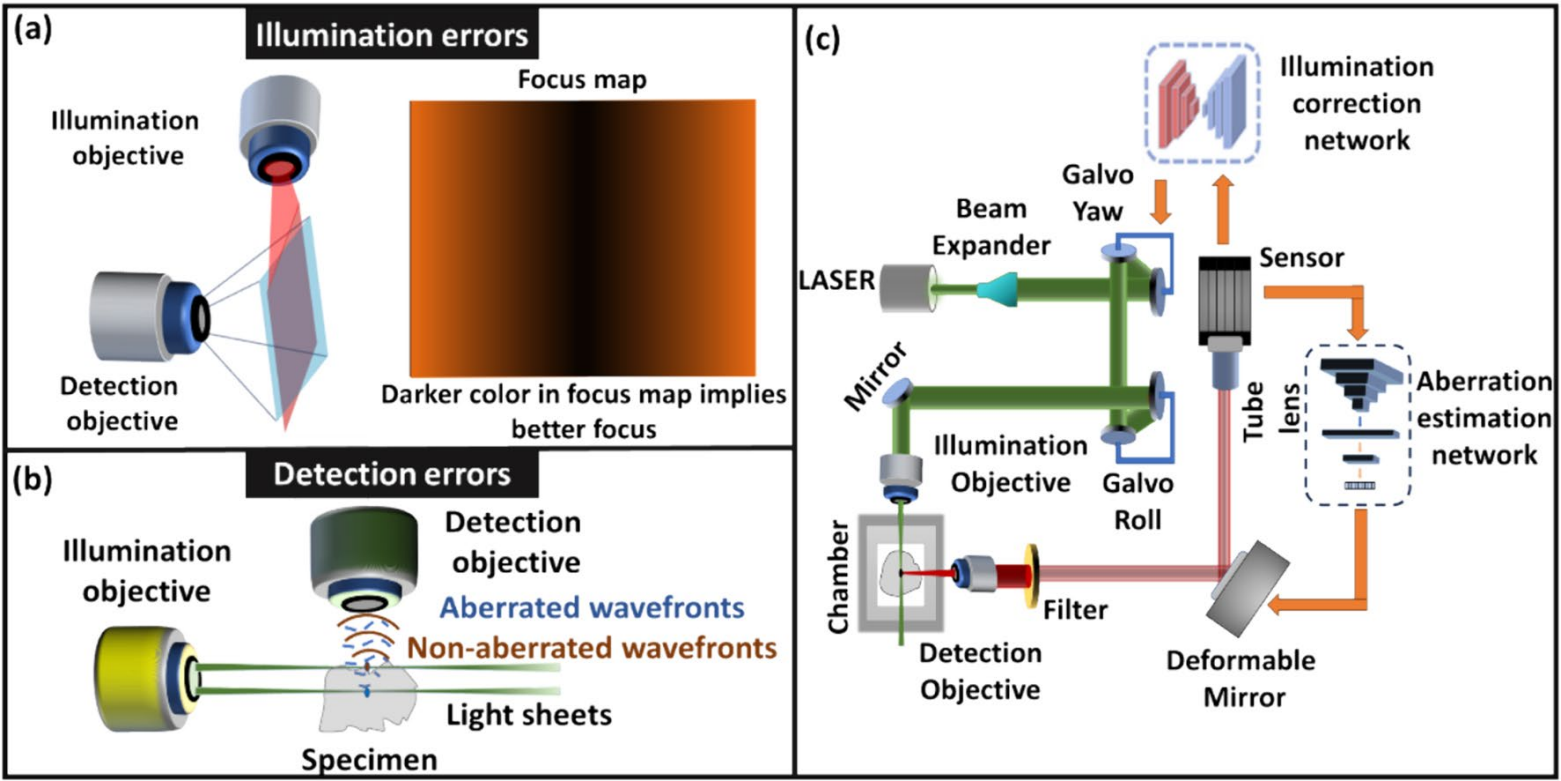
**a** Illumination and **b** detection errors in LSFM. **c** Experimental schematic for correcting the illumination and detection errors in a custom-LSFM, with a deformable mirror, and two galvo mirrors

Light-sheet fluorescence microscopy (LSFM) has become an essential tool in life sciences due to its fast acquisition speed and optical sectioning capability. As such, LFSM is widely employed for imaging large volumes of tissue cleared samples [93]. LSFM operates by projecting a thin light sheet into the tissue, exciting fluorophores, and the emitted photons are then collected by a wide-field detection system positioned perpendicular to the illumination axis of the light sheet [93, 94]. The quality of LSFM images hinges on the performance of both the illumination and detection aspects of the microscopy system. On the illumination side: challenges arise from the non-coplanar alignment of the illumination beam and the focal plane of the detection lens, resulting in uneven focus across the field of view (FOV) (Fig. 10a) [95]. In the detection side, when imaging deep, the tissue components introduce aberrations into the imaging system, particularly when imaging complex specimens such as cochlea, bones, or whole organisms with transitions from soft to hard tissue (Fig. 10b) [94]. Most researchers tend to address either the illumination or detection errors independently, often neglecting their interconnected nature. In this research, we systematically quantified the correction procedures for both illumination and detection errors. Then, we developed two distinct deep learning methods: one for illumination correction and the other for aberration correction on the detection side. The proposed system is thoughtfully designed to achieve the highest quality 3D imaging without the need for human intervention.

### Methodology

The initial phase of our research involved establishing the order for addressing aberrations, namely, whether to correct illumination or detection errors first [94]. Following this, two distinct deep learning models were developed: one for rectifying sample induced detection aberrations and the other for addressing illumination errors, simply put, making sure that the illumination beam was parallel and overlapped with the objective detection plane. In the detection network, we employed a 13-layer RESNET-based network, trained and validated on valuable biomedical samples like porcine cochlea and mouse brain [96]. During training, data are generated by first correcting aberrations using a classical grid search approach per imaging location. Once the aberrations are corrected, a known aberration is introduced into the non-aberrated images using a deformable mirror (DM), and two defocused images with known aberrations are captured. During the testing phase, the network receives two defocused images as input and estimates coma, astigmatism, and spherical aberrations, and the DM is utilized to correct the aberrations based on the predictions of the network. To correct illumination errors, a U-net-based network was utilized and integrated into our LSFM setup [95]. This algorithm captured two defocused images as well, and the images served as input to the deep learning model. The network generated a defocus map. Subsequently, this map is employed to estimate and rectify angular and defocus aberrations through the utilization of two galvo scanners and a linear motorized stage (Fig. 10c).

### Results

**Fig. 11:**
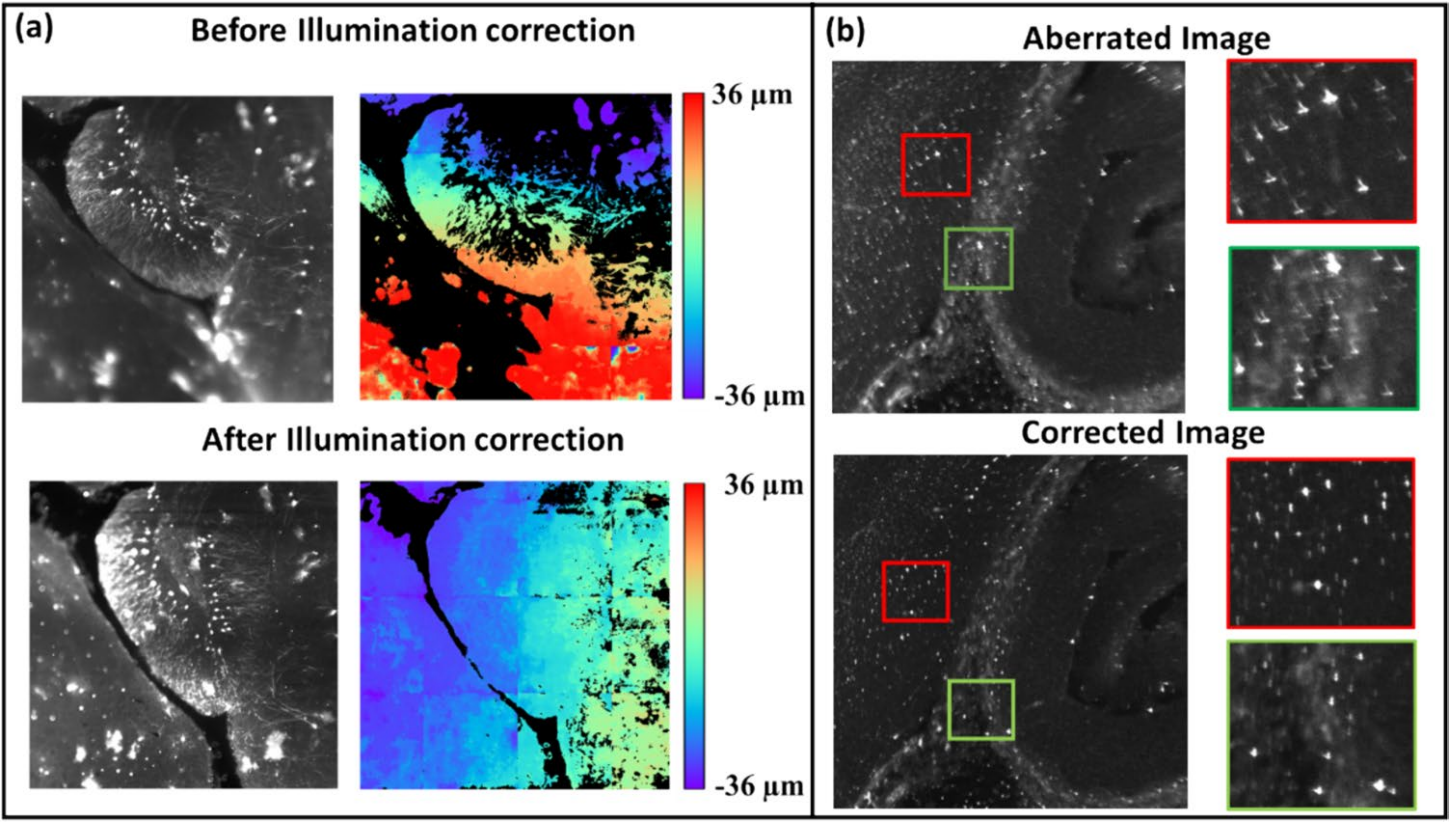
**a** Illumination correction in LSFM. **b** Detection correction in LSFM

The experimental demonstration of the proposed work was performed using a custom-built LSFM system (Fig. 10c). Tissue cleared brains were used to experimentally demonstrate the proposed work. We have found that it is better to first correct the illumination errors and only then the detection aberrations. Figure 11a shows the image before and after illumination correction. Before the correction, only the top portion of the FOV is in focus whereas after the illumination correction, the entire FOV is in focus as seen in the defocus map. The color bar in Fig. 11a shows the defocus level. Figure 11b shows the images before and after correction of detection aberrations.

### Conclusion and future perspectives

In this work, we have developed machine learning based method to correct illumination and detection errors in LSFM. The proposed system can estimate errors from two defocused images. The developed technique will be pragmatic in fully automated error free 3D imaging of large tissue samples without any human intervention. The Python codes for Illumination correction and detection correction are given in https://github.com/Chenli235/AngleCorrection_Unet and https://github.com/maniRr/Detection-correction and in supplementary materials S6.

## Complex amplitude reconstruction of objects above and below the objective focal plane by IHLLS fluorescence microscopy (Christopher Mann, Zack Zurawski, Simon Alford, Jonathan Art, and Mariana Potcoava)

### Background

The Incoherent Holographic Lattice-Light Sheet (IHLLS) technique, which offers essential volumetric data and is characterized by its high sensitivity and spatio-temporal resolution, contains a diffraction reconstruction package that has been developed into a tool, HOLO_LLS that routinely achieves both lateral and depth resolution, at least micron level [28, 97, 98]. The software enables data visualization and serve a multitude of purposes ranging from calibration steps to volumetric imaging of live cells, in which the structure and intracellular milieu is rapidly changing, where phase imaging gives quantitative information on the state and size of subcellular structures [98–100]. This work presents a simple experimental and numerical procedures that have been incorporated into a program package to highlight the imaging capabilities of IHLLS detection system. This capability is demonstrated for 200 nm suspension microspheres and the advantages are discussed by comparing holographic reconstructions with images taken by using conventional Lattice-Light Sheet (LLS). Our study introduces the two configurations of this optical design: IHLLS 1L, used for calibration, and IHLLS 2L, used for sample imaging. IHLLS 1L, an incoherent version of the LLS, creates a hologram via a plane wave and a spherical wave using the same scanning geometry as the LLS in dithering mode. Conversely, IHLLS 2L employs a fixed detection microscope objective to create a hologram with two spherical waves, serving as the incoherent LLS version. By modulating the wavefront of the emission beam with two diffractive lenses uploaded on the phase SLM, this system can attain full Field of View (FOV) and deeper scanning depth with fewer z-galvanometric mirror displacements.

### Methodology

**Fig. 12:**
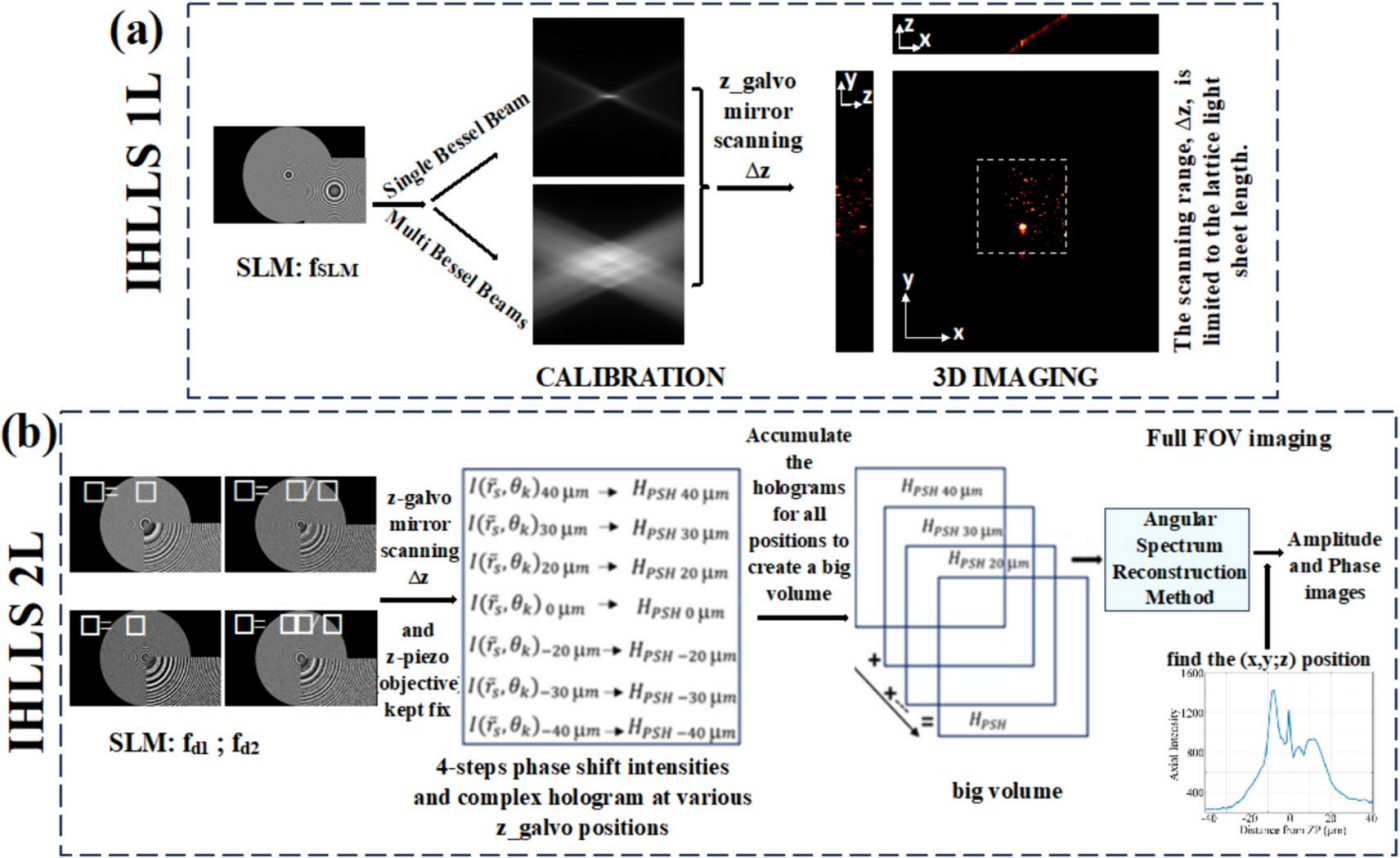
Schematic of glass-lensless IHLLS detection system. **a** IHLLS 1L with one diffractive lens; **b** IHLLS 2L with two diffractive lenses

**Fig. 13:**
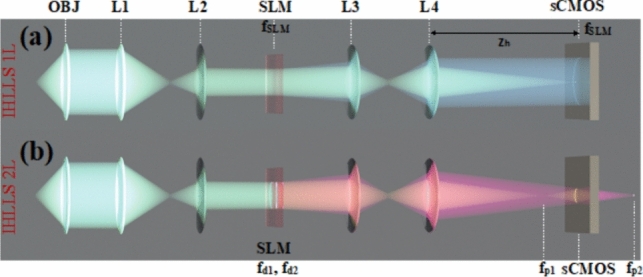
Optical configuration of IHLLS [97]; f_SLM_ = 400 mm, f_d1_ = 435 mm, f_d2_ = 550 mm; here, we chose two focal lengths of size closer to the calibration focal length

The schematic of the IHLLS detection system is shown in Fig. 12. The IHLLS system is a home-built extra hardware added to an existing lattice light-sheet instrument. The IHLLS system is composed of two parts which must both operate in order for the system to perform as intended. The z-scanning principle in IHLLS 1L, same as in LLS, is that both the z-galvanometric mirror (zgalvo) and the detection objective (zpiezo), synchronize in motion to scan the sample in 3D, Fig. 12a. This case is used for calibration purposes, to mimic the conventional LLS but using a diffractive lens of focal length f_SLM [36, 101]. In the IHLLS 2L case, two diffractive lenses of finite focal lengths, with non-shared randomly selected pixels, Fig. 12b, are simultaneously uploaded on the SLM and four phase-shifting intensity images with different phase factors are recorded and saved in the computer sequentially and numerically processed by in-house diffraction software. The complex hologram of an object point located at (${\overline{r} }_{s},{z}_{s}$) = (${x}_{s}, {y}_{s}, {z}_{s}$), as it was described in [36, 101], but using a four-step phase-shifting equation has the expression: $H_{PSH} \left( {x,y} \right) \, = \,I\left( {x,y;\,\theta = 0 } \right) - I\left( {x,y;\,\theta = \frac{\pi }{2}} \right) - i\left( {I\left( {x,y;\,\theta = \pi } \right) - I\left( {x,y;\,\theta = \frac{3\pi }{2}} \right)} \right)$, where $I\left( {x,y;\theta_{k} } \right) = C\left[ {2 + Q\left( {\frac{1}{{z_{r} }}} \right)\exp \left( {i\theta_{k} } \right) + Q\left( { - \frac{1}{{z_{r} }}} \right)\exp \left( { - i\theta_{k} } \right)} \right] ,$ are the intensities of the recorded holograms for each phase shift, ${\theta }_{k}$, *C* is a constant, and $z_{r}$ is the reconstruction distance. The SLM transparency for the two beams has the expression: ${C}_{1}Q\left(-\frac{1}{{f}_{d1}}\right)+{C}_{2}\text{exp}\left(i\theta \right)Q\left(-\frac{1}{{f}_{d2}}\right)$, $Q(b)\, = \,\exp [i\pi b\lambda^{ - 1} (x^{2} + y^{2} )]$ is a quadratic phase function, ${\text{C}}_{\text{1,2}}$ constants, ${\text{f}}_{\text{d}1}$, ${\text{f}}_{\text{d}2}$ are the two diffractive lenses focal lengths, Fig. 13a and b, designed for a specific emission wavelength, and θ is the shift phase factor of the SLM. The two diffractive lenses focus on the planes ${\text{f}}_{\text{p}1}$ and ${\text{f}}_{\text{p}2}$, in the front and behind the camera. In IHLLS 2L technique, ${\text{C}}_{\text{1,2}}=0.5$ and the phase factor has four phase shifts, $\uptheta =0,\uppi /2,\uppi , 3\uppi /2$. When ${\text{f}}_{\text{d}1}=\boldsymbol{\infty }$, Fig. 12a, with an uneven distribution of the two constants, with only one the phase factor of θ = 0, the expression becomes: $0.1 + 0.9\exp \left( {i\theta } \right)Q\left( { - \frac{1}{{f_{SLM} }}} \right)$, and this case refers to the technique called IHLLS 1L. Phase shifted intensity images and hologram reconstructions at multiple z-galvo displacement positions − 40 μm to 40 μm in steps of ∆z = 10 μm were performed on an experimental dataset of 200 nm polystyrene beads acquired with the home-made LLS and IHLLS systems.

### Results

**Fig. 14:**
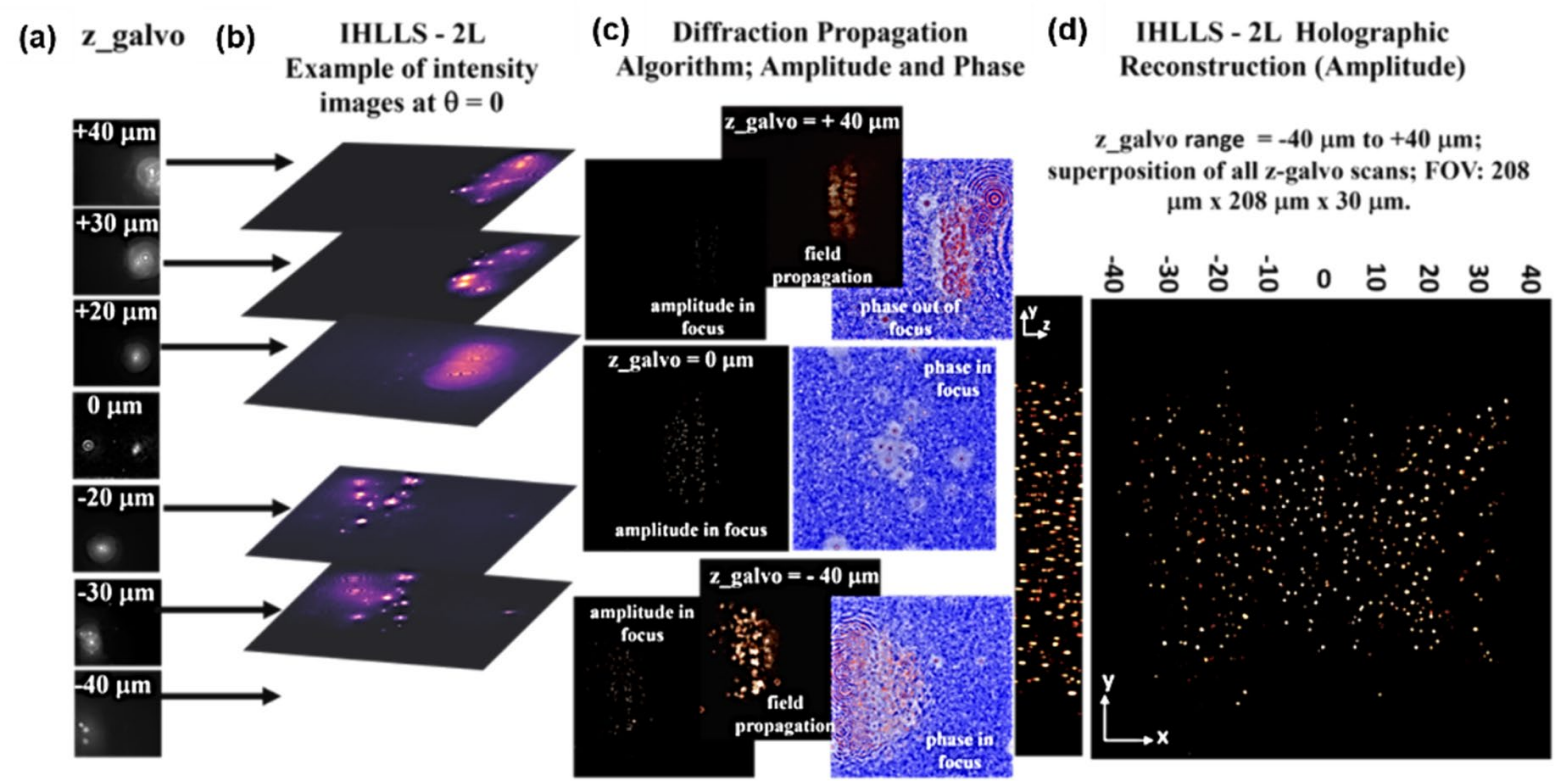
**a** Z-galvo scanning locations. **b** IHLLS – 2L intensity images at θ = 0. **c** Amplitude and phase obtained by Angular Spectrum propagation method. **d** Holographic reconstruction

In this work, we show how to numerically compute IHLLS diffraction patterns with the HOLO_LLS package. The entire package is implemented in MATLAB or Python. Here, we present the MATLAB version. We split the reconstruction process into four steps to produce numerical approximation of the full electric field (amplitude and phase) of the object: (a) addition of all complex fields built by phase shifting holography (PSH) at various z_galvo positions to create a bigger field; (b) apply parameter optimization to the complex wave hologram, a real-space bandpass filter that suppresses the pixel noise while retaining information of a characteristic size, (c) reconstruct the object data from the hologram (backpropagate), and (d) 3D volume representation from the obtained object data. The diffraction subroutine uses the Angular Spectrum Method as the Fresnel and Fraunhofer regimes are limited by the requirement of a different grid scale and by certain approximations [102]. As an example of the methods explained, we present MATLAB pseudocodes for making diffractive lenses and for the 3D volume reconstruction from phase-shift holographic images. We hope this software improves the reproducibility of research, thus enabling consistent comparison of data between research groups and the quality of specific numerical reconstructions. The recorded intensity distributions, amplitude and phase after Fresnel propagation and reconstruction results for different scanning positions of z-galvo mirror from 40 µm to −  40 µm in steps of 10 µm are shown in Fig. 14.

### Conclusion and future perspectives

Our approach will enable automated capture of complex data volumes over time to achieve spatial and temporal resolutions to track dynamic movements of cellular structure in 3D over time. It will enable high temporal resolution of the spatial relationships between cellular structures and retain both amplitude and phase information in the reconstructed images. We have theoretically and practically demonstrated the feasibility of the approach to provide a working microscope system. Our next steps will automate 3D scanning and IHLLS 2L imaging in multiple wavelengths by sweeping excitation through hundreds of z-axis planes. We will then fully automate reconstruction software. Our overall goals are to integrate phase image acquisition in multiple z planes and excitation wavelengths into the existing SPIM software suite. The MATLAB pseudocodes for the HOLO_LLS are provided in the supplementary materials S7.

## Sparse-view computed tomography for passive two-dimensional ultrafast imaging (Yingming Lai and Jinyang Liang)

### Background

Sparse-view computed tomography (SV-CT) is an advanced computational method to obtain the three-dimensional (3D) internal spatial structure [i.e., (*x*, *y*, *z*)] of an object from a few angle-diverse projections [103]. Compared with traditional CT, SV-CT effectively reduces acquisition time with minimally compromising imaging quality. Since its invention, SV-CT has been prominently applied scenarios such as in x-ray medical imaging and industrial product testing scenarios to reduce the radiation dose received by patients and samples [104–106]. In recent years, SV-CT has begun to be noticed as an advanced imaging strategy for efficiently recording spatiotemporal information [i.e., (*x*, *y*, *t*)] [107, 108]. Despite enabling ultrafast imaging speeds, these techniques are based on active laser illumination, making them unsuitable for self-illumination and color-selective dynamic scenes. In this chapter, we present a newly developed compressed ultrafast tomographic imaging (CUTI) method by applying SV-CT to the spatiotemporal domain with passive projections.

### Methodology

**Fig. 15:**
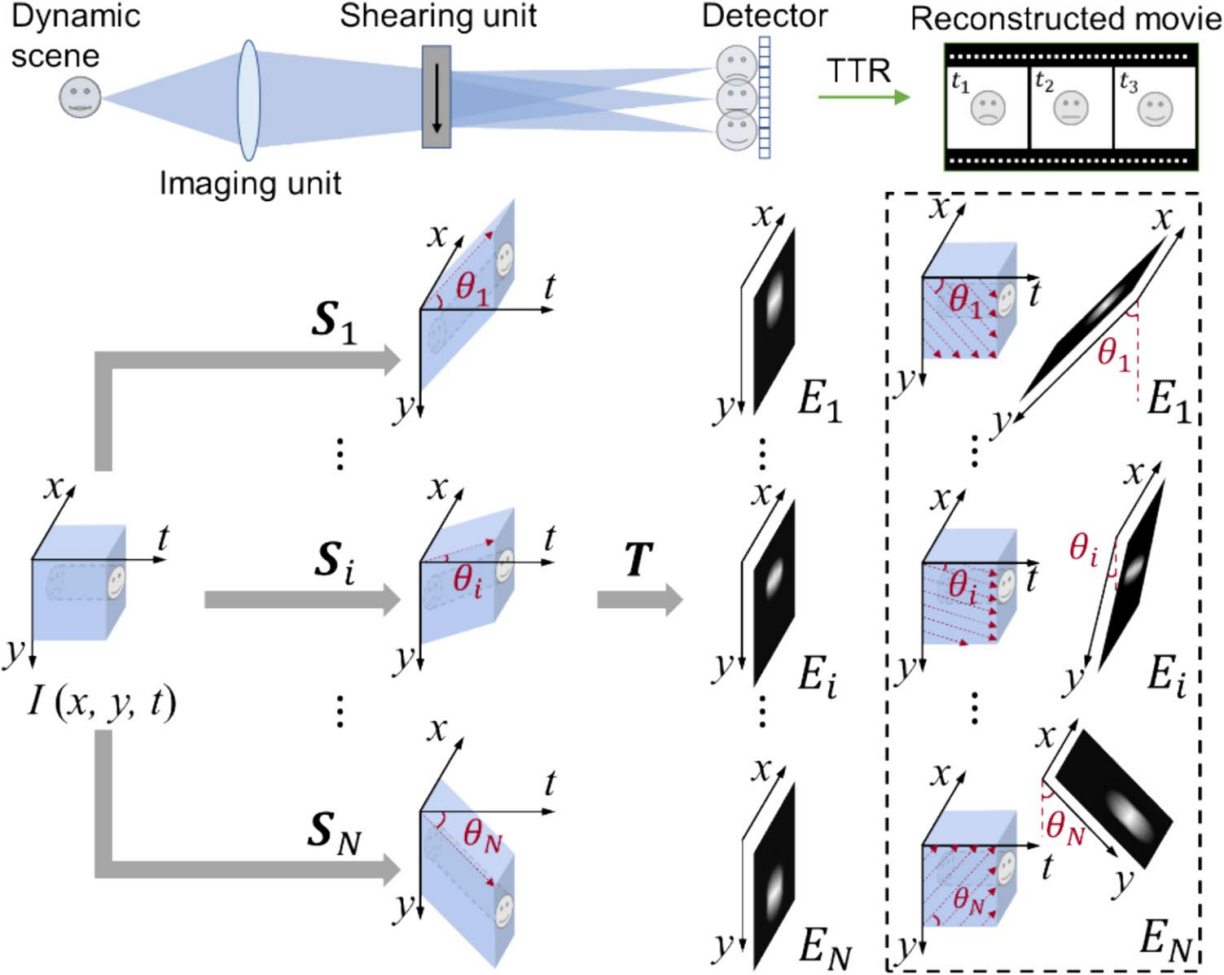
Operating principle of compressed ultrafast tomographic imaging (CUTI). TTR, TwIST-based tomographic reconstruction. Inset in the dashed box: illustrations of the equivalent spatiotemporal projections in data acquisition. Adapted with the permission from Ref. [110]

CUTI achieves spatiotemporal SV-CT based on streak imaging whose typical configuration includes three parts: an imaging unit, a temporal shearing unit, and a two-dimensional (2D) detector. As shown in Fig. 15, after being imaged by the imaging unit, the dynamic scene *I*(*x*, *y*, *t*) is deflected to different spatial positions on the detector by the temporal shearing unit [109]. The multiple-scale sweeping speeds, accessible by the shearing unit, enable the passive projections of the (*x*, *y*, *t*) datacube from different angles in the spatiotemporal domain [110].

The projection angle is determined by the maximum resolving capability of CUTI in both the spatial and temporal dimensions. Particularly, the dynamic information is spatiotemporally integrated into each discrete pixel on the 2D detector after the temporal shearing. Thus, the size of discrete pixels (denoted by ${p}_{\text{c}}$) and the maximum shearing velocity (denoted by ${v}_{\text{max}}$) determine the maximum resolving capability of CUTI in the $t$-axis. During the observation window of ${v}_{\text{max}}$ determined by the sweep time (denoted by ${t}_{\text{s}}$), CUTI’s sequence depth (i.e., the number of frames in the recovered movie) is calculated by ${L}_{t}=\left|{v}_{\text{max}}\right|{t}_{\text{s}}/{p}_{\text{c}}.$ In the $i$th acquisition, the streak length in the spatial direction (e.g., the $y$-axis) is expressed by ${L}_{\text{s}}={v}_{i}{t}_{\text{s}}/{p}_{\text{c}}$, where ${v}_{i}$ is the shearing velocity in the $i$th acquisition ($i=1, 2, 3,\dots ,N$). Hence, the spatiotemporal projection angle, denoted by ${\theta }_{i}$, is determined by

1 $$\theta_{i} = \tan^{ - 1} \left( {\frac{{L_{s} }}{{L_{t} }}} \right) = \tan^{ - 1} \left( {\frac{{v_{i} }}{{\left| {v_{\max } } \right|}}} \right)$$

The sparse projections at different angles of a dynamic event *I(x, y, t)* can be expressed as

2 $$E=\left[\mathbf{T}\mathbf{S}I\left(x,y,t\right)\right],$$

where $E={\left[{E}_{1},{E}_{2}, \dots ,{E}_{N}\right]}^{T}$ is the set of streak measurements, $\mathbf{T}$ is the operator of spatiotemporal integration, and $\mathbf{S}={\left[{\mathbf{S}}_{1}, {\mathbf{S}}_{2}, \dots ,{\mathbf{S}}_{N}\right]}^{T}$ is the set of temporal shearing operations corresponding to various projection angles.

The image reconstruction of CUTI is based on the framework of SV-CT and the two-step iterative shrinkage/thresholding (TwIST) algorithm [111]. The acquired sparse projections are input into a TwIST-based tomographic reconstructions (TTR) algorithm (detailed in the Supplementary information). With an initialization $\hat{I}_{0} = \left( {{\mathbf{TS}}} \right)^{T} E$, the dynamic scene can be recovered by solving the optimization problem of

3 $$\hat{I} = \arg \mathop {\min }\limits_{I} \frac{1}{2}\left\| {E - {\mathbf{TS}}I} \right\|_{2}^{2} + \tau \Phi_{TV} \left( I \right),$$

where $\widehat{I}$ is the reconstructed datacube of the dynamic scene, $\tau$ is the regularization parameter, and ${\Phi }_{\text{TV}}(\cdot )$ is the 3D total-variation regularization function [112].

### Results

**Fig. 16:**
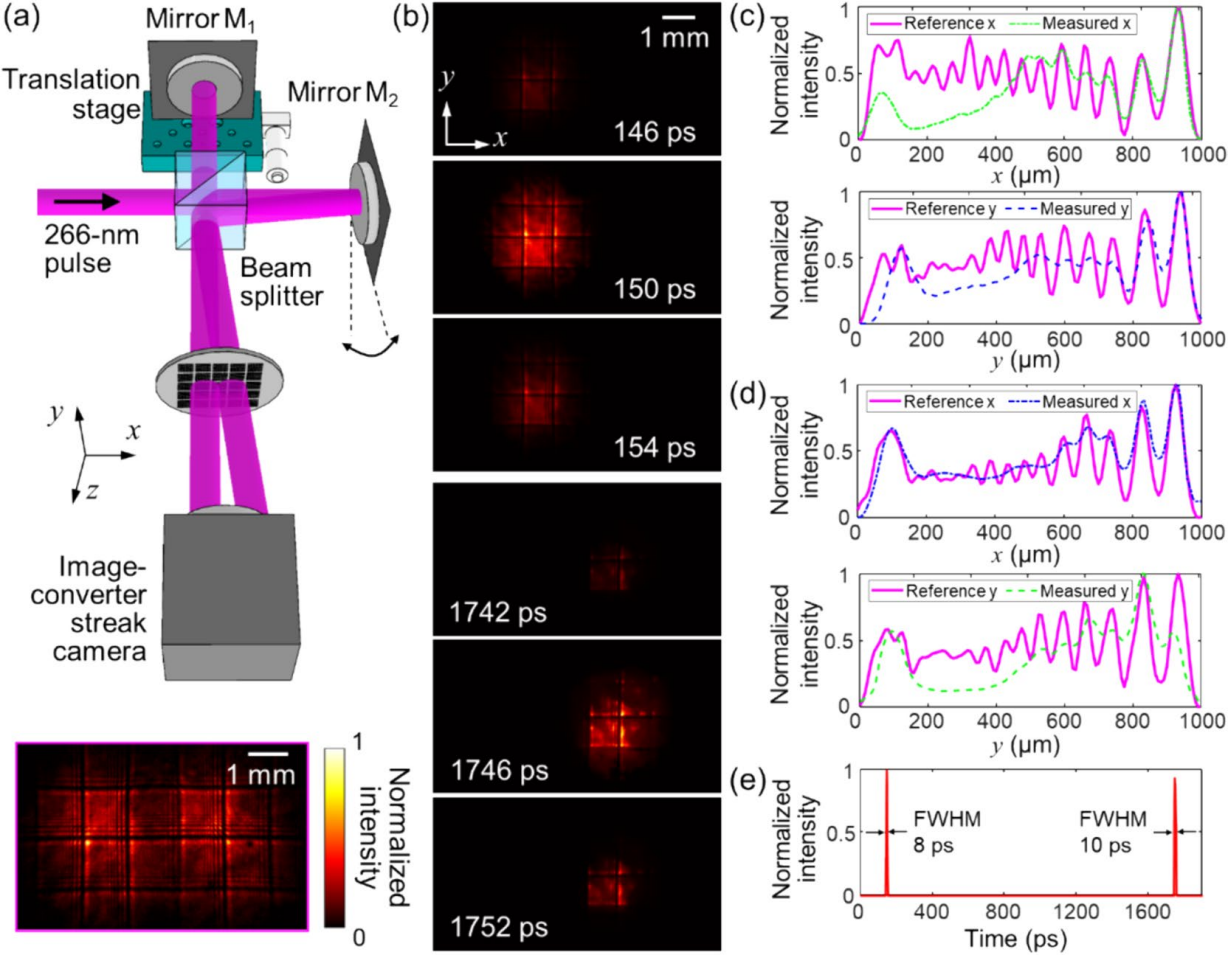
Capture two spatially and temporally separated UV pulses by implementing CUTI to a standard UV streak camera. **a** Experimental setup. M1 − M2: mirrors. Magenta-boxed inset: the reference image was captured without using temporal shearing. **b** Representative frames of the reconstruction scenes. **c** Selected cross-sections of the resolution target in the *x*- and *y*-direction at *t* = 150 ps. (**d**) As (**c**), but shows the profiles *t* = 1746 ps. **e** Temporal trace of the reconstruction. *FWHM* full width at half maximum. Adapted with permission from Ref. [110]

The performance of CUTI was demonstrated using an image-converter streak camera to capture an ultrafast ultraviolet (UV) dynamic event [110]. Figure 16a illustrates the generation of two spatially and temporally separated 266-nm, 100-fs laser pulses via a Michelson interferometer, with a 1.6-ns time delay introduced between them. These pulses undergo modulation by a resolution target as shown in the inset of Fig. 16a. Subsequently, 11 spatiotemporal projections were acquired within the angular range *θ*_*i*_ ∈ [− 45°, + 45°] employing a 9° angular step. By setting the regularization parameter to *τ* = 0.0204, the event was successfully reconstructed using the TTR algorithm at an imaging speed of 0.5 trillion (0.5 × 10^12^) frames per second. Figure 16b represents six representative frames in the reconstruction of the two pulses. To quantitatively assess the image quality, selected cross-sections were extracted at the first pulse (at 150 ps) and the second pulse (at 1746 ps). These results were compared with the reference image captured without temporal shearing (Fig. 16c, d). Using a 10% contrast threshold, at *t* = 150 ps, the spatial resolutions were determined as 15.6 and 14.1 lp/mm in the *x*- and *y*-directions, respectively. At *t* = 1746 ps, the values were 13.2 and 14.1 lp/mm. Figure 16e shows the reconstructed temporal trace of this event.

### Conclusion

As a new computational ultrafast imaging method, CUTI grafts SV-CT to the spatiotemporal domain. The method has been demonstrated in a standard image-converter streak camera for passively capturing an ultrafast UV dynamic event. In the future, CUTI’s image quality can be improved by using an image rotation unit for a larger angular range [113] and adopting advanced SV-CT algorithms [114, 115]. CUTI is expected to contribute to the observation of many significant transient phenomena [116, 117].

## Computational reconstruction of quantum objects by a modal approach (Fazilah Nothlawala, Chané Moodley and Andrew Forbes)

### Background

Optical imaging and holography have traditionally been based on exploiting correlations in space, for instance, using position or pixels as the basis on which to measure. Subsequently, structured illumination with computational reconstruction [118] has exploited the orthogonality in random and Walsh-Hadamard masks, implemented for high-quality 3D reconstruction of classical objects [119] as well as complex amplitude (amplitude and phase) reconstruction of quantum objects [120]. Recently, a modal approach has been suggested to enhance the resolution in imaging [121], taking the well-known ability to modally resolve optical fields for their full reconstruction [122] to that of physical and digital objects. This has been used to infer step heights with nanometer resolution [123], to resolve quantum objects [124], in quantum metrology [125], in phase imaging [126] and suggested as a means of searching for exoplanets [127]. Here, we will apply it to reconstruct quantum images of complex objects and compare it to conventional quantum computational approaches.

### Methodology

**Fig. 17:**
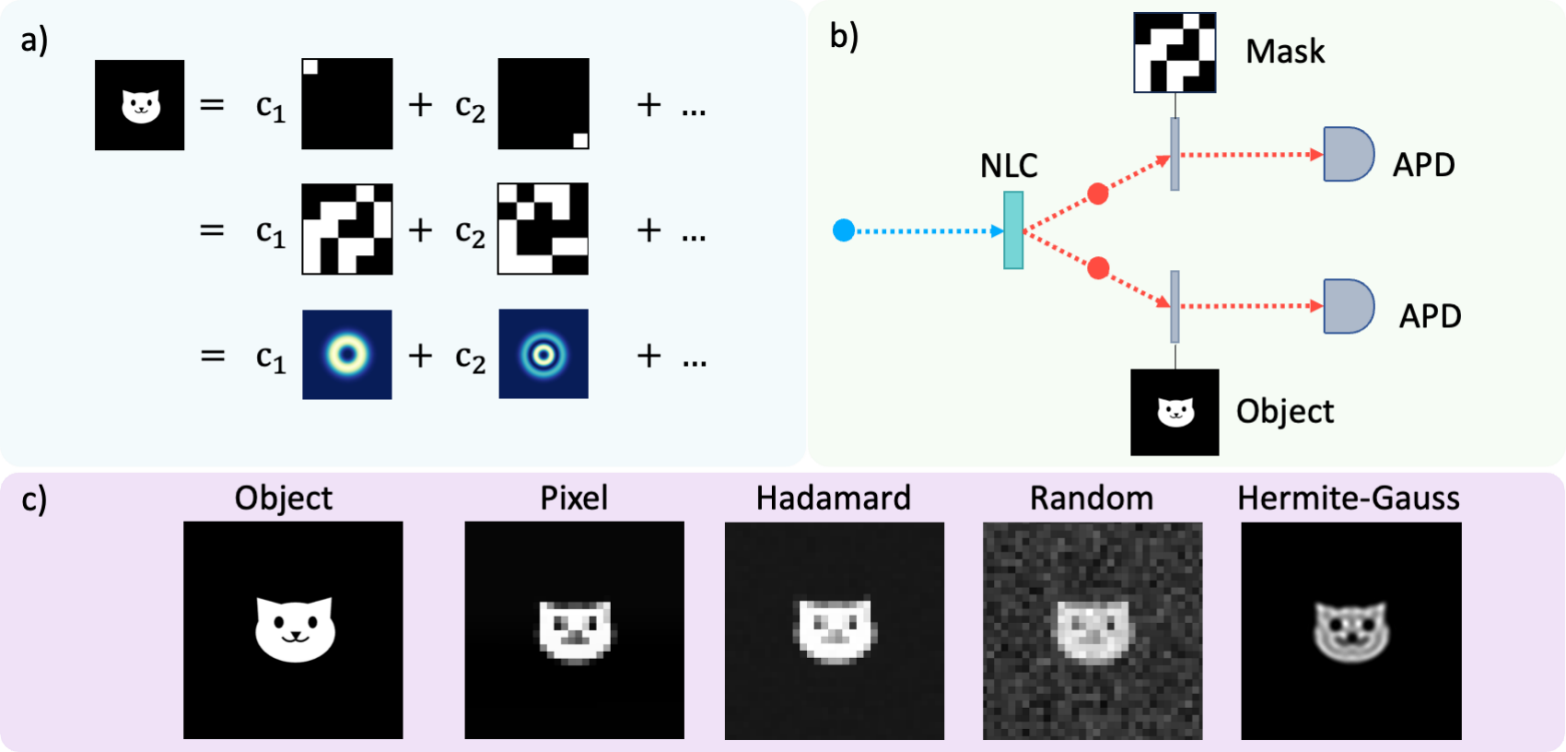
**a** An object can be reconstructed using any complete and orthonormal basis. Three different bases are depicted in this figure: the pixel basis, random basis and a modal basis, respectively. **b** Simple schematic of the experiment where two entangled photons are produced from a nonlinear crystal, one directed to the object and the other to the mask that displays the basis projections. **c** Computational reconstructions of a cat using four mask options

The idea is very simple: any complete and orthonormal basis can be used to reconstruct a function, and this function can represent a physical or digital object. In the present context it is the image of the object. This is depicted graphically in Fig. 17a evolving from a pixel basis (top), to a random basis (middle) and finally to a modal basis (bottom). In the case of the latter, the modal function must be chosen with some care to minimize the number of terms in the sum.

Because the right hand side can include modal phases, any physical property of the left hand side can be inferred, including full phase retrieval. We do exactly this for the recognition of quantum and classical objects using the experimental set-up shown in Fig. 17b. Two entangled photons are produced by spontaneous parametric downconversion (SPDC) in a nonlinear crystal and relay imaged from the crystal plane to the object plane in one arm, and to the image plane in the other arm, the latter with a spatial light modulator as a modal analyzer. Thereafter, each photon is collected by optical fibre and detected by single photon avalanche photodiodes (APDs). The spatial light modulator in the imaging arm is used to display digital match filters for each mode in the basis, while the single mode fibre collection performs an optical inner product to return the modal weights. The intra-modal phase is determined by displaying a superposition of modal elements, two of which (sine and cosine) allow the phase to be known unambiguously. All three measurements together (one for amplitude and two for phase) return the complex weighting coefficient. The final image is then computationally reconstructed by adding the terms on the right hand side with the correct complex weights. The process can be augmented by machine learning and artificial intelligence tools to speed up the reconstruction (with fewer projections) and/or to enhance the final image quality. A simulation of the experiment was performed with computational images of a “cat” object shown in Fig. 17c for four bases.

### Results

**Fig. 18:**
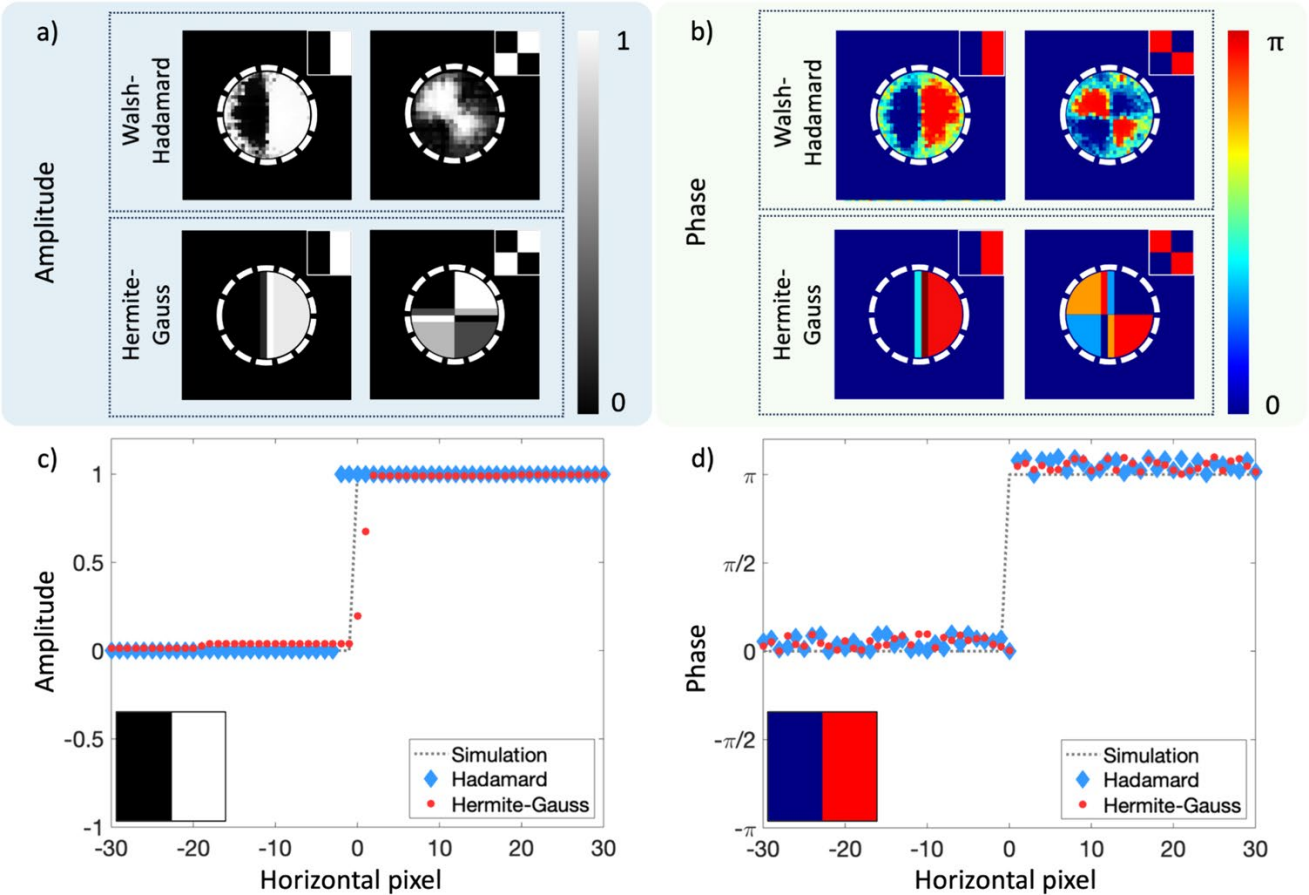
**a** Amplitude and **b** phase reconstructions for a checkerboard pattern and a step object (shown as insets), using Hermite-Gauss (HG) and Walsh-Hadamard masks. The outer area of the dashed white circle represents the region where noise was suppressed due to lack of SPDC signal. 2D cross-sectional plots of the **c** amplitude and **d** phase step functions with the object (simulation), and reconstructions with the Walsh-Hadamard (blue diamonds) and HG (red dots) masks

To illustrate how this approach can be used for quantum objects, we use test cases of (I) an amplitude step and checkerboard pattern object, and (II) a phase step object and checkerboard pattern object for both the Walsh-Hadamard and HG mode reconstructions, with experimental images shown in Fig. 18a and b. The outer area of the dashed white circle for each reconstruction represents the region where noise was suppressed due to lack of SPDC signal. We see the reconstructed images of both the amplitude and phase objects show a high fidelity with both reconstruction approaches (Walsh-Hadamard and HG modes), however the phase objects show a higher object-image fidelity overall. Figure 18c and d provide a quantitative comparison between the object (simulated reconstructed) and the image through a cross-section, showing good agreement between the object (simulated reconstruction) and the experimental reconstructions for both the Hadamard (blue) and Hermite-Gauss (red) amplitude and phase steps, albeit with a low level of noise, characteristic to quantum experiments, present.

### Conclusion and future perspectives

While scanning methods employing the pixel, Walsh-Hadamard and random bases depend directly on the number of pixels required within the image, the modal approach proves beneficial in that there is no direct correlation between the number of scans required and image resolution. The resolution is set by the optical system itself, while the number of modes required to image the object is dependent on the complexity of the object. The modal approach requires a judicious choice of modal basis as well as the number of terms required to image the object. The introduction of phase only and amplitude only scanning through a modal approach allows for the ability to probe individual properties of an unknown object. The future prospects for computational methods in optical imaging and holography are highly promising, with trends indicating integration of AI for enhanced image reconstruction, the advancement of 3D holography with improved resolution, and the potential impact of quantum techniques. These developments will benefit various fields, including bio-photonics, material science, and quantum cryptography. The introduction of quantum computing and interdisciplinary collaborations will likely act as a catalyst for innovation, expanding the applications and accessibility of optical imaging and holography across industrial and research domains.

## Label-free sensing of bacteria and viruses using holography and deep learning (Yuzhu Li, Bijie Bai and Aydogan Ozcan)

### Background

Microorganisms, like bacteria and viruses, play an indispensable role in our ecosystem. While they serve crucial functions, such as facilitating the decomposition of organic waste and signaling environmental changes, certain microorganisms are pathogenic and can lead to diseases like anthrax, tuberculosis, influenza, etc. [128]. The replication of bacteria and viruses can be detected using culture-based methods [129] and viral plaque assays [130], respectively. Though these culture-based methods have the unique ability to identify live and infectious/replicating bacteria and viruses, they are notably time-consuming. Specifically, it usually requires > 24 h for bacterial colonies to form [129] and > 2 days for viral plaques [131] to grow to sizes discernible to the naked eye. In addition, these methods are labor-intensive, and are subject to human counting errors, as experts/microbiologists need to manually count the number of colony-forming units (CFUs) or plaque-forming units (PFUs) within the test plates after the corresponding incubation period to determine the sample concentrations. Therefore, a more rapid and automated method for detecting the replication of bacteria and viruses is urgently needed.

The combination of time-lapse holographic imaging and deep learning algorithms provides a promising solution to circumvent these limitations. Holographic imaging, regarded as a prominent label-free imaging modality, is effective at revealing features of transparent biological specimens by exploiting the refractive index as an endogenous imaging contrast [17, 132]. Consequently, it can be employed to monitor the growth of colonies or viral plaques during their incubation process in a label-free manner. This allows for the capture of subtle spatio-temporal changes associated with colony or viral plaque growth, enabling early detection of them when they are imperceptible to human eye. However, the presence of other potential artifacts (e.g., bubbles, dust, and other random features created by the uncontrolled motion of the sample surface) can hinder the accurate detection of true bacterial colonies or viral plaques. To mitigate such false positive events, deep learning algorithms become critical in automatically differentiating these randomly appearing artifacts from true positive events by leveraging the unique spatio-temporal features of CFU or PFU growth. In this chapter, we will present how the integration of time-lapse holographic imaging and deep learning enables the early detection of bacterial colonies or viral plaques in an automated and label-free manner, achieving significant time savings compared to the gold-standard methods [133–135].

### Methodology

**Fig. 19:**
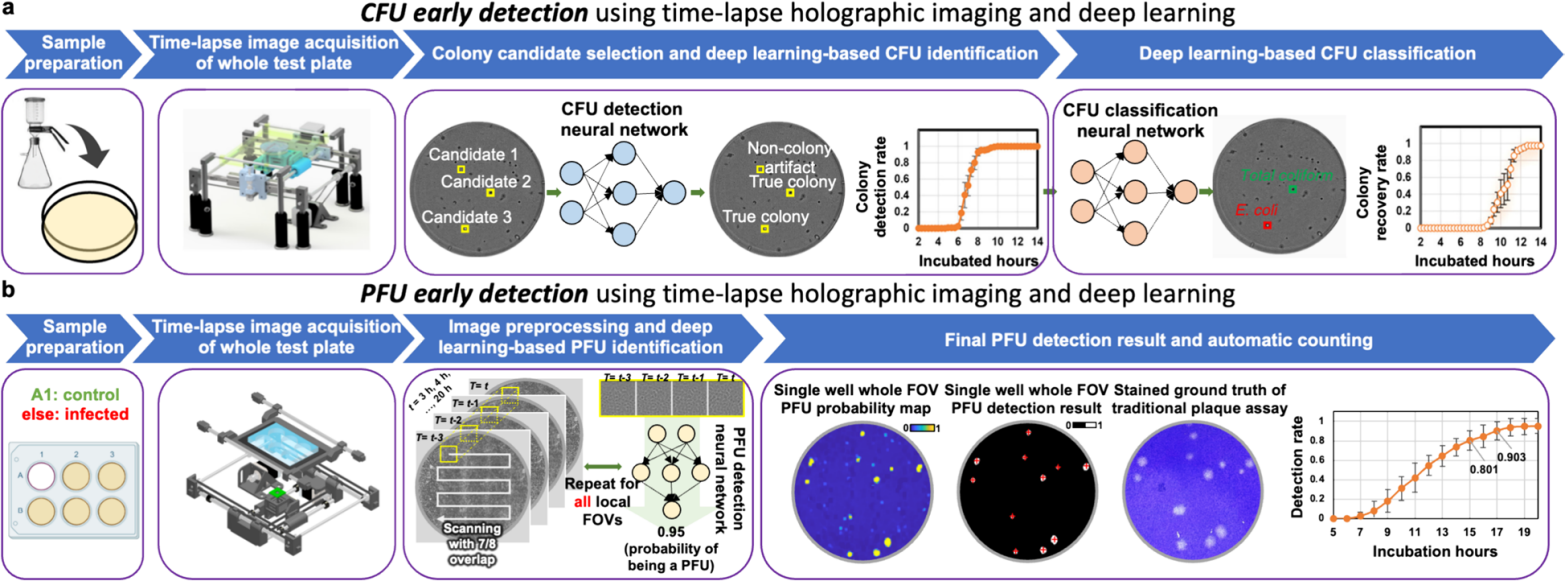
Workflows used for label-free sensing of bacteria (CFUs) and viruses (PFUs) using time-lapse holographic imaging and deep learning. **a** Workflow for CFU early detection using holography and deep learning. **b** Workflow for PFU early detection using holography and deep learning

The primary workflow for detecting CFUs and PFUs using holography and deep learning includes key steps such as time-lapse hologram acquisition of test well plates, digital holographic reconstruction, image processing, and deep learning-based CFU/PFU identification and automatic counting, as illustrated in Fig. 19. The specific methodologies employed in each step differ for CFU and PFU detection, as detailed below.

For the CFU detection [133], as shown in Fig. 19a, after the sample is prepared by inoculating bacteria on a chromogenic agar plate, it is positioned on a customized lens-free [17, 136] holographic microscopy device for time-lapse imaging which utilized a digital in-line holographic microscopy configuration. The sample is illuminated by a coherent laser source, and the resulting holograms are scanned across the entire sample plate by a complementary metal–oxide–semiconductor (CMOS) sensor. These captured time-lapse holograms are digitally stitched and co-registered across various timestamps to mitigate the effects of random shifts in the mechanical scanning process, and digitally reconstructed to retrieve both the amplitude and phase channels of the observed sample plate. Subsequently, a differential analysis-based image processing algorithm is employed to select colony candidates. These candidates are then fed into a CFU detection neural network to identify true colonies from non-colony candidates (e.g., bubbles, dust and other spatial artifacts). Following this, a CFU classification neural network is subsequently employed to classify true colonies identified by the CFU detection network into their specific species. Note that the CMOS image sensor in this workflow can also be replaced by a thin-film-transistor (TFT) image sensor with a much larger imaging field-of-view (FOV) of ∼ 7–10 cm^2^ [134]. In this case, the whole FOV of the sample plate can be captured in a single shot using the TFT image sensor and the obtained holograms are inherently registered across all the timestamps, eliminating the need for mechanical scanning, image stitching, and image registration steps that are used in the CMOS sensor-based system.

For the PFU detection [135], as shown in Fig. 19b, the process of hologram capture and image preprocessing are similar to those used in the CFU detection system. However, the candidate selection and identification procedures are not employed in the PFU detection task. Instead, the reconstructed time-lapse phase images of the whole test well are directly converted into a PFU probability map by applying a PFU detection neural network to the whole well image. This PFU probability map is further converted to a binary detection mask after thresholding by 0.5, revealing the sizes and locations of the detected PFUs at a given time point. The neural networks employed in these studies utilized a DenseNet architecture [137], with 2D convolutional layers replaced by Pseudo3D convolutional blocks [138] to better accommodate time-lapse image sequences. Nonetheless, the network structures suitable for similar work can be changed to more advanced architectures to meet the specific requirements of different detection targets.

### Results

Following the workflows described above, the presented CFU detection system based on the CMOS image sensor showcased its capability to detect ~ 90% of the true colonies within ~ 7.8 h of incubation for *Escherichia col*i (*E. coli*), ~ 7.0 h for *Klebsiella pneumoniae *(*K. pneumoniae*), and ~ 9.8 h for *Klebsiella aerogenes* (*K. aerogenes*) when tested on 336, 339, and 280 colonies for *E. coli*, *K. pneumoniae*, and *K. aerogenes*, respectively. Compared to the gold-standard Environmental Protection Agency (EPA) approved culture-based methods (requiring > 24 h of incubation), this system achieved time savings of > 12 h [133]. As for the TFT sensor-based CFU detection system with simplified hardware and software design [134], its detection time was slightly longer compared to the CMOS image sensor-based system, attributed to the larger pixel size of the TFT sensor (~ 321 μm). When tested on 85 *E. coli* colonies, 114 *K. pneumoniae*, and 66 *Citrobacter* colonies, this TFT sensor-based CFU detection system achieved ~ 90% detection rate within ~ 8.0 h for *E. coli*, ~ 7.7 h of incubation for *K. pneumoniae*, and ~ 9.0 h for *Citrobacter*.

Regarding the automated colony classification task, the CMOS sensor-based CFU detection system correctly classified ~ 80% of all the colonies into their species within ~ 8.0 h, ~ 7.6 h, and ~ 12.0 h for *E. coli*, *K. pneumoniae*, and *K. aerogenes*, respectively. In contrast, the TFT sensor-based CFU system was able to classify the detected colonies into either *E. coli* or non-*E. coli* coliforms (*K. pneumoniae *and *Citrobacter*) with an accuracy of > 85% within ~ 11.3 h for *E. coli*, ~ 10.3 h for *K. pneumoniae*, and ~ 13.0 h for *Citrobacter*.

Regarding the PFU detection system, when evaluated on vesicular stomatitis virus (VSV) plates (containing a total of 335 VSV PFUs and five negative control wells), the presented PFU detection system was able to detect 90.3% of VSV PFUs at 17 h, reducing the detection time by > 24 h compared to the traditional viral plaque assays that need 48 h of incubation, followed by chemical staining—which was eliminated through the label-free holographic imaging of the plaque assay. Moreover, after simple transfer learning, this method was demonstrated to successfully generalize to new types of viruses, i.e., herpes simplex virus type 1 (HSV-1) and encephalomyocarditis virus (EMCV). When blindly tested on 6-well plates (containing 214 HSV-1 PFUs and two negative control wells), it achieved a 90.4% HSV-1 detection rate at 72 h, marking a 48 h reduction compared to the traditional 120-h HSV-1 plaque assay. For EMCV, a detection rate of 90.8% was obtained at 52 h of incubation when tested on 6-well plates (containing 249 EMCV PFUs and two negative control wells), achieving 20 h of time-saving compared to the traditional 72-h EMCV plaque assay. Notably, across all detection time points, there were no false positives detected for all the test wells.

### Conclusion and future perspectives

By leveraging deep learning and holography, the CFU and PFU detection systems discussed in this chapter achieved significant time savings compared to their gold-standard methods. The entire detection process was fully automated and performed in a label-free manner—without the use of any staining chemicals. We believe these automated, label-free systems are not only advantageous for rapid on-site detection but also hold promise in accelerating bacterial and virological research, potentially facilitating the development of antibiotics, vaccines, and antiviral medications.

## Accelerating computer-generated holography with sparse signal models (David Blinder, Tobias Birnbaum, Peter Schelkens)

### Background

Computer-generated holography (CGH) comprises many techniques to simulate light diffraction for holography numerically. CGH has many applications for holographic microscopy and tomography [22], display technology [139], and especially for computational imaging [140]. CGH is computationally costly because of the properties of diffraction: every point in the imaged or rendered scene will emit waves that can affect all hologram pixels. That is why a multitude of algorithms have been developed to accelerate and accurately approximate these calculations [141].

One particular set of techniques of interest is *sparse CGH algorithms*. These encode the wavefield in a well-chosen transform space where the holographic signals to be computed are sparse; namely, they only require a small number of coefficient updates to be accurate. That way, diffraction calculations can be done much faster, as only a fraction of the total coefficients will be updated. Examples include the use of the sparse FFT [142], wavefront recording planes that express zone plate signals in planes close to the virtual object, resulting in limited spatial support, and coefficient-shrinking methods such as WASABI relying on wavelets [143].

A transform that has been especially effective in representing holographic signals is the Short-time Fourier transform (STFT). Unlike the standard Fourier transform, the STFT determines the frequency components of localized signal sections as it changes over time (or space). One important reason for its effectiveness in holography is that the impulse response of the diffraction operator is highly sparse in phase space, expressible as a curve in time–frequency space [144]. This has shown to be effective for STFT-based CGH with coefficient shrinking [144] and the use of phase-added stereograms [145, 146].

Recently, the Fresnel diffraction operator itself was accelerated using Gabor frames, relying on the STFT [147]. This resulted in a novel Fresnel diffraction algorithm with linear time complexity that needs no zero-padding and can be used for any propagation distance.

### Methodology

The Fresnel diffraction operator expresses light propagation from a plane z = z_1_ to z = z_2_ by

4 $$U\left( {x,y;\,z_{2} } \right) = \frac{{e^{ikd} }}{ikd}\int {\int_{ - \infty }^{ + \infty } {U\left( {\xi ,\eta ;\,z_{1} } \right)\exp \left( {\frac{ik}{{2d}}\left[ {\left( {x - \xi } \right)^{2} + \left( {y - \eta } \right)^{2} } \right]} \right)d\xi d\eta ,} }$$

relating the evolving complex-valued amplitude $U$ over a distance $d={z}_{2}-{z}_{1}$, with wavelength $\lambda$, wavenumber $k=\frac{2\pi }{\lambda }$ and imaginary unit $i$. Because this integral is separable along $x$ and $y$, we can focus on the operation only along $x,$ namely

5 $$U\left(x;{z}_{2}\right)={\int }_{-\infty }^{+\infty }U\left(\upxi ;{z}_{1}\right)\text{exp}\left(\frac{ik}{2d}{\left(x-\upxi \right)}^{2}\right)d\upxi ={\int }_{-\infty }^{+\infty }U\left(\xi ;{z}_{1}\right)\mathcal{K}\left(\text{x}-\upxi \right)d\xi ,$$

where $\mathcal{K}$ is the Fresnel convolution kernel. This expression can be numerically evaluated with many techniques [146], but essentially boil down to either spatial convolution or frequency domain convolution using the FFT. We proposed a third approach via *chirplets*:

6 $$\mathcal{G}=\left\{\hspace{0.17em}\text{exp}\left(\alpha {t}^{2}+\beta t+\gamma \right)\hspace{0.17em}|\hspace{0.17em}\alpha ,\beta ,\gamma \in {\mathbb{C}}\hspace{0.17em}\wedge \hspace{0.17em}\mathfrak{R}\left(\alpha \right)<0\right\}$$

which is a generalization of Gaussians with complex-valued parameters. The set of chirplets G is closed under multiplication and convolutions; consider two chirplets $\text{u}=\text{exp}\left(a{t}^{2}+bt+c\right)$, $\widehat{u}=\text{exp}\left(\widehat{a}{t}^{2}+\widehat{b}t+\widehat{c}\right)$; we have that, $\forall u,\widehat{u} \in \mathcal{G},$

7 $$u\cdot \widehat{u}=\text{exp}\left(\left(a+\widehat{a}\right){t}^{2}+\left(b+\widehat{b}\right)t+\left(c+\widehat{c}\right)\right)\in \mathcal{G},$$

and

8 $$u*\widehat{u}=\sqrt{\frac{-\pi }{a+\widehat{a}}}\text{exp}\left(\frac{a\widehat{a}}{a+\widehat{a}}{t}^{2}+\frac{a\widehat{b}+\widehat{a}b}{a+\widehat{a}}t-\frac{{\left(b-\widehat{b}\right)}^{2}}{4\left(a+\widehat{a}\right)}+c+\widehat{c}\right)\in \mathcal{G}.$$

Because the Fresnel convolution kernel $\mathcal{K}$ can be seen as a degenerate chirplet (where α is purely imaginary), any Chirplet that gets multiplied or convolved with $\mathcal{K}$ will also result in a chirplet. Finally, chirplets can be integrated over as follows:

9 $$\forall u\in \mathcal{G}: {\int }_{-\infty }^{+\infty }u\left(t\right)dt=\sqrt{\frac{\pi }{-a}}\text{exp}\left(c-\frac{{b}^{2}}{4a}\right).$$

Thus, if we express the holographic signal in both source and destination planes in terms of chirplets, we can analytically relate the output chirplet coefficients in the plane z = z_2_ as a function of their inputs from the plane z = z_1_.

**Fig. 20:**
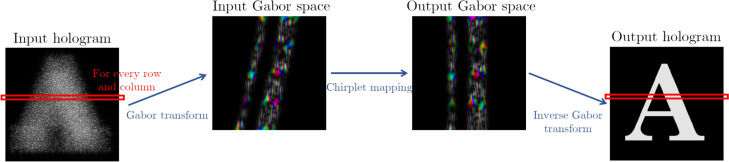
Chirplet-based Fresnel transform pipeline. Every row and column can be processed independently thanks to the separability of the Fresnel transform. The Gabor coefficients can be processed with the chirplet mapping, and transformed back to obtain the propagated hologram

A Gabor frame with Gaussian windows can serve as a representation of a signal by a collection of chirplets, using the STFT [148]. This means we can use a Gabor transform to obtain the chirplet coefficients, transform them using the aforementioned equations, and retrieve the propagated signal by applying the inverse Gabor transform, cf. Fig. 20.

### Results

Because of the sparsity of chirplets for holograms, each input Gabor coefficient will only significantly affect a small number of output Gabor coefficients, no matter the distance *d*. Therefore, only a few coefficients need updating while maintaining high accuracy. Combined with the fact that the computational complexity of the Gabor transform is linear as a function of the number of samples, this results in an *O(n)* Fresnel diffraction algorithm.

**Fig. 21:**
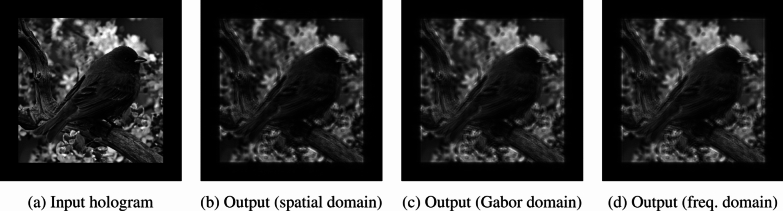
Side-by-side comparison of an example hologram propagated with different algorithms, at *d* = 5 mm. **a** The input hologram, followed by multiple reconstructions, using **b** the spatial domain method, **c** the proposed Gabor domain method and **d** the frequency domain method. The proposed Gabor technique appears to be visually identical to the reference spatial and frequency domain algorithms

We calculated a 1024 × 1024-pixel hologram with a pixel pitch of 4 μm, distance d = 5 cm, and a wavelength λ = 633 nm, cf. Fig. 21. Using a sheared input coefficient neighborhood with a radius of 5 of input coefficients per output coefficient, we obtain a PSNR of 68.3 dB w.r.t. the reference reconstruction. Preliminary experiments on a C +  + /CUDA implementation give a speedup of about 2 to 4 on a 2048 × 2048-pixel hologram compared to FFT-based Fresnel diffraction [149]. The sparsity factor can be chosen to trade off accuracy and speed.

### Conclusion and future perspectives

We have combined two of Dennis Gabor’s inventions, holography, and the Gabor transform, creating a novel method for numerical Fresnel diffraction. It is a new, distinct mathematical way to express discrete Fresnel diffraction with multiple algorithmic benefits. The method requires no zero-padding, poses no limits on the distance *d* and inherently supports off-axis propagation and changes in pixel pitch or resolution. Its sparsity enables a linear complexity algorithm implementation. We plan to investigate these matters and perform detailed experiments in future work. This novel propagation technique may serve as a basis for more efficient and flexible propagation operators for various applications in computational imaging with diffraction.

## Layer-based hologram calculations: practical implementations (Tomoyoshi Shimobaba)

### Background

Holographic displays have attracted significant attention as one of the most promising technologies for three-dimensional (3D) displays. They require optical systems capable of rendering holograms with a large spatial bandwidth, in addition to algorithms and computational hardware that can efficiently compute these holograms at high speeds [139]. Computational algorithms designed for hologram generation can be broadly categorized into several methods, including point cloud, polygon, layer, light field, and deep learning-based approaches. Each method has its own set of advantages and disadvantages, and currently, there is no universally perfect method identified. In this context, we focus on the layer method.

The layer method computes holograms from a 3D scene represented by an RGB image and a depth image (RGB-D image). Depth cameras, such as the Kinect, are now readily available for capturing RGB-D images. Alternatively, RGB-D images can be obtained from computer graphics generated using 3D graphics libraries such as OpenGL. A prototype of a holographic near-eye display utilizing RGB-D images has been successfully developed and has effectively presented 3D images to viewers without causing discomfort [150]. The layer method decomposes a 3D scene into multiple 2D images (referred to as layers) or point clouds, from which holograms can be computed [151]. This chapter provides a detailed exposition of the layer method.

### Methodology

**Fig. 22:**
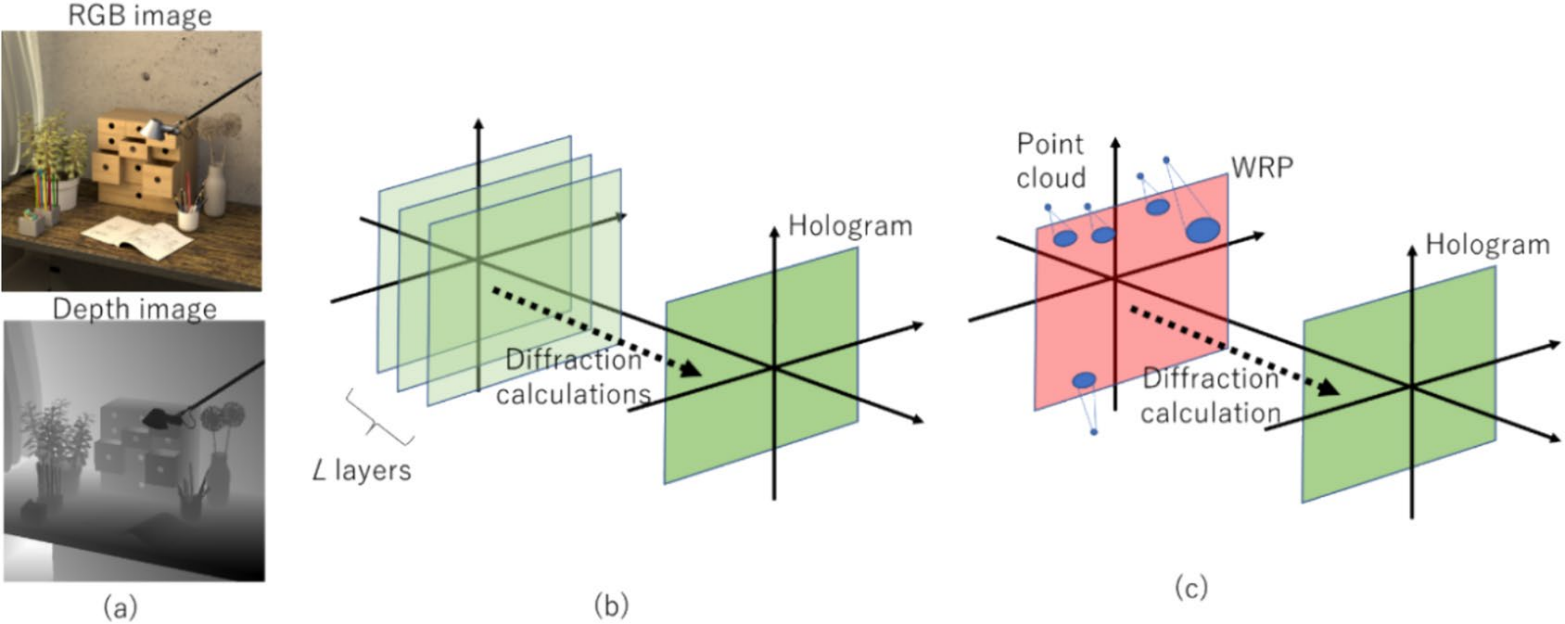
RGB-D image and layer hologram calculation via the layer method: **a** RGB-D image [149], **b** layer hologram calculation by diffraction calculation, **c** layer hologram calculation by point cloud method

A schematic of the hologram computation using the layer method is presented in Fig. 22, wherein an example of RGB and depth images is illustrated. Figure 22b details the process of computing a layer hologram through diffraction calculations. This RGB-D image serves as the basis for decomposing the 3D scene into multiple layers. The light waves emitted from each of these layers are individually computed and subsequently combined on the hologram, resulting in the final hologram [152]. This can be expressed as follows:

10 $$h\left( x \right) = \sum\limits_{{j = 1}}^{L} {\rm{\mathcal{P}}_{j} } \{ u\left( x \right)M_{j} \left( x \right)\psi \left( x \right)\} ,$$

where *x* is the position vector, *L* is the number of layers, ${\mathcal{P}}_{\text{j}}$ is the operator representing the diffraction calculation, $u\left(x\right)$ is one channel with RGB images, ${M}_{j}\left(x\right)$ is a function of extracting the *j*-th layer and is set to 1 if a pixel in the depth image $d\left(x\right)$ matches the depth index *j*; otherwise, it is set to 0. It is defined as follows:

11 $$M_{j} \left( x \right) = \left\{ {\begin{array}{*{20}l} {1 \left( {d\left( x \right) = j} \right)} \hfill \\ {0 \left( {otherwise} \right)} \hfill \\ \end{array} } \right.$$

Representative diffraction calculations employed for ${\mathcal{P}}_{\text{j}}$ involve the utilization of Fresnel diffraction and the angular spectrum method. While these calculations can be expedited through convolution using fast Fourier transforms (FFTs), owing to the cyclic nature of convolution, wraparound noise may be introduced into the reproduced image. To mitigate this wraparound issue, when the hologram size is $N \times N$ pixels, the diffraction calculation is extended to $2N \times 2N$ pixels, with the extended region being zero-padded. This extension, however, leads to increased computation time and greater memory usage. To address this challenge, diffraction calculations using pruned FFT and implicit convolution methods have been proposed as means to alleviate this problem [153].

The function *ψ*(*x*) represents an arbitrary phase, and both random and compensation phases are used in layer holograms [154]. The random phase is defined as ${\psi }_{R}\left(x\right)=\text{exp}\left(2\pi in\left(x\right)\right)$, where $i$ is the imaginary unit, and $n\left(x\right)$ is a random number within the range of 0 to 1. While the random phase has the drawback of introducing speckle noise in the reproduced image, it has the benefit of broadening the viewing angle of the reproduced image and regulating the depth of field in the reproduction. The compensation phase is defined as ${\psi }_{C}\left(x\right)=\text{exp}\left(i\uppi {z}_{j}\right)$, where ${z}_{j}$ represents the distance between the *j*-th layer and the hologram. Phase differences between layers can lead to unwanted diffraction waves (referred to as ringing artifacts) [155] that are superimposed on the reproduced image. The compensation phase serves the purpose of reducing the phase difference between each layer to zero, thereby diminishing ringing artifacts.

Layer holograms can also be computed through the point cloud method, as depicted schematically in Fig. 22c. In this approach, a point cloud is generated from the RGB-D image, and subsequently, the following calculations are executed [156, 157]

12 $$w\left( {\text{x}} \right) = \mathop \sum \limits_{{{\text{l}} = 1}}^{{\text{N}}} {\text{u}}_{{\text{l}}} \exp \left( {{\text{ikr}}_{{\text{l}}} } \right),$$

where ${u}_{l}$ is the *l*-th object point, *k* is the wavenumber, and ${r}_{l}$ is the distance between the object point and a certain point in the hologram. To expedite the computation, a virtual plane, known as the wavefront recording plane (WRP), is positioned in close proximity to the point cloud. The light waves are subsequently recorded on the WRP, denoted as *w*(*x*), using Eq. (12) [156, 157]. Once all the object point information is recorded on the WRP, the hologram can be generated by performing a diffraction calculation from the WRP to the hologram. Layer images often exhibit sparsity. In such instances, using FFTs in Eq. (10) for calculations would be less efficient owing to the presence of layers containing many zeros. If each layer is sparse, it would be more efficient to calculate the hologram by using Eq. (12). The introduction of multiple WRPs is a possibility, and optimal values for the number and placement of these WRPs exist [158].

Holograms obtained using Eqs. (10) and (12) are inherently complex-valued. Typical spatial light modulators (SLMs) are capable of performing either amplitude or phase modulation. Therefore, complex holograms need to be converted into a format suitable for SLMs. In the case of amplitude modulation SLMs, methods are employed that either directly extract the real part of the complex hologram to produce an amplitude hologram or utilize techniques such as single sideband and half-zone plate processing to achieve a complex hologram even when using amplitude modulation SLMs [159, 160]. For phase modulation SLMs, complex holograms are transformed into phase-only holograms through methods such as double phase hologram [161, 162], error diffusion method [163], binary amplitude encoding [164], and bleached hologram [165].

### Results

**Fig. 23:**
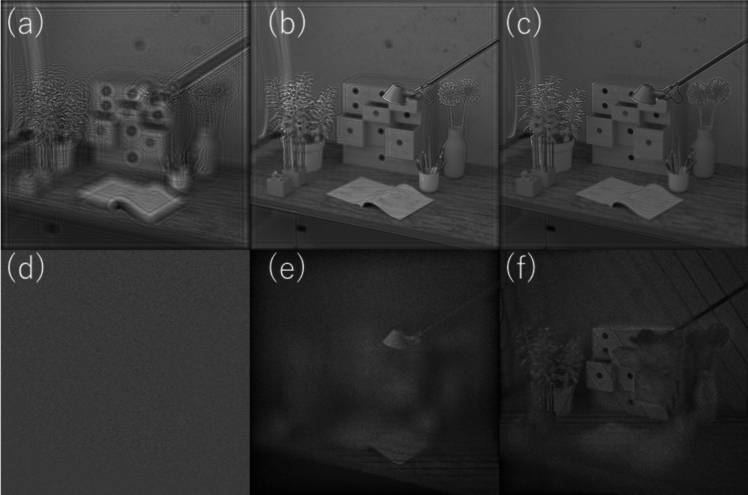
Layer holograms and reproduced images. **a** Hologram using compensation phase, **b** reproduced image focused on standlight, **c** reproduced image focused on shelf, **d** hologram using random phase, **e** reproduced image focused on standlight, **f** reproduced image focused on shelf. The contrast and brightness of each reproduced image were adjusted for ease of viewing

The results of computing the layer hologram from the RGB-D image in Fig. 22a using Eq. (10) are displayed in Fig. 23. For these diffraction calculations, the angular spectrum method was applied [166]. The following parameters were used: a wavelength of 532 nm, a minimum distance of 50 mm between the hologram and the 3D scene, a pixel pitch of 3.74 µm, a thickness of 5 mm for the 3D scene (where a zero-pixel value in the depth image corresponds to a distance of 50 mm from the hologram, and a pixel value of 255 represents a distance of 55 mm), and a total of 32 layers.

Figure 23a shows the hologram utilizing the compensation phase for *ψ(x)* is presented. Further, Fig. 23b and c show the reproduced images derived from the hologram. Figure 23b displays the reproduced image with a focus on the standlight, while Fig. 23c exhibits the reproduced image with emphasis on the shelf. Figure 23d illustrates the hologram created using the random phase for *ψ(x)*. Correspondingly, Fig. 23e and f reveal the reproduced images from the hologram, with a specific focus on the standlight and the shelf, respectively. Notably, the reproduced image originating from the hologram utilizing the compensation phase manifests a deep depth of field, whereas the reproduced image obtained from the hologram using the random phase exhibits a shallow depth of field. Furthermore, the reproduced image of the random phase hologram displays a pronounced presence of speckle noise.

### Conclusion and future perspectives

This section describes the calculation of layer holograms, and the Python code with accompanying comments can be found in the supplementary material. The layer holograms discussed here are commonly applied in near-eye holographic displays [150]. However, this method may not be suitable for holographic displays with broad viewing angles and expansive fields of view, where the observer's eye position can be freely adjusted [167]. For such holographic displays, an alternative approach involves calculating layer holograms using multi-view images in conjunction with depth images [168]. Many hologram computations using deep learning methods also involve inferring layer holograms from RGB-D images. The layer hologram calculations presented in this chapter, serve a valuable purpose in generating training datasets for deep learning. Additionally, while layer holograms derived through deep learning may face challenges in achieving deep-depth reproduction images [169], the computational approach introduced in this chapter allows for greater flexibility in setting the depth parameter. Commented Python code for implementing layer hologram calculation is given in the supplementary materials S9.

## Learned compressive holography (Vladislav Kravets and Adrian Stern)

### Background

In [170] we introduced Compressive Fresnel Holography (CFH)—a technique that uses only a small subset of samples of the hologram to capture the object’s three-dimensional information. The CFH was built upon the Compressive Sensing (CS) [171–173] theory, which states that objects that are sparse or have a sparse representation in some mathematical domain can be economically sampled and reconstructed by employing an appropriate sampling scheme and reconstruction algorithm. Using this technique, a compression ratio of up to 12.5:1 was demonstrated in [170] for Fresnel coherent digital holography. The method was extended for incoherent holography in [174, 175] facilitating the sensing effort by an order of magnitude. Theoretical guarantees for CFH were derived in [176], and comprehensive sampling conditions are summarized in Chapter 9 in [173].

The CS theory considers a linear sensing model described as $\mathbf{g}={\varvec{\Phi}}\mathbf{f}$ where $\mathbf{f}\in {\mathbb{C}}^{{\varvec{N}}}$ represents the objects, $\mathbf{g}\in {\mathbb{C}}^{{\varvec{M}}}$ the measured samples and ${\varvec{\Phi}}\in {\mathbb{C}}^{{\varvec{M}}\times {\varvec{N}}}$ is the sensing matrix. In CS *M *< *N*. There are two common types of sensing matrices ${\varvec{\Phi}}$: Random Modulators (RM) and Partial Random Ensemble (PRE). The RM sensing matrix is an M by N random matrix with entries commonly drawn from a sub-Gaussian distribution (e.g., Gaussian Bernoulli, etc.). The best-known representative of PRE is the Random Partial Fourier (RPF) sensing matrix, which is constructed by randomly picking out M rows from a Fourier Basis. The CFH sensing model relates to this method, suggesting randomly sampling only *M* samples from a full Fresnel transformation ensemble, as we demonstrated in [170]. We further have shown that it is advantageous to sample Fresnel holograms randomly according to a non-uniform pattern [170]. Similar non-uniform CS sampling was also studied for other CS settings (e.g., [177, 178]) using theoretical analysis of the sensing matrix. In this chapter, we present a data-driven deep learning method to determine the optimal random-like sampling. We apply the recently introduced LPTnet [179] to choose the optimal Fresnel samples and to reconstruct the object from the CS samples. LPTnet was demonstrated in [179] to push the classical CS limits by almost two orders of magnitudes when applied to regular 2D imaging. Here, we demonstrate its usefulness for CFH.

**Fig. 24:**
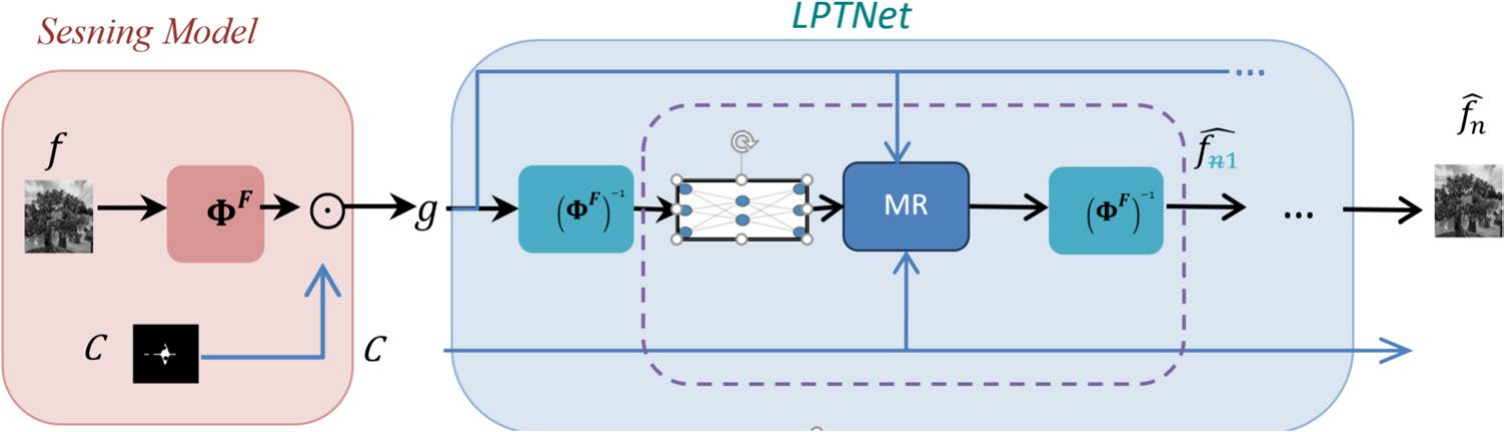
Schematic description of the learned compressive Fresnel holography: LPTnet is used to determine the hologram sampling point and the parameters of the reconstruction deep neural network

LPTnet is an innovative CS framework that utilizes end-to-end Deep Learning (DL) for jointly optimizing the sensing process and the CS reconstruction algorithm. Unlike traditional CS methods that often rely on random sampling techniques, LPTnet intelligently selects optimal samples from a predetermined transformation ensemble using deep learning. Figure 1 shows a simplified scheme of LPTNet. The sensing process is modeled as Hadamard (pointwise) multiplications of the fully transformed image with a binary mask, that is, ${\mathbf{g}={\varvec{\Phi}}}^{{\varvec{F}}}\mathbf{f}\circ \mathbf{c}$, where ${{\varvec{\Phi}}}^{{\varvec{F}}}$ denotes the non-sampled (typically unitary) transform, and **c** the sampling mask. The nonzero values of **c** serve as indicators of the transformed values to be selected. The zero values of the mask **c** effectively null out the fully transformed values, leaving only the partial transformed ensemble ${\varvec{\Phi}}\mathbf{f}$, representing the compressed measurments, **g**. During the training phase, optimization is performed on a reconstruction scheme along with the selection map **c**. In the reconstruction phase, an inverse transform ${\left({{\varvec{\Phi}}}^{\mathbf{F}}\right)}^{-1}$ is first applied on the measurements, **g**. Due to the partial sampling, the obtained image is highly distorted; therefore, a sequence of refinement iterations is performed. In each iteration (dashed box in Fig. 1), a deep neural network is first applied to reconstruct the image. The estimated image is then converted to the transformed domain, and the real measurements are reinforced while leaving the (implicitly) inferred missing coefficients (MR block in Fig. 24). Finally, the data is transformed back ${\left({{\varvec{\Phi}}}^{{\varvec{F}}}\right)}^{-1}$ to the image domain. A detailed description of LPTNet architecture and training process can be found in [179].

### Methodology

**Fig. 25:**
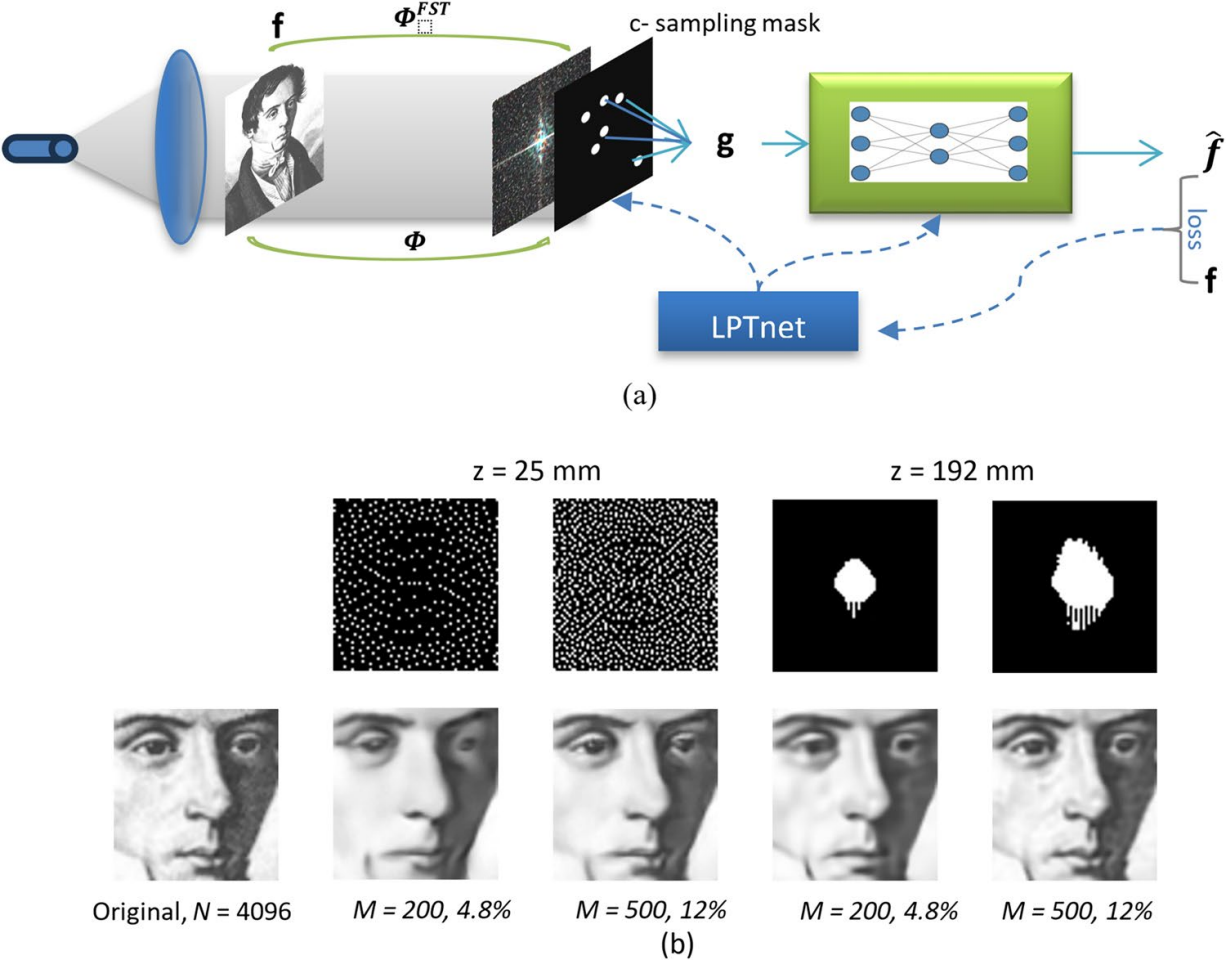
**a** Schematic description of the learned compressive Fresnel holography: LPTnet is used to determine the hologram sampling point and the parameters of the reconstruction DNN. **b** Learned hologram sub-sampling map and reconstruction with LPTNet. The top row displays learned sampling maps for 200 samples (4.8% compression rate) and 500 samples (12% compression rate) at *z* = 25 mm and *z* = 192 mm. The bottom row shows the respective hologram reconstructions

The schematic of LPTnet-based compressive Fresnel holography is shown in Fig. 25a. The relation between the object complex field amplitude, *f*, and the field at the image plane is mathematically described in the far field regime [173] by:

13 $$g(p\Delta _{z} ,q\Delta _{z} ) = \exp \left\{ {\frac{{j\pi }}{{\lambda z}}\left( {p^{2} \Delta _{z}^{2} + q^{2} \Delta _{z}^{2} } \right)} \right\}\rm{\mathcal{F}}_{{2D}} \left[ {f(k\Delta _{0} ,l\Delta _{0} )\exp \left\{ {\frac{{j\pi }}{{\lambda z}}\left( {k^{2} \Delta _{0}^{2} + l^{2} \Delta _{0}^{2} } \right)} \right\}} \right],$$

where ${\mathcal{F}}_{2D}$ is the 2D Fourier transform, $\lambda$ is the illumination wavelength, ${\Delta }_{z}$ is the sensor pixel size, the object sampling interval is ${\Delta }_{0}=\lambda z/\left({n\Delta }_{z}\right)$ , and $0\le p,q,k,l\le n-1$. Assuming no loss of generality, we consider the complex field amplitude image to be of size *n* by *n* and $N={n}^{2}$ is the total number of samples. Equation (1) can readily be written in a vector matrix form [173], $\mathbf{g}={{\varvec{\Phi}}}^{FST}\mathbf{f}$, where ${{\varvec{\Phi}}}^{FST}$ describes the full ensemble of Fresnel transformation. A partial sample of transformation, ${\varvec{\Phi}}$, is found by applying the LPTNet [179].

Similar to Fig. 24, we can represent the CS Fresnel transform as $g=c\circ {{\varvec{\Phi}}}^{FST}\left(f\right)$, where ∘ is the Hadamard (point-wise) product and $c\in {\mathbb{R}}^{n\times n}$ is a learned binary sampling map (Fig. 25a) which can be found using the LPTNet [179], and ${{\varvec{\Phi}}}^{FST}$ is the full Fresnel transform ensemble.

### Results

We have simulated CFH with *W* = 30 mm, *n* = 64, *λ* = 550 nm. The top row in Fig. 25b shows the learned sampling maps, *c*, for *z* = 25 mm and for z = 192 mm with *M* = 200 and *M* = 500 samples each. Notice that each case has its own Fresnel transformation ensemble, ${{\varvec{\Phi}}}^{FST}={{\varvec{\Phi}}}^{FST}\left(z,\frac{M}{N}\right)$, therefore LPTnet finds an appropriate sampling map. It can be seen that for short distances, where the Fresnel transformation is not much different from that of the object, the optimal sampling pattern is uniformly random, which is in agreement with the universal CS oracles that do not employ learning tools. However, as the field propagates, the sampling pattern becomes patterned. Figure 25b (center and bottom) demonstrates the image reconstructions of Augustin-Jean Fresnel portrait (Fig. 25b) from as few as *M* = 200 and *M* = 500 samples for *z* = 25 mm for *z* = 192 mm.

### Conclusion and future perspectives

In this study, we introduced Learned Compressive Holography—a method that determines the optimal hologram sampling pattern according to the particular imaging conditions. For this purpose, we utilized the LPTnet framework, which jointly optimizes the sampling pattern and a reconstitution deep neural network via a learning process. We have shown that applying the LPTnet framework to compressive Fresnel holography can enhance image reconstruction from a reduced number of samples. This approach, which aligns with CS principles, has proven effective in reconstructing detailed images with fewer resources. The use of LPTnet to select optimal samples from the Fresnel transform is a notable improvement over traditional random sampling methods. Our results show the potential of this method for efficiently handling sparse data. In the future, we will investigate the use of CFH for other types of coherent and incoherent holography methods.

## Computational optical phase imaging: from digital holographic interferometry to intensity diffraction tomography (Shun Zhou, Jiaji Li, Jiasong Sun, Qian Chen, and Chao Zuo)

### Background

One of the prominent challenges encountered in optical microscopy relates to contrast enhancement. Traditional microscopy relies on the mechanism of intensity-based detection, which necessitates the use of staining agents to visualize transparent specimens, such as biological cells. On the contrary, label-free microscopy has emerged as an ideal method for exploring the physiological activities and long-term dynamic processes of living cells. In 1932, Zernike introduced the technique of phase contrast microscope which utilizes the principle of aperture modulation and spatial filtering, significantly enhancing the contrast for transparent specimens [180]. Nevertheless, while this phase imaging method excels in two-dimensional (2D) qualitative visualization, it has not yet been successfully extended to three-dimensional (3D) quantitative measurements.

Inspired by Zernike’s concepts, various innovative label-free microscopic techniques gradually emerged, among which quantitative phase imaging (QPI) is considered one of the most promising approaches [181]. In particular, 3D optical diffraction tomography (ODT) can be realized by combining QPI with computed tomography, providing true 3D refractive index (RI) distribution inside the sample. The realization of ODT is of great significance for revealing the intrinsic mechanisms of cell biology and pathophysiology. Unfortunately, the measurements of the quantitative phase cannot get rid of laser and optical interference for over half a century. Inherent defects of interferometric detection, such as complex interference devices, speckle noise, and coherent diffraction limit have not been fundamentally resolved for a long time, and these enduring obstacles hinder the widespread applications and long-term future development of interferometric holography in the field of biological imaging.

Over the past decade, we have spearheaded research in computational optical phase imaging domestically and also exerted influence internationally. We are primarily focusing on the theory development of generalized phase definition under partially coherent light field and phase transfer function (PTF), and the technique advances of spatial bandwidth product (SBP) enhancement and intensity diffraction tomography. These efforts contribute to the development of innovative theories and methods for non-interferometric quantitative phase and diffraction tomographic imaging.

### Methodology and results

#### From fully coherent field to partially coherent field

**Fig. 26:**
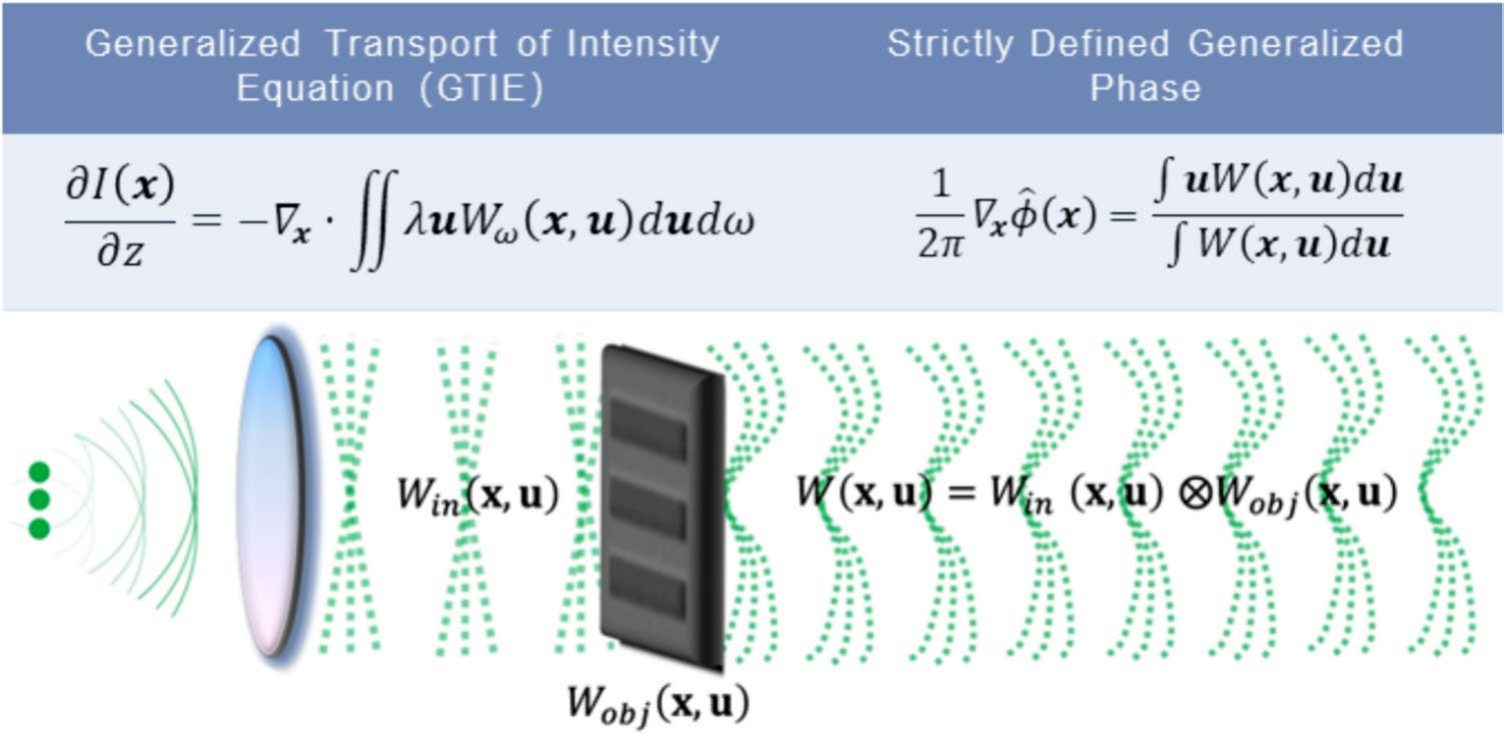
Generalized TIE and generalized phase under partially coherent field derived from the Wigner distribution function in phase space

The scalar diffraction theory proposed by Huygen is sufficient to accurately describe the propagation of the light field in free space and its complex amplitude distribution on an arbitrary plane for the case of a fully coherent illumination light field. The corresponding inverse problem can be solved iteratively by phase retrieval methods such as GS, hybrid input–output algorithm (HIO), or directly by the transport of intensity equation (TIE) under the paraxial approximation [182]. It is worth noting that the intensity forward models relied upon by these phase retrieval techniques assume of fully coherent illumination. However, the partially coherent fields exhibit statistical properties with random fluctuations and cannot be fully described by the 2D complex amplitude, so there is no clear phase definition. To address the above issues, as depicted in Fig. 26, we established the generalized TIE and provided a strict definition of the generalized phase under partially coherent field based on the Wigner distribution function in phase space [183]. The generalized phase serves as a scalar potential function, with its gradient representing the first-order conditional frequency moment of the Wigner distribution function under the partially coherent field, thus extending the well-posedness of TIE from the fully coherent wavefield to the wavefield in any coherent state. Based on Poynting's theorem in the unbounded space, we have strictly proven the existence and uniqueness of the solution to the equation under non-homogeneous Neumann boundary conditions [184]. This achievement effectively addresses a long-standing theoretical problem of obtaining an exact solution to TIE [185], which lays a theoretical foundation for QPI from interference to non-interference and from fully coherent illumination to partially coherent illumination.

#### From coherent diffraction limit to incoherent diffraction limit

**Fig. 27:**
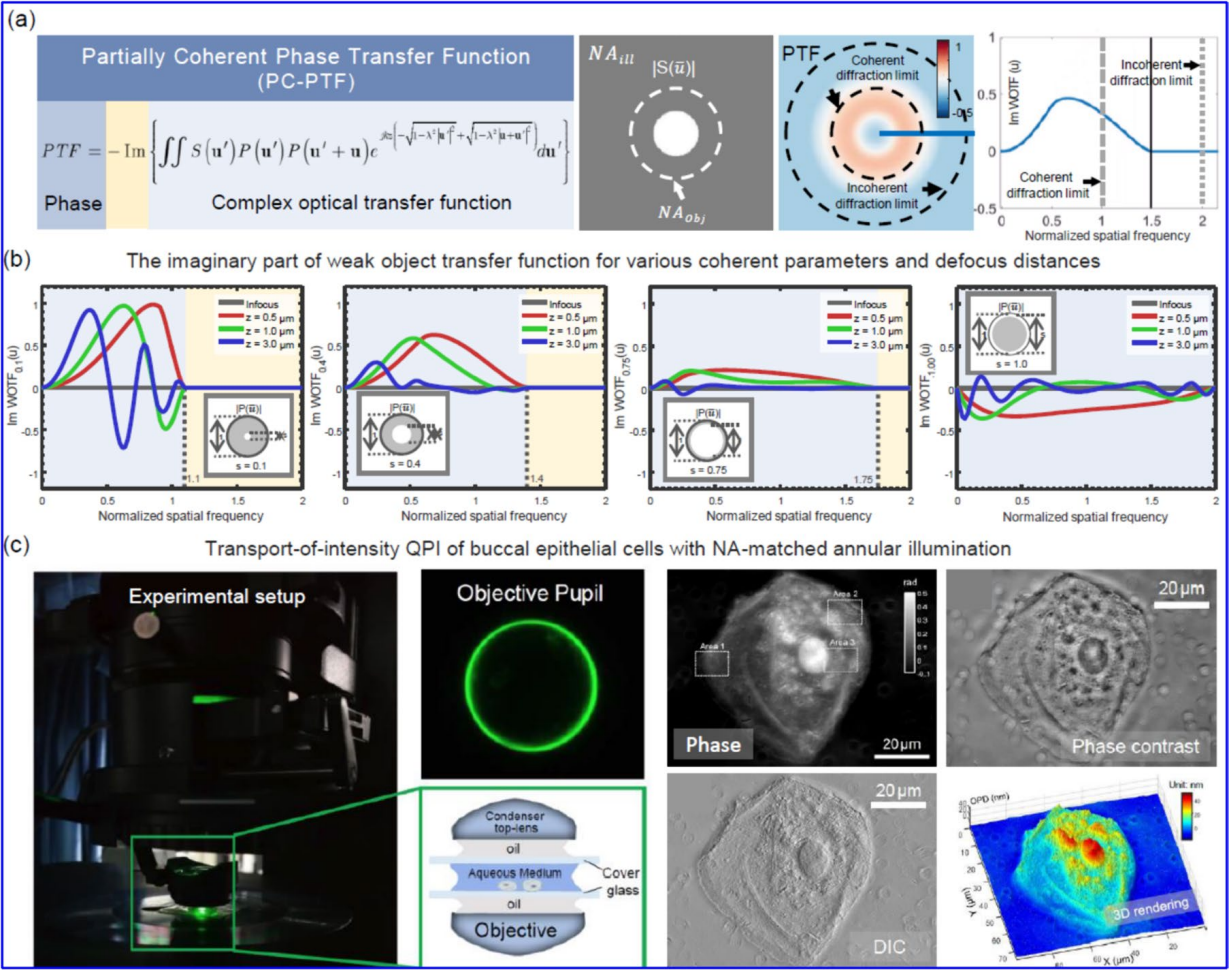
QPI under partially coherent illumination. **a** Partially coherent phase transfer function. **b** The imaginary part of weak object transfer function for various coherent parameters and defocus distances. **c** High-resolution imaging of buccal epithelial cells based on transport-of-intensity quantitative phase microscopy with annular illumination

Extending QPI technique from fully coherent to partially coherent illumination also offers the benefit of enhancing imaging resolution thanks to the inherent synthetic aperture. Classical Fourier optics theory reveals that the incoherent optical transfer function (OTF) is the normalized autocorrelation of the coherent transfer function (also known as pupil function), resulting in a cutoff frequency twice that of the coherent diffraction limit. However, this conclusion cannot quantitatively describe the phase imaging characteristics under partially coherent illumination that lies between fully coherent and incoherent. More importantly, as coherence decreases, the incoherent imaging system degrades to a linear system of intensity and loses its capability to image phase objects. To address these challenges, we have developed the PTF theory under partially coherent illumination by separating the contribution of the specimen and system within the image formation process [186] , providing a more nuanced understanding of how these factors interact to produce the final image. The equation and visual representations of the PTF, including 2D cross-sectional illustration and line profile, are shown in Fig. 27a. These illustrations provide a clear visual explanation of imaging resolution enhancement through the use of partially coherent illumination. Moreover, the imaginary part of the weak object transfer function for various coherent parameters s (the ratio of illumination NA to objective NA) and defocus distances are illustrated in Fig. 27b. The PTF theory not only reveals the trade-off between cutoff frequency and response amplitude under traditional circular illumination apertures but also demonstrates that annular illumination matching the objective NA effectively expands the support domain up to twice the cutoff frequency corresponding to the objective NA, while maintaining optimal response [187, 188] . Based on this, we have proposed a transport-of-intensity QPI method that utilizes NA-matched annular illumination, effectively extending the imaging resolution from the coherent diffraction limit to the incoherent diffraction limit. The corresponding experimental setup is depicted in Fig. 27c. The effectiveness of our proposed approach is exemplified by the imaging of subcellular structures within buccal epithelial cells. Figure 27c demonstrates the multi-mode imaging results, encompassing quantitative phase, phase contrast, differential interference contrast (DIC), and pseudo-color 3D rendering. These collectively underscore the superior imaging capability of our method in revealing intricate cellular details that were previously inaccessible with traditional imaging techniques.

#### From defocus phase reconstruction to ptychographic bandwidth expansion

**Fig. 28:**
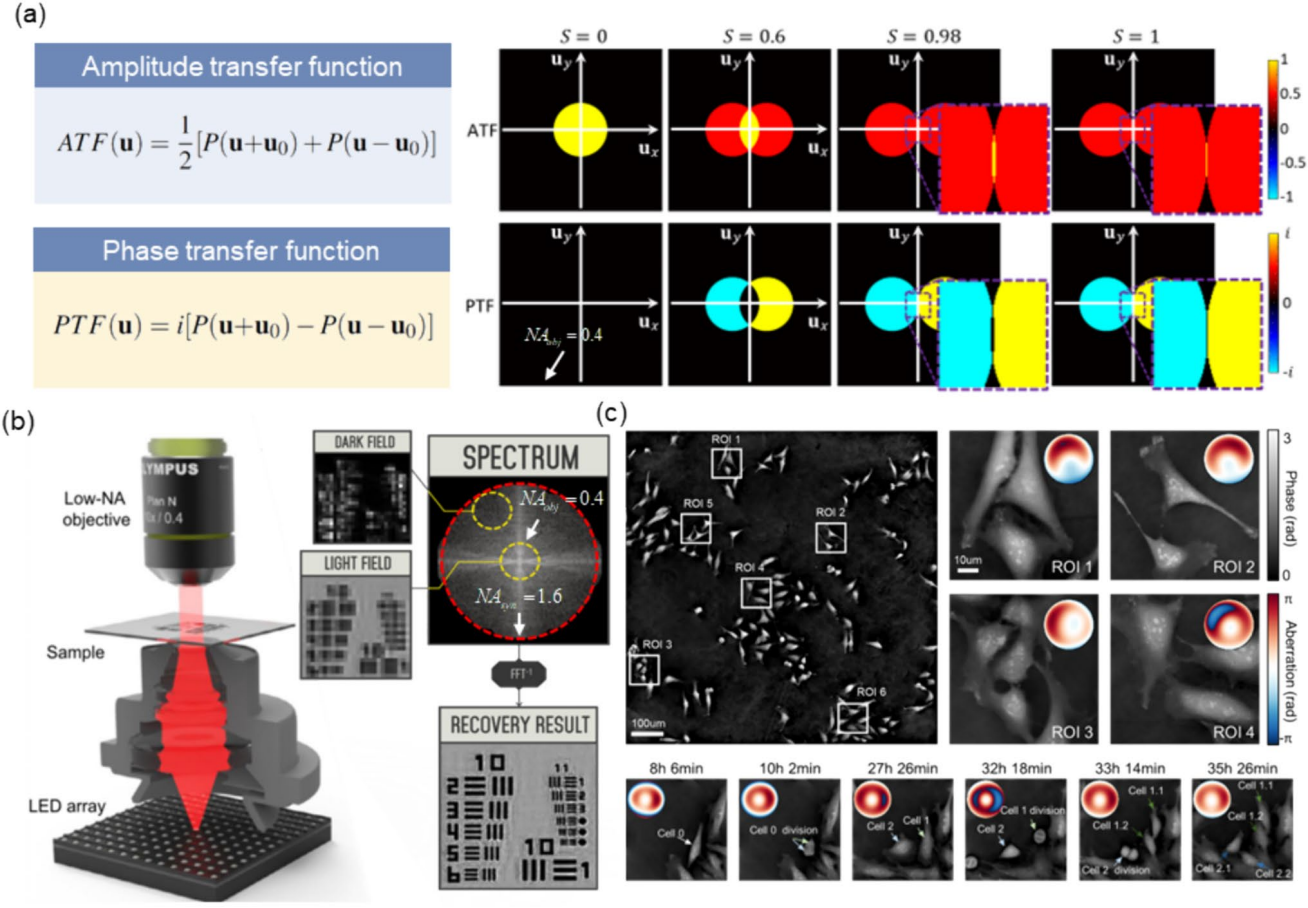
Wide-field, high-resolution FPM. **a** OTF under asymmetric coherent illumination. **b** Resolution-enhanced FPM based on high-numerical-aperture illuminations. **c** High-speed, long-time, and adaptive-aberration-correction high-throughput phase imaging based on annular illumination FPM

To achieve QPI, it is crucial to introduce imaginary components into the complex function of the OTF under partially coherent illumination. In TIE, this is accomplished by introducing defocus into the imaging system (complex pupil function). Another method involves breaking the radial symmetry of the imaging system by employing asymmetric illumination or asymmetric aperture, such as differential phase contrast (DPC) and Fourier ptychographic microscopy (FPM) [189]. With this approach, we derived the PTF under asymmetric illumination, as depicted in Fig. 28a, which reveals the intrinsic relationship between the illumination/detection numerical aperture ratio and the imaging SBP in the FPM. By applying the large illumination NA to a low-magnification objective, the imaging system’s SBP can be expanded by utilizing the multiple differences between the illumination NA and the objective NA [190, 191]. In Fig. 28b, we built an FPM system based on a high-NA programmable condenser, achieving super-resolution and high-throughput imaging with an equivalent NA of 1.6 under the large field of view of a 10 × objective using oil-immersed condenser illumination. Additionally, our research has revealed that FPM is constrained by matched illumination condition and proposed a high-speed FPM approach based on annular illumination, enabling high-speed, long-time, and adaptive-aberration-correction high-throughput phase imaging [192, 193], as illustrated in Fig. 28c.

#### From 2D phase imaging to 3D tomographic imaging

**Fig. 29:**
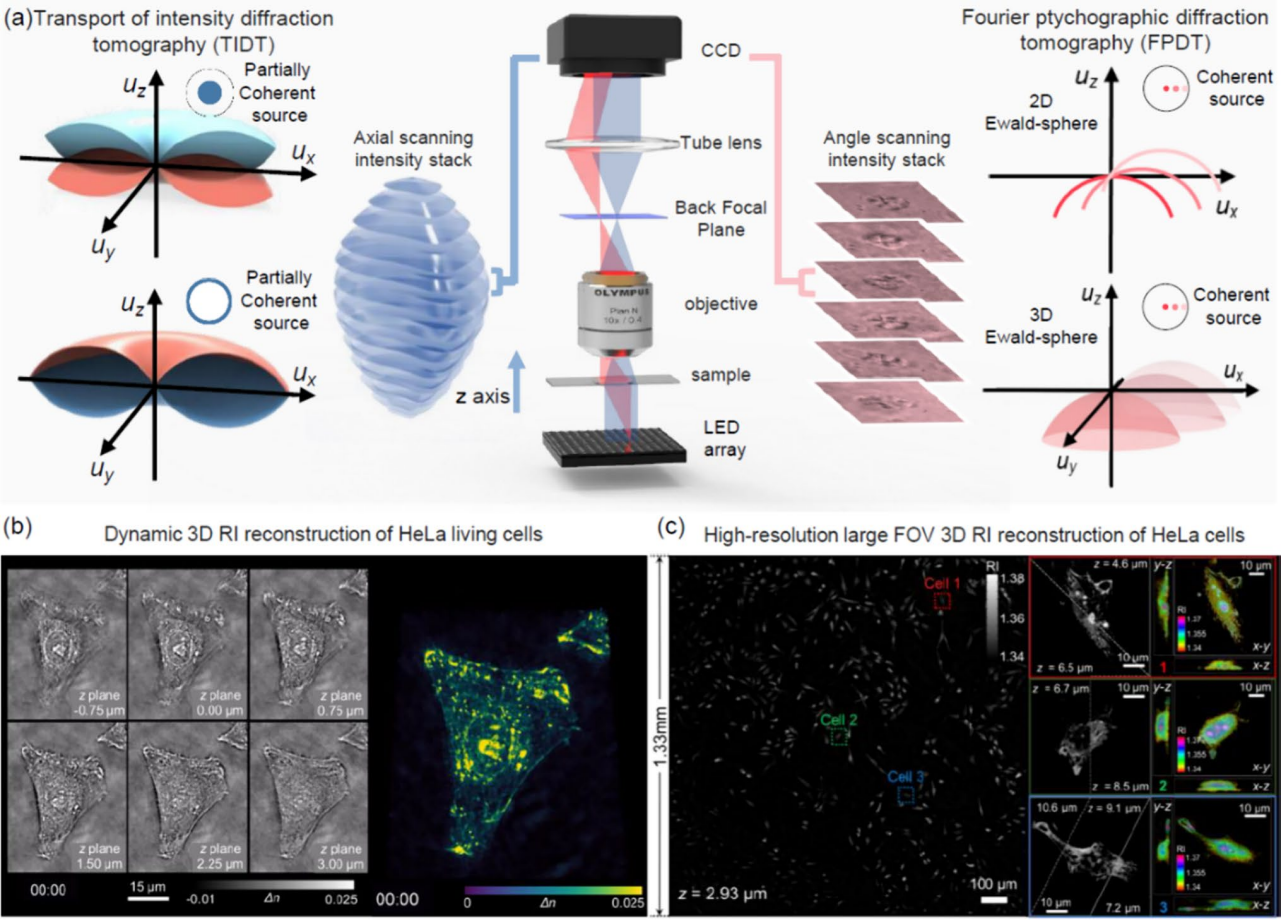
Label-free 3D microscopy based on IDT. **a** Schematic of TIDT and FPDT. **b** Dynamic 3D RI imaging of HeLa live cells using TIDT. **c** High-resolution, large field of view 3D RI imaging of HeLa cells using FPDT

Although the approaches of TIE and FPM effectively eliminate the defect of QPI based on interference, the persistence of the divide-and-conquer thought of “phase recovery followed by diffraction tomography” still constrains the realization of 3D tomography. In the past five years, our research focus has gradually shifted from phase imaging to diffraction tomography, venturing into a new class of non-interferometric label-free 3D microscopic imaging techniques—intensity diffraction tomography (IDT). IDT incorporates the principles of “phase retrieval from intensity” and “RI reconstruction from phase”, bypassing the intermediate step of “phase measurement”. It allows for the direct reconstruction of 3D RI distribution exclusively using the intensity information generated by illumination angle scanning or axial scanning of the sample. As depicted in Fig. 29a, IDT can be categorized into two main implementations: transport of intensity diffraction tomography (TIDT) based on axial scanning [186, 194] and Fourier ptychographic diffraction tomography (FPDT) based on illumination angle scanning [195, 196]. Specifically, TIDT expands the 2D plane intensity transmission of TIE to 3D volume transmission, achieving parallelized coverage of the object's 3D scattering potential spectrum through the use of partially coherent illumination. TIDT first records the intensity image stack of the sample’s scattered field at different axial positions under partially coherent illumination, and then performs 3D deconvolution based on the 3D phase optical transfer function corresponding to the imaging system to obtain the 3D RI distribution information of the sample. This method enables label-free 3D imaging with a lateral resolution of 206 nm and an axial resolution of 520 nm under a high NA oil immersion objective, and the dynamic 3D RI imaging results of HeLa live cells are shown in Fig. 29b. On the other hand, FPDT expands the 2D plane aperture ptychography of FPM to 3D volume ptychography and establishes the intensity forward model under both bright- and dark-field illumination based on the first-order Born and Rytov approximation, respectively. A 3D spectrum updating model is further built based on the quantitative relationship between the scattering potential of the sample and the recorded intensity. FPDT uses a low-NA objective to acquire a sequence of intensity images corresponding to different illumination angles scanned sequentially with a programmable light-emitting-diode array. Then, this method gradually combines these intensity images into a 3D spectrum of the object using a ptychographic reconstruction algorithm. After the convergence of the algorithm, an inverse Fourier transform is performed to obtain the sample’s 3D RI distribution. By employing high-NA dark-field illumination, FPDT achieves high-throughput label-free 3D diffraction tomography with a lateral resolution of 390 nm and an axial resolution of 899 nm across a 10 × FOV of 1.77 mm^2^ and a depth of focus of ~ 20 μm. The high-resolution and large FOV 3D RI imaging results of HeLa cells are shown in Fig. 29c, which contains nearly 4000 Hela cells.

### Conclusion and future perspectives

The emergence of computational optical quantitative phase imaging and intensity diffraction tomography means rigorous coherence and interferometry are no longer prerequisites for QPI and ODT, which marks the great progress of label-free microscopic imaging techniques based on phase detection into a novel stage. And these advancements will open up new possibilities for label-free 3D microscopy and are expected to be widely applied in various biomedicine and life sciences. Nonetheless, these techniques still face a series of ongoing challenges and problems permitting further exploration in the future, including the combination of TIDT and FPDT to break through the limit of matched illumination condition [197], the optimization of the forward model for samples with multiple scattering to go beyond the Born and Rytov approximations [198], and the suppression of the missing-cone problem in Ewald sphere spectrum to expand the axial support region [199]. Furthermore, the potential combination of IDT and 3D super-resolution fluorescence microscopy imaging technique holds promise for opening a new window to observe nanoscale details inside living cells at the single-cell and subcellular levels. The breakthrough brought by computational optical phase imaging might provide more valuable insights into applications such as single-cell morphology and dynamics analyses, cellular interactions, cellular responses, and label-free pathology diagnosis. Detailed insights into computational optical quantitative phase imaging and intensity diffraction tomography, including specific theories and methods, are consolidated in [186]. For a deeper comprehension of the MATLAB source codes, readers are encouraged to refer to the details provided in [186] and visit https://scilaboratory.com/code.html.

## Computational hyperspectral quantitative phase imaging from spectrally multiplexed observations (Igor Shevkunov, Vladimir Katkovnik, and Karen Egiazarian)

### Background

We consider a novel setup and a novel computational algorithm for hyperspectral (HS) QPI from total intensity observations, which are the sums of spectral intensities over a wide spectral range. This setup is explicitly based on computational analysis of the observations and does not require any spectral devices, which makes HS imaging simple. This imaging can be applied to non-invasive and label-free sample observations, which is especially valuable for biological and medical laboratories where dying and labeling might harm a specimen [182]. Our HSQPI is an extension of the basic ideas of the phase retrieval techniques [200]. Overall, the phase retrieval problems are ill-posed and require multiple diverse observations for high-quality imaging. This kind of diversity can be achieved in different ways: through varying registration distances [201], multiple phase encoded apertures [202], or sets of wavelengths [203]. The latter case provides hyperspectral modality which in turn provides a broadening of the technique with the imaging in a wide spectral range. Typically, spectral observations for phase retrieval are registered for each spectral channel separately, channel-by-channel, which is realized either by sets of narrow-band filters [203] or by a tunable light source [204]. Contrary to it, we use our recently developed algorithm, named HS Phase Retrival (HSPhR) algorithm [205], which provides hyperspectral phase retrieval in parallel for all spectral channels from the spectrally multiplexed observations. The separation of spectral channels is achieved due to modulation encoding phase-masks and the developed HSPhR, including the original spectral proximity operators and complex-domain alternating direction algorithm of multipliers (ADMM) [206].

**Fig. 30:**
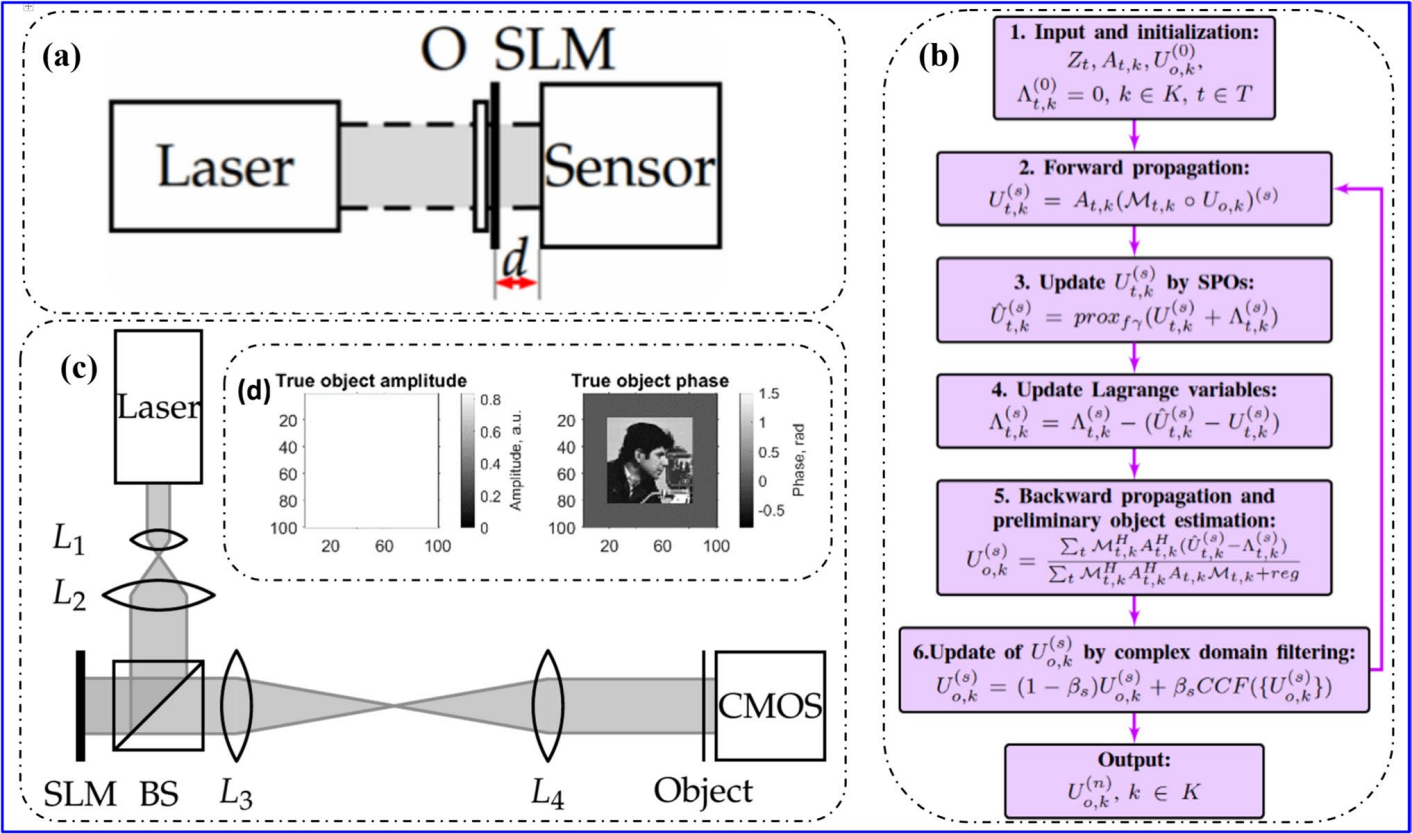
**a** Schematic optical setup corresponding to our tests and data formation model; **b** HSPhR algorithm. **c** Reflective SLM-based experimental setup. The laser is a supercontinuum light source; ${L}_{1},{L}_{2}$ are beam-expanding lenses; BS is a beasplitter; ‘SLM’ is a Spatial Light Modulator; ${L}_{3},{L}_{4}$ are lenses of a 4f-telescopic system, projecting the wavefront from SLM to the object plane shown as ‘Object’, and CMOS is the registration camera. **d** Amplitude and phase of the object on SLM for 744 nm

In the considered setup of the HS phase retrieval (see Fig. 30a), we utilize random phase masks ${\mathcal{M}}_{t,k}\in {\mathbb{C}}^{\text{N}}$ which, along with propagation operator ${\text{A}}_{\text{t},\text{k}}$, encode the spectral property of the object ${U}_{o,k}\in {\mathbb{C}}^{N}$ into the total spectral intensity observations ${Y}_{t}={\sum }_{k\in K}{\left|{A}_{t,k}\left({\mathcal{M}}_{t,k}\circ {U}_{o,k}\right)\right|}^{2}, \, t=1,\dots ,T$, where ${Y}_{t}\in {\mathbb{R}}^{M},$ and ${A}_{t,k}\in {\mathbb{C}}^{M\times N}$ is an image formation operator modeling propagation of 2D object images from the object plane to the sensor, ‘$\circ$’ stands for the element-by-element (Hadamard) product of two vectors. ${U}_{o,k}\in {\mathbb{C}}^{N}$ is the object of interest, where $N=nm$, and $n$ and $m$ are the width and height of 2D image; $k$ stays for the spectral variable, $t$ is a number of the experiment with the total number of experiments $T$. HS phase retrieval is a reconstruction of the complex-valued object ${U}_{o,k}$, *k*$\in K,$ from intensity measurements ${Y}_{t}$. The total intensity ${Y}_{t}$ is calculated over the spectrum range as the sum of the channel spectral intensities. For the noisy case, ${Y}_{t}$ is replaced by ${Z}_{t}={Y}_{\text{t}}+{\upvarepsilon }_{\text{t}}$, where ${\upvarepsilon }_{t}$is the additive noise.

### Methodology

The computational solution is acquired in an iterative approach, where ADMM Lagrange multipliers, ${\Lambda }_{k,t}$, improve its convergence and spectral proximity operators, SPOs, provide noise suppression at the sensor plane, see the algorithm structure in Fig. 30b. As in all phase retrieval iterative loops, the first guess initialization (Step 1) is required, which we assume as 2D random white-noise Gaussian distribution for objects’ phase and a random uniform 2D positive distribution on (0, 1] for amplitude independent for each *k*. Initial Lagrange multipliers ${\Lambda }_{k,0}=0$. The forward propagation is produced for all $k\in K$ and $t$ (Step 2). The update of the wavefront at the sensor plane (Step 3) is produced by the proximal operators. In Step 4, the Lagrange variables are updated. The backward propagation of the wavefront from the sensor plane to the object plane is combined with an update of the spectral object estimate in Step 5. The sparsity-based regularization by Complex Cube Filter (CCF) [207] is relaxed by the weight-parameter 0 < *β*_*s*_ < 1, at Step 6. After fixed number of iterations the outcome is the HS object estimation, ${U}_{o,k}.$

### Results

The optical setup implemented in our physical experiments is shown in Fig. 30c. This phase object and the modulation phase masks ${\mathcal{M}}_{t,k}$ are realized on a spatial light modulator (SLM). The SLM is a GAEA-2 Holoeye, 4160 × 2464 pixels with a pixel size of 3.74 μm. The super-continuum laser source is limited to a range of 550–1000 nm (YSL photonics CS-5). The camera is monochrome Blackfly S, model BFS-U3-200S6M, FLIR, with a pixel size of 2.4 μm. In Fig. 30c, the illumination wavefront expanded by lenses ${ L}_{1}$ and${L}_{2}$ propagates to SLM through the beamsplitter (BS), where SLM changes the wavefront phase distribution according to the object and modulation mask phases. This modulated wavefront is projected to the ‘Object’ plane by the 4f telescopic system, composed from achromatic doublet lenses $L_{3} \, {\text{and}}\, L_{4}$ (with a diameter of 12.7 mm and a focal length of 50 mm). Further, the light beam propagates freely 2 mm to the registration camera ‘CMOS.’

**Fig. 31:**
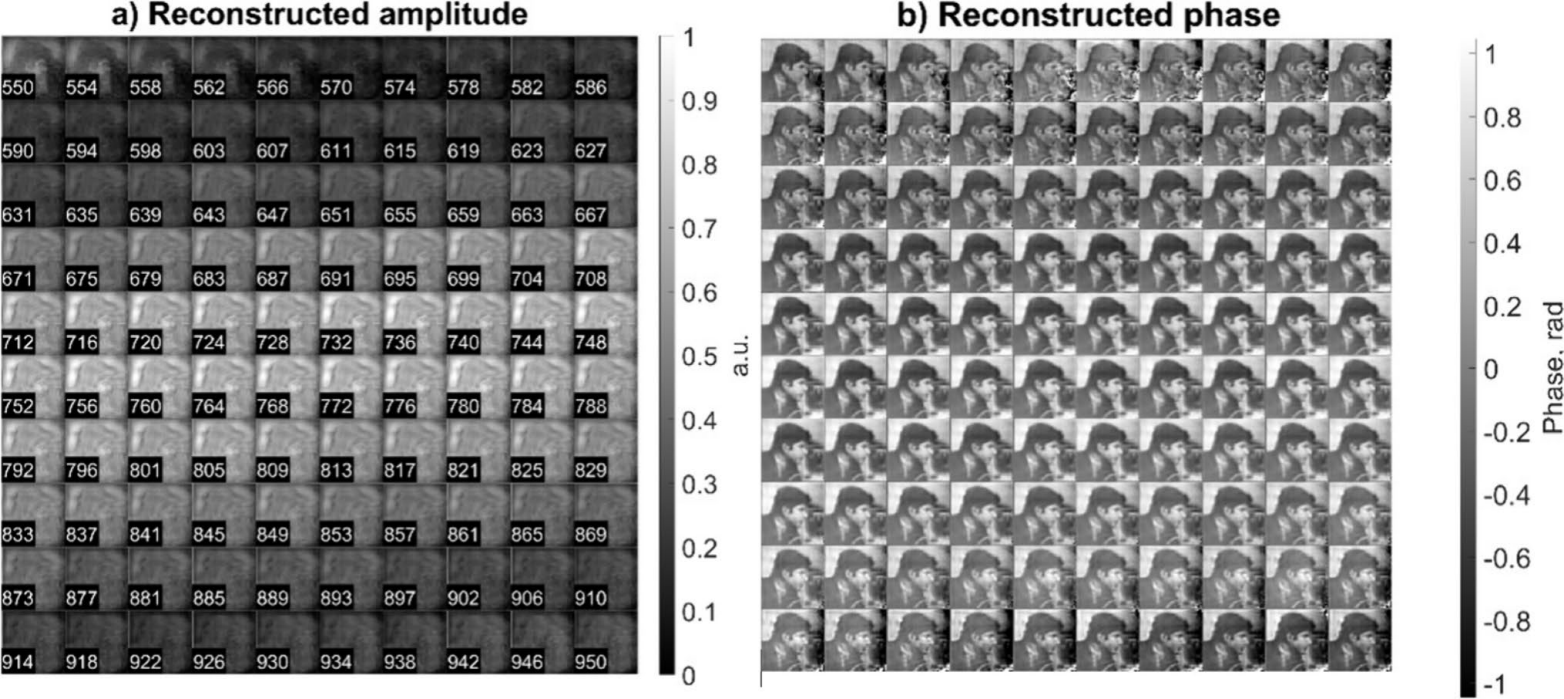
The reconstructed HS amplitudes (**a**) and phases (**b**) of the object. The wavelength number shown in the amplitude images are valid to phase images of the same location

The SLM parameters were chosen to limit phase range of the object (cameraman image, 64 × 64 pixels) to [0: π] rad in the whole spectral range, this phase distribution for wavelength of 744 nm is shown in Fig. 30d. Reconstruction results are demonstrated in Fig. 31, which are done for T = 300 observations and K = 100 wavelengths, SNR of observations was 34 dB. The reconstructed spectral amplitude intensities correspond quite accurately to the spectral distribution of the used laser with the intensity maximum at λ = 750 nm. The spectral phase image quality varies from low to high accordingly to variations of the spectral laser intensity with the best results for the high intensity values.

### Conclusion and future perspectives

The novel computational algorithm for HSPhR from multiplexed total intensity observations has been developed. The multiple random phase masks are used for phase encoding in the object plane. The algorithm is based on a complex domain version of ADMM [206] and the original spectral proximity operators derived for noisy intensity observations. The physical experiments confirm that the algorithm is able to retrieve complex-domain spectral components of the object from the noisy spectrally multiplexed intensity observations. The algorithm does not require constraints conventional for the phase retrieval problem, e.g., such as aperture or/and phase bindings through thickness and refractive index. The proposed approach could be useful in various applications in biomedical imaging, remote sensing, and materials science. The commented MATLAB code is provided in the supplementary materials S10.

## Quantitative phase imaging through spatial convolutions (Jeonghun Oh and YongKeun Park)

### Background

QPI is a label-free imaging technique increasingly employed in biological research [208–210] and preclinical studies [181, 211–213]. It distinguishes itself by leveraging the contrast derived from variations in the refractive index (RI) and thickness of a specimen [214]. Nevertheless, challenges persist in optimizing throughput and enhancing productivity within QPI systems. One such challenge involves the trade-off between spatial resolution and field of view (FOV), dictated by the limited pixel count of detectors. Another issue is that the experimental realization of interferometry results in bulky, complicated, and unstable systems, which are often incompatible with conventional microscopes. Moreover, several areas for improvement in QPI have been identified. These include mitigating the effects of multiple scattering in thick samples [198, 213, 215], minimizing the dependency on light source coherence [27, 216–218], and addressing the lack of molecular specificity [219–222].

Recently, spatial transform techniques have emerged as promising solutions within the QPI domain [223–226]. These methods are intricately connected to the cepstrum concept in Fourier analysis [227, 228] and have their roots in analytical optical research conducted during the 1970s and 1980s [229–232]. Specifically, non-interferometric QPI is gaining attention because it allows the light emanating from the sample field to meet the criteria for Hilbert transform-based techniques by manipulating the Fourier spectrum. In this manuscript, we will explore instances where the spatial convolutions with emphasis on the Hilbert transform have been effectively employed in QPI.

### Applications of Hilbert transform to QPI

An optical field is described by a complex function *f(x)* in one dimension, which means the function *f(z)* whose domain is restricted to the real axis. From the property of complex logarithmic functions, the principal logarithm of *f(z)* is represented in the real axis as follows:

14 $${\text{Log}}\left[ {f(x)} \right] = \ln \left| {f(x)} \right| + i{\kern 1pt} {\text{Arg}}\left[ {f(x)} \right],$$

where Arg denotes the principal argument. The real and imaginary parts are related only to the intensity and phase of *f(x)*, respectively. If *f(z)* has zeros in at most one half-plane, the imaginary part of Log[*f(x)*] can be obtained by the Hilbert transform of its real part. One can measure the real part of Log[*f(x)*] directly, so the Hilbert transform provides the quantitative phase information of the complex optical field.

**Fig. 32:**
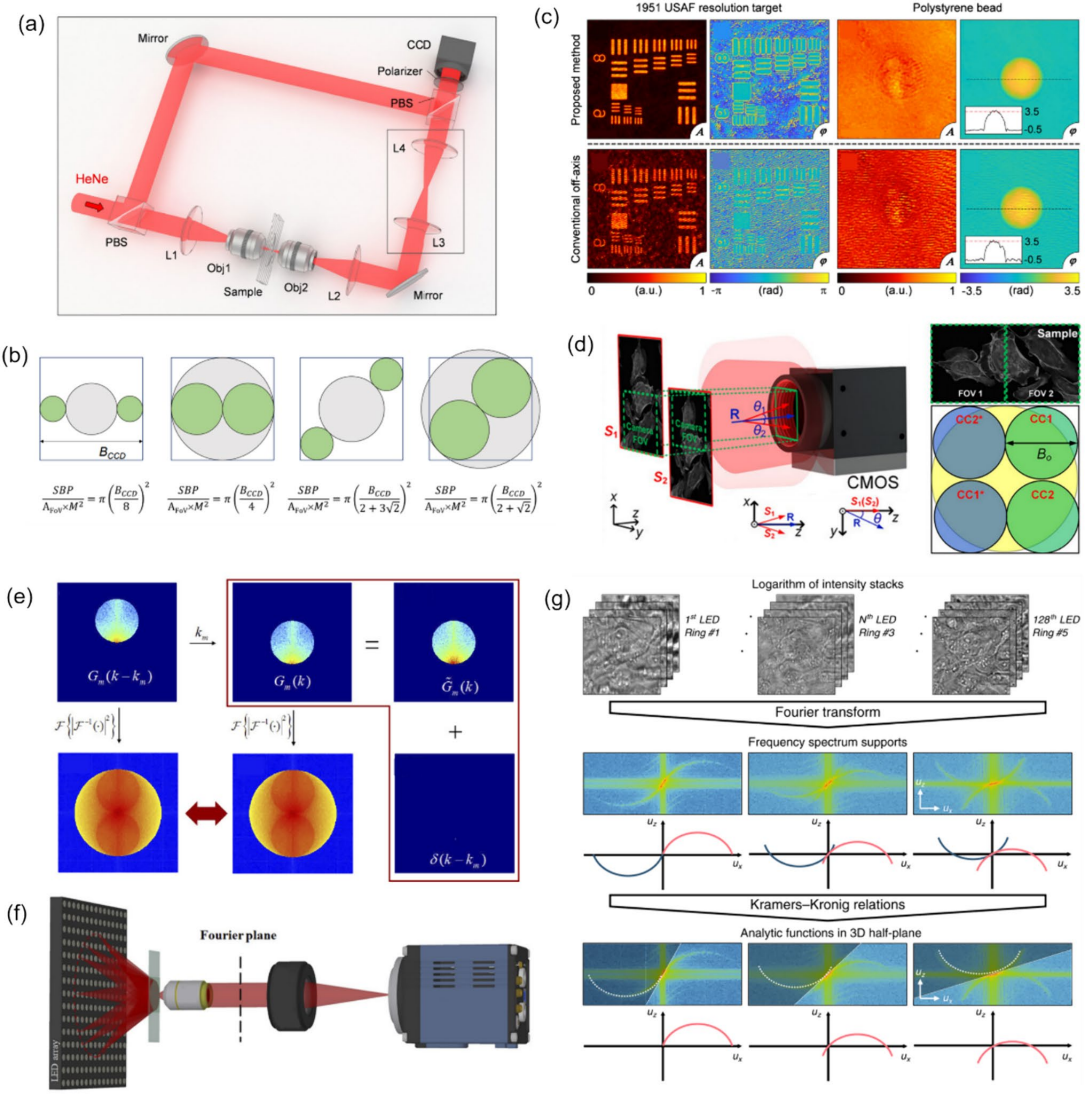
Various approaches in QPI exploiting spatial convolutions. **a** Optical configuration of conventional off-axis holography [224]. *L#* lens; *Obj#* objective lens; *PBS* polarizing beam splitter; *CCD* charge-coupled device. **b** Fourier transforms of interferogram under various configurations. *SBP* space-bandwidth product; *M* magnification. The gray and green circles indicate the auto- and cross-correlation terms, respectively. **c** Demonstration of off-axis holography using the Hilbert transform. The USAF resolution target and polystyrene bead are displayed, showcasing their amplitude and phase images, respectively. **d** Extension of the FOV through spatial multiplexing [233]. *CC* cross-correlation. **e** Principle of non-interferometric QPI using the Hilbert transform [236]. The Fourier transforms of the optical field and its intensity are depicted. **f** Optical setup of non-interferometric QPI leveraging the Hilbert transform. This study employed a light-emitting diode array. **g** Application of the Hilbert transform to three-dimensional space for diffraction tomography [194]. Intensity stacks are captured for each angle of illumination

The application of the Hilbert transform in off-axis holography seeks to enhance the space-bandwidth product (SBP)—a metric defined by the system's spatial resolution and imaging FOV [223, 224]. Figure 32a illustrates the optical setup of conventional off-axis holography, where a slightly tilted reference field interferes with the sample field on the detector plane. While maintaining the same arrangement of optical components, the magnification and numerical aperture (NA) of an objective lens are adjusted to meet the conditions necessary for implementing the Hilbert transform. Specifically, the Fourier transform of the reference field must reside at the boundary of the Fourier spectrum. Additionally, the amplitude of the reference beam should exceed that of the sample beam.

Figure 32b illustrates multiple Fourier spectra associated with these methods. In traditional off-axis holography, it is essential that the cross-correlation term does not overlap with the auto-correlation term in the Fourier plane. However, when employing the Hilbert transform, such overlap is not only permissible but also advantageous for enhancing the SBP (Fig. 32c). To further amplify the SBP, the authors introduce cylindrical lenses, which expand the Fourier spectrum unidirectionally. In [233], a different tactic is employed to extend the FOV. Here, the sample beam is split and directed onto the detector plane from different angles, in conjunction with the use of the Hilbert transform (Fig. 32d). This method is commonly used in off-axis holography [234, 235]. Notably, the achievable SBP remains consistent between the configurations described in [225, 233], affording the optical system the flexibility to adopt either approach.

Hilbert-transform-based QPI offers considerable advantages, particularly for non-interferometric configurations, thereby significantly enhancing the system's usability. In [194, 226, 236], the unscattered light, which corresponds to the DC component in Fourier space. This approach obviates the need for an auxiliary reference arm, thereby expanding the possibilities for field retrieval based purely on intensity distributions. A thorough mathematical framework to underpin this methodology is expounded upon in [237], drawing upon complex analysis. The system complies with holomorphic properties by appropriately constraining the NA. To preclude the formation of complex zeros in the upper half-plane, meticulous control over both the position and amplitude of the unscattered light is exercised. The fundamental principle underlying this approach is depicted in Fig. 32e, where a delta function is shown to act as the Fourier transform of the reference field.

In [226], the spatial frequency of the unscattered light is manipulated by projecting obliquely oriented, spatially coherent light that matches the system's NA. This modulation of the spatial frequency was achieved through the use of galvanometric mirrors in conjunction with a superluminescent light-emitting diode (sLED) with a bandwidth of 5 nm. Utilizing an sLED negates the need for stringent temporal coherence. Alternatively, a liquid crystal spatial light modulator positioned at the Fourier plane could replace this configuration to trim the Fourier spectrum [238]. In [236], the authors employ an LED array to attain a broader bandwidth of 20 nm while simplifying the illumination setup (Fig. 32f). Intriguingly, the principles of the Hilbert transform are versatile enough to be applied to any intensity profile characterized by edge-dominant Fourier spectra. In [194], a three-dimensional Fourier support for each angle of illumination is reconstructed by applying the Hilbert transform to a 3D stack of intensity profiles (Fig. 32g). For the purpose of recovering the cap delineated by the Ewald sphere, techniques like sample rotation and truncation of the half Fourier spectrum are employed to obtain the optical field corresponding to the given frequency support [225, 239]. Note that all these methodologies are predicated on the one-dimensional Hilbert transform.

### Discussion and future perspectives

The utilization of spatial convolutions significantly influences the field of QPI, bringing forth distinct advantages such as the enhancement of the SBP in off-axis holography and enabling non-interferometric modalities with temporally low-coherent light sources. These benefits have been demonstrated across various applications of QPI that employ the Hilbert transform. Moreover, the Hilbert transform can potentially advance other QPI techniques. For instance, its integration with iterative imaging approaches like Fourier ptychography can provide a robust initial guess for field retrieval, thereby improving the overall imaging process.

While the Hilbert transform brings noteworthy advantages to QPI methodologies, it is not without limitations. One such constraint arises from the requirement that the amplitude of either the reference beam or the unscattered term must be strong, thereby limiting the dynamic range of measurements in off-axis holography. This drawback becomes even more pronounced in non-interferometric settings, where the types of samples that can be imaged are restricted based on the contribution from the scattered term [237]. Furthermore, accurate positioning of the DC term at the Fourier spectrum's boundary is essential; otherwise, reconstruction errors may occur. These errors are notably challenging to rectify post-acquisition, unlike in interferometric methods. The application of optical fields acquired under Hilbert-transform-based imaging conditions to diffraction tomography can also introduce image artifacts, primarily due to the constraints of the Rytov approximation [226]. Additionally, the necessity for an illumination modulation unit along with multiple image acquisitions renders the direct application of the Hilbert transform approach somewhat demanding. To mitigate this, some studies have explored the use of multiplexing techniques to reduce acquisition time, including polarization [240] and spectral multiplexing [241, 242].

The broader challenges associated with Hilbert transform-based imaging lie fundamentally in the constraints imposed by the presence of complex zeros in the upper half-plane [232, 237, 243–245]. The reconstruction of analytical optical fields hinges not only on these complex zeros but also on the application of the Hilbert transform itself. The analytical properties of these complex optical fields warrant further exploration within the context of QPI. Viewed diachronically, the question of whether Fienup’s hybrid input–output algorithm can accurately reconstruct the complex field from acquired intensities is intricately tied to these same analytical properties [246–248]. Factors such as the shape of the Fourier spectrum and the image support have direct implications for the feasibility of field retrieval [249]. In future studies, we intend to delve deeper into the holomorphic characteristics of complex optical fields.

## Affine transform-based twin-image suppression for in-line lensless digital holographic microscopy (Marcin J. Marzejon, Mikołaj Rogalski, Maciej Trusiak)

### Background

Conventional microscopy techniques often face challenges when imaging transparent objects, as these objects lack sufficient contrast for clear visualization. To address this limitation, a group of techniques known as QPI has emerged [181], allowing for the capture of phase information that represents optical path differences within the sample. Thus, QPI enables high-contrast imaging of transparent samples. Among QPI techniques, the in-line Lensless Digital Holographic Microscopy (LDHM) stands out for its large field-of-view, simplicity, and cost-effectiveness, as it does not rely on bulky and complex setups, making it a promising tool for transparent object high-throughput imaging.

**Fig. 33:**
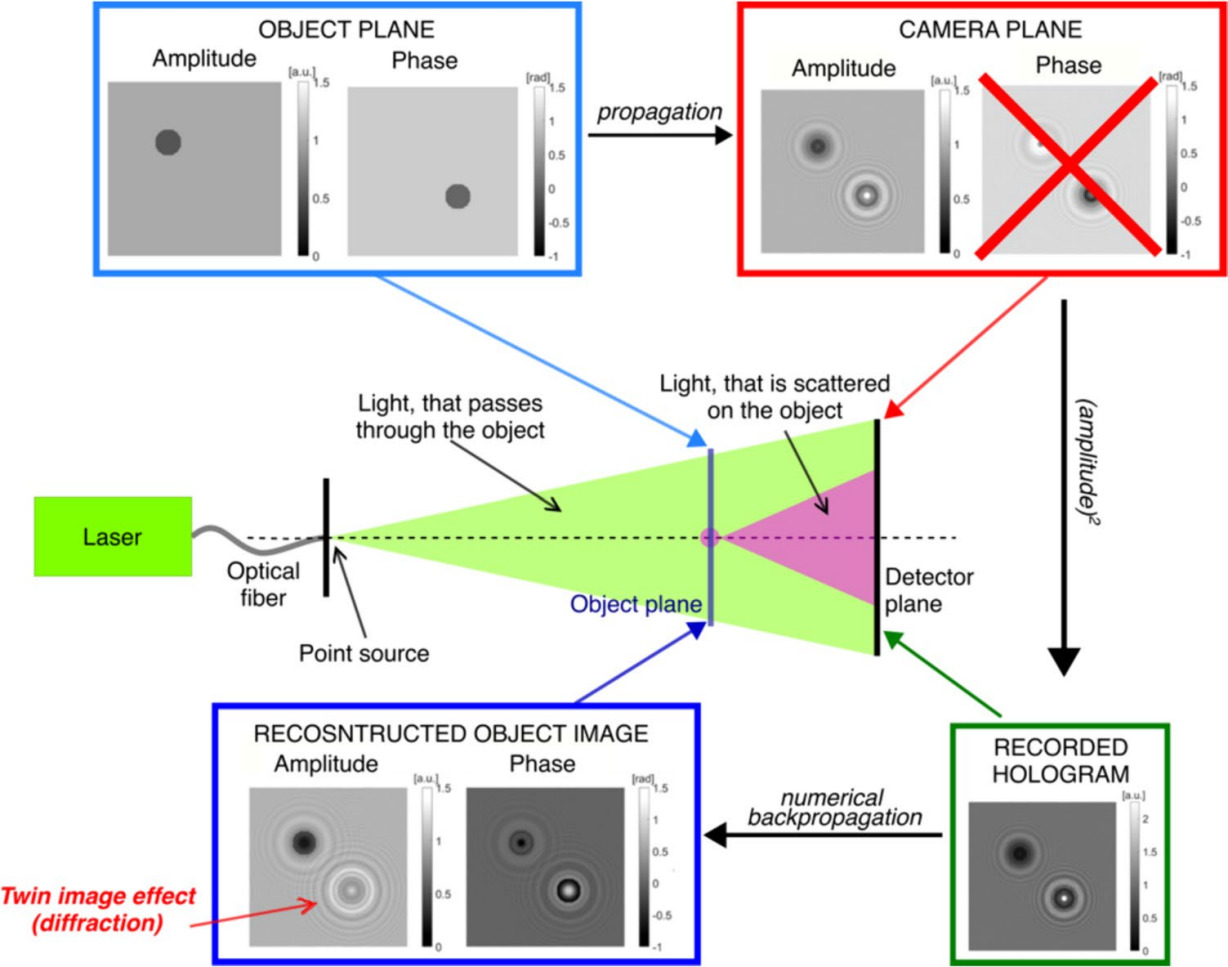
Working principle of in-line lensless digital holographic microscopy (LDHM). The schematic diagram presents the simulated optical field at the object and camera planes for pure amplitude (upper left corner) and a pure phase object (lower right corner)

The in-line LDHM is an imaging technique based on the concept of common-path holography introduced by D. Gabor in 1948 [250]. The simplest possible optical setup of the system consists of a (point) light source and a sensor, and the imaging object is placed somewhere between those two elements, as presented in Fig. 33. The illuminating wavefront passes through the sample, and part of it is diffracted on the sample, with the majority of the light passing in a ballistic mode. The diffracted and non-diffracted wavefronts interfere at the sensor plane, forming an interference pattern—a Gabor hologram. The sample is numerically reconstructed via repropagation (refocusing) from the hologram to the object plane, using usually the angular spectrum (AS) algorithm [251].

In-line LDHM eliminates the need for traditional bulky optical imaging systems. Advantages of this technique include, among others, the ability to image a large sample area label-free and in high resolution, simple construction, and generally low cost of the system [252]. Additionally, hardware and algorithmic modifications may allow for obtaining sub-pixel resolution [253]. The straightforward architecture and small number of elements in the system enable the setup to be scalable and the dimensions of the device to be adjusted depending on the requirements of the working environment. Another advantage of LDHM is the ability to clearly image biosamples (e.g., single living cells, tissue sections, thin-structured samples, diluted biological samples, etc.), that meet the Gabor holographic conditions, without the use of exogenous contrast (e.g. fluorescent staining) [252]. LDHM has found many applications—biological sample imaging [252, 254–262] medical diagnostics [17, 263–265], bio-objects 4D tracking [266], metrology and quality control [267], among others [19, 268].

The main challenges in the in-line Gabor LDHM are the coherent noise (spurious interference patterns caused by back reflections, inhomogeneities, speckles, coherent artefacts, etc.) and the twin-image problem (see the reconstructed hologram in Fig. 33). The above-mentioned factors are responsible for the introduction of phase distortion that propagates during the reconstruction of the tested object. The twin-image problem results from the fact that the intensity of the optical field is recorded on the detector, which includes the field coming from the object (1st order) and its coupled field (–1st order), and an incoherent autocorrelation term (0 order) [253], which overlap in a common-path configuration. For biological samples, there may be a challenge of low photon budget imaging as the exposure of cells and tissues should be kept low due to phototoxicity. However, our group showed, that it is possible to operate in the low photon budget regime down to the illumination power of 7 μW and still get good quality images in terms of the contrast and the hologram phase and amplitude reconstruction resolution [269].

So far, several solutions have been proposed to overcome the limitations of the in-line LDHM. The coherence noise may be effectively reduced, eg., by using a rotating diffuser [267] or a partially coherent illumination [257, 271]. The first method, proposed by our group, enables the reduction of the amplitude and phase noise for a technical test target imaging by 51% and 35%, respectively. The tests on biological samples revealed a reduction of the speckle noise by 33% [270]. Twin images can be removed by hardware and algorithmic alterations in the optical setup. e.g., by recording holograms for multiple wavelengths [252, 272–274], for at least two different sample-camera distances (axial shift, multi-height approach) [253, 255, 275, 276], by using the Talbot grating illumination [277], or deep learning algorithms [278]. Details of the selected strategies for the twin-image removal will be discussed in the following parts of this manuscript.

We propose here an affine transform approach to align holograms in multi-height phase retrieval for twin-image suppression in LDHM.

### Twin-image removal strategies for in-line lensless digital holographic microscopy

**Fig. 34:**
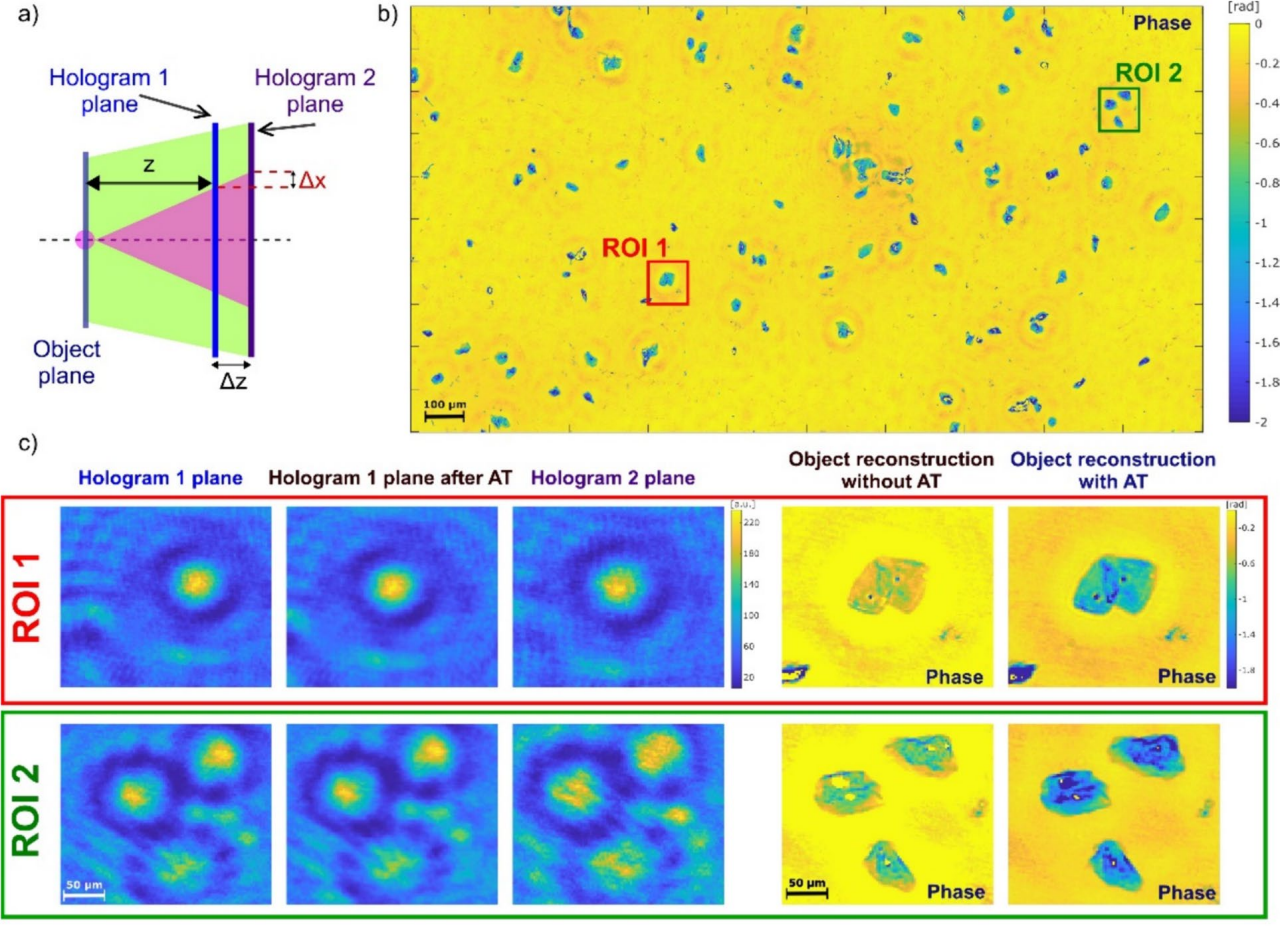
Twin-image removal in the in-line lensless digital holography microscopy by multi-height hologram acquisition. **a** The idea of the method. Δz denotes the hologram (sensor) axial (longitudinal) plane shift and Δx denotes the in-plane (transversal) shift of the hologram features positions for the two hologram planes. **b** The phase image of the human cheek cell sample (after reconstruction). **c** Zoom for ROI 1 and ROI 2 with indicated holograms from various planes, corrected hologram in plane 1 (AT stands from Affine Transform), and the reconstructed object phase. Note, that after correction, the diffraction patterns in holograms 1 and 2 are localized in the same XY position

The hologram recorded by a sensor is an intensity-only projection of the complex field containing information about both the amplitude and phase of the object. The phase may be retrieved by using the iterative GSA [200], having at least two input images—intensity (real) defocused projections of the complex field linked via the Fourier transform (in the case of in-line LDHM—two holograms). One of the solutions presented by Greenbaum and Ozcan is to acquire two holograms at two different sensor planes see (Fig. 34a) [253]. Then, the pair of acquired holograms may be used as an input for the GSA, and the full information of the object at both acquisition planes is retrieved. From our experience, 5 to 25 iterations of the GSA enables one to retrieve the complex wavefront (amplitude and phase) with good quality. Then, knowing the distance between the hologram acquisition plane and the object plane, the complex hologram may be backpropagated into the object plane with minimized twin-image errors. The solution described in [253] was adapted later by Mico et al. [255] for in-line digital holographic microscopy with lenses.

The multi-height approach [253, 255] is a very convenient algorithm, easy to implement in the laboratory system using an automated translational stage. The only disadvantage of this method is that for different sensor positions, the corresponding parts of the holograms are placed at the different camera pixels [defocus-like effect; denoted as Δx in Fig. 34a]. Therefore, when employing the AS method to backpropagate both holograms, the corresponding object details will be shifted by Δx in both reconstructions (varying with the defocus distance), making it impossible to straightforwardly apply the GSA. To avoid this problem, we propose here the hologram pre-processing method as follows. First, each of the holograms [Fig. 34c; *Hologram 1 and Hologram 2 plane*] is backpropagated to the object plane using the AS algorithm. Next, the features of the reconstructions are detected using the Speeded-Up Robust Features (SURF) algorithm [279] (MATLAB implementation – *detectSURFFeatures*). In the following steps, those features are extracted (*extractFeatures*), and the corresponding features in both reconstructions are matched (*matchFeatures*). Based on the shift between the feature pairs, the affine transformation of one of the holograms is retrieved (*estgeotform2d*). The hologram pre-processing part is finished by applying the retrieved affine transform to the second hologram and correcting its translation and magnification [see Fig. 34c; *Hologram 1 plane after AT*]. The resulted holograms are the input data for the GSA. After the phase retrieval, the image of the object is obtained by the backpropagation of complex hologram to the object plane [AS algorithm; Fig. 34b and c].

Another twin-image removal strategy was presented by Mico et al. [255]. In this paper, the authors used a simultaneous multi-wavelength illumination (450 nm, 532 nm, and 635 nm) and RGB camera sensor. The data from a single shot were spectrally resolved using the blue, green, and red channels of the acquired image, resulting in three holograms (B: 450 nm, G: 532 nm, R: 635 nm) from a single frame. Due to the different wavelengths, the phase shift between holograms in the B, G and R channels appears to enable iterative phase retrieval. It is worth noting that the multi-wavelength illumination approach is somewhat analogous to the multi-height approach in terms of data multiplexing (redundancy). In both strategies, one records multiple (at least two) phase-shifted holograms—either by the geometrical path difference Δz (multi-height approach) or by the change in the illumination wavelength. The authors of [4] proposed a modified version of the GSA that enables phase retrieval from three recorded holograms, linked to the multi-height algorithm presented in this paper via additional complex field filtering employed before each propagation. Complex field filtering helps to decrease the noise level and avoid numerical reconstruction errors of the sample’s RI by assuming the minimum (maximum) RI value in the sample. The proposed strategy can be modified in terms of the number of illumination wavelengths—the required minimum is two. The multi-wavelength approach can also be implemented with a monochrome sensor or for wavelengths that are not spectrally separable with an RGB camera by consecutive acquisition of holograms for each wavelength separately.

### Summary and future perspectives

Recent achievements in the in-line LDHM have marked significant progress in the resolution and sensitivity of the technique, enabling the visualization of finer cellular and subcellular structures with remarkable clarity. Innovations in computational algorithms and hardware have also accelerated image reconstruction and processing, making the technology more efficient. Looking ahead, the prospects for in-line LDHM are promising. The technology is poised to further revolutionize biomedicine by facilitating the rapid diagnosis of diseases, monitoring cellular responses to therapies, and contributing to the development of personalized medicine. Its application in point-of-care devices could enable cost-effective and portable diagnostic tools, particularly in resource-limited settings. Low photon budget imaging capabilities give hope to shift toward exotic radiation regimes. As the field continues to evolve, the integration of artificial intelligence and machine learning algorithms could enhance the automation of data analysis and interpretation, making this microscopy technique an invaluable asset for both research and healthcare applications. The MATLAB codes for Affine transform-based twin-image suppression for in-line Lensless Digital Holographic Microscopy are given in supplementary materials S11.

## High throughput low coherence quantitative phase microscopy (Paweł Gocłowski, Azeem Ahmad, Vishesh Dubey, Maciej Trusiak, Balpreet S. Ahluwalia)

### Background

Quantitative phase imaging (QPI) is a label-free and non-invasive method that utilizes the intrinsic spatial refractive index variation of the specimen to generate high-contrast and quantitative image. Quantitative phase microscopy (QPM) has found various applications in bio-medical research [280]. Contrary to fluorescence microscopy, QPM brings strong benefits, whereas fluorescence labelling is not allowed because it alters the natural states of the delicate biological specimens such as sperm cells. In addition, fluorescence microscopy suffers from photo-bleaching and introduce photo-toxicity to the specimens due to the use of high laser powers. QPM allows to extract quantitative parameters of the specimens such as refractive index, cell dry mass, surface area, volume and others [281, 282].

**Fig. 35:**
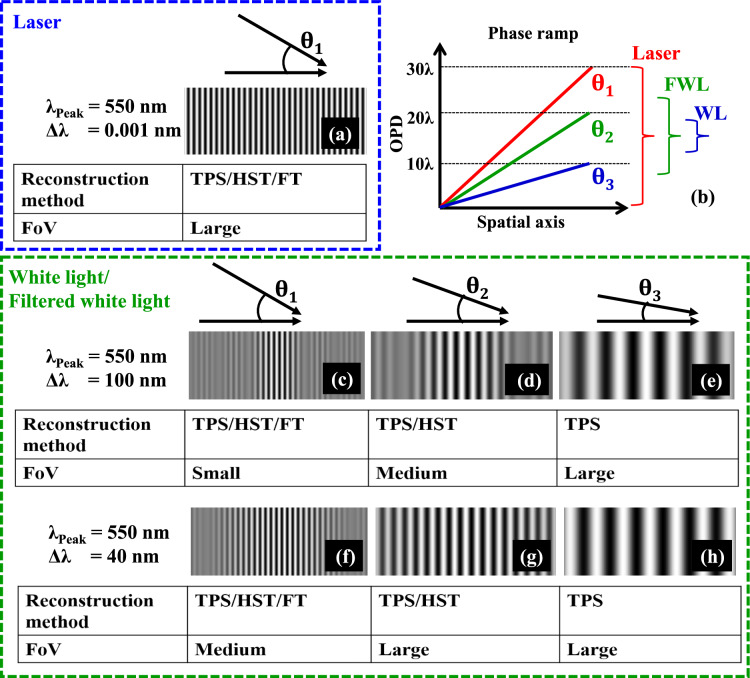
Conceptual diagram comparing low and high coherence of the light source. Basic interference is shown between two plane waves with an angle between them for a laser, *WL*—white light and *FWL*—filtered white light (**b**). Laser illumination allows to generate high density interference fringes over entire FoV (**a**). (**c**-**h**) Variation of fringe width with angles. With incoherent illumination it is only possible to generate sparse fringes over large FoV (**e**, **h**) or dense fringes over small FoV (**c**, **f**)

Traditionally, highly coherent light sources such as lasers are used in QPM systems to easily obtain the interference fringes. Unfortunately, the high coherence of the light source leads to the generation of speckle noise and coherent noise severely reducing the spatial phase sensitivity of the QPM system. These problems led to the growing popularity of low coherence QPM (LC-QPM), where incoherent light sources like light emitting diodes (LEDs) or halogen lamps are utilized [283–285]. Low coherence, however, brings strict optical path difference (OPD) requirements to the optical system. The concept figure is shown in Fig. 35. The OPD between the object and the reference arms of the interferometer must be smaller than the temporal coherence of the light source (which is only ~ 10 μm for LEDs and ~ 2 μm for halogen lamp) to form high contrast fringes. LC-QPM does not generate high density fringes over the large field of view (FoV) corresponding to large OPD (red line in Fig. 35) due to the short temporal coherence length. Therefore, a tradeoff must be made regarding the phase reconstruction when using LC-QPM. Temporal Phase Shifting (TPS) [286] can recover the accurate phase map of the sample from any type of interferogram, but temporal resolution is partially sacrificed because of the multi-frame requirement. Single-shot methods such as Fourier Transform (FT) [287] can perform phase reconstruction using only single interferogram, but it works properly only for high-density fringes for lossless phase reconstruction. The use of FT method in LC-QPM albeit gives high temporal resolution, it comes with a compromised FoV and reduced spatial resolution. In this work, we demonstrate that the use of Hilbert Spiral Transform (HST) [288] based phase recovery algorithm is an attractive route for LC-QPM which works with low-density, curved and circular interference fringes, supporting large and scalable FoV, enhanced spatial phase sensitivity and temporal resolution limited by the camera speed owing to its single-shot approach.

### Methodology

**Fig. 36:**
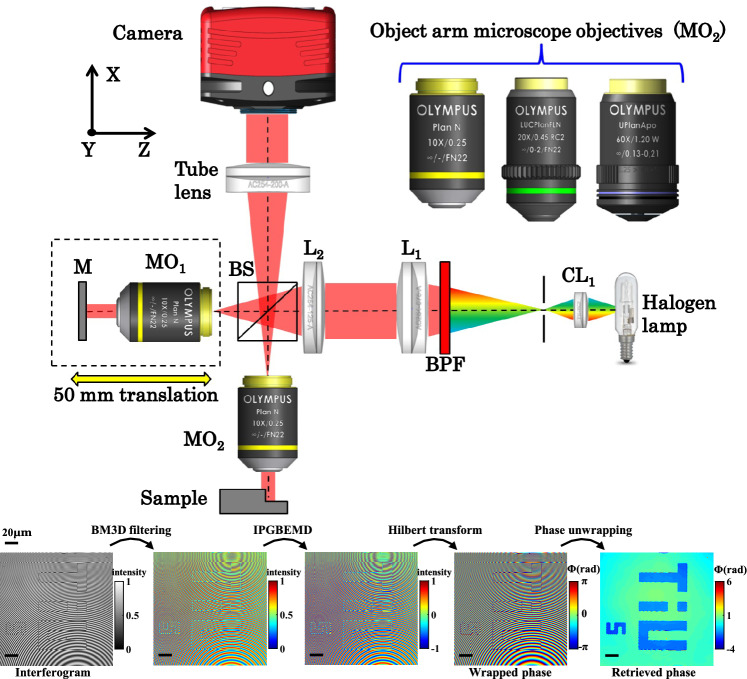
Top**—**schematic drawing of the experimental setup. *MO*_*1–2*_ microscope objectives; *BS*_*2*_ beam splitter; *L*_*1–2*_ achromatic doublet lenses; *CL*_*1*_ coupling lens; *BPF* bandpass filter; *M* mirror. Bottom—phase reconstruction procedure: noise filtration by BM3D, background removal by iPGBEMD, phase retrieval by HST and phase unwrapping by Miguel 2D algorithm

The schematic of our hybrid experimental–numerical approach is presented in Fig. 36. The optical setup is based on Linnik interferometer configuration with objective lenses in both the object and the reference arms. Halogen lamp is utilized as the light source. White light is subsequently filtered with bandpass filter (632 nm peak wavelength and 10 nm spectral bandwidth), collimated with lens L1, split into 2 by a beam splitter BS and focused with lens L2 at the back focal planes of the objective lenses. The object and the reference beams are reflected at the sample and the reference mirror respectively, get recombined at BS2 and finally form the interferogram at the camera.

The bottom part of the Fig. 36 shows our post-processing path to fully reconstruct phase map of the sample from the raw interferograms. Pre-processing of the fringe pattern is necessary to properly perform HST. Firstly, noise is removed by Block Matching 3D algorithm (BM3D) [289]. Image is subsequently filtered by an improved Period-Guided Bidimensional Empirical Mode Decomposition (iPGBEMD) algorithm [290], which detaches the fringe component (oscillating around 0 mean value) from the image background. The wrapped phase is then retrieved from pre-processed interferogram by HST and unwrapped with Miguel 2D algorithm [291]. Total computation time of the whole phase reconstruction process is around 5 min for medium-advanced personal computer.

### Results

**Fig. 37:**
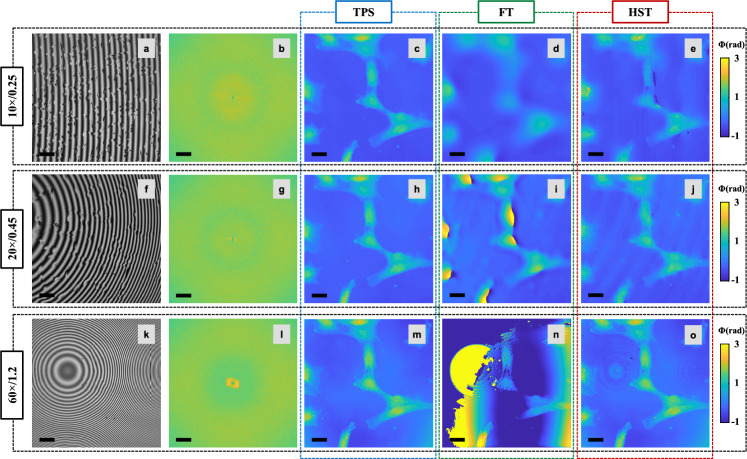
Experimental results of MEF cells for 10 × /0.25, 20 × /0.45 and 60 × /1.2 objective lenses: raw interferograms (**a**, **f**, **k**), FT spectra (**b**, **g**, **l**), phase reconstructed with TPS (**c**, **h**, **m**), phase reconstructed with FT (**d**, **i**, **n**) and phase reconstructed with HST (**e**, **j**, **o**). The imaging objective lens MO_2_ as shown in Fig. 35 is changed while MO_1_ was kept constant 10 × /0.25 N.A

To prove utility of this approach, we have acquired experimental interferograms of Mouse Embryonic Fibroblasts (MEFs) and reconstructed the phase maps with three algorithms: HST, FT, and TPS (as the ground truth). The results are presented in Fig. 37.

Three objective lenses are used in the object arm (10 × /0.25, 20 × /0.45 and 60 × /1.2, i.e., MO_2_ in Fig. 35) while 10 × /0.25 was kept in the reference arm. For 20 × and 60 × , fringes are no longer straight and become either curved or circular, because OPD adjustment required to observe interference in low coherent light generates wavefront curvature mismatch between the object and the reference arms.

The results demonstrate that HST is a versatile tool capable of phase reconstruction for wide range of fringe curvatures and densities. Both FT and HST are a single-shot method enabling high-temporal resolution, but FT is limited to dense and linear interference fringes that are difficult to generate in the LC-QPM configuration especially for non-identical objective lenses in the object and reference arm. For linear and curved fringes (Fig. 37d, i), FT reconstructed phase maps have poor spatial resolution because of the small separation between Fourier peaks in the frequency domain. For closed fringes, FT reconstruction generates significant reconstruction artefacts (Fig. 37n). Contrary, HST reconstruction (Fig. 37e, j, o) is more robust towards curved and circular fringes and provides high temporal resolution (limited only by camera speed) than TPS at the cost of slightly worse spatial resolution.

### Conclusions and future perspectives

The combination of LC-QPM system with single-frame HST phase reconstruction allows to increase the throughput of the measurement by achieving very high temporal resolution limited only by acquisition speed of the camera without sacrificing spatial resolution. Additionally, HST is more robust towards various shapes of fringe patterns, i.e., curved and circular, that open possibilities of working with unbalanced interferometry set-up and thus supporting scalable FoV. This approach can benefit bio-imaging of highly dynamic specimens where both high-spatial sensitivity and high imaging speed are necessary.

## Pixel super-resolution phase retrieval for high-resolution lensless holographic microscopy (Yunhui Gao and Liangcai Cao)

### Background

**Fig. 38:**
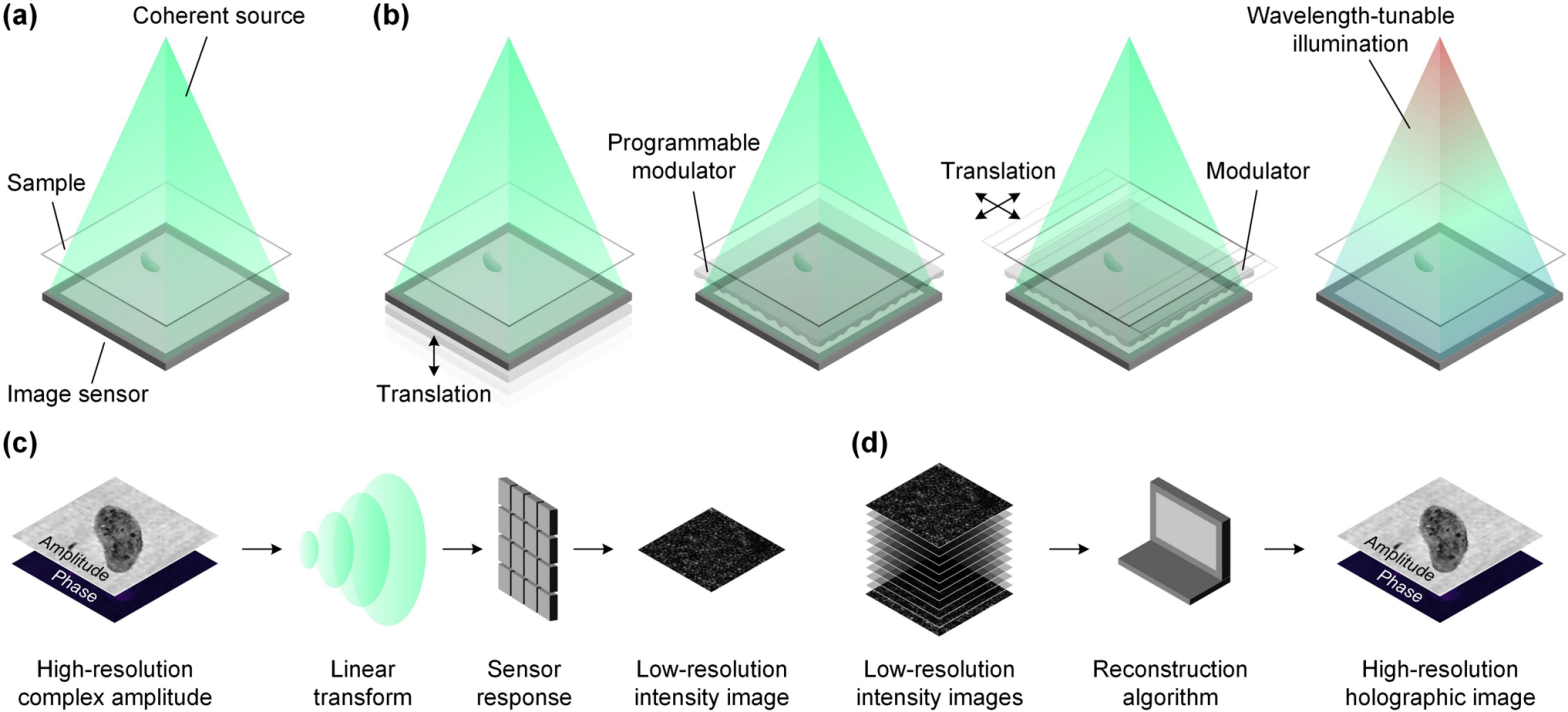
Schematic illustration of lensless holographic microscopy. **a** General experimental setup. **b** Typical diversity measurement schemes, where multiple intensity images are recorded with varying imaging distances, modulation patterns, translation positions, and illumination wavelengths, etc. **c** The forward model of the imaging process. **d** The captured low-resolution intensity images are numerically post-processed to retrieve the high-resolution holographic image

The principle of lens optics lies at the foundation of many of today’s imaging technologies. Recently, however, lensless imaging has emerged as an alternative yet competitive imaging modality at the microscopic scale [16]. Contrary to the conventional point-to-point imaging framework, in lensless microscopy, a diffraction pattern is directly recorded on an image sensor, as shown in Fig. 38a. By leveraging advancements in computational imaging theories and image processing algorithms, lensless microscopy provides potential solutions to address the intrinsic limitations associated with traditional lens-based imaging methodologies. First, a large field-of-view comparable to the sensor area and a diffraction-limited spatial resolution can be achieved simultaneously, bypassing the limited space-bandwidth product of lens optics. Second, based on a coherent imaging model, lensless imaging enables holographic reconstruction, i.e., retrieving both the absorption and the phase information of the sample. Additionally, compared to lens-based benchtop devices, lensless microscopy facilitates a highly compact, light-weight and cost-effective setup, thereby enabling low-cost and portable operations in resource-limited areas. Lensless imaging has been successfully demonstrated in high-throughput pathology [292], cytometry [293], surface metrology [294], and polarimetry [295].

Despite their distinct advantages, lensless microscopy introduces new technical challenges that need to be addressed. Due to the sensor’s intensity-only response and large pixel size, phase and subpixel information cannot be directly resolved from raw measurements. To achieve high-resolution holographic imaging, computational imaging approaches have been incorporated into the context of lensless microscopy, which are referred to as pixel super-resolution phase retrieval techniques. In this chapter, we present a brief overview of the pixel super-resolution phase retrieval techniques for high-resolution lensless holographic microscopy.

### Methodology

From the perspective of computational imaging, pixel super-resolution phase retrieval involves two key steps: the physical encoding of a high-resolution holographic image into low-resolution intensity-only measurements, and the numerical decoding of information from the raw data.

**Fig. 39:**
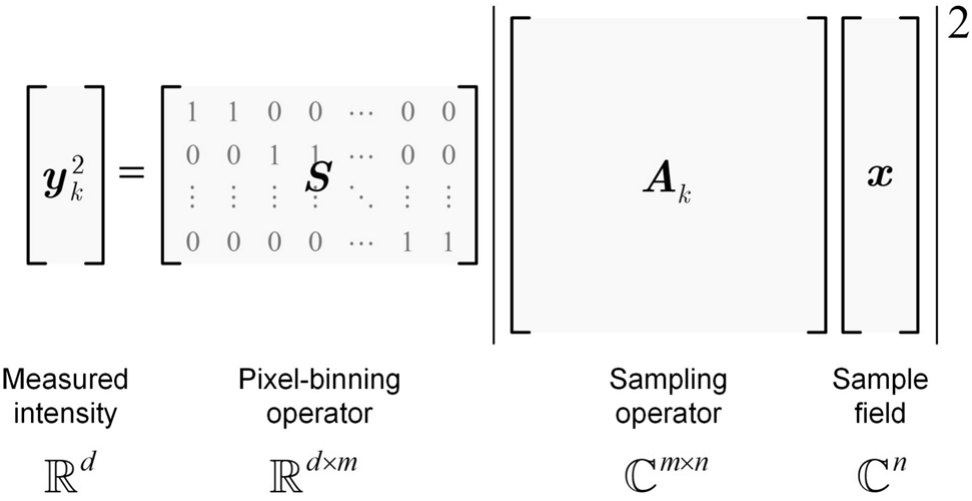
Conceptual illustration of the forward model defined by Eq. (15)

The encoding step entails the design of optical systems and sampling schemes that can translate the sample’s complex field and subpixel information into measurable intensity images. It has been found that any complex sampling operator, including the free-space propagation of light, can potentially serve as an information encoding candidate [296, 297]. Given the ill-posed nature of the image reconstruction problem, measurements are typically performed by recording multiple diffraction patterns with varying physical parameters, a process known as the diversity measurement scheme. Such diversity can be achieved by varying parameters such as the sample-to-sensor distances [253, 298], wavefront modulation patterns [202, 299], lateral translation positions [300], and illumination wavelengths [204, 301, 302], as schematically depicted in Fig. 38b. The general forward model can be expressed as

15 $${{\varvec{y}}}_{k}^{2}={\varvec{S}}{\left|{{\varvec{A}}}_{k}{\varvec{x}}\right|}^{2},\boldsymbol{ }\boldsymbol{ }\boldsymbol{ }\boldsymbol{ }k=\text{1,2},\dots ,K,$$

where ${\varvec{x}}\upepsilon {\mathbb{C}}^{n}$ denotes the high-resolution holographic image of the sample, ${{\varvec{A}}}_{k}\in {\mathbb{C}}^{m\times n}$ denotes the sampling operator with respect to the *k*th out of *K* diversity measurements, ${\varvec{S}}\in {\mathbb{R}}^{d\times m}$ (with $m=\sigma d$ and $\sigma$ being a positive integer) denotes the pixel binning operator of the sensor pixels, and ${{\varvec{y}}}_{k}^{2}\in {\mathbb{R}}^{d}$ denotes the recorded intensity image corresponding to the *k*th measurement. The physical and mathematical models of lensless microscopy are shown in Figs. 38c and 39, respectively. As the number of measurements, increases the problem becomes well-posed, indicating that the recovery of the high-resolution holographic image is indeed physically feasible.

Given a dataset of low-resolution intensity images, the second step involves numerically decoding the high-resolution holographic image through computation. Although the reconstruction algorithms can vary based on the specific experimental settings and applications, a general approach is based on the following inverse problem:

16 $$\widehat{{\varvec{x}}}=\underset{{\varvec{x}}}{\text{argmin}}\frac{1}{2K}\sum_{k=1}^{K}{\Vert \sqrt{{\varvec{S}}{\left|{{\varvec{A}}}_{k}{\varvec{x}}\right|}^{2}}-{{\varvec{y}}}_{k}\Vert }_{2}^{2}+R\left({\varvec{x}}\right)$$

where the high-resolution holographic image is obtained by minimizing an objective function. The first term in the objective function ensures that the estimated solution is consistent with the forward model of Eq. (15). Considering the ill-posedness of the problem, especially under conditions of limited measurements, the introduction of an additional regularization function *R(x)* becomes necessary. The regularization function can incorporate prior knowledge such as sparsity [303] or implicit features [304, 305]. Formulating pixel super-resolution phase retrieval as a standard optimization problem of Eq. (16) enables the use of standard numerical optimization tools such as gradient descent or proximal gradient algorithms. To support further application, a MATLAB implementation of the pixel super-resolution phase retrieval algorithm is available at Ref. [306].

### Results

**Fig. 40:**
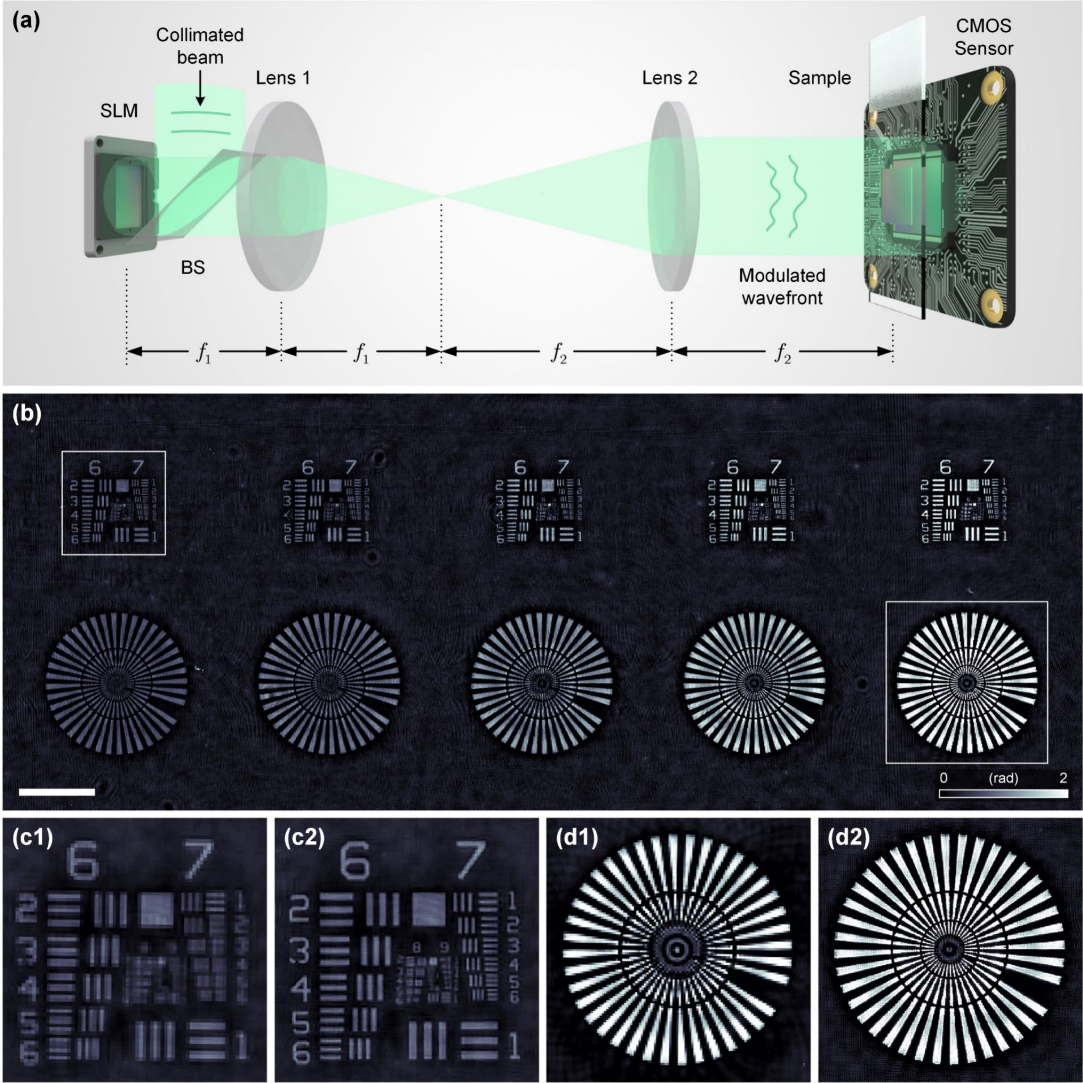
Experimental realization of high-resolution lensless holographic microscopy. **a** Experimental setup based on modulation diversity. **b** Retrieved phase profile of a quantitative phase target. The scale bar is 200 μm. (**c1**)/(**c2**) and (**d1**)/(**d2**) show the enlarged areas of **b** without/with pixel super-resolution, respectively. Figure **a** is adapted from [303]

As a proof of concept, high-resolution lensless holographic microscopy was experimentally validated in Ref. [299] utilizing phase modulation diversity with a spatial light modulator (SLM). A collimated and polarized coherent beam from a 532 nm laser is modulated by a reflective phase-only SLM (GAEA-2, HOLOEYE), and illuminates the sample at the conjugate plane of a 4f system (focal lengths ${f}_{1}={f}_{2}=100 \text{ mm}$). A CMOS image sensor (QHY163M, pixel pitch 3.8 µm) is positioned approximately 5.4 mm away from the sample, forming a lensless setup. The experimental setup is schematically shown in Fig. 40a. During data acquisition, a total of *K* = 64 of pre-designed modulation patterns are sequentially uploaded to the SLM, and the corresponding holograms are synchronously recorded by the image sensor. The SLM has been calibrated in advance using a self-referenced method, and the modulation patterns are designed with smooth random profiles so as to offer modulation diversity while minimizing the crosstalk effect [307, 308]. The captured raw holograms are subsequently used for numerical reconstruction of the pixel super-resolved complex sample field according to Eq. (16). Figure 40b displays the experimentally reconstructed phase profile of a quantitative phase target (QPT, Benchmark Technologies), with phase values consistent with the ground truth data. Figure 40c1 and d1 present the enlarged phase images without using pixel super-resolution (*σ* = 1), where the spatial resolution is limited by the sensor pixel size. In contrast, with the help of pixel super-resolution technique, one can overcome the sampling limit imposed by the sensor pixels, achieving a diffraction-limited spatial resolution, as illustrated in Fig. 40c2 and d2.

### Conclusion and future perspectives

Pixel super-resolution phase retrieval is a computational imaging technique that combines encoding optics and decoding algorithms to realize high-resolution holographic microscopy. At current stage, one primary technical limitation is the considerable time consumption during both measurement and reconstruction due to the large data volume. Opportunities for optimization in terms of both optical designs and numerical algorithms are yet to be fully explored, offering potential avenues for enhancing the performance of lensless microscopy [309–311]. Furthermore, the interpretation of high-resolution holographic images for practical clinical and biomedical applications continues to present challenges, which could be potentially addressed with the advancements in artificial intelligence [312].

## A Regularized auto-encoder for the reconstruction of phase and amplitude in digital in-line holography (R.V. Vinu, G. Gopakumar, Ziyang Chen, and Jixiong Pu)

### Background

Holography has been a substantial optical imaging modality for several decades with the exciting three-dimensional complex-valued image reconstruction potential from recorded two-dimensional intensity distribution of the diffracted wavefront [313]. The advancements in modern high-resolution sensors and sophisticated computational techniques made the transition of conventional analog holography to the digital holography (DH) with productive range of applications in various optical imaging scenarios such as label-free biological imaging, life sciences, biomedicine, etc. [258, 314–316]. The DH systems utilize the off-axis or in-line schemes for the generation of holograms and utilize computational techniques for the faithful reconstruction of the amplitude and phase information of the object. Accurate phase recovery is pivotal in the holographic imaging framework, but it remains a challenge in most of the advanced imaging applications. All these years witnessed the introduction and effective implementation of several techniques based on Fresnel-Kirchoff integral, non-paraxial transfer function, compressive sensing, etc. for the reconstruction of phase information of the object [317–320]. Many of these methods are computationally complex and time consuming or require additional frequency domain filtering or phase-shifting mechanisms. On the other hand, there is a recent emergence of machine learning approaches in various phase recovery scenarios and the introduction of deep learning approaches in holography such as phase recovery in holography using deep learning in neural networks [321], deep learning in coherent imaging systems [322], end-to-end deep learning for DH [323], deep digital in-line holography [324], deep learning-based polarization holographic microscope [325], etc. In this chapter, we present a machine learning assisted holographic image reconstruction technique with a regularized auto-encoder for the phase and amplitude reconstruction in a digital in-line holography (DIH) scheme.

### Methodology

**Fig. 41:**
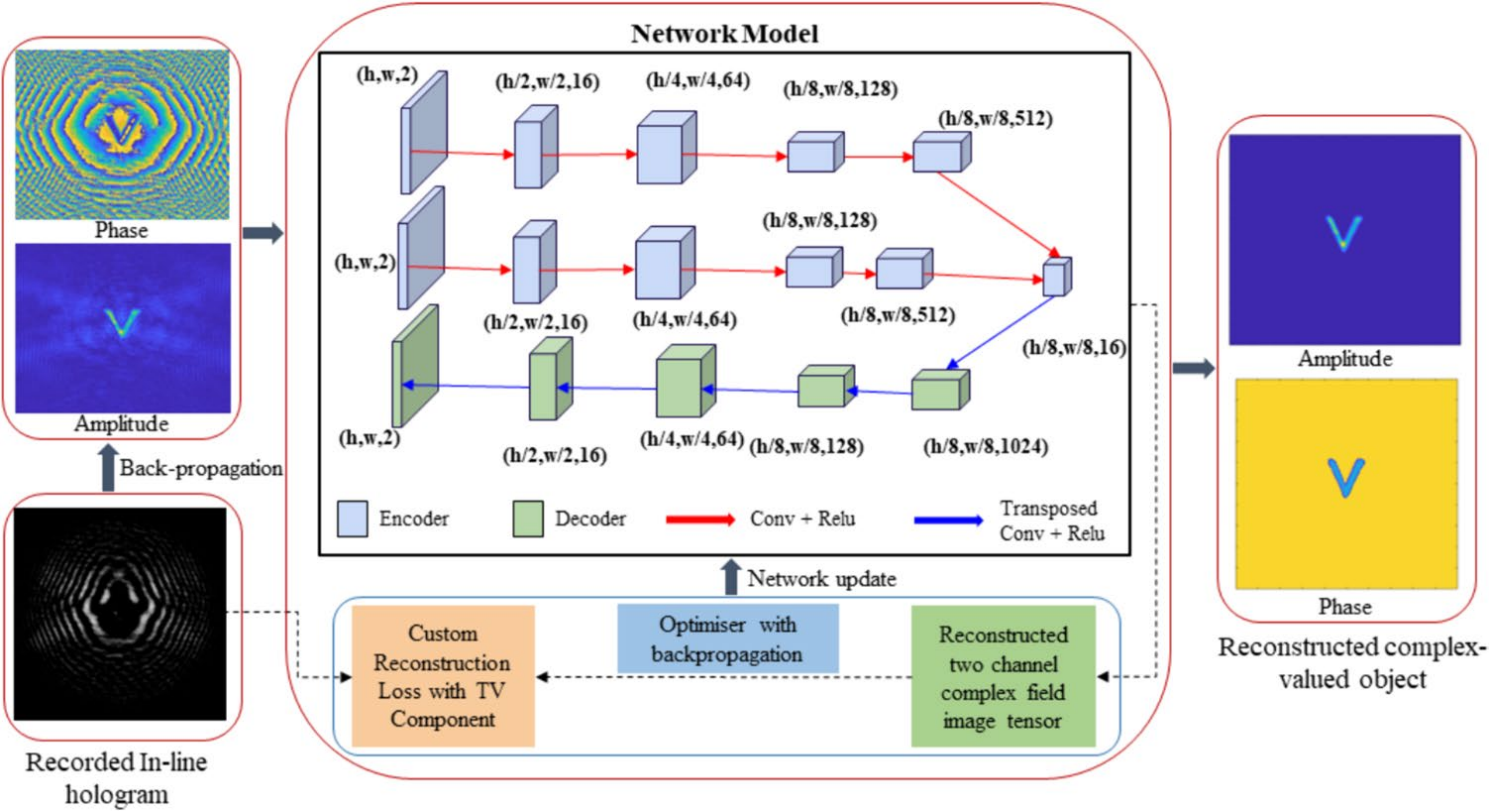
Schematic of deep learning architecture built around the dual autoencoder generative network model

The deep learning architecture implemented for the single-shot digital in-line holographic reconstruction of the phase and amplitude information of the object is shown in Fig. 41. In the architecture, the reconstruction of amplitude and phase information of a known complex-valued object ‘V’ from the recorded in-line hologram is demonstrated. A spatial light modulator (SLM) is utilized to encode the object with a unit amplitude distribution and a uniform phase value. The intensity distribution of the in-line hologram is recorded at a specific distance using a camera, and the respective in-line hologram is fed to the network as described in Fig. 41. The recorded hologram suffers from the twin image problem [250, 326], where the real and virtual images of the object overlap with each other along with the zeroth order term. A novel learning architecture using regularized autoencoder is implemented for the twin image removal and the accurate reconstruction of phase and amplitude information of the object. The recorded in-line hologram is initially processed to obtain the complex-field image representation by employing the back propagation technique with the use of the propagation transfer function, $P = \exp \left[ {ikz\sqrt {1 - \left( {\lambda f_{x} } \right)^{2} - \left( {\lambda f_{y} } \right)^{2} } } \right]$, where ${f}_{x}$ and ${f}_{y}$ are the spatial frequencies, $\lambda$the wavelength, z the propagation distance, and $k=\frac{2\pi }{\lambda }$ the wave number.. The proposed dual encoder-single decoder-based network is trained such that it minimizes the regularized mean squared error between the input hologram and the hologram reconstructed from the latent representation produced as output of the network. For the latent representation, the encoder first converts the complex field image obtained through initial approximation to lower dimensional encoded representation which is then decoded as close as possible to the input. During this process, the network learns to reconstruct images that are robust to distortions and scattering of light that can occur when light waves travel through a medium with varying refractive index. In addition, the network model minimizes the reconstruction loss by adding a custom total variation (TV) component to the MSE of the loss function. A detailed network architecture and digital reconstruction procedure is given in the Supplementary material S1. Moreover, we have explored and quantitatively compared the reconstruction quality of the regularized network with a ‘contractive network’ and a ‘parallel network’. The contractive network consists of a contractive component for regularizing the MSE loss instead of TV component and a parallel network consisting of dual encoder and dual decoder that separately operating on the phase and amplitude approximations.

### Results

**Fig. 42:**
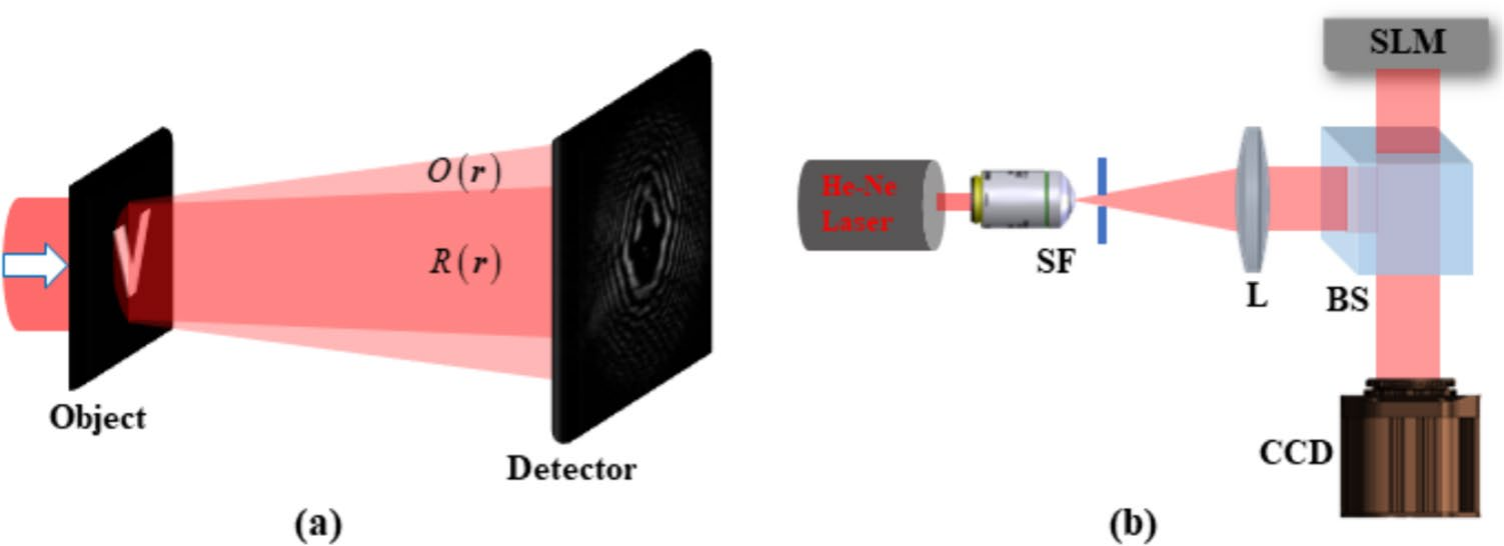
**a** Conceptual schematic of the in-line hologram generation and detection; **b** experimental schematic of the recording of an in-line hologram of a reflecting type of object; *O(r)* object scattered field, *R(r)* non-scattered reference field, *SF* spatial filter assembly, *L* lens, *BS* beam splitter, *SLM* spatial light modulator, *CCD* charge coupled device camera

**Fig. 43:**
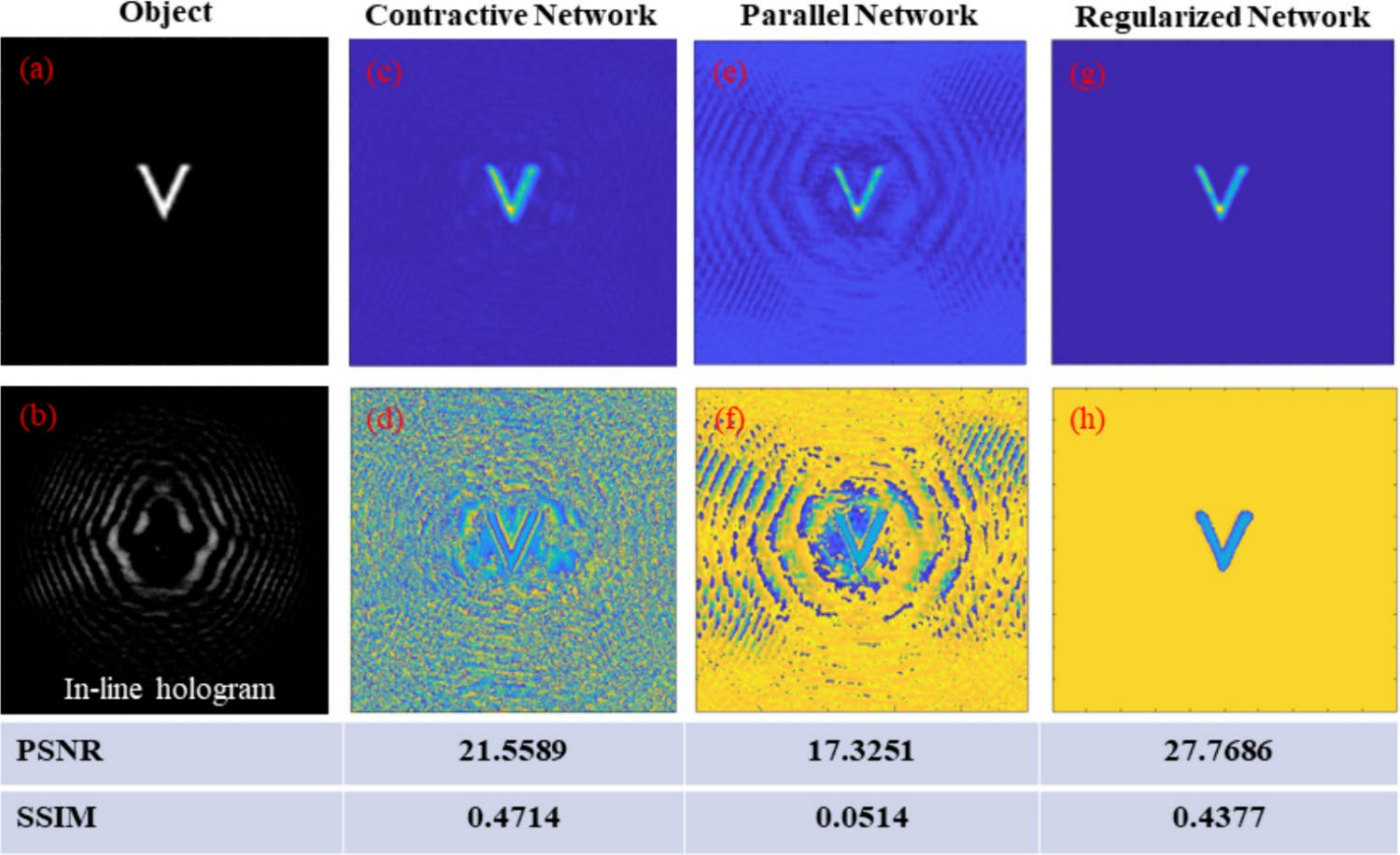
Experimental results: **a** object “V” displayed using SLM, **b** intensity distribution of recorded in-line hologram, **c**–**h** amplitude phase reconstruction results with various network architectures. The PSNR and SSIM comparison of various network architectures are given in the last two rows

The conceptual schematic of the generation and detection of an in-line hologram with coherent beam illumination on an object and the respective experimental geometry are shown in Fig. 42. A spatially filtered and collimated beam from a He–Ne laser source (Melles Griot, 25-LHP-928-230) is used as the light source for the experimental system. The complex valued object is introduced into the experimental scheme using an SLM. The intensity distribution of the in-line hologram was recorded with an image sensor at 295 mm from the object plane. In the DIH reconstruction process, the twin image artifact elimination from the recorded in-line hologram was carried out using the regularized autoencoder generative model. In addition, the approach tested various network architectures such as contractive variation network, parallel dual encoder dual decoder network, etc., and a quantitative performance analysis comparison is implemented in the respective reconstruction results of phase and amplitude information of the object. The complex-valued object, recorded in-line hologram, and reconstruction results with contractive network, parallel network, and the regularized auto-encoder network are shown in Fig. 43. In comparison to other network architectures, the regularized autoencoder architecture was found to be highly efficient and productive in the simultaneous accurate reconstruction of phase and amplitude from s single intensity distribution. The peak signal to noise ratio (PSNR) and the structural similarity index (SSIM) of different network architectures are shown in the last two rows of Fig. 43, which indicates the superior reconstruction quality of regularized auto-encoder network over other architectures.

### Conclusion and future perspectives

A novel regularized autoencoder architecture capable of single-shot reconstruction of phase and amplitude from an intensity distribution of in-line hologram has been developed. The applicability of the deep learning architecture in DIH is demonstrated with a quantitative comparison to various network architectures. The versatility of the architecture in comparison to existing methods is expected to lighten up divergent application domains in biomedical imaging, quantitative phase microscopy, digital holographic microscopy, etc. The pseudocode describing the network architecture is provided in the supplementary materials S12.

## Imaging in complex media: from wavefront shaping to computational imaging (Sylvain Gigan, Hilton Barbosa De Aguiar)

### Background

Complex Media are turbid inhomogeneous systems, where light propagation is severely affected by the refractive index fluctuations, resulting not just in aberrations, but also in scattering. They range from the atmosphere (for instance turbulent atmosphere to clouds) to the ocean, but also to materials like papers, paint, and most crucially biological tissues, which are highly heterogeneous. When trying to image at depth or through such systems, imaging with ballistic photons rapidly become extremely challenging, due to their exponential attenuation with depth [327]. Conversely, scattered light is transported through the inhomogeneous medium much deeper, being only subject to a “mild” linear attenuation with depth, in the absence of absorption. The key questions are how to tackle and mitigate the effect of scattering, and how to exploit scattered light for imaging. While diffuse incoherent optical techniques have been investigated for deep imaging [328] they only offer a poor resolution. However, the scattering process is essentially a coherent process leading to a speckle pattern, i.e. a complex interference pattern with diffraction limited features. After the seminal work of Vellekoop and Mosk in 2007 [329], a wealth of approaches have been put forward to image at depth with optical resolution exploiting scattered light. While most of the approaches have been initially exploiting physical approaches to disentangle scattered light, mostly exploiting wavefront shaping and spatial light modulators to re-focus light in tissues, the last years have seen a surge in computational approaches, where physical control of light has been increasingly complemented or even outright replaced by computational approaches. In this short chapter, we want to review some of the main algorithms that have been applied, with some highlights on the work of our team.

### Signal processing

**Fig. 44:**
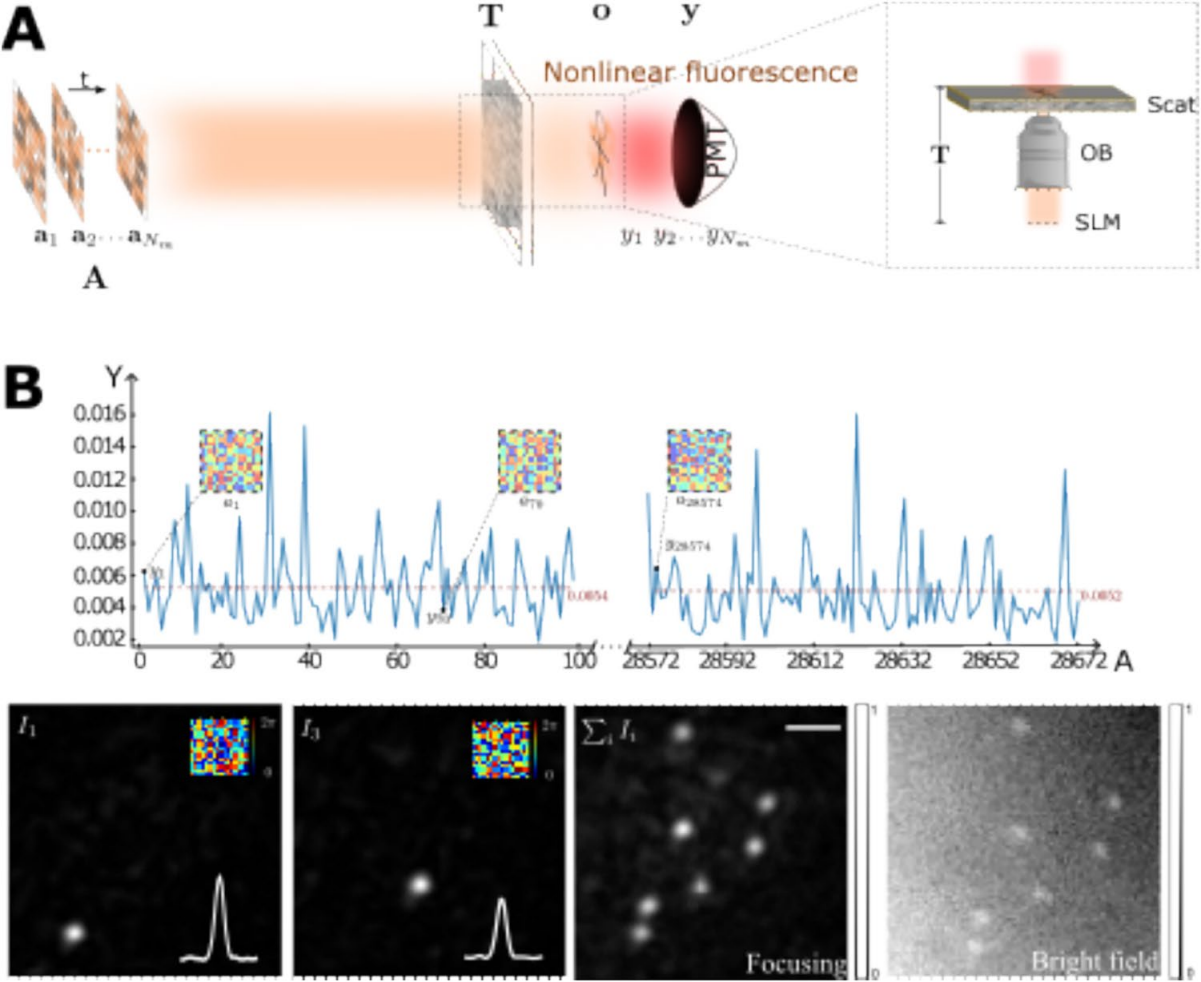
A single-pixel approach to recover the transmission matrix. **A** Simplified schematic view of a general fluorescence microscope with a single-pixel detector. Random wavefront generated by a SLM **a**_Nm_ impinges on a scattering medium with a transmission matrix **T** and excites 2-photon fluorescence (2PF) of an extended object **o**. The fluorescence signal **y** is collected by a single-pixel detector (e.g. photo-multiplier tube (PMT)). A matrix **A** is made from the known wavefronts **a**_Nm_. A gradient-descent-based algorithm is used to solve the forward problem min_**t**i_ ||**y−**∑|**At**_**i**_|^4^||^2^, where **t**_**i**_ are filtered columns of **T** by the positions of **o**. **B** Selected experimental realization. (Upper panel) 2PF signal detected vs. number of random wavefronts. Representative wavefronts used are shown in the insets. (Bottom panels) Inspected foci for two selected column after retrieval of **z z**, a sum of all images for each focus demonstrating unique single focus for each column retrieved, and comparison with brightfield image confirming the number of sources [335]. The MATLAB codes for speckle analysis and control are given in [359]

Let us first summarize the issues of imaging with scattered light that makes computational approaches appealing. The first one is obviously the scrambling of the information by the tissues, akin to a multiplication by a random matrix [330]. On the one hand, this means that recovering information is essentially an inverse problem [331], tapping into the vast literature on the topic, from regularization issues to the introduction of priors on the object to image, or on the scattering medium itself. A major aspect has been the use of compressed sensing approaches, particularly well adapted to the random nature of the transmission matrices [332] or to the sparse nature of some complex media such as multimode fibers [333]. Conversely, single-pixel approaches have also been quite successful for imaging in complex media [334, 335] (Fig. 44).

The second important ingredient is the fact that we measure intensities, while most imaging techniques require access to the phase. While certain imaging techniques are coherent and thus are amenable to direct (holographic) access to the field [336–338] most popular techniques are either incoherent-based (for instance fluorescence, and spontaneous Raman) or do not provide easy access to the phase of the light. In this case, the general framework of phase-retrieval algorithms has provided important advances [339–341], in particular exploiting the speckle correlations such as the memory effect [342–344]. We have also recently proposed advanced demixing techniques based on phase-retrieval to perform phase-conjugation from a set of incoherent fluorescent sources [345], or to retrieve the transmission matrix from multiplexed single-pixel measurements in two-photon fluorescence [335].

The last class of useful algorithms are matrix factorization and matrix completion algorithms. In particular, non-negative matrix factorization algorithms have proved effective in demixing and disentangling incoherent objects in scattering media, provided they either naturally fluctuate, as in functional imaging [346, 347], or provided we can excite them in a dynamic way [348, 349]. A recent advance in matrix factorization algorithms is matrix completion which, provided that some sparsity constraint is met, could be used for imaging as recently shown in the context of spontaneous Raman [350] (Fig. 44).

### Machine learning and artificial intelligence

While conventional signal processing algorithms have proved very effective at deep imaging in complex media, the recent rise in AI and deep learning has obviously impacted strongly the field. A first class aimed at replacing and extending conventional algorithms, in particular to solve generalized phase-retrieval problems [351, 352]. Furthermore, deep neural networks have also rapidly been used to rapidly retrieve the transmission matrix of various complex media invasively [353–355]. However, a particularly important ingredient to efficiently apply these algorithms in imaging in general, and in imaging in complex media in particular, is to enable non-invasive imaging/characterization of the transmission matrix. One way to achieve that is to include physical priors in the neural network, in other words to exploits physics-aware or physics-informed approaches [356], as illustration in Fig. 45 from [357].

**Fig. 45:**
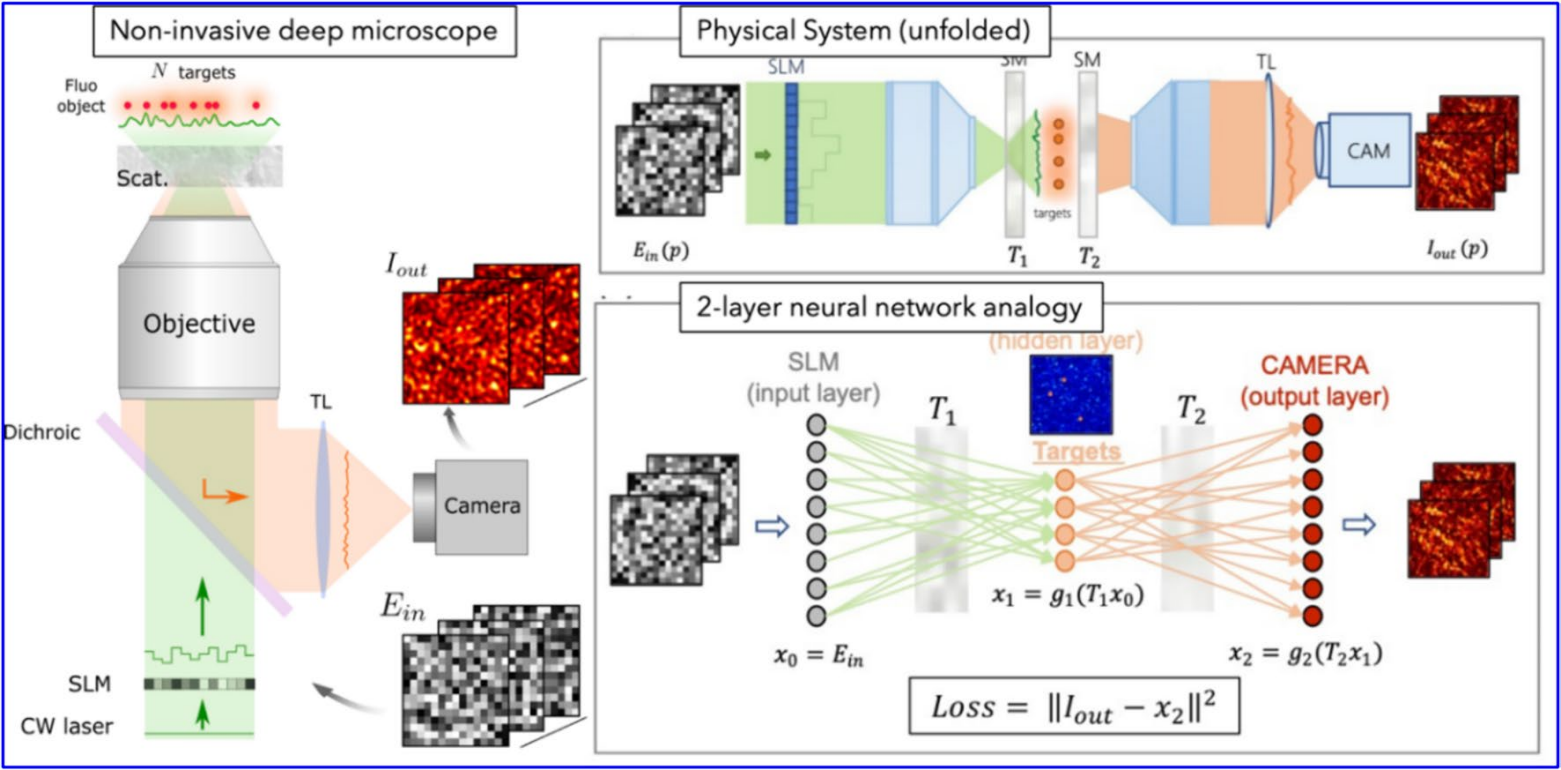
Machine learning and neural network approach to imaging in scattering media, a non-invasive deep imaging setup, where excitation light is linearly transported through a scattering medium to an object, and where the emitted incoherent light traverses back the scattering layers to be detected, can be mapped to a two-layer neural network, where the “hidden” object can be found as the hidden layer, in between two linear matrices, which can be learned from training the NN on a set of input (wavefronts) and output (camera images) (adapted from [357])

### Conclusion and future perspectives

It is clear that stemming from the seminal work of Vellekoop and Mosk [329], focusing light and scanning a focus cannot be the sole way for deep imaging. A current ongoing solution is to exploit advanced computational methods, and we reviewed some of the recent advances, with a focus on the work of our group. Looking forward, it seems probable that signal processing algorithms will tend to be replaced by (or integrated in) a more general machine learning framework, that provide similar and superior performances and a greater adaptability to various imaging modalities. There are also many exciting recent developments in machine learning for vision and imaging processing, such as diffusion model, neural fields [358], or attention-based mechanisms, that will certainly sustain the progresses in deep imaging in complex media.

## Encoding radial correlations in multimode fibers with wavefront shaping (Sarp Feykun Şener, Mert Ercan and Hasan Yılmaz)

### Background

The spatial memory effect is a speckle correlation pivotal in computational imaging through scattering materials such as biological tissue, fog, a layer of white paint, etc. [342, 343, 360, 361]. In general, the spatial memory effect refers to the property when a spatial transform is applied to a wavefront incident onto a medium, the output speckle transforms to a highly correlated speckle with respect to the initial one [361–364]. Specifically, the angular memory effect enables non-invasive imaging capabilities, particularly when traditional imaging methods fail due to medium opacity [342, 343, 360, 361]. Initially, a GS-type phase retrieval algorithm is employed to computationally reconstruct the image of an object hidden behind an opaque layer, which is based on the data obtained from a total fluorescence measurement in reflection [342]. This method is later refined to capture the same data from a single shot image captured by a camera in reflection [343].

However, the utility of the memory effect in imaging is not free from limitations. The finite memory-effect range constrains the field of view of the reconstructed images, and the field of view decreases as the thickness of the opaque layer increases. To overcome this limitation, an operator-based method was developed, which allows to realize high correlations beyond the conventional angular memory effect range at arbitrary angles through a diffusive medium [365]. While the translational memory effect is observed in square fibers [366, 367], conventional fibers do not have translational but rotational memory effect due to the rotational symmetry in their structure [368], and the memory in the radial direction is limited [369]. In this chapter, we introduce the operator-based technique into multimode fibers to encode radial memory effect at arbitrary radial translations. This technique utilizes the transmission matrix (*t*), a mathematical tool that interconnects the input field and the output field through a linear complex medium.

### Methodology

The mode decomposition method is used to simulate the wave propagation through the system and calculate the transmission matrix (*t*) of the multimode fiber. Fiber modes are initially calculated with the use of a pre-existing code [370]. As the first step, a correlation coefficient is defined by using the bra-ket notation in which ket ($|\psi \rangle$) represents vectors and bra ($\left.\langle \psi \right|$) denotes the corresponding complex conjugate and the inner product $\langle \psi |\psi \rangle$ provides a measure of their overlap. The aim is to customize the spatial memory effect to examine the correlation at the output. Therefore, we consider the inner product between the transmitted field pattern $t\left|\psi \right.\rangle$, and the one with arbitrary radial translations ${r}_{i}$ and ${r}_{o}$ for input and output, $X^{\dag } \left( {r_{o} } \right)tX\left( {r_{i} } \right)\left| \psi \right\rangle$ [363]:

17 $$C\left( {r_{i} ,r_{o} } \right) = \frac{{\left\langle \psi \right|t^{\dag } X^{\dag } \left( {r_{o} } \right)tX\left( {r_{i} } \right)\left| \psi \right\rangle }}{{\sqrt {\left\langle \psi \right|t^{\dag } t\left| \psi \right\rangle \left\langle \psi \right|X^{\dag } \left( {r_{i} } \right)t^{\dag } tX\left( {r_{i} } \right)\left| \psi \right\rangle } }}$$

where $X$ operates on the transmission matrix by translating the incoming and outgoing field profiles by ${r}_{i}$ and ${r}_{o}$, respectively. Maximum $C\left({r}_{i},{r}_{o}\right)$can be achieved for arbitrary radial translations ${r}_{i}$ and ${r}_{o}$ by shaping the initial wavefront $\left|\psi \right.\rangle$. For this purpose, a translational memory operator whose eigenvectors give the maximum correlation is defined by using the calculated transmission matrix, which can be expressed as [365]:

18 $$\hat{Q}\left( {r_{i} ,r_{o} } \right) = \left( {t^{\dag } t} \right)^{ - 1} t^{\dag } X^{\dag } \left( {r_{o} } \right)tX\left( {r_{i} } \right)$$

**Fig. 46:**
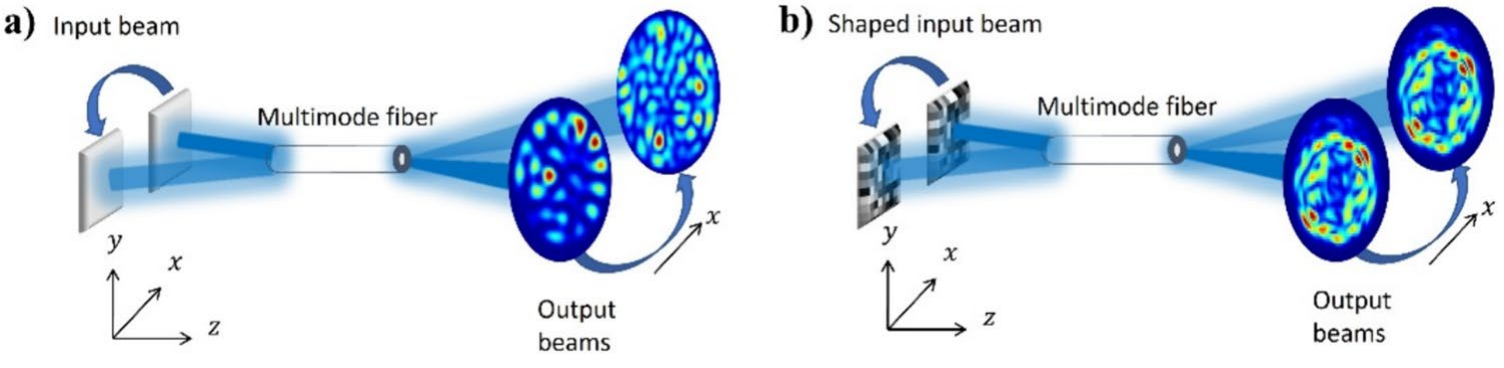
**a** Radial memory effect is not observed when a random wavefront is incident on a multimode fiber. **b** Radial memory effect is observed when the input wavefront is shaped as an eigenvector of the memory operator

By multiplying ${t}$ with ${X}\left({r_{i} } \right)$ from the right, the fields on the input facet of the fiber effectively translate while multiplying $t$ with $X^{\dag } \left( {r_{o} } \right)$ from the left translates the outputs so that $X^{\dag } \left( {r_{o} } \right)tX\left( {r_{i} } \right)$ becomes the translated version of the original transmission matrix. The expression utilizes Moore-Penrose matrix inversion as the transmission matrix is not necessarily a square matrix. Eigenstates of this operator $\widehat{Q}\left|{V}_{n}\right.\rangle ={\lambda }_{N}|{V}_{N}\rangle$ will now correspond to the fields that carry the effect we encoded in them, namely radial translational memory effect. When the input wavefront is translated in the customized direction, the output wavefront is translated in the desired direction in Fig. 46. In our case, we define our memory operator by selecting equal values for input and output translations.

### Results

**Fig. 47:**
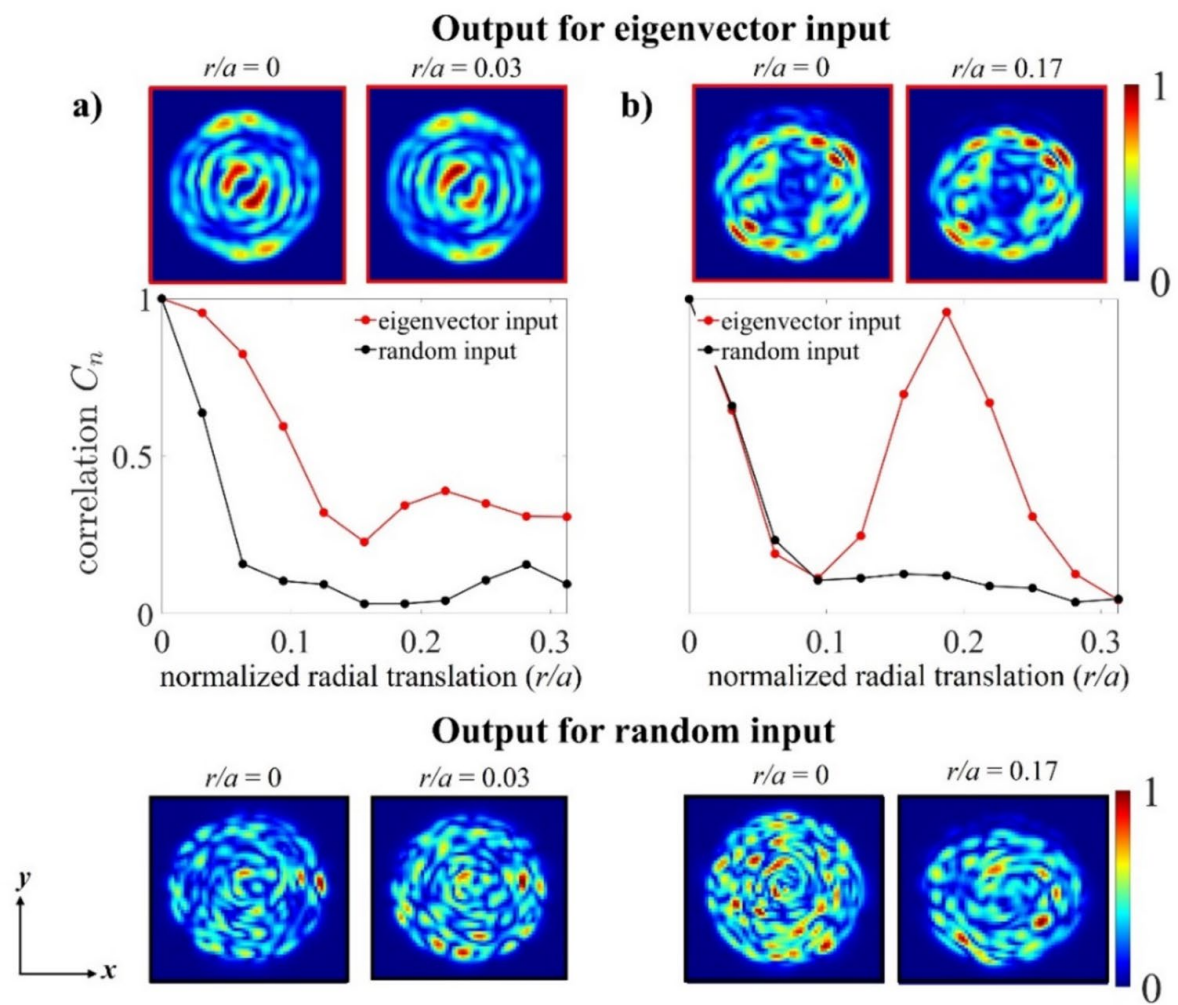
Correlation coefficient as a function of normalized radial distance *r*/*a* (*a* is the fiber radius) for the outputs of a random input field and an input eigenstate of the operator $\widehat{Q}$, for two different examples: Operator is defined at (**a**) *r*/*a* = 0.03 (**b**) *r*/*a* = 0.17 for both input and output. Coordinate system is chosen such that the radial translations project along -*y* direction

Figure 46 shows the correlation coefficient as a function of normalized radial distance for the outputs of a random input field and an input eigenstate of the operator $\widehat{Q}.$ In Fig. 47a, normalized radial translations of both input and output fields for the operator $\widehat{Q}$ are defined at *r/a* = 0.03. When the input undergoes a translation, we observe a gradual decrease in correlation at the output field, nearly double compared to what is observed with a random input. Moving to Fig. 47b, we apply the same operator definition to a normalized radial translation of *r/a* = 0.17. Here, we observe a decrease in correlation and the disappearance of our speckle pattern when the input is initially translated. However, with the continued translation of the input until reaching the defined *r*_*i*_ the original output field pattern is restored, leading to a high correlation. The output intensity patterns before and after translation show this contrast: translating the random input results in decorrelation at the output speckle, whereas the translating the input eigenvector to the target translation results in a translation of the output speckle without a significant decorrelation.

### Conclusion and future perspectives

In conclusion, we numerically encoded the translational memory effect into a multimode fiber which is typically observed in rectangular multimode fibers [366, 367] or forward scattering media [364]. We observe a non-monotonic correlation function as we increase the target translation. By combining the radial memory effect with the rotational memory effect [368], one can realize memory effect-based fiber-optic imaging. However, measuring the transmission matrix from the distant end is often infeasible in practical applications such as multimode fiber endoscopy. Recently, it is shown that the transmission matrix can be measured from the proximal end [371]. Our future work aims to define the memory effect operator by using the transmission matrix calculated from the proximal end, leveraging the optical reciprocal nature of multimode fibers. The MMF simulation tools are provided in [370]. The MATLAB code for encoding_ME, calculating the memory operator whose eigenvectors give high correlations at encoded translation value by using the transmission matrix of a fiber is given in the supplementary materials S13.

## Computational diffuse imaging using artificial intelligence (Ganesh M. Balasubramaniam, Gokul Manavalan, and Shlomi Arnon)

### Background

**Fig. 48:**
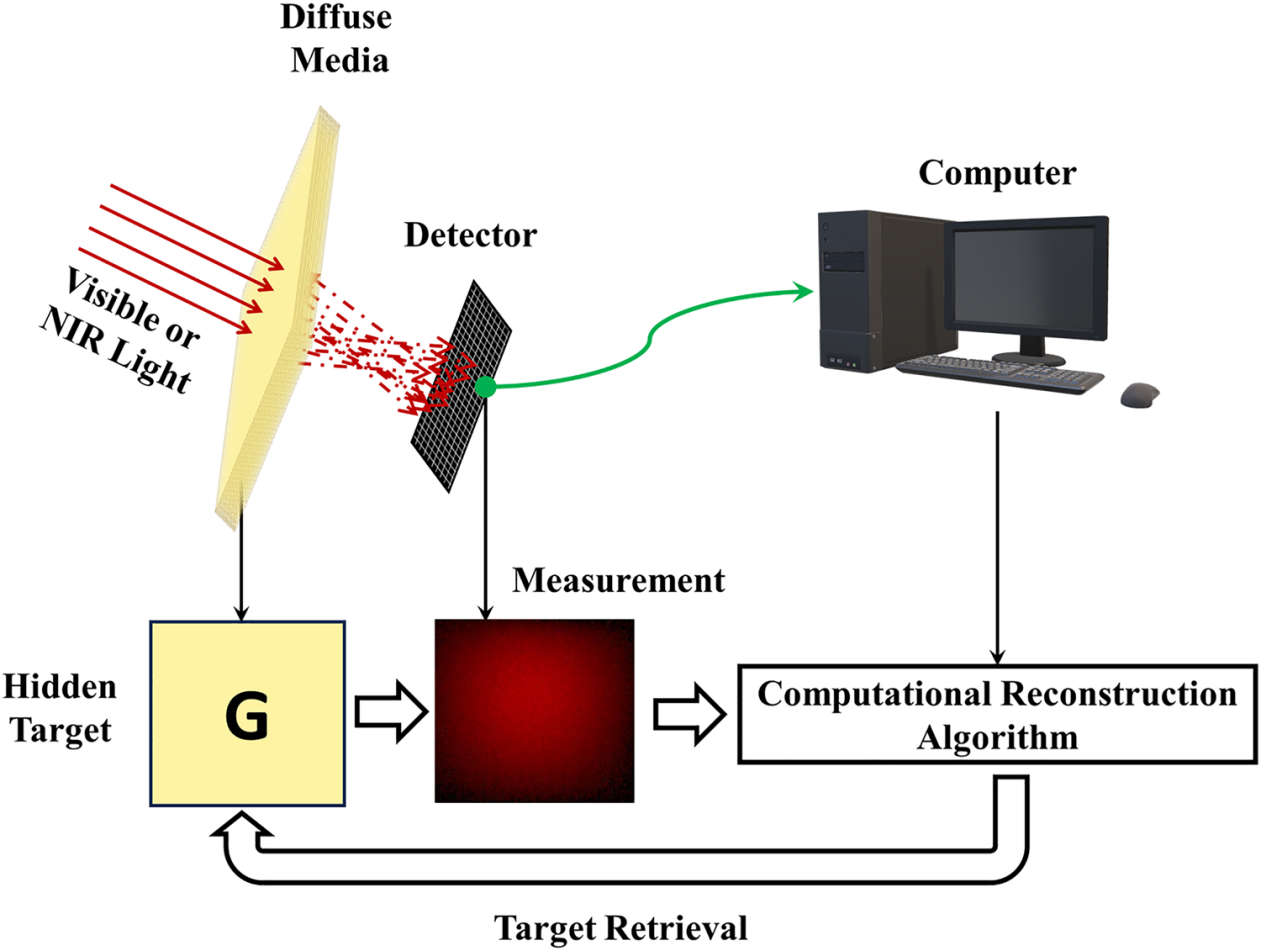
General schematic of diffuse optical imaging

Computational diffuse optical imaging is an advanced modality that uses visible or near-infra-red (NIR) light to image turbid media [372, 373]. The distinctive advantage of diffuse optical imaging lies in its non-invasive and non-ionizing nature, allowing real-time, three-dimensional visualization of tissue structure and function. In accordance with the image reconstruction approach employed, the methodology can be categorized into two distinct modalities: diffuse optical imaging (DOI), which facilitates two-dimensional imaging, and (DOT), which enables three-dimensional volumetric reconstructions of the medium [374]. The general schematic of DOI and DOT is shown in Fig. 48. This technology has found extensive applications across diverse domains, including neuroscience [375], oncology [376], and cardiovascular research [377], facilitating the study of parameters such as blood flow, tissue oxygenation, and composition [378].

Nonetheless, computational diffuse imaging has its limitations. It is constrained by limited tissue penetration depth, leading to reduced spatial resolution in deeper tissue regions. Furthermore, the scattering and absorption of light can introduce inherent inaccuracies in image reconstruction, challenging the precision of data obtained, particularly in highly inhomogeneous tissues [379, 380]. Notwithstanding these challenges, diffuse optical imaging continues to hold substantial promise and is the focus of ongoing research to overcome its limitations and expand its potential in medical and biological sciences.

One of the many solutions to address the challenges faced by diffuse imaging involves integrating various artificial intelligence (AI) techniques, an umbrella term used to represent various deep learning and machine learning methods, into the image reconstruction processes. AI encompasses a broad range of computational techniques that enable machines to perform tasks typically requiring human intelligence. ML refers to algorithms that allow systems to learn from and make predictions or decisions based on data, and DL, a subset of ML, involves neural networks with many layers that can model complex patterns in data [381, 382]. The fusion of diffuse imaging and AI signifies a promising avenue for streamlining and enhancing the imaging process, thereby amplifying the applicability and potential of computational diffuse imaging across various scientific and medical domains. The next section discusses the current status of such imaging methodologies.

### Current state-of-the-art

Contemporary scholarly literature consistently demonstrates that utilizing AI for computational diffuse imaging yields significant advantages. These advantages encompass expedited computation, simplified algorithmic implementation, and heightened accuracy in reconstructing three-dimensional volumetric data. The algorithms employed in computational diffuse imaging fall into two main categories: deep learning algorithms, such as convolutional neural networks (CNNs), and machine learning algorithms, such as gradient boosting trees. CNNs excel in autonomously learning features from data, rendering them highly valuable for image analysis and reconstruction tasks in computational diffuse imaging. These networks excel at identifying intricate patterns within large datasets, enhancing the accuracy and efficiency of image reconstruction [381, 382]. Regression-based neural networks employ regression techniques to predict continuous values, such as image pixel intensities, effectively addressing the inverse problem inherent in image reconstruction processes [382]. Moreover, machine learning (ML) algorithms, including methods such as Extreme Gradient Boosting, demonstrate significant potential in efficiently handling small datasets. These ML methods offer faster computational performance and do not require extensive computational resources, such as GPUs, making them highly suitable for various applications in diffuse imaging [377, 382]. By leveraging these AI techniques, significant improvements in image reconstruction accuracy and computational efficiency can be achieved, thereby advancing the field of computational diffuse imaging.

In 2020, Jaejun Yoo and colleagues leveraged a convolutional neural network to perform the inversion of the Lippman-Schwinger integral for diffuse optical tomography image reconstruction [383]. Their approach achieved a mean squared error (MSE) of 0.0049 ± 0.0012 and a Pearson coefficient (R) of 0.5657. More recently, Balasubramaniam et al. introduced a feed-forward networks neural network based on regression techniques to address the inverse problem in DOT [384]. The outcomes of their image reconstruction approach underscore the viability and efficiency of the regression-based neural network as a credible alternative to established numerical methods. In another study, Murad et al. conducted experimental investigations to concurrently reconstruct tissue-mimicking samples' absorption and scattering coefficients using a one-dimensional convolutional neural network (1D-CNN) [385]. Notably, incorporating basic batch normalization (BN) layers led to substantial accuracy enhancements and reduced computation time for DOT image reconstruction.

Furthermore, Mozumder et al. adopted a model-based deep learning (DL) approach to enhance the estimation of absorption and scattering coefficients in diffuse media [386]. This research demonstrated that the proposed DL method also yields significant reductions in computation time. Yongyi Zhao et al. introduced a novel learned iterative shrinkage thresholding algorithm for addressing the inverse problem in DOT. Empirical experiments on authentic datasets demonstrated that the Unrolled-DOT approach surpassed the performance of existing learning-based algorithms. Significantly, it achieved a remarkable reduction in both runtime, exceeding tenfold, and mean-squared error compared to conventional physics-based solvers [387]. Furthermore, it is noteworthy that a wealth of insightful reviews and instructive tutorials are available, offering valuable perspectives on using deep learning for reconstructing DOT images [374, 380, 388, 389].

Machine learning (ML) algorithms present another potential avenue: they excel with limited data, function efficiently without GPU support, and typically offer faster computational performance than DL methods. Zou et al. introduced an ML model incorporating physical constraints for DOT image reconstruction, showcasing substantial performance, particularly in high-contrast samples [390]. Recently, a fusion of Extreme Gradient Boosting and Genetic Programming was developed to identify anomalies in simulated heterogeneous breast tissues. The reconstructed breasts exhibited a noteworthy average cosine similarity exceeding 0.97 ± 0.07 and an average root mean square error approximating 0.1270 ± 0.0031 when compared against the ground truth [391]. Additionally, Manojlović et al. devised a machine learning algorithm capable of precise estimation of the optical properties of the skin, facilitating real-time diffuse imaging of the hand [392].

The significant findings detailed in this section underscore AI's potential role in enhancing computational diffuse imaging and broadening its applicability.

### Summary and future perspectives

In summary, we explored how recent advances in AI are being utilized to address the challenges in computational diffuse imaging, particularly in DOT. Several studies were mentioned, showcasing the promising results of AI algorithms in improving diffuse image reconstruction and estimation of optical properties. However, using AI for computational diffuse image reconstruction has drawbacks, including the need for substantial labeled data, computational complexity, risks of overfitting, limited interpretability, tuning challenges, difficulties in generalization, and integration complexities [380]. Addressing these issues is vital to fully realize the potential of AI in DOT for clinical and research applications. Continued advancements in AI algorithms, including deep learning, along with the utilization of advanced technology, such as sensitive and extensive detector arrays, large-scale laser or LED arrays, and computationally robust server systems, will likely improve the accuracy and spatial resolution of image reconstructions, enabling more detailed and informative images.

Another promising avenue for further research is integrating multi-wavelength light sources and structured illumination techniques for diffuse imaging [395–397]. Such advancements hold the potential to significantly enhance the quality of imaging, enabling more robust applications in real-world settings. A detailed explanation and code to create a simulated dataset for AI-based DOT is shown in supplementary materials S14 [391, 393, 394].

## Computational imaging with randomness (Ryoichi Horisaki)

### Background

Computational imaging is a powerful framework to innovate optical imaging systems by orchestrating optics and information science [398]. Compared with the conventional approach, where optical and computational processes are designed independently, computational imaging has improved performance and minimized optical hardware in imaging systems. Recent advancements in information science, such as deep learning, have enforced the impact of this field. I will present our recent research topics related to computational imaging with randomness.

### Imaging through scattering media

Visualization of inside or behind scattering media is important in various imaging applications, such as biomedical microscopy, astronomical observatories, security cameras, and vehicle sensors. Recently, imaging through strongly scattering media, where ballistic photons do not exist, has been studied actively [379]. Noninvasive methods, which do not need to locate a sensor or a light source for calibration or reference, are especially attractive for practical applications.

**Fig. 49:**
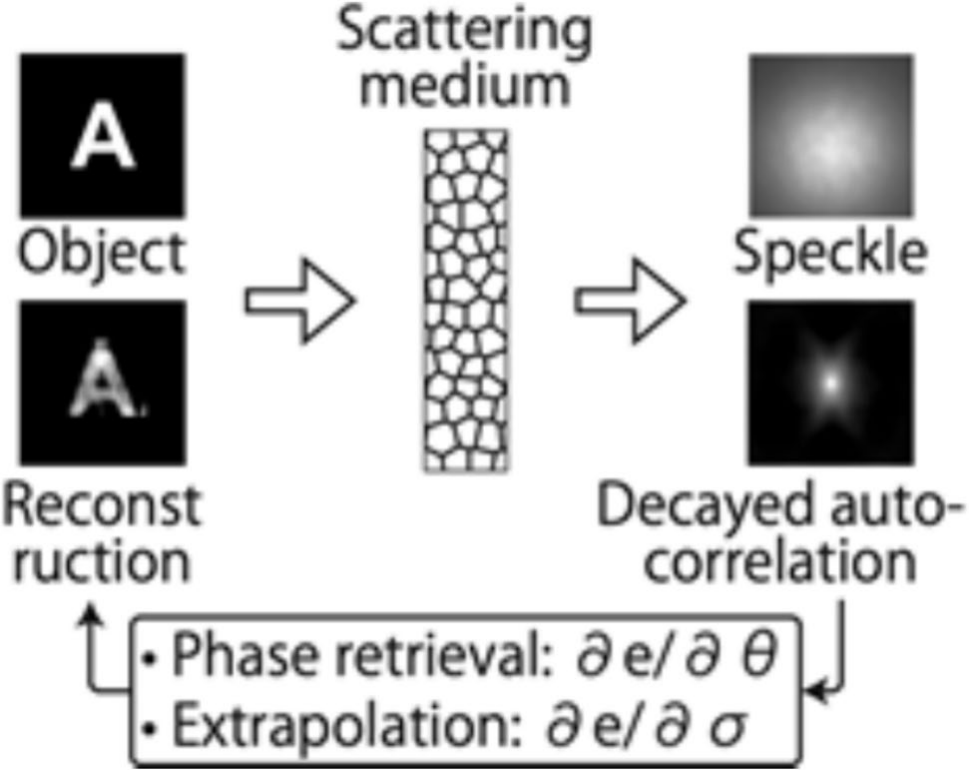
Extrapolated speckle-correlation imaging

Speckle-correlation imaging is a representative noninvasive method for imaging through scattering media [343]. Speckle-correlation imaging assumes the shift invariance of scattering impulse responses, called the memory effect. The object behind the scattering media is reconstructed from the captured speckle image by taking the autocorrelation and phase retrieval. One issue of speckle-correlation imaging is a limited field of view because the range of the memory effect is small, and the autocorrelation of the captured speckle is decayed. To solve this, we develop a gradient descent algorithm to simultaneously estimate the phase *θ* of the frequency spectrum and the decay coefficient *σ* of the speckle correlation for its extrapolation, as shown in Fig. 49 [399, 400].

The abovementioned speckle-correlation imaging techniques suppose two-dimensional objects. We extend speckle-correlation imaging to depth imaging and spectral imaging [401, 402, 403] . In these extensions, we utilize the axial memory effect and the spectral memory effect, respectively. These methods are promising for multidimensional imaging applications because of their minimal optical hardware and calibration-free process.

### Blind deconvolution

Blind deconvolution is a deconvolution technique without prior information on the impulse scattering media [404]. This problem is similar to that of speckle-correlation imaging. However, blind deconvolution does not have to suppose random and well-developed impulse responses, which is assumed in speckle-correlation imaging. Therefore, blind deconvolution is applicable to weakly scattering media.

An issue of blind deconvolution is unstableness in simultaneous estimations of the object and the impulse response. To solve this issue, we utilize a coded aperture to reduce unknown variables on the pupil plane, where light-blocked pixel values by the coded aperture on the pupil are imposed to be zeros and are not estimated in the reconstruction process [405, 406].

### Optical phase conjugation

Optical phase conjugation is a technique for controlling light behind scattering media [407]. Light passing through scattering media from a light source is time-reversely input to the scattering media, and the light source is optically reproduced behind scattering media. Conventional approaches for phase conjugation employ coherent light sources and wavefront sensors. However, these requirements increase the complexity of the optical setup.

We introduce incoherent light to optical phase conjugation to solve the above issue [408]. One problem of incoherent optical phase conjugation is a strong background light on the optically reproduced pattern due to the realness and nonnegativity of incoherent light. We develop a method for background suppression by using pixel shuffling to estimate the background light, which is computationally subtracted from the optically reproduced pattern.

### Computer-generated holography

Computer-generated holography is a technique to calculate an interference pattern, which is called a hologram, to generate a target optical field [409]. It is important for optical stimulation and tweezer for life science, laser processing for precision engineering, and three-dimensional display for virtual/augmented reality.

In general, coherent light is used for computer-generated holography. However, it raises issues on speckle noise, hardware cost, and eye harmfulness. We realize computer-generated holography with incoherent light [410]. This method calculates a hologram with a propagation model of incoherent light with random wavefronts. Three-dimensional color image reproduction is demonstrated with a chip-on-board white light-emitting diode.

### Conclusion and future perspectives

Advancements in information science enable us to utilize randomness for imaging techniques. These minimize the optical hardware and enhance imaging performance. Recent progress in optics, such as metalens, will extend the design space for further innovating computational imaging systems [411].

## Computational imaging with post-processing of the randomness (Manisha, Tanushree Karmakar, Aditya Chandra Mandal and Rakesh Kumar Singh)

### Background

Imaging through randomness is a challenging, yet practical problem, due to the scrambling of the light during propagation through a random scattering media [413–416]. The presence of scatterer places limits on the ability to image through dust, fog or atmospheric turbulence, etc. When coherent light passes through a random media, it generates a coherent noise, also known as speckle [412]. Despite the difficulties that speckle patterns introduce, significant efforts have been made to either reduce the speckles by cancellation or harness them to extract significant information embedded into the speckle grains. Several hardware-based techniques have been employed to cancel the randomness and some of these techniques are adaptive optics [417], transmission matrix [330], phase conjugation [418], etc. However, such hardware dominated methods have strict practical constraints for optimal performance of the techniques. On the other hand, an alternate route is to post-process the experimentally recorded speckle pattern [343, 419–425]. Such computational methods are free from the need to estimate and correct the wave front error and hence reduce the constraints on the experimental realization.

The post-processing of the random intensity patterns can be used to evaluate the statistical properties for the computational imaging. With the strategy to exploit the randomness, here we introduce two different works on the post-processing of the random intensity patterns for the imaging and recovering complex field information from the randomness. First is on the recovery of the complex Fourier spectrum from the second order intensity correlation and use this information for imaging [421]. The Fourier spectrum of an object obscured by the random scattering medium can be retrieved up to the diffraction limit. Second is to use the randomness for illumination and recording the hologram in the autocorrelation of the intensity rather than in the intensity and then apply the numerical reconstruction [425]. The higher order correlation provides higher spatial resolution with statistically independent light sources [425–428]. These two techniques provide an alternative approach to use randomness and apply post-processing of the random intensity patterns for quantitative imaging behind a scattering medium.

### Methodology

**Fig. 50:**
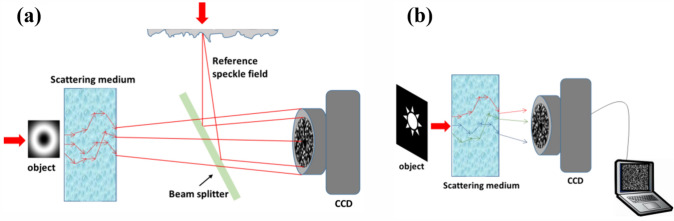
Implementation of correlations to image through **a** static, and **b** dynamic diffuser

**Fig. 51:**
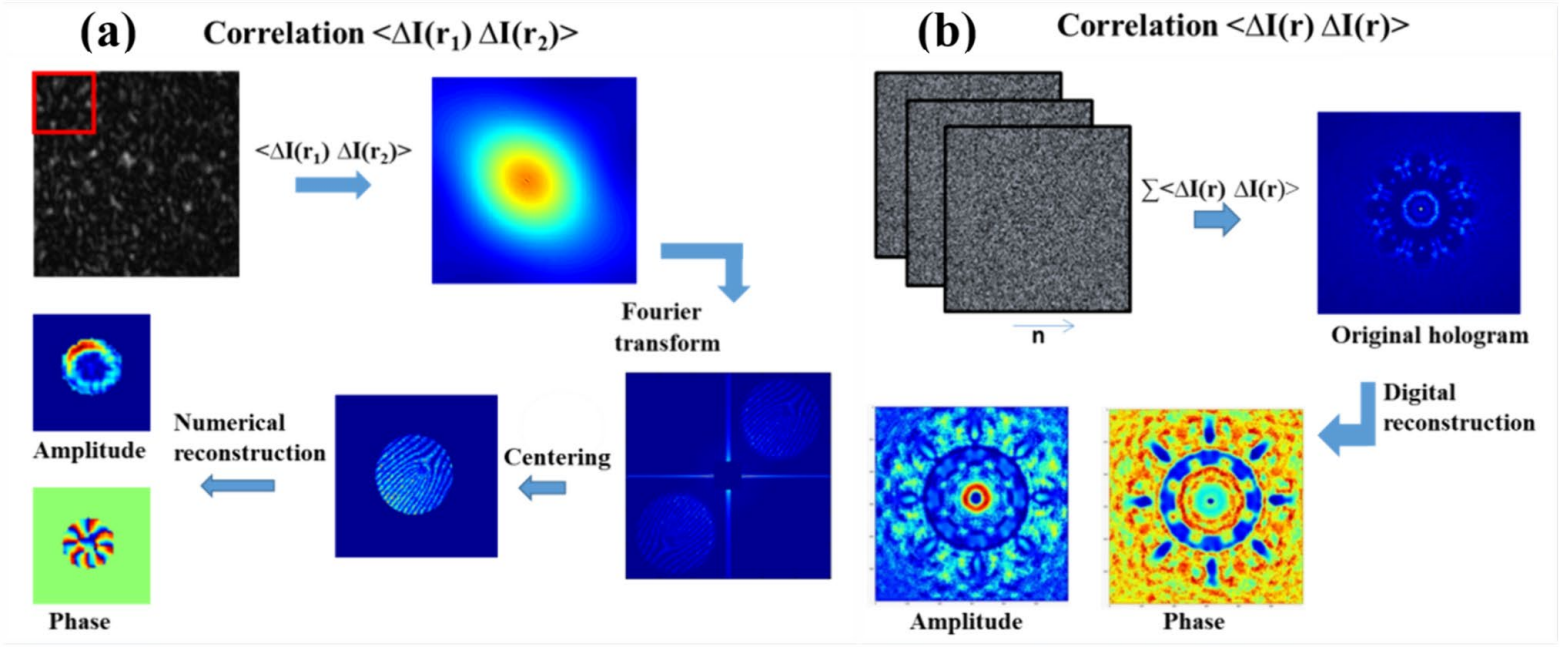
A flow chart highlighting imaging of the complex valued object from post-processing of the random intensities **a** cross-correlation of intensities using spatial averaging, **b** auto-correlation of intensities and temporal averaging

Two different strategies for computational imaging with the post-processing of the coherent random pattern are highlighted in Fig. 50. Both methods involve the two-step process. First process is recording of the coherent random pattern by a digital detector such as complimentary metal-oxide semiconductor (CMOS) or a charged coupled device (CCD) as shown in Fig. 50. For instance, Fig. 51a represents a case where an object is obscured by the random scattering medium and detector captures random intensity patterns. An instantaneous random intensity pattern, at a position vector $u$ and time t, is represented as $I\left( {u,t} \right) = \left| {E\left( {r,t} \right) \otimes h} \right|^{2}$, where $E\left(r,t\right)$ is coherent and randomly scattered object field at the scattering point $r$, $h(u-r)$ denotes the propagation kernel and $\otimes$ represents the two-dimensional convolution. The coherent field at the scattering plane is considered as $E\left(r,t\right)=O(r){e}^{i\varphi (r,t)}$, where $O\left(r\right)$ is non-stochastic object information and $\varphi (r,t)$ is a random phase introduced by the scatterer. The intensity at the detector plane, i.e. $I\left(u,t\right)$ is used to digitally evaluate the intensity cross-covariance function as $C\left(\Delta u\right)=\langle \Delta I\left(u\right)\Delta I(u+\Delta u)\rangle$, where $\Delta I\left(u\right)=I\left(u\right)-<I(u)>$ and $\langle \rangle$ represents the ensemble averaging. For the Gaussian random field, the intensity cross-covariance relates to the Fourier spectrum of the object as $C\left(\Delta u\right)={\left|FT \left\{O(r)\right\}\right|}^{2}$, where $FT$ represents the two-dimensional Fourier transform. Thus, digital evaluation of the cross-covariance provides only modulus of the Fourier spectrum of the object and phase information is lost. For recovery of the phase information and imaging behind the scattering medium, we introduce an interferometric approach and intensity pattern at the detector plane is now expressed as $I\left(u\right)={\left|E\left(u\right)+{E}_{R}(u)\right|}^{2}$, where $E\left(u\right)$ is coherent random field from the object arm and ${E}_{R}(u)$ is an independent coherent random field derived from an independent random scatterer as shown in Fig. 50a. Thus, the intensity cross-covariance is now updated as $C\left(\Delta u\right)={\left|FT\left\{O(r)\right\}+FT\left\{R(r)\right\}\right|}^{2}$, where $R(r)$ is a reference point source. This interferometric approach permits to recover the desired Fourier spectrum of the object from the intensity cross-covariance and object hidden behind the scattering media is recovered from the complex Fourier spectrum [417].

On the other hand, Fig. 50b represents a new scheme to record the hologram in terms of the auto-correlation of the intensity rather than the intensity. A single realization of the field immediately after the scatterer is represented as $E\left(r,t\right)=O(r){e}^{i\varphi (r,t)}$. The random intensity at the camera plane is represented as $I\left( {u,t} \right) = \left| {E\left( {r,t} \right) \otimes h\left( {u - r} \right)} \right|^{2}$. Now we introduce single point intensity cross-covariance as $C\left(u,u\right)=\langle \Delta I\left(u,t\right)\Delta I(u,t)\rangle$. For an incoherent light source, this single point intensity cross-covariance transforms to $C\left(u,u\right)= \mid I\left(r\right)\otimes h^{2}\mid^{2}$. For a uniform source $I\left(r\right)=1,$ the intensity correlation transforms to $C\left(u,u\right)\propto {h}^{4}$. Therefore, the correlation of intensity fluctuations is proportional to fourth power of the point spread function (PSF) of an imaging system. This results in an improved image quality in comparison to hologram recorded in the intensity [426]. We use this feature in the holography where hologram is recorded in terms of correlation of intensity fluctuations obtained by post-processing of the random patterns, and then apply digital reconstruction method to reconstruct the complex valued object hidden behind the scatterer [426].

### Results

Second process involves digital processing of the recorded random intensity patterns. The intensity cross-correlation is evaluated from the random intensity patterns as described in Fig. 51 in a flow chart. Ensemble averaging can be replaced either by temporal or spatial averaging depending on the experimental conditions. The cross-covariance function $C\left( {m,\,n; \,m + m^{\prime } ,\,n + n^{\prime } } \right)$ is evaluated as $\mathop \sum \nolimits_{k = 1}^{M} \frac{{\Delta I_{k} \left( {m,n} \right)\Delta I_{k} \left( {m + m^{\prime } ,n + n^{\prime } } \right)}}{M}$, where $\left( {m,n} \right)$represents the pixel number of the intensity pattern, and $M$ is number of different realizations of random patterns. The cross-covariance function highlights formation of fringes due to interference of coherence waves [429]. Subsequently, the Fourier fringe analysis is applied to reconstruct the object from the cross-covariance function. A flow chart highlights computations steps involved in recovery of the non-stochastic object from the randomness. On the other hand, single point correlation of the intensity fluctuations is estimated as $C\left( {m,\,n;\,m,\,n} \right) = \mathop \sum \nolimits_{k = 1}^{M} \frac{{\Delta I_{k} \left( {m,n} \right)\Delta I_{k} \left( {m,n} \right)}}{M}$. As an example, we use single point correlation of the intensity fluctuations to digitally record an inline hologram and then numerically reconstruct this hologram for reconstruction of the complex valued object. Post-processing of random intensity patterns for auto-correlation is represented in a flow chart of Fig. 51 and numerical codes for both methods are available on the GitHub (details are present in the data availability section). The twin image issue in the reconstruction of the in-line hologram is resolved by an unsupervised learning based method using an auto-encoder scheme.

### Conclusion and future perspectives

Computational imaging based on the cross-correlation and auto-correlation of the intensities are discussed. A flow chart representation is presented to describe computational steps involved in the post-processing of the random intensity patterns for utilizing the randomness in the development of new un-conventional imaging methods. These techniques are expected to find applications in imaging through randomness and in the digital holography. Codes of random illuminations for recording hologram and then applying numerical reconstruction and twin image removal are available in https://github.com/OpticsInformationLab/Random-Illumination-HolographyDL and the codes of recovery of the wavefront from spatially fluctuating fields using the two point intensity correlation, i.e., fourth order correlation are available in https://github.com/OpticsInformationLab/IntensityCorreltion and supplementary section.

## Iterative approach for aperture engineering at sharp focusing to structuring vector light (S. N. Khonina, S.G. Volotovskiy, and A.P. Porfirev)

### Background

Aperture engineering at sharp focusing conditions allows to solve different problems: improvement of resolution in imaging [430–432], optical trapping and manipulation [433, 434], laser material processing and structuring [435, 436]. To date, many algorithms have been developed for solving the inverse diffraction problem in frame of paraxial scalar theory, however, the peculiarities of sharp focusing conditions require not only to use non-paraxial propagation operators, but also to take into account the vector nature of electromagnetic radiation. These features complicate calculation algorithms, especially in the case of an iterative approach. This section discusses an iterative algorithm to calculate the vectorial input field which is necessary to achieve a wanted field distribution in the focus of a high numerical aperture system using Richards–Wolf integrals.

In this section, we present an iterative approach to solve the inverse diffraction problem under sharp focusing conditions using Richards–Wolf integrals [437]. We compute an apodization function in the entrance plane to provide vector light focusing with a defined transverse and polarization structure.

### Methodology

Suggested iterative algorithm consists of four main steps. On the first iteration vector field in the entrance of the focusing system is defined (maybe randomly) for each component ${g}_{j}^{(1)}\left(\theta ,\phi \right)$ of the electric part of light field ${{\varvec{g}}}^{(1)}\left(\theta ,\phi \right)={g}_{x}^{(1)}\left(\theta ,\phi \right){{\varvec{e}}}_{x}+{g}_{y}^{(1)}\left(\theta ,\phi \right){{\varvec{e}}}_{y}+{g}_{z}^{(1)}\left(\theta ,\phi \right){{\varvec{e}}}_{z}$. Vector field in the focal plane at *p*-th iteration ${{\varvec{E}}}^{(p)}\left(\rho ,\varphi \right)=\left({E}_{x}^{(p)}(\rho ,\varphi ),{E}_{y}^{(p)}(\rho ,\varphi ),{E}_{z}^{(p)}(\rho ,\varphi )\right)$ can be found using Richards–Wolf integrals [433]. On the next step, we apply specified constraints and replace obtained focal components with desired ones: ${\widehat{{\varvec{E}}}}^{(p)}(\rho ,\varphi )={\Omega }_{1}\left[{{\varvec{E}}}^{(p)}(\rho ,\varphi )\right]$, where ${\Omega }_{1}\left[\cdot \right]$ is a set of conditions that have an impact of amplitude and phase distribution, and also the polarization distribution. Particularly, we can apply a condition to reduce or maximize certain components, and also to concentrate intensity in a given area. After that we perform an inverse integral transformation to calculate input distribution at *p*-th iteration ${\hat{\mathbf{g}}}^{(p)} \left( {\theta ,\phi } \right)$. At the next step we apply desired constraints to the vector distribution in the input plane: ${{\varvec{g}}}^{(p+1)}\left(\theta ,\phi \right)={\Omega }_{2}\left[{\widehat{{\varvec{g}}}}^{(p)}\left(\theta ,\phi \right)\right]$ and a new iteration can be start. The iterative process stops after achieving specified goals with a certain error or execution of specified number of iterations.

### Results

**Fig. 52:**
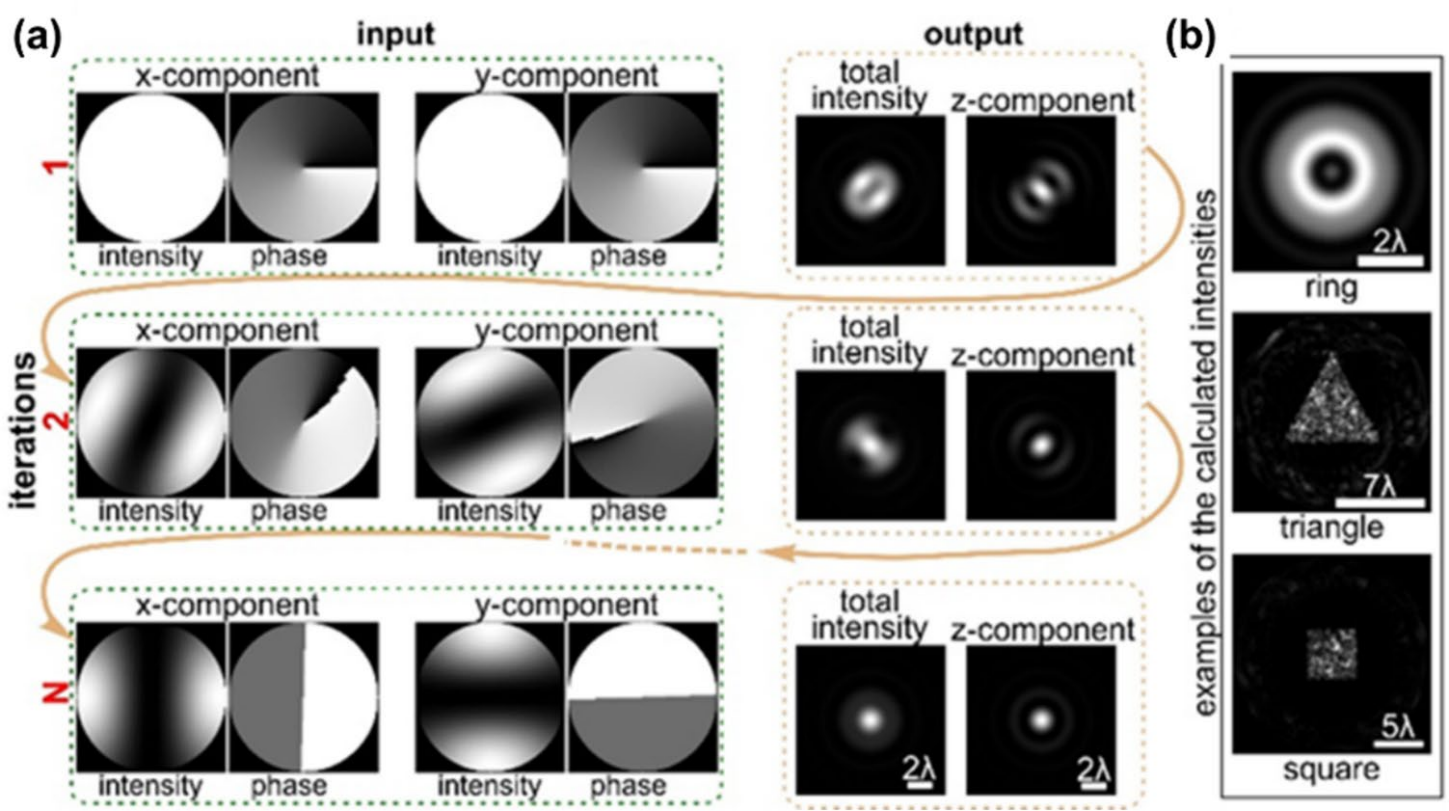
Calculation results: **a** Iterative procedure of the design of an element concentrating energy in a circle with radius 0.5 λ, **b** examples of the total intensity distributions shaped with designed elements

Results of iterative calculation of the input field that provides compact (with FWHM = 0.5 λ) energy concentration in the z-component of the focal field at sharp focusing (with numerical aperture NA = 0.99) are shown in Fig. 52a. In this case, conditions in the entrance plane ${\Omega }_{2}\left[\cdot \right]$ include only transverse components, and conditions in the focal area ${\Omega }_{1}\left[\cdot \right]$ include maximization of the longitudinal component and concentration of intensity in a circle with diameter of λ. The initial distribution we select as phase vortex functions of first order with uniform amplitude distribution: $g_{{x,y}}^{{(1)}} \left( {\theta ,\phi } \right) = \exp (i\phi )$. As can be seen, as a result of the iterative process, the input field was calculated, the transverse components of which correspond to the radial polarization [bottom line, left side of Fig. 52a]. Figure 52b demonstrates the possibility of using the developed method to calculate fields that provide vector focusing into various shapes (ring, triangle, square).

### Conclusion and future perspectives

An iterative approach for aperture engineering and solving the inverse diffraction problem under sharp focusing conditions was proposed. Designed complex distributions in the entrance of the focusing system allows one to shape desired intensity distribution in the focal area. In this case it is possible to control the z-component (longitudinal) of the shaped light field. As was recently shown, the ability to control the z-component of the field provides higher precision in laser processing of thin films of a number of photo-sensitive materials in order to fabricate the desired nano- and micro-reliefs [438].

## Four-polarisation imaging for determination of orientation beyond the spatial resolution (Soon Hock Ng, Meguya Ryu, Blake Allan, Vijayakumar Anand, Donatas Narbutis, Daniel Ierodiaconou, Junko Morikawa, Saulius Juodkazis)

### Background

E. Abbe in 1882 defined the resolution of periodic and regular features as the minimum distance apart at which given elements delineated separately $d = \frac{\lambda }{2n\sin \alpha } \equiv \frac{\lambda }{2NA}$ [439, 440], where n is the refractive index at the focal region, α is the half-angle of the focusing cone. In many applications such as Light Detection and Ranging (LIDAR), aerial and satellite imaging with low-NA optics and at long wavelengths, the resolution improvement is sought after and could extend functionalities (e.g., to add imaging capability at radio frequencies in altimeters: C-band 5.3 GHz 5.66 cm or K_u_-band 13.575 GHz 2.21 cm). Here, we discuss two distinct cases of low resolution where polarisation is invoked to determine orientation in the image (hence in the object): (1) imaging from a drone (10–120 m height) at visible spectral range and (2) imaging at THz and far-IR spectral ranges, where polarisation optical toolbox has to be improved. Formulae and numerical estimates are given here in detailed steps to explicitly show how conclusions are arrived at. We discuss future roadmap applications for polarisation resolved imaging in reflection/scattering in the case of natural light (hence, polarised) illumination. The capability to resolve orientation anisotropies beyond the spatial resolution is a promising new beginning on this roadmap.

### Methodology and results

Resolution of the optical imaging and micro-lithography d ∼ λ/NA is improved at shorter wavelength *λ* and higher numerical aperture NA. However, at long wavelengths across the IR (μm), THz (sub-mm), and radio waves (mm-cm) spectral ranges resolution is inherently low and it is even more deteriorated when low-NA optics are used for imaging, e.g., in remote sensing, Light Detection and Ranging—LIDAR, imaging from drones, and satellites. We show that the orientation features of an object can be revealed in the image using polarisation based analysis. When four polarisation images are acquired simultaneously, real-time monitoring of an optical anisotropy (in absorption reflection or scattering) and its temporal evolution becomes accessible. Polarisation analysis of acquired images considering polarisation of natural light adds complexities which could be solved using numerical deep learning and artificial intelligence (AI) approaches.

It has been proven that the orientation of a linear or circular grating (a binary on–off transmission) with period 0.2 μm can be determined in transmission images at IR chemical fingerprinting spectral range at wavelength 3.3 μm and resolution of d ∼ 5 μm  [441] using the four polarisation (4-pol.) method [442, 443], which is also applicable to optical non-propagating near-field at THz and far-IR spectral region [444]. Both, *λ* and NA did not provide the required spatial resolution d, which was 25 × lower (large d) than that required for optical resolution of the features, gratings made of 100-nm-wide gold lines on sapphire with a duty cycle of 0.5. This functionality is applied to the biomedical field to determine structural anisotropy of tissue and tumors in biopsies [445]. Importantly, the separation of absorption anisotropy due to dipole orientation can be decoupled from birefringence which has a twice faster angular dependence [446] when measured in transmission. Polarisation analysis at four polarisations separated by π/4angle is widely used in machine vision and robotics for edge detection by extracting Stokes S_1,2_ parameters [447].

**Fig. 53:**
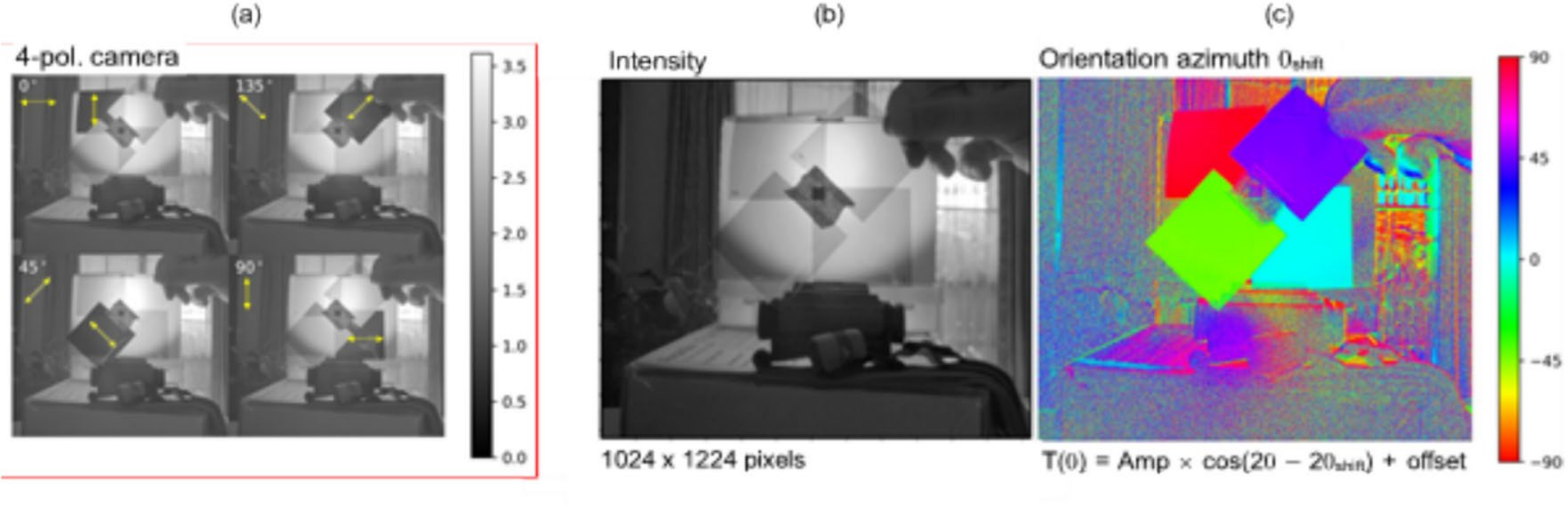
**a** Four-polarisations (4-pol.) camera (CS505MUP1 Thorlabs) images of a room scene at wire-grid linear polarisers orientation θ angles of 0, *π*/4, *π*/2, and 3*π*/4 (marked by arrows in top-left corners). Polarisers are on-chip integrated and the pixel size is 3.45 μm. The arrow markers on the dark linear polarisers (LPVISE2X2, Thorlabs) show a cross-Nikol condition since they polarise light perpendicular to the on-chip polarisers. **b** Intensity $I = \left( {I_{0} + I_{\pi /4} + I_{\pi /2} + I_{3\pi /4} } \right)/2$; camera has 12-bit resolution. **c** The azimuth θ_shift_ calculated from the transmittance best fit T(θ) = Amp × cos (2θ − 2θ_shift_) + offset for each pixel or as $\theta_{shift} = \frac{1}{2}\arctan_{2} \left( {I_{\pi /4} - I_{3\pi /4} /I_{0} - I_{\pi /2} } \right)$ are equivalent [428]

**Fig. 54:**
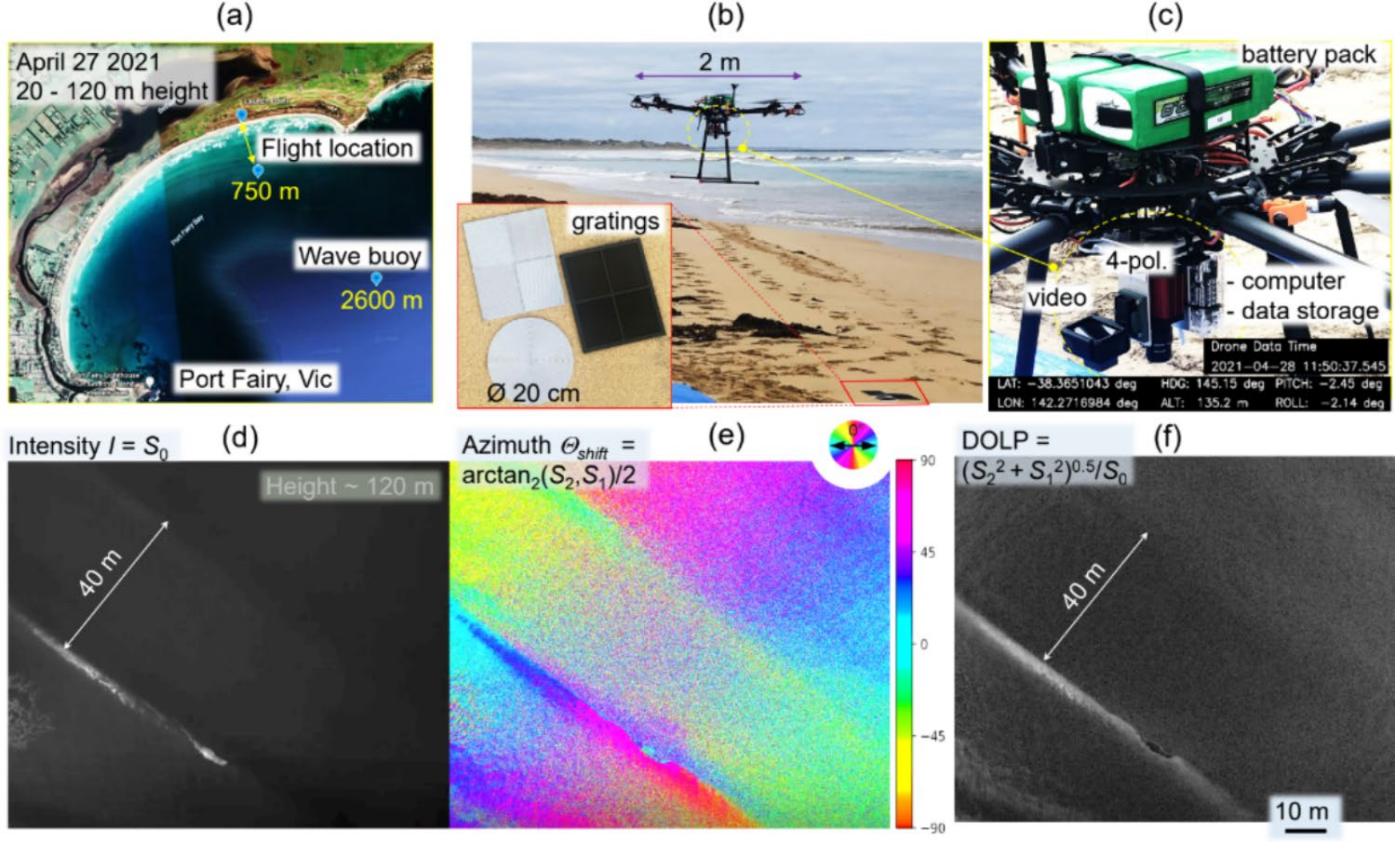
**a** Location of the drone flight near Port Fairy, Victoria at ∼ 700 m distance from the shore towards a buoy which monitors the wave height. **b** Drone hovering above beach; experiments on 27 April 2021 (afternoon). Inset shows 3D printed orientation gratings with a period of 1 mm. **c** Four-polarisations (4-pol.) camera (CS505MUP1 Thorlabs). Polarisers are on-chip integrated and the pixel size is 3.45 μm. Payload weight of the functional jig is < 0.5 kg, time of flight maximum 0.5 h, and flight distance 0.7–1 km offshore depending on visibility. Black panels at the bottom of the image show the logged information of the time, latitude LAT, longitude LON, heading HDG, altitude ALT, pitch, and roll angles. **d** Intensity or Stokes parameter ${S}_{0}=I={(I}_{0 }+{I}_{\pi /4}+{I}_{\pi /2}+{I}_{3\pi /4})/2$; camera has 12-bit resolution. **e** The azimuth θ_shift_ calculated from the best fit T(θ) = Amp × cos (2θ − 2θ_shift_) + offset for each pixel or as ${\theta }_{shift}= {\frac{1}{2}arctan}_{2}({I}_{\pi /4}-{I}_{3\pi /4}/ {I}_{0}-{I}_{\pi /2})$ are equivalent [428], S_1,2_ are Stokes parameters. **f** The degree of linear polarisation $DOLP=\sqrt{{{S}_{2}^{2}+S}_{1}^{2}}/{S}_{0}$

In transmission, the 4-pol. method works at the visible spectral range (small λ ∼ 0.5 μm) but low-NA ≡ 1/(2F_#_) ≈ 0.01 for the image analysis shown in Fig. 53. Here, the lens diameter was D = 8 mm and the f-number is defined as F_#_ = D/F where F is the focal length of the objective lens. The Abbe resolution was only d = λ/(2NA) ≈ 25 μm. Anisotropy of absorbance in polymer polarisers is clearly determined using 4-pol. analysis while the intensity image (usual camera) does not show dichroism, or different absorbance for different polarisations (in this case for the linear polarisation).The orientation azimuth θ_shift_ calculated from the transmittance best fit T(θ) = Amp × cos (2θ − 2θ_shift_) + offset for each pixel or as${\theta }_{shift}= {\frac{1}{2}arctan}_{2}({I}_{\pi /4}-{I}_{3\pi /4}/ {I}_{0}-{I}_{\pi /2})$ were shown to be equivalent [440]; here arctan_2_ is the 2-argument arctangent, which return angle in the full 2π range. Recently, it was demonstrated that use of 4-pol. camera with four polarisers at π/4 different orientations integrated on the individual pixels of CMOS chip it is possible to implement 4-pol. method at visible spectral range and low-NA for detection of structural anisotropy of objects in reflected and scattered light [448]. The imaging was made using 4-pol. camera attached to a drone. The f-number of objective lens was F_#_ = 1.4, which defines the numerical aperture NA ≡ 1/(2F_#_) = n sin α (n = 1 in the air). The light collection angle α = 23.24° defines the field-of-view (FOV) in the image for the given height H of the drone (camera) as $\text{tan}\alpha =\frac{\text{FOV}}{2}/\text{H}$. For the maximum allowed H = 120 m for a flight of a civil drone, we obtain FOV = 2H tan α = 92 m (Fig. 54). An autonomous camera-based jig was assembled with a computer and data storage for data logging (Quobasystems Pty.). Recording is triggered remotely at 433 MHz channel while flight control of the drone is at the 2.4 GHz band. The magnification of a lens is defined as the ratio between the sensor size h and the FOV: *m = h/FOV* was determined from the fixed drone height H = 444 cm with the camera 2448 × 2048 pixels of 3.45 μm size as *m* = 0.84456/339 = 2.491 × 10^ −3^ (1/m = 401.4 times); sensor width × height = 8.4456 × 7.0656 mm^2^ or optical format of “2/3” with 11 mm diagonal.

**Fig. 55:**
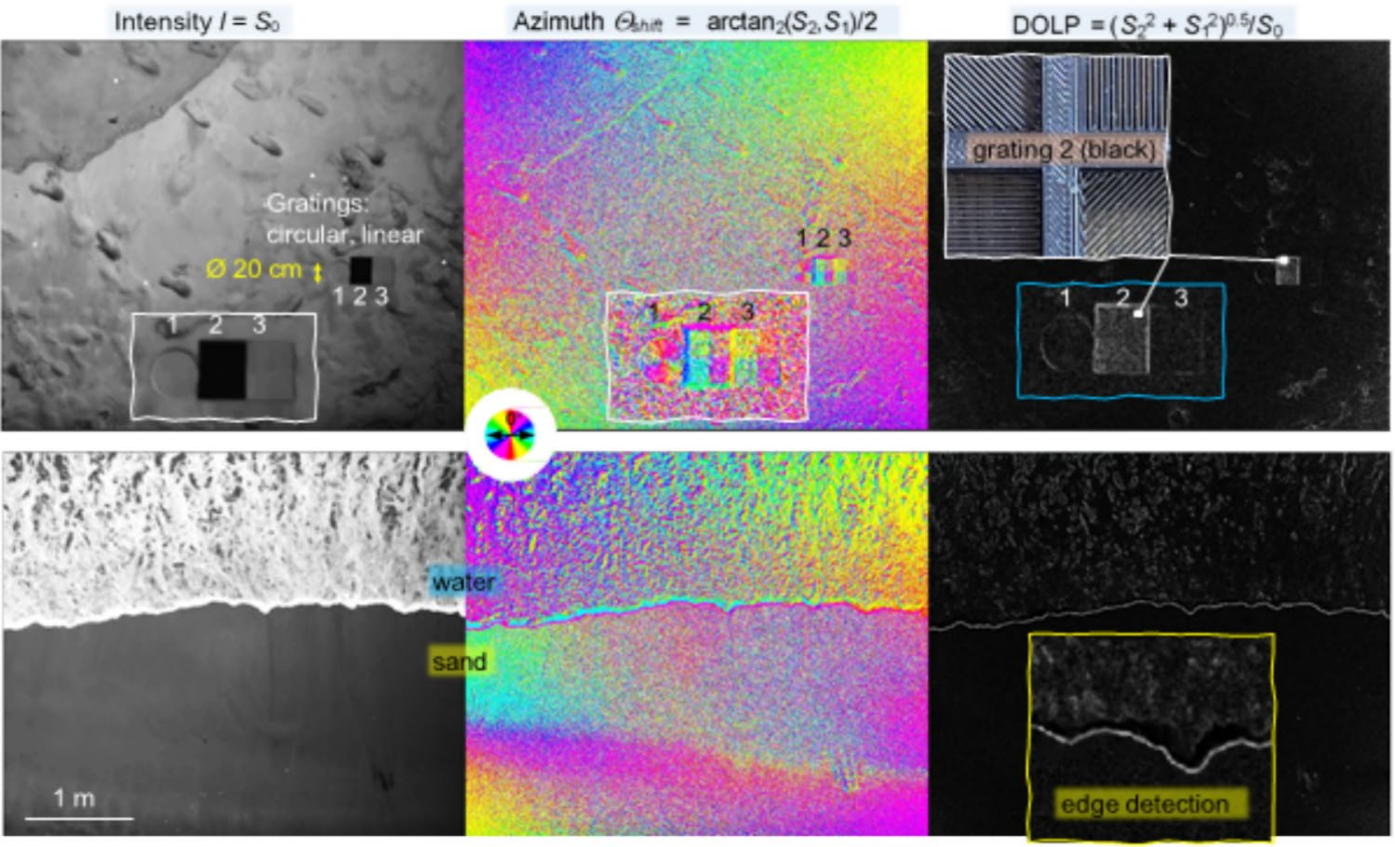
Intensity, azimuth and DOLP for image of 3D printed 1-mm-period gratings placed on beach sand and water edge on the sand drone hovering above beach at ∼ 5 m; experiments on 27 April 2021. Insets show close up views of gratings. Four-polarisations (4-pol.) camera (CS505MUP1 Thorlabs). 3D printed gratings were on a white board and transparent free-standing (black) made from a black-color feed-fiber

The absolute minimum resolvable spot size viewable on the object, the object space resolution ζ_O_ = ζ_I_/m, is defined by the image space resolution ζ_I_. For the pixel size ζ_I_ = 3.45 μm, object space resolution was ζ_O_ = 1.385 mm while the period of the 3D printed gratings was smaller at 1 mm (0.5 duty cycle). Hence, the gratings were not resolved by F_#_ = 1.4 camera from the 4.44 m drone flight. However, 4-pol. analysis allowed us to determine the azimuth of gratings’ orientation [440]. We also showed that the azimuth determined by the software of the camera is fully consistent with image analysis of separate frames taken at different four polarisations. Figure 55a shows imaging of gratings with the 4-pol. camera from ∼ 5 m height at different presentations of total intensity or Stokes S_0_, orientation azimuth θ_shift_ and DOLP, respectively. Images of the water edge region reveal different reflectivity of sand at varying water saturation (Fig. 55b). For long wavelengths, at THz sub-1 mm spectral range, it is possible to realise 4-pol. method in the attenuated total reflection (ATR) mode [449]. Non-propagating optical near-field can be used for absorbance mapping at different polarisations in the identical way as with far-field [443]. Realisation of such computer tomography (CT) based approach at long-IR THz spectral window is currently non-existent and is one of good candidates for the roadmap applications in polarisation analysis.

### Conclusion and future perspectives

Four-polarisation camera makes it possible to acquire instantaneous images for tracing time evolution of anisotropy in absorption, reflection or scattering in real time, e.g., a still image of a breaking wave discussed above. Such anisotropy can be distinguished even below spatial resolution given by the Abbe’s criterion. Since natural light is partially polarised $Pol\sim {sin}^{2}\gamma /1+{cos}^{2}\gamma$ (γ is the angle between the Sun, point of observation and camera), the imaging depends on the position of the object, its reflectivity (material, orientation, surface tilt etc.), light source (Sun light) and camera positions. The long wavelength and low-NA imaging can be improved using 4-pol. analysis with on-chip integrated linear polarisers. Among many applications, the measurement of wave height is one of the practical targets using 4-pol. method. With a fixed time stamp of azimuth image using drone and direct measurement of the wave height using dedicated sea buoy (see Fig. 53a) correlations can be established. Next roadmap applications should consider use of an artificial intelligence (AI) trained approach for the determination of the color (azimuth) distributions, which could reveal spatially non-resolved patterns of surface waves due to wind in addition to the waves of tens-of-meters. Satellites such as altimeters can expand their functionality by polarisation analysis of reflected signals at several polarisations. It is essential that images/signals of all four polarisations are acquired simultaneously. Mars Reconnaissance Orbiter HiRISE imagery around the Perseverance rover [450] shows patterns of surface relief with distinct anisotropy, which could be explored beyond the diffraction limit using the 4-pol method.

## Super-resolution imaging using structured light (Gangi Reddy Salla, Ravi Kumar, Sakshi, Inbarasan Muniraj, Shashi Prabhakar and R. P. Singh)

### Background

Structured light has been very popular in recent years due to its potential applications in microscopy and imaging, as it beats the diffraction limit [451–456]. Structured light can be controlled periodically, for instance as dots, stripes, optical random patterns, or speckles [457]. We also have various structured light fields that form optical lattices at systematic or arbitrary levels, which can be generated using computational holography [361, 452]. In microscopy, the sample is imaged using the fluorescence, which has limited resolution due to the diffraction limit [447]. With the help of structured light, one can increase the resolution beyond the diffraction limit, by at least two folds. The “confocal microscopy” technique has been effectively utilized to find the single nitrogen-vacancy centers in diamonds for producing the perfect single photon source with non-classical optical properties. Imaging beyond the diffraction limit is known as super-resolution microscopy and has gained a lot of interest for imaging nanomaterial or biological samples [454, 463]. They also have applications in imaging live cell samples, neuroscience and investigating viral structures along with their interactions with the host.

### Methodology

The first microscopic set up using structured light was introduced by Tony Wilson at Oxford University and used for generating thin optical sections in a conventional widefield microscope [456]. Later, the same was utilized for improving the resolution of the imaging set-up and overcoming the diffraction limit. This technique also provides a clearer image compared to convolutional and confocal microscopy. It has been proved that the speckles can increase the resolution and field of view, which reduces the experimental configuration. In general, the following steps are mainly involved in super-resolution microscopy using structured light: first, we need to illuminate the sample with structured light using a high numerical aperture objective lens and then collect the fluorescence light using 2-D detector array such as CCD camera. Secondly, we obtain multiple images by varying the structured light patterns or phases associated with them. Finally, we apply the de-convolution techniques and reconstruction algorithms for constructing the object information beyond the diffraction limit [452].

**Fig. 56:**
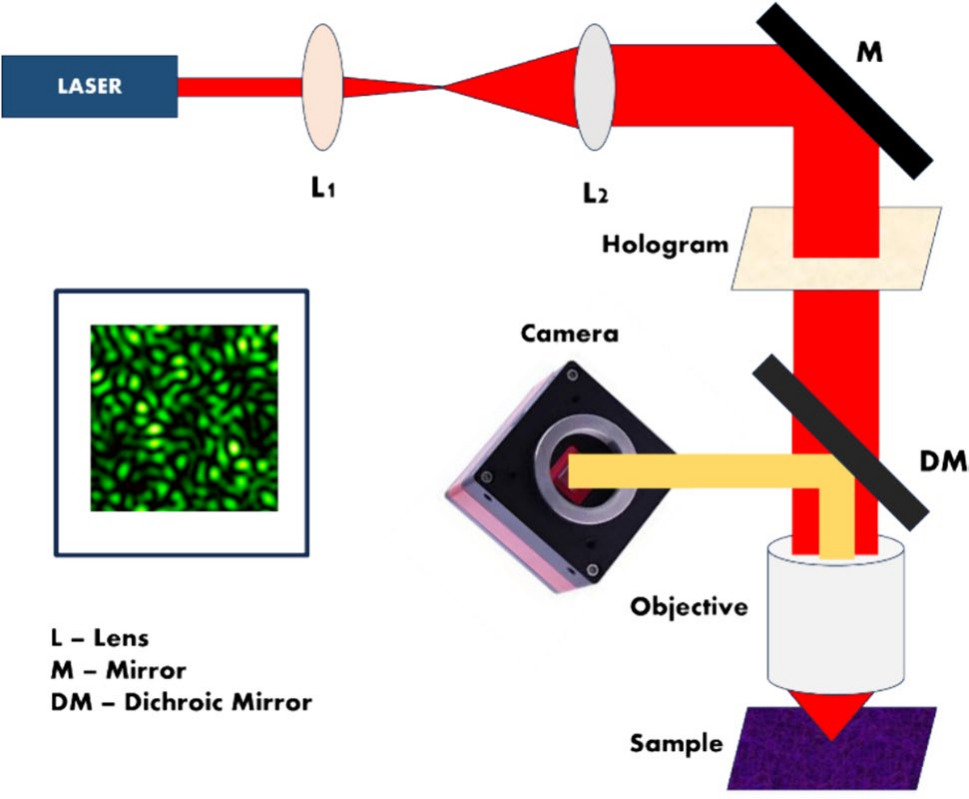
Schematic of the experimental set up used for super-resolution microscopy using structured light, and a sample of structured light is shown in the inset

The schematic for super-resolution microscopy and one type of structured light pattern (speckle) have been shown in Fig. 56. As can be seen, one needs to use a collimating lens and beam expander along with a polarizer, and then place a hologram such as a sinusoidal grating pattern for creating the structured light. The structured light can also be generated by inducing the random phase to the coherent light beam, which can be done in the lab simply by passing the light beam through a rough surface such as a ground glass plate [457–459]. To note, the proposed set-up is alignment-free along with their robustness against the aberrations, and one can reconstruct the image without having the prior information of structured pattern [361]. The object of interest can be illuminated by low-intensity laser light that generates a speckle pattern reflected from a wall. The optical memory effect can translate the generated speckle pattern on the object.

### Conclusion and future perspectives

Although super-resolution microscopy with structured light has made significant advancements, it still faces challenges, such as the need for specialized equipment and complex post-processing. Future developments in this field may involve enhancing imaging speed, improving the ease of use, and expanding its applicability to a broader range of samples. We also need to search for new structured light patterns to improve the resolution further and integrate them for live imaging of the biological cell samples. It is also observed that the random structured light can be simply generated using the random phase function. The properties such as size and distribution of these random patterns can be easily controlled by changing the phase of the coherent light beam, i.e., utilizing special light beams such a Laguerre-Gaussian, Bessel-Gaussian and perfect vortex beams [457–459]. Moreover, the effect of diffraction of the random optical pattern by using diffracting and non-diffracting speckles can also be explored to design better microscopic imaging technologies. The commented MATLAB code for simulating and propagating the random patterns generated using various types of spatial modes of light is provided in the supplementary information. MATLAB code for simulating optical random patterns is given in supplementary materials S16.

## Polarization encrypted diffractive optical elements for point spread function engineering (Vipin Tiwari and Nandan S. Bisht)

### Background

PSF engineering is a robust computational technique, that is widely used in the computational imaging domain to obtain optimized image reconstruction. PSF engineering is typically implemented by placing a tailored phase mask at the pupil plane of an imaging lens to control the emerging beam dynamics of an imaging system. For instance, imaging systems with extended Depth of Field, and low SNR are highly desirable in various interesting applications such as optical microscopy [460], beam shaping [461], computational imaging [138] for better image acquisition. One of the most common methods to obtain enhanced depth of field is to reduce the aperture size at the pupil plane, but it limits the total light throughput of the imaging system. Other interesting developments in PSF engineering include the use of cubic phase masks (CPMs) [462] and, logarithmic aspheric method [463]. However, such methods require tedious post-processing deblurring computational algorithms. In recent years, some notable studies on PSF engineering have been reported using advanced phase masks, such as Radial Quartic Phase Masks [464], and scattering diffusers [465]. Besides, extended depth of field is achieved in holography using deep learning methods with additional features, such as autofocusing and phase recovery [466]. It should be noted that the aforementioned techniques are mostly based on either amplitude or phase-coded masks. However, polarization is another interesting feature of light, which can be exploited to design novel coded masks in PSF engineering, which leads to the development of polarization-coded Apertures (PCAs).

PCA imaging has opened new trends in PSF engineering as it enables to obtaining enhanced depth of field of diffraction-limited lens with minimal power attenuation by encoding orthogonal polarization states at different portions of conventional binary DOEs [467]. However, experimental fabrication of PCA is a challenging task. Fortunately, modern electro-Opto-mechanical devices, such as SLMs and photo Elastic Modulators (PEMs) are commercially available and can be utilized to design such PCAs computationally by exploiting their modulation characteristics [468]. Recently, SLM based PCA imaging has been experimentally demonstrated using its polarization modulation characteristics for extended depth of field and axial intensity [469].

### Methodology

**Fig. 57:**
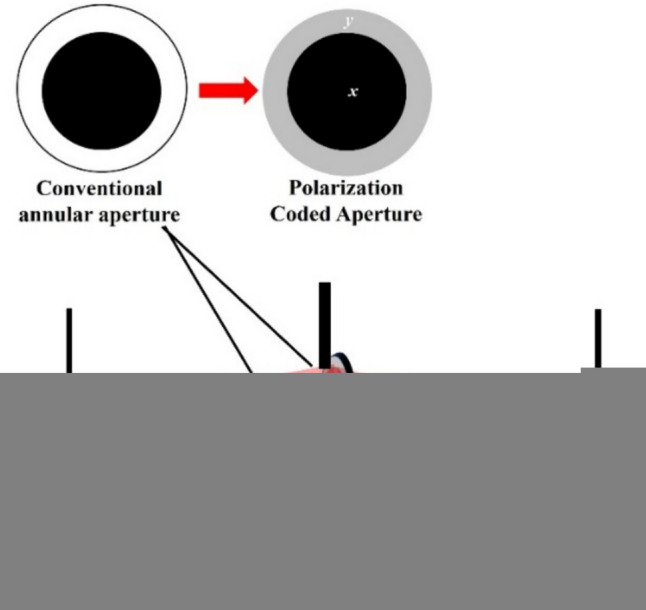
Schematic of PCA equipped imaging of diffraction-limited imaging lens. Inset shows a design of Polarization encrypted annular (ring) aperture by the OPM method

Polarization-encrypted DOEs can be designed by the Orthogonal Polarization Multiplexing (OPM) method. For instance, a PCA can be designed at the pupil plane of an imaging lens by encoding orthogonal polarization states at the central (x-polarized) and ring portion (y-polarized) of a conventional annular aperture (Fig. 57). Polarization multiplexing is beneficial as there is no interference between orthogonal polarization states at two regions of the annular aperture. Such PCA equipped imaging system results in an extended depth of field for diffraction-limited imaging lens at the detector plane [467]. On the other hand, resultant PSF can be obtained by simply adding PSFs due to both regions of PCA (central and ring) at the detector plane, which makes use of complete aperture and thus preserves the total light throughput as well.

### Results

**Fig. 58:**
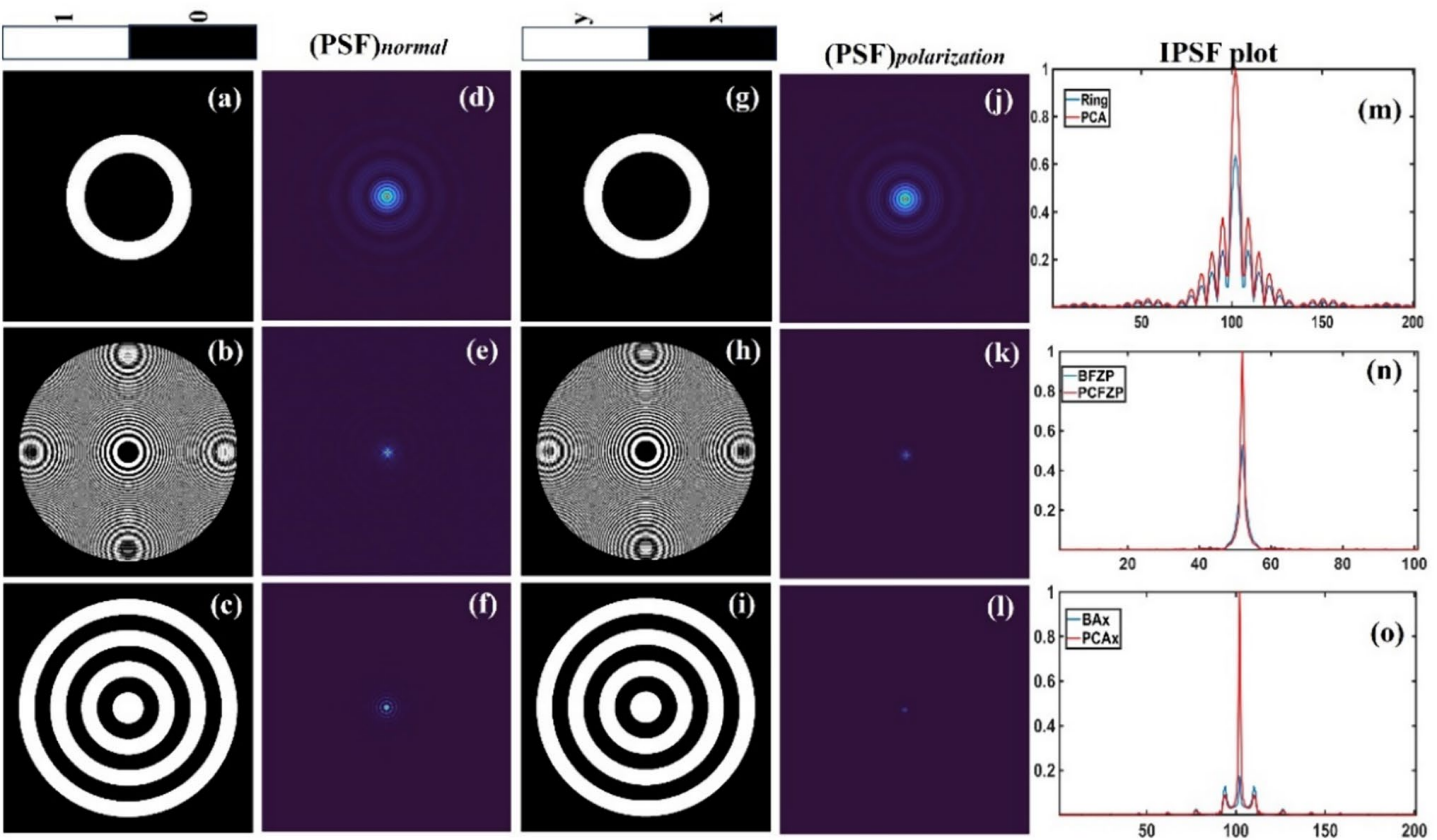
PSF engineering for polarization encrypted DOEs. **a**–**c **Conventional binary DOEs (ring, FZP, Axicon respectively) **d**–**f** corresponding PSFs respectively. **g**–**i** Polarization coded DOEs (ring, FZP, axicon respectively)**. j**–**l** Corresponding PSFs respectively. **m**–**o **Intensity plots for conventional DOEs (blue line) and polarization encrypted DOEs (red line) respectively

A comparative PSF study is carried out for conventional DOEs [ring, Binary Fresnel Zone Plate (BFZP), Binary Axicon (BAx)] and corresponding polarization encrypted DOEs [PCA, Polarization Coded Binary Fresnel Zone Plate (PCBFZP), Polarization coded Axicon (PCAx)] respectively and results are illustrated in Fig. 58. PSF plots (Fig. 58m–o) indicate a significant hike in central peak intensity for polarization-encrypted DOEs as compared to conventional DOEs.

### Conclusion and future perspectives

The inclusion of polarization-encrypted masks in conventional coded apertures (amplitude and phase) can play a pivotal role in computational imaging. Existing demonstrations support PCAs as potential candidate for obtaining improved imaging characteristics of an optical system. Moreover, PCAs exhibit the capability to utilize the maximum light throughput that can be useful in optical techniques under low illumination. Despite the exceptional light-gathering properties, experimental demonstration of PCA is a challenging task in itself. Fabrication techniques such as thin film fabrication and electro-lithography can be utilized for in-hand fabrication of such polarization-encrypted DOEs. The MATLAB codes for designing PCA is given in supplementary materials S17.

## Summary and conclusion (Joseph Rosen and Vijayakumar Anand)

In this roadmap, computational methods in four sub-fields of optical imaging and holography, namely, incoherent digital holography, quantitative phase imaging, imaging through scattering layers, and super-resolution imaging are reviewed. A total of 83 authors from the research groups of 28 prominent researchers in this field contributed to this roadmap. The above review confirms the changing trend from the need for manufacturing advanced optical components and developing optical methods to the development of computational methods. Most of the computational techniques reviewed here have overcome the limitations of manufacturing optical elements and optical methods or improving the performance of existing optical imaging and holography systems to the least extent. We believe that in addition to showing many commonalities in computational methods between different sub-fields, this roadmap shows the possibility of collaboration between fields and sets the pace for the next stages of development for optical imaging and holography.

## Electronic supplementary material

Below is the link to the electronic supplementary material.

Supplementary file1 (DOCX 1108 kb)

## Data Availability

The data can be obtained from the authors upon reasonable request.

## Appendix

See Table 1 here.

**Table 1:** List of acronyms

Serial number	Acronym	Abbreviation
1	Digital holography	DH
2	Three dimension	3D
3	Incoherent digital holography	IDH
4	Point spread function	PSF
5	Phase shifting interferometry	PSI
6	Phase shifting incoherent digital holography	PS-IDH
7	Fresnel incoherent correlation holography	FINCH
8	Single-shot phase shifting	SSPS
8	Two polarization-sensitive phase-only spatial light modulator	TPP-SLM
9	Computational coherent superposition	CCS
10	Polarization-filterless polarization-sensitive polarization-multiplexed phase-shifting incoherent digital holography	P^4^IDH
11	Light emitting diode	LED
12	Diffractive optical element	DOE
13	Random multiplexing	RM
14	Polarization multiplexing	PM
15	Signal-to-noise ratio	SNR
16	Transport of amplitude into phase using the Gerchberg Saxton algorithm	TAP-GSA
17	Degrees of freedom	DoF
18	Coded aperture imaging	CAI
19	Numerical aperture	NA
20	Lucy-Richardson-Rosen algorithm	LRRA
21	United states air force	USAF
22	Single molecule localization microscopy	SMLM
23	Self-interference digital holography	SIDH
24	Electron multiplying charge-coupled device	EMCCD
25	Light-sheet	LS
26	Light-sheet fluorescence microscopy	LSFM
27	Field of view	FOV
28	Deformable mirror	DM
29	Incoherent holographic lattice-light sheet	IHLLS
30	Lattice-light sheet	LLS
31	Phase shifting holography	PSH
32	Matrix laboratory	MATLAB
33	Sparse-view computed tomography	SV-CT
34	Compressed ultrafast tomographic imaging	CUTI
35	Two dimension	2D
36	Two-step iterative shrinkage/thresholding	TwIST
37	TwIST-based tomographic reconstructions	TTR
38	Ultra violet	UV
39	Spontaneous parametric down conversion	SPDC
40	Avalanche photo diode	APD
41	Hermite-Gauss	HG
42	Colony-forming unit	CFU
43	Plaque-forming unit	PFU
44	Complementary metal–oxide–semiconductor	CMOS
45	Thin-flim-transistor	TFT
46	Environmental protection agency	EPA
47	Vesicular stomatitis virus	VSV
48	Herpes simplex virus type 1	HSV-1
49	Encephalo myo carditis virus	EMCV
50	Computer-generated holography	CGH
51	Short-time Fourier transform	STFT
52	Red green blue-depth image	RGB-D
53	Fast Fourier transforms	FFT
54	Wavefront recording plane	WRP
55	Compressive Fresnel holography	CFH
56	Compressive sensing	CS
57	Random modulators	RM
58	Partial random ensemble	PRE
59	Random partial Fourier	RPF
60	Deep learning	DL
61	Quantitative phase imaging	QPI
62	Optical diffraction tomography	ODT
63	Refractive index	RI
64	Phase transfer function	PTF
65	Spatial bandwidth product	SBP
66	Hybrid input-output algorithm	HIO
67	Transport of intensity equation	TIE
68	Optical transfer function	OTF
69	Differential phase contrast	DPC
70	Fourier ptychographic microscopy	FPM
71	Intensity diffraction tomography	IDT
72	Transport of intensity diffraction tomography	TIDT
73	Fourier ptychographic diffraction tomography	FPDT
74	HyperSpectral	HS
75	HyperSpectral quantitative phase imaging	HSQPI
76	Complex cube filter	CCF
77	Beam splitter	BS
78	Complementary metal–oxide–semiconductor	CMOS
79	HyperSpectral phase retrieval	HSPhR
80	Space-bandwidth product	SBP
81	superluminescent light-emitting diode	sLED
82	Lensless digital holographic microscopy	LDHM
83	Angular spectrum	AS
84	Speeded-up robust features	SURF
85	Quantitative phase microscopy	QPM
86	Low coherence quantitative phase microscopy	LC-QPM
87	Optical path difference	OPD
88	Temporal phase shifting	TPS
89	Fourier transform	FT
90	Hilbert spiral transform	HST
91	Block matching 3D algorithm	BM3D
92	Improved period-guided bidimensional empirical mode decomposition	iPGBEMD
93	Mouse embryonic fibroblasts	MEF
94	Digital in-line holography	DIH
95	Peak signal to noise ratio	PSNR
96	Structural similarity index	SSIM
97	Charge coupled device	CCD
98	2-photon fluorescence	2PF
99	Photo multiplier tube	PMT
100	Near-infra-red	NIR
101	Diffuse optical imaging	DOI
102	Diffuse optical tomography	DOT
103	Artificial intelligence	AI
104	One-dimensional convolutional neural network	1D-CNN
105	Mean squared error	MSE
106	Batch normalization	BN
107	Deep learning	DL
108	Machine learning	ML
109	Full width half maximum	FWHM
110	Light detection and ranging	LIDAR
111	Four-polarisation	4-pol.
112	Degree of linear polarisation	DOLP
113	Latitude	Lat
114	Longitude	LON
115	Heading	HDG
116	Altitude	ALT
117	Attenuated total reflection	ATR
118	Computer tomography	CT
119	Cubic phase mask	CPM
120	Polarization-coded aperture	PCA
121	Photo elastic modulators	PEM
122	Orthogonal polarization multiplexing	OPM
123	Fresnel zone plate	FZP
124	Binary Fresnel zone plate	BFZP
125	Binary axicon	BAx
126	Polarization coded binary fresnel zone plate	PCBFZP
127	Polarization coded axicon	PCAx
