# Supplementary material for: Roadmap on computational methods in optical imaging and holography [invited]
Source: Appl Phys B. 2024 Aug 29;130(9):166. doi: 10.1007/s00340-024-08280-3 (PMC11362238; doi:10.1007/s00340-024-08280-3)
Supplement: Supplementary file 1 — Supplementary file1 (DOCX 1108 kb) [file 340_2024_8280_MOESM1_ESM.docx]

Supplementary Materials of Roadmap on Computational Methods in Optical Imaging and Holography

*S*1 C-code for generating spatially multiplexed Fresnel phase lens – Section 2 (Tatsuki Tahara)

#include<stdio.h>

#include<math.h>

#include<stdlib.h>

#define SIZE 2048 // Number of pixels of the image sensor and the spatial light modulator

#define LCPS 0.00000425 // Pixel pitch of the spatial light modulator

#define PI 3.141592

#define Lambda 0.000000445 // Designed wavelength

#define f 0.850 // Designed focal length

#define threshold 0.5 // Threshold value

#define PS (PI/2.0) // Phase shift

double flag[SIZE][SIZE]; // Flag for spatial multiplexing of two Fresnel phase lenses

double pl1[SIZE][SIZE],pl2[SIZE][SIZE],pl3[SIZE][SIZE],pl4[SIZE][SIZE]; // Spatially multiplexed Fresnel phase lenses

int l, k;

for (l = 0; l < SIZE; l++) {

for (k = 0; k < SIZE; k++) {

flag[l][k] = genrand_real3();

if (flag[l][k] >= threshold) {flag[l][k] = 1.0;}

else {flag[l][k] = 0.0;}

}

}

for (l = 0; l < SIZE; l++) {

for (k = 0; k < SIZE; k++) {

if (flag[l][k] >= threshold) {

pl1[l][k] = pl1[l][k] + (2.0 * PI / Lambda * ((pow((LCPS * ((double)(l) - SIZE / 2.0)), 2) + pow((LCPS * ((double)(k) - SIZE / 2.0)), 2)) / (2.0 * f)));

pl1[l][k] = atan2(sin(pl1[l][k]),cos(pl1[l][k]));

pl2[l][k] = atan2(sin(pl1[l][k]+PS),cos(pl1[l][k]+PS));

pl3[l][k] = atan2(sin(pl2[l][k]+PS),cos(pl2[l][k]+PS));

pl4[l][k] = atan2(sin(pl3[l][k]+PS),cos(pl3[l][k]+PS));

}

else {pl1[l][k] = 0.0; pl2[l][k] = 0.0; pl3[l][k] = 0.0; pl4[l][k] = 0.0;}

}

}

*S*2 C Code for conducting the phase-shifting interferometry for selectively extracting polarization information– Section 2 (Tatsuki Tahara)

#include<stdio.h>

#include<math.h>

#define SIZE 2048 // Number of pixels of the image sensor and the spatial light modulator

//1-3: phase shifts for vertical polarization, 4-6: phase shifts for horizontal polarization.

double holo0[SIZE][SIZE],

holo1[SIZE][SIZE], holo2[SIZE][SIZE], holo3[SIZE][SIZE],

holo4[SIZE][SIZE], holo5[SIZE][SIZE], holo6[SIZE][SIZE];

double verre[SIZE][SIZE]; // Real part of the vertically polarized object wave

double verim[SIZE][SIZE]; // Imaginary part of the vertically polarized object wave

double horre[SIZE][SIZE]; // Real part of the horizontally polarized object wave

double horim[SIZE][SIZE]; // Imaginary part of the horizontally polarized object wave

int l, k;

for (l = 0; l < SIZE; l++) {

for (k = 0; k < SIZE; k++) {

verre[l][k] = (holo0[l][k] - holo2[l][k]) / 2.0;

verim[l][k] = (holo1[l][k] - holo3[l][k]) / 2.0;

horre1[l][k] = (holo0[l][k] - holo5[l][k]) / 2.0;

horim1[l][k] = (holo4[l][k] - holo6[l][k]) / 2.0;

}

}

*S3* MATLAB code for implementing transport of amplitude into phase based on Gerchberg-Saxton algorithm (TAP-GSA) *– Section 3* (Shivasubramanian Gopinath, Joseph Rosen and Vijayakumar Anand)

%% Step1-Define basic parameters for calibration

N=1200; % Enter matrix size

x=-N/2:N/2-1; % Define x-co-ordinate axis

y=-N/2:N/2-1; % Define y-co-ordinate axis

pixel=8*10^-6; % Enter pixel size

lambda=0.63*10^-6; % Enter wavelength value

[X,Y]=meshgrid(x*pixel,y*pixel); % Create a calibrated workspace

R=sqrt(X.*X+Y.*Y); % Define the radius

Aperture=zeros(N,N); % Create a matrix with zeros

Aperture(R<N/2*pixel)=1; % Create a aperture

imagesc(Aperture) % Display the aperture

**%%** Step2-Design multiplexed lens and simulate intensity distribution

z1=1000; % Enter the object distance value

f1=0.14; % Enter the focal length value of first lens

f2=0.25; % Enter the focal length value of second lens

Q1=exp(1i*(pi/(lambda*z1))*(X.*X+Y.*Y)); % Propagate to distance z1

Lens1=exp(-1i*(pi/(lambda*(f1)))*(X.*X+Y.*Y)); % Lens function 1

Lens2=exp(-1i*(pi/(lambda*(f2)))*(X.*X+Y.*Y)); % Lens function 2

LensF2=Lens1+Lens2.*exp(1i*(2)*2*pi/3); % Create a phase shift to Lens2 and add Lens1 and Lens2 ; % Enter the phase shift value in ( ) either 0 or 1 or 2. Here '2' is entered.

z2=0.178; % Enter the image distance value

Field1=Q1.*LensF2.*Aperture; % Interaction

Field_11=padarray(Field1, [N/2 N/2]); % Pads the array of Field1 with zeros

Q2=exp(1i*(pi/(lambda*z2))*(X1.*X1+Y1.*Y1)); % Propagate to distance z2

Field41=ifftshift(ifft2(fft2(Field_11).*fft2(Q2))); % Take inverse fourier transform

I=Field41(N/2+1:3*N/2,N/2+1:3*N/2).*conj(Field41(N/2+1:3*N/2,N/2+1:3*N/2));% Calculate the intensity distribution

I3=I/max(max(I)); % Normalize the intensity distribution between 0 and 1

imagesc(I3) % Display the image of intensity distribution

**%%** Step3-(TAP-GSA)

Inputb=ones(N,N); % Create a matrix with ones

Matrix=angle(LensF2); % Take the Phase of LensF2

I1=I/max(max(I)); % Normalize the intensity distribution between 0 and 1

% Matrix=rand(N,N)*2*pi; % Create a random matrix and multiply by 2pi

iter=100; % Enter iteration value

Q1=exp(1i*(pi/(lambda*z1))*(X1.*X1+Y1.*Y1)); % Define Propagator to distance z1

Q2=exp(1i*(pi/(lambda*z2))*(X1.*X1+Y1.*Y1)); % Define Propagator to distance z2

Q3=exp(-1i*(pi/(lambda*z2))*(X1.*X1+Y1.*Y1)); % Define Propagator from distance z2

Q4=exp(-1i*(pi/(lambda*z1))*(X1.*X1+Y1.*Y1)); % Define Propagator from distance z1

for p=1:iter % Start for loop

p % Show iteration number

A1=Inputb.*exp(1i*Matrix).*Aperture; % Create new complex field with phase of LensF2 and uniform intensity

A11=padarray(A1,[N/2 N/2]).*Q1; % Pads the array of A1 with zeros and multiply with propagator Q1

A2=ifftshift(ifft2(fft2(A11).*fft2(Q2))); % Take inverse fourier transform

C1=angle(A2); % Take the phase of C2

C2=angle(Field41); % Take the phase of Field41

C1(601:1800,601:1800)=C2(601:1800,601:1800); % Define the DOF range

A2=padarray(sqrt(I1),[N/2 N/2]).*exp(1i*(C1)); % Pads the array of sqaure root of I1 with zeros

Matrix1=angle(Q4.*ifftshift(ifft2(fft2(A2).*fft2(Q3)))); % Take the phase of calculated field

Matrix=Matrix1(N/2+1:3*N/2,N/2+1:3*N/2); % Calculate matrix

imagesc(Matrix) % Display the image

pause(0.1) % pause to see the evolution of the solution

end % end for loop

Phase=Matrix; % Assign Matrix to Phase

Lens33=exp(1i*Phase); % Final pure phase solution

%% Step4-Create mask

M2=(angle(Lens33)+pi)/(2*pi); % Take phase of of the obtained solution and normalize it

Mask2=padarray(M2,[0 360]); % Pads the array of M2 with zeros

imwrite(Mask2,'Enter file path and specify file format'); % Save as image

*S4* MATLAB code for PSH engineering for FINCH as CAI *– Section 4* (Francis Gracy Arockiaraj, Saulius Juodkazis and Vijayakumar Anand)

%% The algorithm needs to be run twice: once for PSH engineering and once for object reconstruction

%% Step1-Read image files of PSH and Object and convert them to suitable matrix formats

A=double(imread('File location')); %Read the image file of PSH and convert it into double precision arrays

A=A(:,:,1); %Extract the channel of interest - RGB, A(:,:,1) extracts Red A(:,:,2) extracts Green and A(:,:,3) extracts Blue

A=A/max(max(A));% Normalise the matrix between 0 and 1

imagesc(A) % Display image

B=double(imread('File location'));%Read the image file of object intensity and convert it into double precision arrays

B=B(:,:,1);

B=B/max(max(B));

figure;imagesc(B)

%% Step – 2 Lucy-Richardson-Rosen algorithm

PSF=A; % Assign A to PSF

OI=B; % Assign B to Object intensity

S1 = OI; % Initial guess solution is set as the object intensity pattern

OTF = psf2otf(PSF,size(OI)); % Convert PSF to OTF

iterations = 10;% Enter the iteration number - start with say 5

figure;colormap turbo % Open new figure and set colormap turbo

for i=1:iterations %start for loop

i % show iteration number

FC = (ifft2(fft2(S1).*OTF)); % Forward Convolution

ratio = OI./FC; % Calculate ratio

ratio_f = fft2(ratio); % Fourier transform of ratio

alpha = 0.4; % Enter alpha value between 0 and 1; when alpha is 1 and beta is 1, it is Lucy-Richardson Algorithm

beta = 1; % Enter beta value between 0 and 1

residue= ifft2(conj((abs(OTF).^alpha).*exp(1i*angle(OTF))).*((abs(ratio_f).^beta).*exp(1i*angle(ratio_f)))); % Apply Non-linear reconstruction

S1 = residue.*S1; % Calculate the next solution

imagesc(abs(S1).^1); % Display the solution

pause(0.1) % pause to see the evolution of the solution

end % end for loop

result = abs(S1); % Final solution

imagesc(result) % Display solution

*S5* Python codes for SMLM-SIDH *– Section 5* (Shaoheng Li and Peter Kner)

The code for Single Molecule Localization from SIDH can be found on GitHub: <https://github.com/Knerlab/SIDH_STD> . The code consists of 6 python files for simulating changing different parameters in SIDH configurations with either one plane and one spherical wave or two spherical waves. The relevant functions for the PSF reconstruction and localization are recon(), finch_recon(), finch_recon3D(), and get_STD() which repeats the localization iteration times to determine the SMLM resolution.

S6 Python codes for illumination and detection correction – *Section 6* (Mani Ratnam Rai, Chen Li and Alon Greenbaum)

The python codes for illumination correction is given in GitHub - <https://github.com/Chenli235/AngleCorrection_Unet>. The python codes for detection correction is given in GitHub - <https://github.com/maniRr/Detection-correction>

S7 MATLAB pseudocodes for HOLO_LLS – Section 7 (Christopher Mann, Zack Zurawski, Simon Alford, Jonathan Art, and Mariana Potcoava)

1. Pseudocode for PSH and Hologram reconstruction

1. Initialization:

- Define parameters:

a. Wavelength of light (`wl`)

b. Numerical aperture of objective (`na`)

c. Camera pixel size (`dx`)

d. Determine effective pixel size after magnification (`dx_eff`)

- Set the file paths for the four phase-shift images.

2. Load phase-shift images:

- For each of the four paths:

Load the respective image into a double-precision array.

3. Compute the reconstructed intensity:

- Use the standard 4-step phase-shifting formula to derive the complex image of reconstruction.

4. Set up parameters for volumetric reconstruction:

- Define the start, end, and step size for the reconstruction depths (`z_depths`).

5. Volumetric reconstruction:

- Initialize volumes to store reconstructed slices in magnitude and phase.

- For each depth in `z_depths`:

a. Propagate the complex hologram to the current depth using the `Angular_Propagator` function.

b. Apply band-pass filtering to the propagated slice to enhance the visibility of features.

c. Normalize and store the filtered slice in the magnitude volume.

d. Store the phase of the slice in the phase volume.

e. Compute the axial intensity for the current depth (max intensity of the slice).

6. Plot the axial intensity profile:

- Create a plot showing how the axial intensity changes across the reconstruction depths.

7. Display lateral slices with scale bars:

- Determine the scale bar length in pixels based on a given physical length.

- For each reconstructed slice:

a. Create a scale bar on the slice image.

b. Display the slice with annotations.

c. Pause to allow viewing.

8. Display specific axial slices:

- Define specific depths of interest.

- For each desired depth:

a. Extract the axial slice (X-Z plane) from the magnitude volume.

b. Extract the corresponding lateral slice (X-Y plane).

c. Display the extracted slices in a subplot.

9. Create a video of axial slices:

- Initialize a video writer object.

- For each axial slice in the volume:

a. Display the slice.

b. Capture the current plot as a frame.

c. Write the frame to the video.

- Close the video writer object.

10. Display the Maximum Intensity Projection (MIP):

- Compute the MIP across the Z-dimension.

- Display the MIP image.

11. 3D Visualization of the Reconstructed Volume:

- Compute the isosurface of the reconstructed volume.

- Initialize a 3D visualization figure.

a. Apply coloring based on the Z-depth.

b. Adjust transparency settings.

c. Configure lighting and view.

d. Add labels, title, and interactivity.

- Allow the user to rotate and interact with the 3D visualization.

End.

**More details:**

% Pseudocode:

% 1. Initialization

wl = ...; % Wavelength of light

na = ...; % Numerical aperture of objective

dx = ...; % Camera pixel size

dx_eff = ...; % Effective pixel size after magnification

image_paths = {...}; % Paths to the four phase-shift images

% 2. Load Phase-Shift Images

for i = 1:4

img{i} = double(imread(image_paths{i}));

end

% 3. Compute Reconstructed Intensity

final_intensity = ...; % Compute using 4-step phase-shifting formula

% 4. Set up Parameters for Volumetric Reconstruction

z_depths = ...; % Define the depth values

reconstructed_volume = zeros(...); % Initialize volume to store slices

% 5. Volumetric Reconstruction

for z in z_depths

slice = Angular_Propagator(...); % Propagate to current depth

% Apply band-pass filtering

filtered_slice = bpass(...);

% Store the slices in the volume

reconstructed_volume(:,:,i) = filtered_slice;

% Compute Axial Intensity

AxialIntensity(i) = max(max(abs(filtered_slice)));

end

% 6. Plot Axial Intensity Profile

figure;

plot(...); % Plot axial intensity vs. reconstruction depth

% 7. Display Lateral Slices with Scale Bars

for i = 1:numel(z_depths)

slice_img = abs(reconstructed_volume(:,:,i));

% Add scale bar to the slice image

slice_img(...) = ...; % Logic to insert scale bar

% Display slice with annotations

imshow(slice_img);

title(...);

end

% 8. Display Specific Axial Slices

desired_depths = [...];

for depth in desired_depths

% Extract slices

axial_slice = ...;

lateral_slice = ...;

% Display slices in subplots

subplot(...);

imshow(axial_slice);

subplot(...);

imshow(lateral_slice);

end

% 9. Create a Video of Axial Slices

v = VideoWriter(...);

open(v);

for i = 1:size(reconstructed_volume, 3)

% Display slice, capture as frame, and write to video

imshow(...);

frame = getframe(gcf);

writeVideo(v, frame);

end

close(v);

% 10. Display MIP

mip = max(reconstructed_volume, [], 3);

imshow(mip);

title('Maximum Intensity Projection');

% 11. 3D Visualization

[f, v] = isosurface(reconstructed_volume);

figure;

patch(...); % Create and display 3D patch from isosurface data

1. Pseudocode for making diffractive lenses (IHLLS 1L and IHLLS 2L)

B1. Construct the diffractive lens for the IHLLS 1L (calibration)

function lens_1L = IHLLS1L(dx,dy,Nx,Ny,lambda,f_SLM,C1,C2)

%%%%%%%%%%%%%%%%%%%%%%%%%%%%%%%%%%%%%%%%%%%%%

% FRESNEL LENSES for SLM; IHLLS1L, Constant + Single diffractive lens.

% Authors: Christopher Mann, Zack Zurawski, Simon Alford, Jonathan Art, and Mariana Potcoava

% mpotcoav@uic.edu

% Matlab Version: R2023a

%%%%%%%%%%%%%%%%%%%%%%%%%%%%%%%%%%%%%%%%%%%%%

% Meadowlark SLM characteristics

%Display Type: Reflective LCOS (Phase Only)

%Resolution: 1920 x 1152

%Pixel Pitch: 9.2 µm

% SLM characteristics

Nx=1920;

Ny=1152;

dx=0.0092; %mm, pixel size

dy=dx;

% Focal length

f_SLM=400; %mm

lambda=0.520*10^-3; % mm

%***************************************************

%Construct the lens

[X,Y] = meshgrid(-Nx/2:1:Nx/2-1,-Ny/2:1:Ny/2-1);

xx=X.*dx; yy=Y.*dy;

R = xx.^2 + yy.^2;

R = R./(lambda*f_SLM);

lens_1L=zeros(Ny,Nx); % the phase of the lens

% need only one lens at θ = 0, just to focus the light

%lens_1L = [C_1_+C_2_ exp(iθ) Q(-1/f_SLM_ )], where Q(b)=exp[iπb λ^-1^ ( X^2^ +Y^2^ )];

% C1+C2 = 1;

theta = 0;

lens_1L= C1+C2*exp(1j*theta) *exp(-1i*pi*RR);

%lens_1L(:,:,j)=angle(lens_1L);

%lens_1L(:,:,j)=abs(lens_1L);

end

end

B2. Construct the diffractive lens for the IHLLS 2L (imaging)

function lens_2L = IHLLS2L(dx,dy,Nx,Ny,lambda,f_d1,f_d2,C1,C2)

%%%%%%%%%%%%%%%%%%%%%%%%%%%%%%%%%%%%%%%%%%%%

% FRESNEL LENSES for SLM; IHLLS2L, Dual diffractive lens with focal lengths: f_d1, f_d2

% Authors: Christopher Mann, Zack Zurawski, Simon Alford, Jonathan Art, and Mariana Potcoava

% mpotcoav@uic.edu

% Matlab Version: R2023a

%%%%%%%%%%%%%%%%%%%%%%%%%%%%%%%%%%%%%%%%%%%%

% Meadowlark SLM characteristics

%Display Type: Reflective LCOS (Phase Only)

%Resolution: 1920 x 1152

%Pixel Pitch: 9.2 µm

% SLM characteristics

Nx=1920;

Ny=1152;

dx=0.0092; %mm, pixel size

dy=dx;

% Focal lengths in mm

f_d1=465;

f_d2=555;

lambda=0.520*10^-3; % mm

%***************************************************

%Construct the lens

[X,Y] = meshgrid(-Nx/2:1:Nx/2-1,-Ny/2:1:Ny/2-1);

xx=X.*dx; yy=Y.*dy;

R = xx.^2 + yy.^2;

Rlens1 = R./(lambda*f_d1);

Rlens2 = R./(lambda*f_d2);

lens_2L=zeros(Ny,Nx,4); % the phase of the lens

% Assign random pixels, not shared, for the two lenses, example 50% / 50%

nrpixels_rand=0.5*Nx*Ny;

linearIndexes = sort(randi([1 numel(R)], nrpixels_rand, 1));

% Get locations in terms of row and columns:

[rows, columns] = ind2sub(size(R), linearIndexes);

x_rows=rows.*dx;

y_rows=columns.*dy;

% Set those points to the two lenses

Rlens1(linearIndexes)=0;% lens 1

Rlens2(Rlens1~=0)= 0;%lens 2

%lens_2L = [C1 Q(-1/fd1)+ C2 exp(iθ)Q(-1/fd1)], where Q(b)=exp[iπb λ-1 ( X2 +Y2 )]

% C1+C2 = 1;

% ===== Four - steps PSI loop =====

thetaj = [0 pi/2 pi 3*pi/2]; %phase shift, take the 2pi for checking

for j = 1 : length(thetaj)

lens_2L=C1*exp(-1i*pi*Rlens1)+C2*exp(1i*thetaj(j))*exp(-1i*pi*Rlens2);

%lens_2L(:,:,j)=angle(lens_2L);

%lens_2L(:,:,j)=abs(lens_2L);

end

end

**Examples of figures:**

A. PSH and Hologram reconstruction

A1. Maximum Intensity Projection of Reconstructed Volume
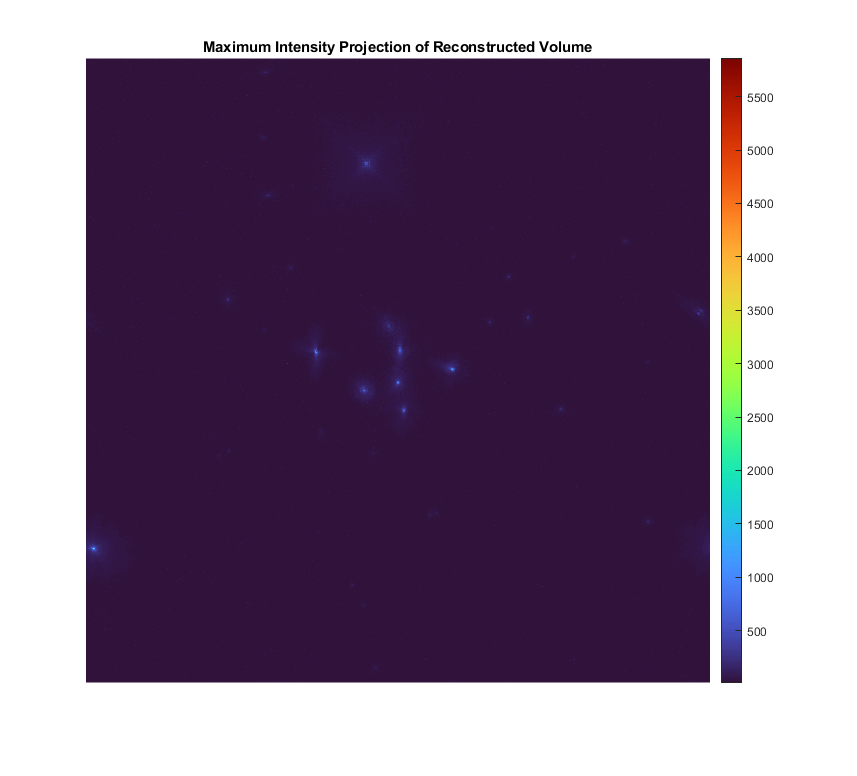


A2. 3D Visualization of Reconstructed Volume


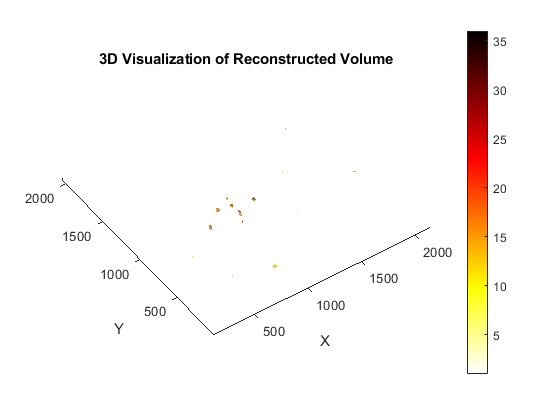


A3. Cross sections


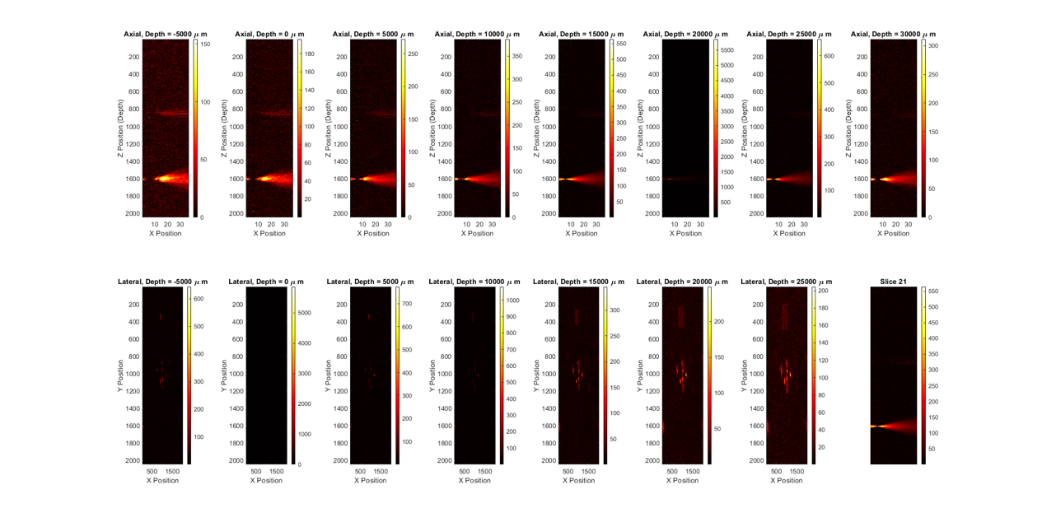


DIFFRACTIVE LENSES

B1. IHLLS 1L (single diffractive lens)


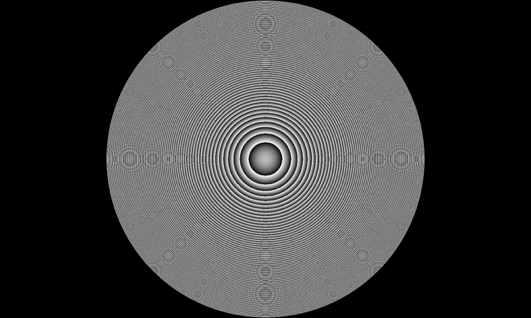


B2. IHLLS 2L (dual diffractive lens)


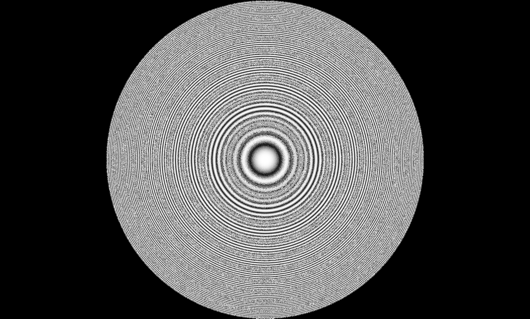

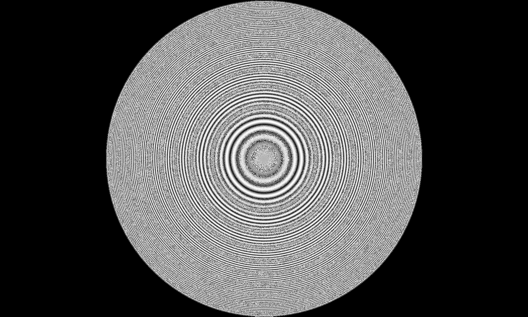


θ=0; θ = π/2;


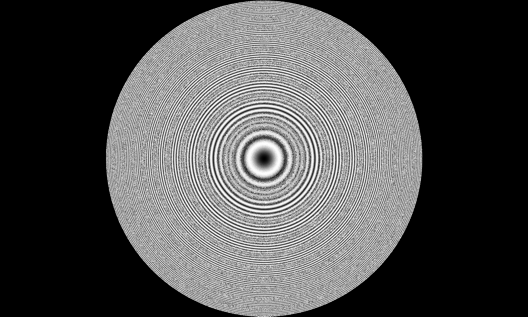

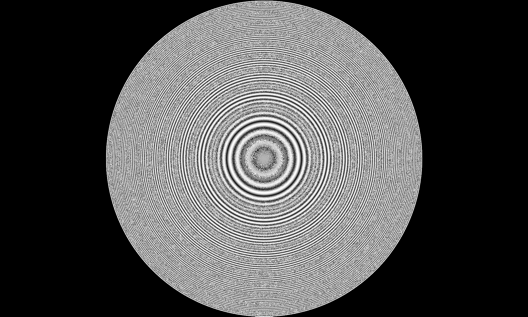


θ = π; θ = 3π/2;

S8 Matlab code for implementing TwIST-based tomographic reconstruction (TTR) – Section 8 (Yingming Lai and Jinyang Liang)

%%%% algorithm of compressed ultrafast tomographic imaging (CUTI)

%%%% Reference: https://doi.org/10.1364/OL.420737

%%%% =====================================

%%%% This script requires the package of the TwIST algorithm available at

%%%% http://www.lx.it.pt/~bioucas/TwIST/TwIST.htm

%%%% =====================================

%%%% The simulation and several customized functions are required. They are available at

%%%% https://github.com/YM-Lai-16/Extra-functions-for-implementing-compressed-ultrafast-tomographic-imaging

%%%% The customized functions should be put into the same folder of "TwIST.m"

%%%% =====================================

close all; clear all; clc

Norm = @(x) (x-min(x(:)))./(max(x(:))-min(x(:))); % Create a normalization function

addpath(genpath('TwIST_v2')); % Load the folder includes the package of TwIST

%% Simulate the SVCT spatiotemporal projections based on streak imaging

load('MoonPhase.mat'); % Load the simulated event

Event = Norm(MoonPhase); % Normalize the simulated event

[N_y0, N_x0, N_t] = size(Event); % Get the size of the event

V_S1 = 1; % S1: shear 1 pixel per frame along the downward direction

D_shearing_y_S1 = abs(round(V_S1.*N_t)); % Sheared distance at the sweeping speed S1

V_S2 = 0.8; % S2: shear 0.5 pixel per frame along the downward direction

D_shearing_y_S2 = abs(round(V_S2.*N_t)); % Sheared distance at the sweeping speed S2

V_S3 = 0.5; % S3: shear 1 pixel per frame along the downward direction

D_shearing_y_S3 = abs(round(V_S3.*N_t)); % Sheared distance at the sweeping speed S3

V_S4 = -1; % S4: shear 0.5 pixel per frame along the upward direction

D_shearing_y_S4 = abs(round(V_S4.*N_t)); % Sheared distance at the sweeping speed S4

V_S5 = -0.8; % S5: shear 0.8 pixel per frame along the upward direction

D_shearing_y_S5 = abs(round(V_S5.*N_t)); % Sheared distance at the sweeping speed S5

V_S6 = -0.5; % S6: shear 0.8 pixel per frame along the upward direction

D_shearing_y_S6 = abs(round(V_S6.*N_t)); % Sheared distance at the sweeping speed S6

%%%% Pad zeros to the datacube.

%%%% Downward: direction of padarray is 'post', which pads after the last array element

%%%% Upward: direction of padarray is 'pre', which pads before the first array element

%%%% If the shearing directions change, assign 'post' or 'pre' accordingly

for i = 1:N_t

im_S1 = padarray(Event(:,:,i), [D_shearing_y_S1, 0], 0, 'post'); % Pad zeros to the bottom of the datacube

im_cube_S1(:,:,i) = circshift(im_S1, [round(V_S1*(i-1)) 0]); % Shear each frame downward at the sweeping speed S1

im_S2 = padarray(Event(:,:,i), [D_shearing_y_S2, 0], 0, 'post'); % Pad zeros to the bottom of the datacube

im_cube_S2(:,:,i) = circshift(im_S2, [round(V_S2*(i-1)) 0]); % Shear each frame downward at the sweeping speed S2

im_S3 = padarray(Event(:,:,i), [D_shearing_y_S3, 0], 0, 'post'); % Pad zeros to the bottom of the datacube

im_cube_S3(:,:,i) = circshift(im_S3, [round(V_S3*(i-1)) 0]); % Shear each frame downward at the sweeping speed S3

im_S4 = padarray(Event(:,:,i), [D_shearing_y_S4, 0], 0, 'pre'); % Pad zeros to the top of the datacube

im_cube_S4(:,:,i) = circshift(im_S4, [round(V_S4*(i-1)) 0]); % Shear each frame upward at the sweeping speed S4

im_S5 = padarray(Event(:,:,i), [D_shearing_y_S5, 0], 0, 'pre'); % Pad zeros to the top of the datacube

im_cube_S5(:,:,i) = circshift(im_S5, [round(V_S5*(i-1)) 0]); % Shear each frame upward at the sweeping speed S5

im_S6 = padarray(Event(:,:,i), [D_shearing_y_S6, 0], 0, 'pre'); % Pad zeros to the top of the datacube

im_cube_S6(:,:,i) = circshift(im_S6, [round(V_S6*(i-1)) 0]); % Shear each frame upward at the sweeping speed S6

end

E_0 = Norm(sum(Event, 3)); % Spatiotemporal integration of the unsheared datacube

E_1 = Norm(sum(im_cube_S1, 3)); % Spatiotemporal integration of datacube sheared at the sweeping speed S1

E_2 = Norm(sum(im_cube_S2, 3)); % Spatiotemporal integration of datacube sheared at the sweeping speed S2

E_3 = Norm(sum(im_cube_S3, 3)); % Spatiotemporal integration of datacube sheared at the sweeping speed S3

E_4 = Norm(sum(im_cube_S4, 3)); % Spatiotemporal integration of datacube sheared at the sweeping speed S4

E_5 = Norm(sum(im_cube_S5, 3)); % Spatiotemporal integration of datacube sheared at the sweeping speed S5

E_6 = Norm(sum(im_cube_S6, 3)); % Spatiotemporal integration of datacube sheared at the sweeping speed S6

I_frame = ones(N_y0, N_x0);

for i = 1:N_t

Full_vector(:,i) = I_frame(:); % Generate an all one matrix the same to the event

end

%% Create the forward model of E_1

Size_y_S1 = N_y0 + D_shearing_y_S1; % Size in the y-direction after temporal shearing

Size_x_S1 = N_x0; % Size in the x-direction after temporal shearing

for i = 1:N_t

if V_S1 >= 0

shift_y_S1(i) = round((i-1).*V_S1); % The shearing distance of each frame along the downward direction

else

shift_y_S1(i) = D_shearing_y_S1+round((i-1).*V_S1); % The shearing distance of each frame along the upward direction

end

shift_x_S1(i) = 0; % The shearing distance of each frame in the x-direction

end

count = 1;

for i = 1:N_t % t

for k = 1:N_x0 % x

for j = 1:N_y0 % y

y_coordinate(count) = j + (k-1)*Size_y_S1 + shift_y_S1(i)+ ...

Size_y_S1*(shift_x_S1(i)); % Assign the y-coordinate according to the sensing operator

x_coordinate(count) = (i-1)*N_y0*N_x0 + (k-1)*N_y0 + j;

t_coordinate(count) = Full_vector(j + (k-1)*N_y0, i);

count = count + 1;

end

end

end

S_1=sparse(y_coordinate,x_coordinate,t_coordinate,...

Size_x_S1*Size_y_S1,N_y0*N_x0*N_t); % Generate the sensing operator at the shearing speed of S1

y_verify_S1 = Norm(reshape(S_1*Event(:),Size_y_S1,Size_x_S1)); % Create a measurement to verify the constructed forward model

figure, imagesc(abs(y_verify_S1 - E_1)); axis equal;axis off;colormap parula, title('Verify S1'); % All the elements should be canceled to be zero

clear x_coordinate;clear y_coordinate;clear t_coordinate;

%% Create the forward model of E_2

Size_y_S2 = N_y0 + D_shearing_y_S2; % Size of y after temporal shearing

Size_x_S2 = N_x0; % Size of x after temporal shearing

for i = 1:N_t

if V_S2 >= 0

shift_y_S2(i) = round((i-1).*V_S2); % The shearing distance of each frame along the downward direction

else

shift_y_S2(i) = D_shearing_y_S2+round((i-1).*V_S2); % The shearing distance of each frame along the upward direction

end

shift_x_S2(i) = 0; % The shearing distance of each frame in the x-direction

end

count = 1;

for i = 1:N_t % t

for k = 1:N_x0 % x

for j = 1:N_y0 % y

y_coordinate(count) = j + (k-1)*Size_y_S2 + shift_y_S2(i)+ ...

Size_y_S2*(shift_x_S2(i)); % Assign the y-coordinate according to the sensing operator

x_coordinate(count) = (i-1)*N_y0*N_x0 + (k-1)*N_y0 + j;

t_coordinate(count) = Full_vector(j + (k-1)*N_y0, i);

count = count + 1;

end

end

end

S_2=sparse(y_coordinate,x_coordinate,t_coordinate,...

Size_x_S2*Size_y_S2,N_y0*N_x0*N_t); % Generate the sensing operator at the shearing speed of S2

y_verify_S2 = Norm(reshape(S_2*Event(:),Size_y_S2,Size_x_S2)); % Create a measurement to verify the constructed forward model

figure, imagesc(abs(y_verify_S2 - E_2)); axis equal;axis off;colormap parula, title('Verify S2'); % All the elements should be canceled to be zero

clear x_coordinate;clear y_coordinate;clear t_coordinate;

%% Create the forward model of E_3

Size_y_S3 = N_y0 + D_shearing_y_S3; % Size of y after temporal shearing

Size_x_S3 = N_x0; % Size of x after temporal shearing

for i = 1:N_t

if V_S3 >= 0

shift_y_S3(i) = round((i-1).*V_S3); % The shearing distance of each frame along the downward direction

else

shift_y_S3(i) = D_shearing_y_S3+round((i-1).*V_S3); % The shearing distance of each frame along the upward direction

end

shift_x_S3(i) = 0; % The shearing distance of each frame in the x-direction

end

count = 1;

for i = 1:N_t % t

for k = 1:N_x0 % x

for j = 1:N_y0 % y

y_coordinate(count) = j + (k-1)*Size_y_S3 + shift_y_S3(i)+ ...

Size_y_S3*(shift_x_S3(i)); % Assign the y-coordinate according to the sensing operator

x_coordinate(count) = (i-1)*N_y0*N_x0 + (k-1)*N_y0 + j;

t_coordinate(count) = Full_vector(j + (k-1)*N_y0, i);

count = count + 1;

end

end

end

S_3=sparse(y_coordinate,x_coordinate,t_coordinate,...

Size_x_S3*Size_y_S3,N_y0*N_x0*N_t); % Generate the sensing operator at the shearing speed of S3

y_verify_S3 = Norm(reshape(S_3*Event(:),Size_y_S3,Size_x_S3)); % Create a measurement to verify the constructed forward model

figure, imagesc(abs(y_verify_S3 - E_3)); axis equal;axis off;colormap parula, title('Verify S3');% All the elements should be canceled to be zero

clear x_coordinate;clear y_coordinate;clear t_coordinate;

%% Create the forward model of E_4

Size_y_S4 = N_y0 + D_shearing_y_S4; % Size of y after temporal shearing

Size_x_S4 = N_x0; % Size of x after temporal shearing

for i = 1:N_t

if V_S4 >= 0

shift_y_S4(i) = round((i-1).*V_S4); % The shearing distance of each frame along the downward direction

else

shift_y_S4(i) = D_shearing_y_S4+round((i-1).*V_S4); % The shearing distance of each frame along the upward direction

end

shift_x_S4(i) = 0; % The shearing distance of each frame in the x-direction

end

count = 1;

for i = 1:N_t % t

for k = 1:N_x0 % x

for j = 1:N_y0 % y

y_coordinate(count) = j + (k-1)*Size_y_S4 + shift_y_S4(i)+ ...

Size_y_S4*(shift_x_S4(i)); % Assign the y-coordinate according to the sensing operator

x_coordinate(count) = (i-1)*N_y0*N_x0 + (k-1)*N_y0 + j;

t_coordinate(count) = Full_vector(j + (k-1)*N_y0, i);

count = count + 1;

end

end

end

S_4=sparse(y_coordinate,x_coordinate,t_coordinate,...

Size_x_S4*Size_y_S4,N_y0*N_x0*N_t); % Generate the sensing operator at the shearing speed of S4

y_verify_S4 = Norm(reshape(S_4*Event(:),Size_y_S4,Size_x_S4)); % Create a measurement to verify the constructed forward model

figure, imagesc(abs(y_verify_S4 - E_4)); axis equal;axis off;colormap parula, title('Verify S4'); % All the elements should be canceled to be zero

clear x_coordinate;clear y_coordinate;clear t_coordinate;

%% Create the forward model of E_5

Size_y_S5 = N_y0 + D_shearing_y_S5; % Size of y after temporal shearing

Size_x_S5 = N_x0; % Size of x after temporal shearing

for i = 1:N_t

if V_S5 >= 0

shift_y_S5(i) = round((i-1).*V_S5); % The shearing distance of each frame along the downward direction

else

shift_y_S5(i) = D_shearing_y_S5+round((i-1).*V_S5); % The shearing distance of each frame along the upward direction

end

shift_x_S5(i) = 0; % The shearing distance of each frame in the x-direction

end

count = 1;

for i = 1:N_t % t

for k = 1:N_x0 % x

for j = 1:N_y0 % y

y_coordinate(count) = j + (k-1)*Size_y_S5 + shift_y_S5(i)+ ...

Size_y_S5*(shift_x_S5(i)); % Assign the y-coordinate according to the sensing operator

x_coordinate(count) = (i-1)*N_y0*N_x0 + (k-1)*N_y0 + j;

t_coordinate(count) = Full_vector(j + (k-1)*N_y0, i);

count = count + 1;

end

end

end

S_5=sparse(y_coordinate,x_coordinate,t_coordinate,...

Size_x_S5*Size_y_S5,N_y0*N_x0*N_t); % Generate the sensing operator at the shearing speed of S5

y_verify_S5 = Norm(reshape(S_5*Event(:),Size_y_S5,Size_x_S5)); % Create a measurement to verify the constructed forward model

figure, imagesc(abs(y_verify_S5 - E_5)); axis equal;axis off;colormap parula, title('Verify S5'); % All the elements should be canceled to be zero

clear x_coordinate;clear y_coordinate;clear t_coordinate;

%% Create the forward model of E_6

Size_y_S6 = N_y0 + D_shearing_y_S6; % Size of y after temporal shearing

Size_x_S6 = N_x0; % Size of x after temporal shearing

for i = 1:N_t

if V_S6 >= 0

shift_y_S6(i) = round((i-1).*V_S6); % The shearing distance of each frame along the downward direction

else

shift_y_S6(i) = D_shearing_y_S6+round((i-1).*V_S6); % The shearing distance of each frame along the upward direction

end

shift_x_S6(i) = 0; % The shearing distance of each frame in the x-direction

end

count = 1;

for i = 1:N_t % t

for k = 1:N_x0 % x

for j = 1:N_y0 % y

y_coordinate(count) = j + (k-1)*Size_y_S6 + shift_y_S6(i)+ ...

Size_y_S6*(shift_x_S6(i)); % Assign the y-coordinate according to the sensing operator

x_coordinate(count) = (i-1)*N_y0*N_x0 + (k-1)*N_y0 + j;

t_coordinate(count) = Full_vector(j + (k-1)*N_y0, i);

count = count + 1;

end

end

end

S_6=sparse(y_coordinate,x_coordinate,t_coordinate,...

Size_x_S6*Size_y_S6,N_y0*N_x0*N_t); % Generate the sensing operator at the shearing speed of S6

y_verify_S6 = Norm(reshape(S_6*Event(:),Size_y_S6,Size_x_S6)); % Create a measurement to verify the constructed forward model

figure, imagesc(abs(y_verify_S6 - E_6)); axis equal;axis off;colormap parula, title('Verify S6'); % All the elements should be canceled to be zero

clear x_coordinate;clear y_coordinate;clear t_coordinate;

%% Create the forward model of the static image (E_0)

count = 1;

for i = 1:N_t

for k = 1:N_x0

for j = 1:N_y0

y_coordinate(count) = j + (k-1)*N_y0 + 0;

x_coordinate(count) = (i-1)*N_y0*N_x0 + (k-1)*N_y0 + j;

t_coordinate(count) = Full_vector(j + (k-1)*N_y0,i);

count = count + 1;

end

end

end

S_0=sparse(y_coordinate,x_coordinate,t_coordinate,N_x0*N_y0,N_y0*N_x0*N_t); % Generate the sensing operator of the static image

y_verify_static = Norm(reshape(S_0*Event(:),N_y0,N_x0)); % Create a measurement to verify the constructed forward model

figure, imagesc(abs(y_verify_static - E_0)); axis equal;axis off;colormap parula, title('Static'); % All the elements should be canceled to be zero

clear x_coordinate;clear y_coordinate;clear t_coordinate;

%% Create the sets of streak measurements (spatiotemporal projections) and sensing operators

E = [E_0(:);...

E_1(:);...

E_2(:);...

E_3(:);...

E_4(:);...

E_5(:);...

E_6(:);...

];

S = [S_0;...

S_1;...

S_2;...

S_3;...

S_4;...

S_5;...

S_6;...

];

%% Run TwIST

%%%% Note: the default maximum number of iterations is 1000. It can be set inside the function "TwIST.m"

close all;

%%%% TwIST parameters

initial_guess = S'*E;

lambda = 1e-4;

tau = 0.01; % Adjust the regularizer parameter

tolA = 1e-12;

tv_iters=2;

Psi = @(x,th) TTR_denoise_func(x,th,N_y0,N_x0,N_t,tv_iters); % Define the regularizer

Phi = @(x) TTR_TVphi(x,N_y0,N_x0,N_t);

[Im_cube,x_debias_twist,obj_twist,...

times_twist,debias_start_twist,mse]= ...

TwIST(E,S,tau, ...

'Lambda', lambda, ...

'Debias',0,...

'Monotone',1,...

'Sparse', 1,...

'Initialization',initial_guess(:),...

'Psi',Psi,...

'Phi',Phi,...

'StopCriterion',1,...

'ToleranceA',tolA,...

'Verbose', 1);

Im_cube = Norm(reshape(Im_cube,N_y0,N_x0,N_t)); % Output from the TwIST algorithm

%% Display the result

bg = mean(Im_cube(1:10, 1:10,:), 'all'); % Calculate the background of the result

figure, set(gcf,'color','k');

for i=1:size(Im_cube, 3)

im = (Im_cube(:,:,i));

subplot(1,2,1), imagesc(Event(:,:,i),[0 1]); axis off;axis image;colormap('gray'); title('Ground truth', 'Color', 'white'); %Display the ground truth

subplot(1,2,2), imagesc(im,[bg 1]); axis off;axis image;colormap('gray'); title('Reconstruction', 'Color', 'white') %Display the reconstructed datacube

pause(0.1);

end

S9 Commented Python code for implementing layer hologram calculation – Section 12 (Tomoyoshi Shimobaba)

# written by Tomoyoshi Shimobaba, Chiba University

# 1st Nov. 2023

import numpy as np

from skimage import io

from skimage.transform import resize

def angular(u, pitch, wl, z): #angular spectrum method

px=pitch[0]

py=pitch[1]

ext=np.zeros((u.shape[0] * 2, u.shape[1] * 2), np.complex64) #zero padding for avoiding wraparound nosie

posx=(ext.shape[1]- u.shape[1]) // 2

posy=(ext.shape[0]- u.shape[0]) // 2

ext[posy : posy + u.shape[0], posy:posy+u.shape[1]]=u

nx=ext.shape[1]

ny=ext.shape[0]

x = np.arange(-(nx//2), (nx+1)//2)

y = np.arange(-(ny//2), (ny+1)//2)

xx, yy = np.meshgrid(x, y)

dx=1.0/(nx*px)

dy=1.0/(ny*py)

trans = np.exp(1j * 2.0 * np.pi *z * np.sqrt((1.0/wl)**2 - (xx*dx)**2-(yy*dy)**2 ))

ext=np.fft.fftshift(ext)

ext=np.fft.fft2(ext)

ext=np.fft.fftshift(ext)

ext*=trans

ext=np.fft.fftshift(ext)

ext=np.fft.ifft2(ext)

ext=np.fft.fftshift(ext)

u = ext[posy : posy + u.shape[0], posy:posy+u.shape[1]]

return u

if __name__ == '__main__':

new_shape = (2048,2048) # hologram resolution

nz = 32 #number of layers

wl = 532e-9 #wavelength

z = 5e-2 #distance

dz = 3e-2 / nz

pitch=(3.74e-6, 3.74e-6) #sampling pitch

rgb=io.imread("table_rgb.png", as_gray=True) ## load RGB image

rgb=resize(rgb,new_shape, True)

rgb=np.sqrt(rgb)

# rgb=rgb.astype(np.complex64) # for random phase. If used, remove comment-out.

# rgb*=np.exp(1j*2.0*np.pi * np.random.rand(*new_shape)) # for random phase. If used, remove comment-out.

dep=io.imread("table_dep.png", as_gray=True) ## load depth image

dep=resize(dep,new_shape)

dep=dep / dep.max() * (nz - 1)

dep=dep.astype(np.uint8)

hol=np.zeros(new_shape, np.complex64)

for i in range(nz):

print(i)

tz=z+i*dz

extract_dep=np.where(dep==i, 255, 0)

extract_rgb=rgb*extract_dep*np.exp(-1j*2.0*np.pi/wl*tz) #compensation phase

# extract_rgb=rgb*extract_dep # for random phase. If used, remove comment-out.

hol += angular(extract_rgb, pitch, wl, tz)

io.imsave("hol.jpg", np.real(hol)) # save hologram

# reconstructions from the hologram

for i in range(nz):

print("reconst ", i)

tz=z+i*dz

reconst=angular(hol, pitch, wl, -tz)

io.imsave("reconst"+str(i)+".jpg", np.abs(reconst))

S10 MATLAB code of the HSPhR algorithm – Section 15 (Igor Shevkunov, Vladimir Katkovnik, and Karen Egiazarian)

%%%%%%%%%%%%%%%%%%%%%%%%%%%%%%%%%%%%%%%%%%%%%%%%%%%%%%%%%%%%%%%%%%%%%%%%

% Demo-code for the paper "Computational hyperspectral quantitative phase

% imaging from spectrally multiplexed observations",

% I. Shevkunov, V. Katkovnik, K.Eguazarian, 2023

%% %%%%%%%%%%%%%%%%%%%%%%%%%%%%%%%%%%%%%%%%%%%%%%%%%%%%%%%%%%%%%%%%%%%%%%%%

%

% Copyright (c) 2020-2023 Tampere University.

% All rights reserved.

% This work (software, material, and documentation) shall only

% be used for nonprofit noncommercial purposes.

% Any unauthorized use of this work for commercial or for-profit purposes

% is prohibited.

%

% AUTHORS:

% I. Shevkunov, V. Katkovnik, K.Eguazarian

% email: igor.shevkunov@tuni.fi

%

%%%%%%%%%%%%%%%%%%%%%%%%%%%%%%%%%%%%%%%%%%%%%%%%%%%%%%%%%%%%%%%%%%%%%%%%%%%%

clear

close all

addpath('.\AUX files')

fprintf ('\n Initialization started \n')

tic

%% ---------General parameters -------------

n1 = 64; % image size in dimension 1

n2 = 64; % image size in dimension 2

distance = 0.002; % propagation distance between object and sensor

dx = 3.45e-6; % pixel size of sensor

K = 6; % number of wavelengths

T = 18; % number of masks and experiments

nn = 150; % number of iterations

%% --------- Options for running reconstruction ------------------

do_CCF_filtering=1; % 1 - CCF filtering is on; 0 - off

do_Lagrange=1; % 1 - Lagrange variable are calculated; 0 - not

betta_CCF=0.3; % reconstruction parameter

%% Effects of Lagrange Multipliers and CCF: demonstration

it_start1=10; % iteration number to turn on Lagrange variables

it_start2=50; % iteration number to turn on CCF

%% ------------ Wavelength spectrum-----------------------

lambda1 = 550e-9; % The first wavelength of the range

lambda_end = 950e-9;% % the last wavelength of the range

delta_lambda = (lambda_end-lambda1)/(K-1);

lambda_set = lambda1:delta_lambda:lambda_end; % wavelengths set

%% ------------ Refractive index creation --------------------------------

glass_type=1; % 1 is for BK7 glass

n_ref = Refraction_Cauchy(lambda_set,glass_type); %refractive index according to Cauchy's equation

%% ------------ Noise parameters --------------------------------------------

do_poisson_noise = 0; % 1 - is for Poisson noise; 0 - Gaussian noise

sigma = 0.001; % standard deviation for Gaussian noise

gamma_gauss = 20*sigma^2; % reconstruction parameter

noise_type = 'Gaussian noise'; % prefix for figures

if do_poisson_noise

noise_type = 'Poisson noise'; % prefix for figures

kappa = 170000; % Poisson noise parameter

gamma_poisson = 6/kappa; % reconstruction parameter

end

%% Make Hyperspectral complex-valued object

%--------(1) upload images for amplitude and phase ------------------------

x_phase = double(imread('image_Cameraman64.bmp'))/255; % upload cameraman 64x64 image

x_ampl = double((imread('peppers_gray.png')))/255; % upload peppers image

x_ampl = x_ampl(211+(1:n1),246+(1:n2)); % crop 64x64 part from peppers

%--------(2) create phase shifting properties of the object w.r.t. wavelenghts and refractive index

s_lambda = 0;

for lambda = lambda_set

s_lambda = s_lambda+1;

varphi(:,:,s_lambda) = x_phase*lambda1*2*pi/lambda*(n_ref(s_lambda)-1); % lambda1 for scaling

fprintf ('.')

end

%--------(3) Make a Hyperspectral object ----------------------------------

x = x_ampl.*exp(1j*varphi);

%--------(4) Make zero-padding for angular spectrum proper propagation ---------------------

Nzp = ceil(max(lambda_set)*distance/(dx)^2); % zero-padding size

if rem(Nzp,2)~= 0;Nzp = Nzp+1;end % zero-padding size needed to be even

x = padarray(x,[Nzp/2 Nzp/2]);

object_coord_1=Nzp/2+(1:n1);

object_coord_2=Nzp/2+(1:n2);

%% Masks creation

Masks_set = zeros(Nzp+n1,Nzp+n2,K,T,'single'); % preallocation

s_lambda = 0;

for lambda = lambda_set

s_lambda = s_lambda+1;

rng(44)

for t = 1:T %% Masks depending on wavelength and refractive index

temp = randsrc(n1,n2,[0 pi/2 -pi/2 pi/4 -pi/4])/lambda*lambda1*(n_ref(s_lambda)-1);% lambda1 for scaling

temp = exp(1j*temp);

Masks_set(:,:,s_lambda,t) = padarray(temp,[round(Nzp/2) round(Nzp/2)],1); %used masks set

fprintf ('.')

end

end

%% Angular Spectrum (AS) Propagation operator

%--------- (1) AS Transfer Function ------------------------------

[LL,KK,~] = size(x);

k = single(-KK/2:KK/2 - 1);

l = single(-LL/2:LL/2 - 1);

[k,l] = meshgrid(k,l);

AS = zeros(size(x));

s_lambda = 0;

for lambda = lambda_set

s_lambda = s_lambda+1;

shift = 2*pi/lambda*distance;

U = 1 - lambda^2*((k/(dx*KK)).^2+(l/(dx*LL)).^2);

TFunc = exp(1i*2*pi/lambda*distance*sqrt(U)); TFunc(U<0) = 0;

AS(:,:,s_lambda) = (TFunc)*exp(-1j*shift); %% AS Trunsfer function

end

clear k l U

%--------- (2) hyperspectral propagation with masks included ----------------

A = @(wf,Masks) ifft2(arrayIshift(AS.*(arrayshift(fft2(conj(Masks).*wf))))); % forward propagation

At = @(wf, Masks) Masks.*ifft2(arrayIshift(conj(AS).*arrayshift(fft2(wf)))); % backward propagation

%% Observations model

%-------preallocations-----------------------------------------------------

B = zeros(LL,KK,K,T,'single');

BB = zeros(LL,KK,K,T,'single');

D = zeros(LL,KK,K,T,'single');

Y = zeros(LL,KK,T,'single');

Z = zeros(LL,KK,T,'single');

%-------observations creation ---------------------------------------------

for t = 1:T %create observations for each mask

Masks = (Masks_set(:,:,:,t));

B(:,:,:,t) = A(x,Masks); % HS wavefront propagated to sensor

Y(:,:,t) = sum((squeeze(abs(B(:,:,:,t)))).^2,3); % noiseless obesrvation summed along wavelengths

if do_poisson_noise % Poisson noise Intensity Observations

Z(:,:,t) = poissrnd(Y(:,:,t)*kappa);

SNR(t) = 10*log10(sum(kappa^2*Y(:,:,t).^2)/sum((Y(:,:,t)*kappa-Z(:,:,t)).^2));

else % Gaussian noise Intensity Observations

Z(:,:,t) = Y(:,:,t)+randn(size(squeeze(Y(:,:,t))))*sigma;

SNR(t) = 10*log10(sum(Y(:,:,t).^2)/sum((Y(:,:,t)-Z(:,:,t)).^2));

end

fprintf ('.')

end

%% Reconstruction Algorithm

%------------(1) Random Initialization ------------------------------------

x_phase = rand(size(x));

s_lambda = 0;

for lambda = lambda_set

s_lambda = s_lambda+1;

varphi_0(:,:,s_lambda) = x_phase(s_lambda)*2*pi/lambda*lambda1*(n_ref(s_lambda)-1);

end

xs = (rand(size(x))).*exp(1j*randn(size(x)));

%% ------------- figures section --------------------------------------------

fig1 = figure('Name','Relative errors','units','normalized','outerposition',...

[0.1 0.4 0.4 0.35],'color', 'w');

Logo=axes('position',[0, 0, .12,.12,]);[logo_im, ~]=imread('Tuni logo.png');image(logo_im); set(Logo,'handlevisibility','off','visible','off'),

lambda_to_show = ceil(K/2); % wavelength number to show wavefront images for ampl and phase

fig2 = figure('Name','Reconstructed amplitude and phase','units',...

'normalized','outerposition',[0.5 0.4 0.22 0.4],'color', 'w');

Logo=axes('position',[0, 0, .21,.105,]);[logo_im, ~]=imread('Tuni logo.png');image(logo_im); set(Logo,'handlevisibility','off','visible','off'),

subplot 221, imshow(abs(x(Nzp/2+(1:n1),Nzp/2+(1:n2),lambda_to_show)),[]), ...

title('True amplitude'), ...

c = colorbar;

c.Label.String = 'Amplitude, a.u.';

subplot 222, imshow(varphi(:,:,lambda_to_show),[]),

title('True phase'),

c = colorbar;

c.Label.String = 'Phase, rad';

%--------------------------------------------------------------------------

fprintf ('\n Initialization took %g seconds', toc)

fprintf ('\n ------------------------------ \n Start of reconstruction.')

E = zeros(size(Z),'single'); %preallocation

%% Main Iterations

for s = 1:nn

tic

%% ---------------------(2) forward propagation--------------------------

for t = 1:T

B(:,:,:,t) = A(xs,Masks_set(:,:,:,t)); % HS object propagated to the sensor

Y(:,:,t) = sum((abs(B(:,:,:,t))).^2,3);

if do_poisson_noise

E(:,:,t) = Z(:,:,t)- kappa*Y(:,:,t); % residual estimation

else

E(:,:,t) = Z(:,:,t)- Y(:,:,t); % residual estimation

end

end

Bold = B; % temporal variable for saving B for the next iteration

%

clear E_solution

%% ------------------(3) Update B by the proximal operators--------------

for t = 1:T

if do_poisson_noise % gives solutions for Poisson noise

E_solution(:,:,t) = Poisson_quadratic_solution(kappa,gamma_poisson,B(:,:,:,t),D(:,:,:,t),Z(:,:,t));

B(:,:,:,t) = (B(:,:,:,t)+D(:,:,:,t))./(1+kappa*gamma_poisson-gamma_poisson*Z(:,:,t)./(E_solution(:,:,t)+eps));

else % gives solutions for Gaussian noise

E_solution(:,:,t) = analysis_cardano_solutions(sigma,gamma_gauss,B(:,:,:,t),D(:,:,:,t),Z(:,:,t));

B(:,:,:,t) = (B(:,:,:,t)+D(:,:,:,t))./(2*gamma_gauss/sigma^2*E_solution(:,:,t)+1);

end

end

%% ---------------- (4) Update Lagrange variables------------------------

if do_Lagrange && s>=it_start1

D = D-1*(B-Bold);

end

%% ---------------- (5) Backward propagation ----------------------------

xst = zeros(size(B),'single');

for t = 1:T

xst(:,:,:,t) = At(B(:,:,:,t)-D(:,:,:,t), Masks_set(:,:,:,t));

end

xs = mean(xst,4);

%% ---------------- (6) Update of U_{o,k} by CCF filtering --------------

if do_CCF_filtering && s>=it_start2

[xs_ccf,~] = CCF(xs);

xs = (1-betta_CCF)*xs +betta_CCF*xs_ccf; % smooth injection of filtering results

end

%% ------------- Relative errors estimation ----------------------------

s_lambda = 0;

for lambda = lambda_set

s_lambda = s_lambda+1;

Relerrs(s_lambda,s) = norm(x(object_coord_1,object_coord_2,s_lambda)...

- exp(-1i*angle(trace(x(object_coord_2,object_coord_2,s_lambda)'*xs(object_coord_1,object_coord_2,s_lambda))))...

* xs(object_coord_1,object_coord_2,s_lambda), 'fro')/norm(x(object_coord_1,object_coord_2,s_lambda),'fro'); % rel. error

end

%% ------------ Draw figures ------------------------------------------

if (rem(s,10) == 0)||s == 2

figure(fig1);

semilogy(squeeze(Relerrs(:,:))','lineWidth',1), grid on %semilogy(1:numel(Relerrs),Relerrs)

xlabel('Iteration'), ylabel('Relative error '),% legend(num2str(lambda_set'*1e9),'NumColumns',2),... 'Orientation','horizontal'

title(['Relative errors vs. iteration count, T=',num2str(T),', K=',num2str(K) '. ' noise_type ', SNR=',num2str(mean(SNR),3) ' dB'])

set(gca,'FontSize',12)

lgnd = legend( num2str(lambda_set'*1e9,3) ,'NumColumns',1,'FontSize',8,'location','bestoutside'); % ,'Orientation','horizontal'

title(lgnd,'\lambda, nm');

figure(fig2);

subplot 223, imshow(abs(xs(Nzp/2+(1:n1),Nzp/2+(1:n2),lambda_to_show)),[]), ...

title('Reconstructed amplitude'), ...

c = colorbar;

c.Label.String = 'Amplitude, a.u.';

subplot 224, imshow(angle(xs(Nzp/2+(1:n1),Nzp/2+(1:n2),lambda_to_show)),[]),

title('Reconstructed phase'),

c = colorbar;

c.Label.String = 'Phase, rad';

sgt = sgtitle([noise_type ' SNR=' num2str(mean(SNR),3) ' dB, \lambda=' num2str(lambda_set(lambda_to_show)*1e9,3) ' nm, {\it ERROR_{rel}}=' num2str(Relerrs(lambda_to_show,s),2), ', iter=' num2str(s)]);

sgt.FontSize = 8;

drawnow

end

fprintf ('\n iteration %u, %2.1f sec., reler=%1.4f', s, toc, mean(squeeze(Relerrs(:,s))))

end

% figure(3), sliceViewer(abs(xs)), title('Reconstructed amplitude'),

% figure(4),sliceViewer(angle(xs)), title('Reconstructed phase')

fprintf ('\n End of reconstructions, mean relative error = %1.3g \n', mean(Relerrs(:,s)))

The AUX files can be downloaded from here –(<https://github.com/Shevkuno/HsPhR>)

S11 Affine transform-based twin-image suppression for in-line Lensless Digital Holographic Microscopy – Section 17 (Marcin J. Marzejon, Mikołaj Rogalski, Maciej Trusiak)

The code for Affine Transform-based twin-image suppression for in-line Lensless Digital Holographic Microscopy can be found on GitHub <https://github.com/MRogalski96/GS_AT>. The code consists of 4 MATLAB files and a folder *Data* with an exemplary data. The main file is *main_GSwithAT.m*. The employed algorithms – angular spectrum propagation, Gerchberg-Saxton phase-retrieval algorithm, and automatic, affine transform-based holograms XYZ alignment are *AS_propagate_p.m*, *GSalgorithm.m*, and *AutoAffineTransform.m*, respectively.

S12 A Regularized Auto-Encoder for the Reconstruction of Phase and Amplitude in Digital In-line Holography – Section 20 (R.V. Vinu, Gopakumar G, Ziyang Chen, and Jixiong Pu)

**Data:** recorded intensity distribution of the in-line hologram

$N_{x}\times N_{y}$ : Size of the hologram

$Z$ : Propagation distance in µm

$\lambda$ : Wavelength of light source in µm

$\Delta_{x}\times\Delta_{y}$: Pixel size of the detector

$H$ : Normalised Hologram

**Procedure**

Step 1:$FTPT\leftarrow exp\left[ ikz\sqrt{1-(\lambda f_{x})^{2}-(\lambda f_{y})^{2}} \right]$

Where$k=\frac{2\pi}{\lambda}$the wave number,$\lambda$the wavelength, z the propagation distance, and$f_{x}$,$f_{y}$are the spatial frequencies defined by$N_{x}$and$N_{y}$in unit intervals.

FTPT is the propagation transfer function that interacts the object field and create the scattered wave for the hologram. Here FTPT is represented as a two-channel image: the first channel holds the real part, and the second channel holds the imaginary part.

Step 2: $F_{H}\leftarrow IFFT2(FFT2(H)*FFTSHIFT(FTPT))$

$F_{H}$is the backpropagated complex-field distribution obtained from the intensity distribution of the recorded in-line hologram.

Step 3:$\hat{H}\leftarrow\text{NetworkModel }(F_{H},W)$

$\hat{H}$is going to be a two-channel image: the first channel$\hat{H}_{1}$holds the real part and the second channel$\hat{H}_{2}$holds the imaginary part.

Step 4: $\text{Loss }J\left( H,\hat{H} \right)=MSE\left( H,\hat{H} \right)+\alpha_{1}V\left( \hat{H} \right)$

Where$MSE$is the Mean Squared Error,$\alpha_{1}$is the regularisation constant$\left( 1e^{-7} \right)$, $V\left( \hat{H} \right)=\sum\left| \hat{H}_{x} \right|+\sum\left| \hat{H}_{y} \right|$is the total variation loss component with$\hat{H}_{x}\text{\& }\hat{H}_{y}$being the X directional and Y directional gradient of the model output$\hat{H}$.

Step 5: Update the model parameters$W$to minimize$J\left( H,\hat{H} \right)$.

$W=W-\alpha_{2}\frac{dJ}{dW}$

Choose ADAM could be the optimizer and the learning rate empirically found good is$\alpha_{2}=0.005$.

Step 6: Repeat steps 3 to 5 until convergence.

Step 7: Output Amplitude and Phase at each grid position of the two-channel image $\hat{H}$.

$Amplitude=\sqrt{{\hat{H}^{2}}_{1}+{\hat{H}^{2}}_{2}}\text{ and }Phase=tan^{-1}\left( \frac{\hat{H}_{2}}{\hat{H}_{1}} \right)$

**Network Model:**

class Net(nn.Module):

def __init__(self):

super(Net, self).__init__()

**#Parallel Encoder 1**

self.conv_init_Eb1 = nn.Sequential(

nn.Conv2d(2, 16, 3, stride=2, padding=1),

nn.ReLU(True),

nn.BatchNorm2d(16),

nn.Conv2d(16, 16, 3, stride=1, padding=1),

nn.ReLU(True),

nn.BatchNorm2d(16),

)

self.conv_1Eb1 = nn.Sequential(

nn.Conv2d(16, 64, 3, stride=2, padding=1),

nn.ReLU(True),

nn.BatchNorm2d(64),

nn.Conv2d(64, 64, 3, stride=1, padding=1),

nn.ReLU(True),

nn.BatchNorm2d(64),

)

self.conv_2Eb1 = nn.Sequential(

nn.Conv2d(64, 128, 3, stride=2, padding=1),

nn.ReLU(True),

nn.BatchNorm2d(128),

nn.Conv2d(128, 128, 3, stride=1, padding=1),

nn.ReLU(True),

nn.BatchNorm2d(128),

)

**#Parallel Encoder 2**

self.conv_nonlinear_Eb1 = nn.Sequential(

nn.Conv2d(128, 512, 3, stride=1, padding=1),

nn.ReLU(True),

nn.BatchNorm2d(512),

nn.Conv2d(512, 16, 3, stride=1, padding=1),

nn.Tanh(),

)

self.conv_init_Eb2 = nn.Sequential(

nn.Conv2d(2, 16, 3, stride=2, padding=1),

nn.ReLU(True),

nn.BatchNorm2d(16),

nn.Conv2d(16, 16, 3, stride=1, padding=1),

nn.ReLU(True),

nn.BatchNorm2d(16),

)

self.conv_1Eb2 = nn.Sequential(

nn.Conv2d(16, 64, 3, stride=2, padding=1),

nn.ReLU(True),

nn.BatchNorm2d(64),

nn.Conv2d(64, 64, 3, stride=1, padding=1),

nn.ReLU(True),

nn.BatchNorm2d(64),

)

self.conv_2Eb2 = nn.Sequential(

nn.Conv2d(64, 128, 3, stride=2, padding=1),

nn.ReLU(True),

nn.BatchNorm2d(128),

nn.Conv2d(128, 128, 3, stride=1, padding=1),

nn.ReLU(True),

nn.BatchNorm2d(128),

)

self.conv_nonlinear_Eb2 = nn.Sequential(

nn.Conv2d(128, 512, 3, stride=1, padding=1),

nn.ReLU(True),

nn.BatchNorm2d(512),

nn.Conv2d(512, 16, 3, stride=1, padding=1),

nn.Tanh(),

)

**#Decoder**

self.deconv_1Db = nn.Sequential(

nn.ConvTranspose2d(32, 512, 3, stride=1, padding=1),

nn.ReLU(True),

nn.BatchNorm2d(512),

nn.ConvTranspose2d(512, 128, 3, stride=1, padding=1),

nn.ReLU(True),

nn.BatchNorm2d(128),

)

self.deconv_2Db = nn.Sequential(

nn.ConvTranspose2d(128, 128, 3, stride=1, padding=1),

nn.ReLU(True),

nn.BatchNorm2d(128),

nn.ConvTranspose2d(128, 64, 3, stride=2, padding=1, output_padding = 1),

nn.ReLU(True),

nn.BatchNorm2d(64),

)

self.deconv_3Db = nn.Sequential(

nn.ConvTranspose2d(64, 64, 3, stride=1, padding=1),

nn.ReLU(True),

nn.BatchNorm2d(64),

nn.ConvTranspose2d(64, 16, 3, stride=2, padding=1, output_padding = 1),

nn.ReLU(True),

nn.BatchNorm2d(16),

)

self.deconv_4Db = nn.Sequential(

nn.ConvTranspose2d(16, 16, 3, stride=1, padding=1),

nn.ReLU(True),

nn.BatchNorm2d(16),

nn.ConvTranspose2d(16, 16, 3, stride=2, padding=1, output_padding = 1),

nn.ReLU(True),

nn.Conv2d(16, 2, 3, stride=1, padding=1),

)

def forward(self, x):

x = x.float()

%Get output from Parallel Encoder 1

Enc1 = self.conv_init(x)

Enc1 = self.conv_1(Enc1)

Enc1 = self.conv_2(Enc1)

Enc1 = self.conv_nonlinear(Enc1)

%Get output from Parallel Encoder 2

Enc2 = self.conv_init_p(x)

Enc2 = self.conv_1_p(Enc2)

Enc2 = self.conv_2_p(Enc2)

Enc2 = self.conv_nonlinear_p(Enc2)

%Decode from the concatenated encoded data

Decx = self.deconv_1(torch.cat((Enc1, Enc2), 1))

Decx = self.deconv_2(Decx)

Decx = self.deconv_3(Decx)

Decx = self.deconv_4(Decx)

return Decx

S13 MATLAB code for customizing the Angular Memory Effect for Scattering Media – Section 22 (Sarp Feykun Şener, Mert Ercan and Hasan Yılmaz)

function [Q, T2, C] = encode_ME(T, MM, rt, rt_range, plot_corr)

%encode_ME calculates the memory operator whose eigenvectors give high correlations at encoded

% translation value by using the transmission matrix of a fiber.

% Based on the work done in H. Yılmaz et al., "Customizing the Angular Memory Effect for Scattering Media".

%

% [Q, T2, C] = encode_ME(T, MM, rt, rt_range, plot_corr)

% outputs:

% Q is the operator whose eigenvectors are memory-encoded

% T2 is the translated transmission matrix in the given translation values for the input and the output

% C is the correlation array

%

% inputs:

% T is the transmission matrix of the fiber with the size N x N (if you are not working with fibers T can be replaced by a Gaussian random matrix or any matrix of your choice)

% MM is the mode matrix of the fiber with the size [N x (mode number)] (if you are not working with fibers MM can be replaced by the identity matrix)

% rt is the radial translation value, which is used to encode the memory

% rt_range is the translation range array to be used for plotting.

% plot_corr is a logic value to indicate whether the function will be plot

% the result

%

% 2023 Sarp Feykun Şener

% ECOS LAB at Bilkent University, UNAM

N = size(T,1);

T1 = T;

T1M = MM'*T*MM;

% DEFINING T2 (multiple shift values)

for j = 1:rt_range(end)+1

%shift input

for i = 1:N

sprintf("T2 is being calculated.. shift input at shift:%.f, %.f (%.f, %.f)",[j,rt_range(end)+1,i,N])

I1 = reshape(T1(i,:),[sqrt(N) sqrt(N)]);

I_inputShift = circshift(I1, [rt_range(j) 0]); % here vertical shift is defined only in -y

TM_inputShift(i,:) = I_inputShift(:);

end

%shift output

for i = 1:N

sprintf("T2 is being calculated.. shift output at shift:%.f, %.f (%.f, %.f)",[j,rt_range(end)+1,i,N])

I2 = reshape(TM_inputShift(:,i),[sqrt(N) sqrt(N)]);

I_outputShift = circshift(I2, [rt_range(j) 0]); % here vertical shift is defined only in -y

T2(:,i,j) = I_outputShift(:);

end

T2M(:,:,j) = MM'*T2(:,:,j)*MM;

end

% DEFINING THE OPERATOR:

Q0 = inv(T1M'*T1M)*T1M'*T2M(:,:,rt); %operator is defined in mode basis, one can assign identity to MM matrix to work in real space

param = 0;

Q = (Q0*exp(1i*param)+Q0')/2;

[V,a] = eig(Q); %the egienvalues and eigen vectors of the operator

if plot_corr == 1

%Below (three) lines shows how to define a random input in mode basis

m = size(MM,2); %number of modes

R0 = randn(m,1) + 1i*randn(m,1);

R = R0/sqrt(R0'*R0); %define random coefficients (random input) for mode contributions

v = 130; %chose an arbitrary eigenstate to observe the output correlation

U1 = T1M*V(:,v); %propagated eigenstate by the unshifted T1 transmission matrix

U1R = T1M*R; %propagated random input by the unshifted T1 transmission matrix

C = 0;C_R=0;

for j = 1:rt_range(end)+1

U2 = T2M(:,:,j)*V(:,v); %propagated eigenstate by the shifted T2 transmission matrix

C_num = abs(U1'*U2)^2;

C_denom = (U1'*U1)*(U2'*U2);

C(j) = sqrt(C_num./C_denom);

U2R = T2M(:,:,j)*R; %propagated rando input by the shifted T2 transmission matrix

C_numR = abs(U1R'*U2R)^2;

C_denomR = (U1R'*U1R)*(U2R'*U2R);

C_R(j) = sqrt(C_numR./C_denomR);

end

lineWidth = 2; % Line thickness for plots

gridAlpha = 0.15; % Grid line transparency (0 is fully transparent, 1 is opaque)

fontSize = 25; % Font size for labels, title, and legends

fontName = 'Times'; % Font name

figure;

set(gcf, 'Units', 'Normalized', 'OuterPosition', [0, 0.04, 1, 0.8]);

set(0,'defaultTextInterpreter','latex');

plot(rt_range, C, 'r-o', 'MarkerFaceColor', 'r', 'markersize', 10, 'linewidth', lineWidth); % Filled circles

xlabel('radial translation', 'FontSize', fontSize-7, 'FontName', fontName); % x-axis in terms of lambda

ylabel('correlation $C_n$ ', 'FontSize', fontSize-7, 'FontName', fontName); % x-axis in terms of lambda

yticks([0,0.5,1])

set(gca, 'GridAlpha', gridAlpha); % Set grid line transparency

hold on;

plot(rt_range, C_R, 'k-o', 'MarkerFaceColor', 'k', 'markersize', 10, 'linewidth', lineWidth); % Filled circles

hold off;

set(gca,'FontSize', fontSize, 'FontName', fontName);

ylim([0 1]);

xlim([0 rt_range(end)]);

leg = legend("eigenvector input","random input","Location","northeast");

set(leg, 'FontName', fontName, 'FontSize', fontSize - 10, 'EdgeColor', 'none');

end

end

S14 Computational Diffuse Imaging Using Artificial Intelligence (*Ganesh M. Balasubramaniam, Gokul Manavalan, and Shlomi Arnon*)

S14.1 Introduction

This supplementary document provides a comprehensive, step-by-step explanation of the MATLAB code utilized to generate a simulated dataset through Diffuse Optical Tomography (DOT) forward measurements using Toast++ [S14.1].

Toast++ is an advanced software suite that simulates light propagation through diffuse media using the finite element method (FEM). The utilization of deterministic FEM simulations for light transport simulation emerges as an optimal approach for the construction of datasets tailored to deep learning applications DOT [S14.2]. Apart from the non-availability of large experimental datasets, this preference arises from the compelling capabilities of FEM-based software like Toast++ to accurately model complex light propagation phenomena, offering a foundation for data that reflects the intricacies of real-world optical interactions, ensuring that the resultant datasets provide the most reliable and informative simulated inputs for training deep learning models to solve the inverse problems using artificial intelligence (AI) in DOT.

In the following sections, we provide a comprehensive breakdown of the dataset creation process, presenting a detailed account of meshing, simulations, and the requisite parameters to be preserved for dataset generation. Enclosed MATLAB code snippets complement the explanations [S14.3].

1. Mesh Creation and Loading the Breast Mesh

Digibreast mesh is used to create the dataset in this document. DigiBreast [S14.4] is a numerical breast phantom and is a versatile resource for simulating various imaging modalities. This mesh-based code enables loading 3D breast shapes and anatomical structures for multi-physics simulations and validation of image reconstruction algorithms. The following code adds specific directories to the MATLAB path, allowing access to relevant functions and data. The paths are added for the 'DigiBreast' and 'iso2mesh' libraries, which are required for subsequent operations. It then loads data from a file named *'DigiBreast.mat*,' which is located in the '*DigiBreast*' directory. This dataset includes information about a forward mesh ('Digimesh') and optical properties ('*OptiProp*'). The code further assigns values to various properties related to different fatty tissue segmentations, identified by numeric labels: *truth*, *thresholdp2*, *dualgaussian*, *threshold2*, and *empirical*. The DigiBreast digital breast phantom can be downloaded from references [S14.4, S14.5].

*%% loading the Mesh and Add paths to the Folder*

*%The DIgiBreast numerical breast phantom can be downloaded from:* [*%http://fanglab.org/software/DigiBreast.html*](http://fanglab.org/software/DigiBreast.html)

*addpath(genpath('___path___\DigiBreast'))*

*addpath(genpath('___path___\iso2mesh-master'))*

*DigiBreastfolder = '___path___\DigiBreast\data\DigiBreast.mat';*

*DigiBreast = load(DigiBreastfolder);*

*Digimesh = DigiBreast.ForwardMesh; % DigiBreast Forward mesh*

*OptiProp = cell2mat(DigiBreast.OpticalProperties(2:end,2:end));*

*Digimesh.value=[Digimesh.glandularity.truth Digimesh.glandularity.thresholdp2 Digimesh.glandularity.dualgaussian...*

*Digimesh.glandularity.threshold2 Digimesh.glandularity.empirical]; % different fatty tissues segmentations*

*% truth=1; thresholdp2=2; dualgaussian = 3; threshold2=4; empirical=5;*

S14.2 Source Detector Configuration

In this dedicated section, we delve into establishing the tomographic geometry tailored explicitly for the precise imaging of breast tissues. Configuring a well-defined geometric setup is paramount to capture and interpret the optical properties within the breast effectively. It starts by converting a finite element mesh ('Digimesh' (see code in the previous section)) into a format suitable for the 'Toast++' software, where mesh nodes and elements are extracted and converted. The grid basis for the simulation is defined with specified dimensions (*bx, by, bz*), and a linear interpolation method is chosen. We then create detection and source points ('*Detec*' and '*D*') to simulate light propagation through breast tissue. These points are distributed along the x, y, and z coordinates, covering a range within the breast mesh. The code then specifies the simulation type, which can be either "*Transmission*" or "*Reflection*," and generates source and detector positions accordingly based on the chosen type [S14.6, S14.7]. Finally, it sets up the mesh for the simulation, defines source and detector properties, and computes the Q-vector and M-vector used in the simulation.

*% Convert FEM mesh to toast mesh.*

*% Import the vertices, nodes and element types from the “digibreast” mesh and assign them to the toast mesh to %form a numerical phantom*

*vtx = Digimesh.node; idx = Digimesh.elem; eltp = ones(length(idx),1).*3;*

*toast_mesh = toastMesh(vtx,idx,eltp);*

*nodes = toast_mesh.Data;*

*refine = false; % boolean - refining the mesh for the tumor area (true if refinement is needed, false if %refinement is unnecessary)*

*segtype = empirical; % chosen segmentatin for forward calculation (see previous section to see the different %types of segmentation). The “segtype” allows us to determine the distribution of optical properties in the %numerical breast phantom.*

*refrec = 1.34; % refractive index.*

*% Define the dimensions of the numerical phantom in terms of number of voxels (or tetrahedrons)*

*bx = 256;*

*by = 128;*

*bz = 64;*

*% Convert the discrete mesh into a continuous medium called “basis.”*

*basis = toastBasis(toast_mesh,[bx by bz],'Linear'); n = toast_mesh.NodeCount();*

*% Determine the array of sources and detectors by selecting points on the opposite boundaries of the numerical %breast phantom. The lines below try to choose only the points that are on the boundaries and within the %dimensions of the numerical phantom. This arrangement can be changed for any configuration based on the %user.*

*az = 90; el = 90; view(az,el) delX = 20; delY = 4;*

*j = 1;*

*Detec = [];*

*D = [];*

*for x = min(Digimesh.node(:,1)):delX:max(Digimesh.node(:,1))%[188]*

*if x==105*

*a=1;*

*end*

*relevant_y = nodes((nodes(:,1)>x-1 & nodes(:,1)<x+1),:);*

*miny = -10; maxy = max(relevant_y(:,2));*

*minz = min(relevant_y(:,3)); maxz = 30;*

*ys = miny:20:maxy;*

*for k = 1: length(ys)*

*Detec(j,:) = [x,ys(k),maxz];*

*D(j,:) = [x,ys(k),minz];*

*j = j+1;*

*end*

*end*

*% Select the type of geometry to arrange the S-D array. Two types can be chosen: reflection (S-D on same side) %or transmission (S-D on opposite sides).*

*simulation_type = "Transmission"*

*r_z= 29.75;% size of breast to arrange the S-D configuration (z-dir)*

*r_x = 100;% size of breast to arrange the S-D configuration (x-dir)*

*r_y = 0;*

*if simulation_type == "Transmission"*

*[Sources, Detectors] = CreateTransmissionSDla(r_x, r_y, r_z);*

*elseif simulation_type == "Reflection"*

*[Sources, Detectors] = CreateReflectionSDla(r_x, r_y, r_z);*

*else*

*[Sources, Detectors] = CreateBothSDla(r_x, r_y, r_z);*

*end*

*% Arrange the sources and detectors by specifying the size and the type of light source that can be used.*

*toast_mesh.SetQM(Sources,Detec);*

*qvec = toast_mesh.Qvec('Neumann','Gaussian',2);*

*mvec = toast_mesh.Mvec('Gaussian',4,0);*

*qvec = full(qvec);*

*mvec = full(mvec);*

1. Optical Properties and Tumor Assignment

In this section, we delve into assigning optical properties to the DigiBreast mesh, a crucial step in our investigation. Additionally, we explore the incorporation of tumors at diverse locations within the phantom. The code segment begins by selecting a random segmentation type ('*segtype*') and preparing a mesh structure using '*Digimesh*' data. The mesh is further modified with added noise and Gaussian filtering. The code then selects random coordinates for a tumor within the breast, ensuring that the tumor remains within the breast boundaries. The tumor's shape and position are defined by '*centroid*' and '*fwhmsize*,' respectively. If '*refine*' is true, the mesh is refined in the tumor region to account for the tumor's shape. The optical properties of the breast tissue are defined based on a weighted combination of absorption and scattering properties, taking into account the tumor's presence and contrast level [S14.2, S14.6, S14.8-S14.10].

*% Insert small spheres within the Digibreast that are different in optical properties from the background mesh.*

*% Sample points until good centroid and radius are achieved (not outside of the breast*

*segtype = randi([1 5],1,1);%empirical;*

*mesh.node = Digimesh.node;*

*mesh.elem = Digimesh.elem;*

*mesh.value = Digimesh.value(:,segtype);*

*value = awgn(mesh.value,4,'measured'); value = reshape(basis.Map('M->B', value),bx,by,bz);*

*value = imgaussfilt(value,0.5); value(value<0)=0;*

*mesh.value = basis.Map('B->M', value);*

*rt = rand(1,1)*13+2; % Create meshes of different sizes to bring variety to the dataset*

*xmin = min(mesh.node(:,1))+1.5*rt; xmax = max(mesh.node(:,1))-1.5*rt;*

*xt = (rand(1,1)*(xmax-xmin) + xmin); % r_t:dim-r_t mm*

*armed = true; % bool - true:still bad params, false:good params*

*wh_idx = 1; % limit to number of tries*

*contin = false;*

*while armed*

*relevant_y = nodes((nodes(:,1)>xt-1.5 & nodes(:,1)<xt+1.5),:);*

*ymin = -15+rt; ymax = max(relevant_y(:,2))-rt;*

*yt = (rand(1,1)*(ymax-ymin) + ymin); % r_t:dim-r_t mm*

*relevant_x = nodes((nodes(:,2)>yt-3 & nodes(:,2)<yt+3),:);*

*xmin = min(relevant_x(:,1)); xmax = max(relevant_x(:,1));*

*if xt-1.0*rt>xmin && xt+1.0*rt<xmax*

*armed = false;*

*end*

*if wh_idx > 10*

*armed = false*

*contin = true;*

*wh_idx = 1;*

*end*

*wh_idx = wh_idx+1;*

*end*

*if contin*

*continue*

*end*

*relevant_z = nodes((nodes(:,1)>xt-4 & nodes(:,1)<xt+4 & nodes(:,2)>yt-4 & nodes(:,2)<yt+4),:);*

*zmin = min(relevant_z(:,3))+1.2*rt; zmax = max(relevant_z(:,3))-1.2*rt;*

*zt = (rand(1,1)*(zmax-zmin) + zmin); % r_t:depth-r_t mm*

*% centroid = [xt yt zt];*

*centroid = [182.31 27.18 10.909];*

*% fwhmsize=rt;*

*fwhmsize = 7.95;*

*%If 'refine' is true, the mesh is refined in the tumor region to account for the tumor's shape. The optical %properties of the breast tissue are defined based on a weighted combination of absorption and scattering %properties, taking into account the tumor's presence and contrast level*

*% refine the mesh in the tumor region (continue if error occurs)*

*if refine*

*try*

*refinment = 0.0001934*fwhmsize^2 + 0.01124*fwhmsize -0.01388;*

*ref_mesh=digibreast_meshrefine(mesh,centroid,fwhmsize,refinment);*

*catch*

*continue*

*end*

*vtx = ref_mesh.node; idx = ref_mesh.elem; eltp = ones(length(idx),1).*3;*

*refrec = 1.34;*

*toast_mesh = toastMesh(vtx,idx,eltp); % create the mesh object*

*toast_mesh.SetQM(Q,D);*

*qvec = full(qvec);*

*mvec = full(mvec);*

*else*

*ref_mesh=mesh;*

*end*

*% Insert the tumors of random sizes at random locations for each iteration. The “digibreast_lesionprofile” %contains all the properties of the tumors like shape, size and location.*

*lesionprofile=digibreast_lesionprofile(ref_mesh.node,centroid,fwhmsize);*

*% Define the optical properties of the lesion (tumor). The references of the optical properties can be found is %the text of section S4.*

*wgt_f = ref_mesh.value.*(1-lesionprofile);*

*wgt_a = (1-ref_mesh.value).*(1-lesionprofile);*

*wgt_l = lesionprofile;*

*contrast = 0.5; %the difference in the optical properties between the lesion and the background.*

*mua = ones(size(lesionprofile)).*(mean(wgt_a)*OptiProp(1,9) + mean(wgt_f)*OptiProp(2,9)); % absorption %coefficient*

*mus = ones(size(lesionprofile)).*(mean(wgt_a)*OptiProp(1,8) + mean(wgt_f)*OptiProp(2,8)); % scattering %coefficient*

*ref = ones(size(mua)).*refrec; % refractive index*

S14.3 Light transport simulations and dataset creation

The following code segment is pivotal in conducting forward measurements and storing essential data for dataset creation. It commences by initializing variables to simulate measurements, computing system matrices, and determining the photon density field [S14.2, S14.11]. The logarithm of this field is then computed and thoughtfully saved in both CSV ('*logY_filename'*) and TIFF image ('*logY_filename1*') formats, facilitating subsequent in-depth analysis and visualization. Furthermore, the code introduces a configuration parameter set ('*cfg*') that encompasses mesh specifications, segmentation characteristics, and optical properties. The absorption properties ('*mua*') are calculated using a weighted combination of various tissue components, with these values meticulously stored in both CSV ('*mua_filename'*) and MAT ('*mua_filename1'*) files.

Additionally, the tumor's radius ('*radius*') is preserved in a CSV file ('*rad_filename*'), and the precise coordinates of the tumor ('*centroid*') are recorded in another CSV file ('*coord_filename*'). This comprehensive dataset is the foundation for solving the Inverse Problem using AI.

*% Begin the light propagation simulations (forward problem)*

*qvec = toast_mesh.Qvec('Neumann','Gaussian',2);*

*mvec = toast_mesh.Mvec('Gaussian',4,0);*

*qvec = full(qvec);*

*mvec = full(mvec);*

*K = dotSysmat(toast_mesh, mua, mus, ref, 0);*

*Phi = K \ qvec;*

*Y = mvec.' * Phi;*

*logY = log(Y);*

*logY_real = real(logY);*

*%Save the measurement matrix (logY_* *filename). This measurement matrix will be the input to any neural %network or machine learning algorithm. The matrix can be saved as an image or as a matrix. Save and work %with matrices to preserve data and not lose a lot of information. However, saving it as an image also works.*

*logY_**filename = convertStringsToChars("___path___/Input_csv/inp" + num2str(i) + ".csv");*

*csvwrite(logY_filename, logY_real.');*

*logY_filename1 = strcat("___path___/Input_image/inp" + num2str(i) + '.tif');*

*imwrite(mat2gray(logY_real.'), logY_filename1);*

*imagesc(logY_real.');*

*% create and save a reference mesh without the tumors and add optical properties. The optical properties of the %DigiBreast numerical phantom is inbuilt in the DigiBreast folder. However, they can also be provided %manually depending on the user.*

*% “cfg” contains all the properties of the mesh and the tumors including the optical properties, location, size %and background parameters.*

*cfg.mesh = ref_mesh;*

*% cfg.refinment = refinment;*

*cfg.centroid = centroid;*

*cfg.radius = fwhmsize;*

*cfg.delX = delX;*

*cfg.delY = delY;*

*cfg.segtype = segtype;*

*cfg.lesionprofile=digibreast_lesionprofile(mesh.node,cfg.centroid,cfg.radius);*

*cfg.wgt_f = mesh.value.*(1-cfg.lesionprofile);*

*cfg.wgt_a = (1-mesh.value).*(1-cfg.lesionprofile);*

*cfg.wgt_l = cfg.lesionprofile;*

*cfg.contrast = contrast;*

*cfg.mua = (cfg.wgt_a.*0.444 + cfg.wgt_f.*0.407 + cfg.wgt_l.*0.72+(0.418-0.358)*cfg.contrast);*

*cfg.mus = (cfg.wgt_a.*OptiProp(1,8) + cfg.wgt_f.*OptiProp(2,8) + cfg.wgt_l.*(OptiProp(1,8)+(OptiProp(3,8)-OptiProp(1,8))*cfg.contrast));*

*mua_basis = reshape(basis.Map('M->B', cfg.mua),bx,by,bz);*

*cfg.refrec = refrec;*

*% Save the absorption coefficient distribution (in case the end goal is to use 1D, 2D or 3D deep neural %networks). If shallow machine learning techniques are used and if the background mesh contains %homogeneous optical property distribution, the tumors can be detected by simply knowing their optical %properties, location and size. Therefore, these parameters can also be saved (very simplified form of DOT where one can reconstruct the breast mesh manually after detecting the tumors).*

*%The parameters that have to be saved depends on the user of the code and the type of AI method used to perform, 3D imaging, sensing or even segmentation.*

*mua_filename = convertStringsToChars("___path___/Ground_Truth_vectorform/label" + num2str(i) + ".csv");*

*csvwrite(mua_filename, cfg.mua);*

*mua_filename1 = convertStringsToChars("___path___/Ground_Truth_3D/label" + num2str(i) + ".mat");*

*save(mua_filename1, 'mua_basis');*

*rad_filename = convertStringsToChars___path___/Ground_Truth_radius/label" + num2str(i) + ".csv");*

*csvwrite(rad_filename, cfg.radius);*

*coord_filename = convertStringsToChars("___path___/Ground_Truth_coordinates/label" + num2str(i) + ".csv");*

*csvwrite(coord_filename, cfg.centroid);*

1. Summary and conclusions

The dataset generated through the code explained in the preceding sections is a valuable resource for training diverse AI algorithms. These algorithms, once trained, can play a pivotal role in addressing the intricate inverse problem of diffuse optical tomography (DOT) and effectively detecting tumors within compressed breast tissues. Leveraging this dataset empowers AI-driven solutions to make significant strides in the field of medical imaging, offering the potential for more accurate and efficient tumor detection in this critical clinical context [S14.2, S14.10, S14.12-S14.14].

In conclusion, the systematic description and implementation of mesh generation, source, and detector configuration, as well as the assignment of optical properties and the inclusion of tumors within the digital phantom (DigiBreast phantom in our case), represent essential facets of any research methodology involving AI-based DOT. The meticulous steps and accompanying codes detailed in the previous sections collectively lay the groundwork for the comprehensive acquisition of data and the subsequent analysis of breast tissues and tumor characteristics. These meticulous procedures are instrumental in advancing our understanding and diagnostic capabilities in optical medical imaging, offering valuable insights that contribute to improved healthcare and diagnostic applications.

References

S. 14.1 M. Schweiger and S. Arridge, "The Toast++ software suite for forward and inverse modeling in optical tomography," J. Biomed. Opt. **19**, 040801 (2014).

S. 14.2 G. M. Balasubramaniam, B. Wiesel, N. Biton, R. Kumar, J. Kupferman, and S. Arnon, "Tutorial on the Use of Deep Learning in Diffuse Optical Tomography," Electron. **11**, (2022).

S. 14.3 A. Hauptman, G. M. Balasubramaniam, and S. Arnon, "Machine Learning Diffuse Optical Tomography Using Extreme Gradient Boosting and Genetic Programming," Bioengineering **10**, 382 (2023).

S.14.4 B. Deng, D. H. Brooks, D. A. Boas, M. Lundqvist, and Q. Fang, "Characterization of structural-prior guided optical tomography using realistic breast models derived from dual-energy x-ray mammography," Biomed. Opt. Express **6**, 2366 (2015).

S.14.5 Q. Fang and D. A. Boas, "Monte Carlo Simulation of Photon Migration in 3D Turbid Media Accelerated by Graphics Processing Units," Opt. Express **17**, 20178 (2009).

S.14.6 B. W. Pogue, T. O. McBride, U. L. Osterberg, and K. D. Paulsen, "Comparison of imaging geometries for diffuse optical tomography of tissue," Opt. Express **4**, 270 (1999).

S.14.7 H. Ben Yedder, M. Shokoufi, B. Cardoen, F. Golnaraghi, and G. Hamarneh, "Limited-angle diffuse optical tomography image reconstruction using deep learning," in *Lecture Notes in Computer Science (Including Subseries Lecture Notes in Artificial Intelligence and Lecture Notes in Bioinformatics)* (2019), Vol. 11764 LNCS, pp. 66–74.

S.14.8 J. M. Schmitt and G. Kumar, "Optical scattering properties of soft tissue: a discrete particle model," Appl. Opt. **37**, 2788 (1998).

S.14.9 S. L. Jacques, "Optical properties of biological tissues: A review," Phys. Med. Biol. **58**, (2013).

S.14.10 A. Hauptman, G. M. Balasubramaniam, and S. Arnon, "Machine Learning Diffuse Optical Tomography Using Extreme Gradient Boosting and Genetic Programming," Bioengineering **10**, 382 (2023).

S.14.11 G. M. Balasubramaniam, N. Biton, and S. Arnon, "Imaging through diffuse media using multi-mode vortex beams and deep learning," Sci. Rep. **12**, 1561 (2022).

S.14.12 G. M. Balasubramaniam and S. Arnon, "Deep-learning algorithm to detect anomalies in compressed breast: A numerical study," in *Optics InfoBase Conference Papers* (OSA Technical Digest (Optical Society of America, 2021), 2021), p. paper DTu3A.5.

S.14.13 G. M. Balasubramaniam, G. Manavalan, A. Hauptman, and S. Arnon, "Infant head subsurface imaging using high-density diffuse optical tomography and machine learning," in *Diffuse Optical Spectroscopy and Imaging IX*, D. Contini, Y. Hoshi, and T. D. O’Sullivan, eds. (SPIE, 2023), p. 29.

S.14.14 G. M. Balasubramaniam, G. Manavalan, A. S. Kadosh, and S. Arnon, "Breast tumor detection using regularized deep-learning diffuse optical tomography," in *Diffuse Optical Spectroscopy and Imaging IX*, D. Contini, Y. Hoshi, and T. D. O’Sullivan, eds. (SPIE, 2023), p. 92.

S15 Computational imaging with post-processing of the randomness (Manisha, Tanushree Karmakar, Aditya Chandra Mandal And Rakesh Kumar Singh) – Section 25

Codes of random illuminations for recording hologram and then applying numerical reconstruction and twin image removal are available in <https://github.com/OpticsInformationLab/Random-Illumination-HolographyDL> and the codes of recovery of the wavefront from spatially fluctuating fields using the two point intensity correlation, i.e., fourth order correlation are available in <https://github.com/OpticsInformationLab/IntensityCorreltion>

S16 Super-resolution Imaging using Structured Light (Gangi Reddy Salla, Ravi Kumar, Sakshi, Inbarsan Muniraj, Shashi Prabhakar and R. P. Singh) – Section 28

%% Code for optical random pattern: %%

clc; clear all; close all;

n=0;

GSB3_512=zeros(1023,1023);

m=0;

[y,x] = meshgrid(1:512,1:512); % Create a calibrated workspace

r = length(x)/2; c = length(y)/2; % Spatial coordinates in cylindraical coordinate system

rho = sqrt((x-r).^2 + (y-c).^2); % Correlation width defines the average grain size

Corr_width = 6.5;

p=rho.^2/Corr_width^2;

theta=atan2(y-256,x-256);

r=sqrt((x-256).^2+(y-256).^2);

E1=r.^(m).*(exp(-1i*m.*theta)); % Spatial mode, here LG beam

window1 = polyval(LaguerrePoly(n),p).*exp(-p).*E1; % Final Speckle field

I = normrnd(0,1,512,512); % Introducing the random phase

GSB1 = conv2(window1,I);

GSB1=abs(GSB1).^2;

GSB1=GSB1(300:700,300:700);

%% Code for functional form of LG %%

% LaguerrePoly.m by David Terr, Raytheon, 5-11-04

% Given nonnegative integer n, compute the

% Laguerre polynomial L_n. Return the result as a vector whose mth

% element is the coefficient of x^(n+1-m).

% polyval(LaguerrePoly(n),x) evaluates L_n(x).

function Lk = LaguerrePoly(n)

if n==0

Lk = 1;

elseif n==1

Lk = [-1 1];

else

Lkm2 = zeros(n+1,1);

Lkm2(n+1) = 1;

Lkm1 = zeros(n+1,1);

Lkm1(n) = -1;

Lkm1(n+1) = 1;

for k=2:n

Lk = zeros(n+1,1);

for e=n-k+1:n

Lk(e) = (2*k-1)*Lkm1(e) - Lkm1(e+1) + (1-k)*Lkm2(e);

end

Lk(n+1) = (2*k-1)*Lkm1(n+1) + (1-k)*Lkm2(n+1);

Lk = Lk/k;

if k<n

Lkm2 = Lkm1;

Lkm1 = Lk;

end

end

end

S17 Polarization Encrypted Difffractive Optical Elements for Point Spread Function Engineering (Vipin Tiwari) – Section 29

%% Polarization encrypted DOEs

clear all

N=1000;

lambda=0.633*10^-6; %wavelength in meters

P=10^-3; % Radius aperture (1 mm)

pixel=8*10^-6;

% PCA for lens

A=zeros(1000);

Radius=100;

x1=501;

y1=501;

for x=1:1000;

for y=1:1000;

if sqrt((x-x1).^2+(y-y1).^2)<Radius;

A(x,y)=1;

end

end

end

B=zeros(1000);

x1=501;

y1=501;

for x=1:1000;

for y=1:1000;

if sqrt((x-x1).^2+(y-y1).^2)<(Radius/sqrt(2));

B(x,y)=1;

end

end

end

R=A-B; % ring aperture

Rp=exp(1i*pi/2)*R;

PCA=angle(Rp); % PCA

%% PSF calculation

Ef=fftshift(fft2(A));

Eh=fftshift(fft2(B));

Er=fftshift(fft2(R));

Ep=fftshift(fft2(PCA));

%imagesc(abs(Er)),axis square, colormap 'gray', axis off;

% Intensity plot

plot(abs(Er(501,400:600)./max(abs(Ep(501,400:600)))),'linewidth',3),set(gca,'FontName','Arial','FontSize',35,'FontWeight','Bold', 'LineWidth', 4);

hold on

plot(abs(Ep(501,400:600)./max(abs(Ep(501,400:600)))),'linewidth',3, 'color','red'),set(gca,'FontName','Arial','FontSize',35,'FontWeight','Bold', 'LineWidth', 4);

axis tight, legend ('Ring', 'PCA'),legend("Position", [0.15544,0.69987,0.0959,0.18245]);

%% Binary Axicon

%sample space

x=1:N;

y=1:N;

[X,Y]=meshgrid(x,y);

R=sqrt((X-N/2).^2+(Y-N/2).^2).*pixel;

Aperture=zeros(N,N);

Aperture(R<N/2*pixel)=1;

A1=ones(N,N);

A2=ones(N,N);

A1(rem(R,P)<P/2)=exp(1i*pi);

Axb=A1.*Aperture;

A2(rem(R,P)<P/2)=exp(1i*pi/2);

Axp=A2.*Aperture; % PCAx

% PSF calculation

EA1=abs(fftshift(fft2(Axb)));

EA2=abs(fftshift(fft2(Axp)));

%imagesc(abs(EA2)),axis square, colormap 'turbo', axis off;

% Intensity plot

plot(abs(EA1(501,400:600)./max(abs(EA2(501,400:600)))),'linewidth',3),set(gca,'FontName','Arial','FontSize',35,'FontWeight','Bold', 'LineWidth', 4);

hold on

plot(abs(EA2(501,400:600)./max(abs(EA2(501,400:600)))),'linewidth',3, 'color','red'),set(gca,'FontName','Arial','FontSize',35,'FontWeight','Bold', 'LineWidth', 4);

axis tight, legend ('BAx', 'PCAx'),legend("Position", [0.15544,0.69987,0.0959,0.18245]);;

%% BFZP

N=1000;

M=100;% number of grating lines

x=1:N;

y=1:N;

[X,Y]=meshgrid(x,y);

R=sqrt((X-N/2).^2+(Y-N/2).^2);

A=ones(N,N);

A2=ones(N,N);

A1=ones(N,N);

A1(R<N/2)=1;

f=3000;%focal length

lambda=0.633;

for n=1:M;

r1(n)=sqrt(n*f*lambda); % 1D FZP equation

end

for n=1:2:M;

for p=1:N;

for q=1:N;

r(p,q)=sqrt((p-N/2)*(p-N/2)+(q-N/2)*(q-N/2));

if r(p,q) >r1(n) && r(p,q) < r1(n+1);

A(p,q)=exp(1i*pi);

end

end

end

end

for n=1:2:M;

for p=1:N;

for q=1:N;

r(p,q)=sqrt((p-N/2)*(p-N/2)+(q-N/2)*(q-N/2));

if r(p,q) >r1(n) && r(p,q) < r1(n+1);

A2(p,q)=exp(1i*pi/2);

end

end

end

end

FZP=A1.*A; % FZP_n

FZP_pol=A2.*A1; %FZP_pol

%imagesc(abs(Efzp_pol(400:600,400:600))),axis square, colormap 'turbo', axis off;

% PSF calculation

Efzp=fftshift(fft2(FZP));

Efzp_pol=fftshift(fft2(FZP_pol));

plot(abs(Efzp(501,450:550)./max(abs(Efzp_pol(501,450:550)))),'linewidth',3),set(gca,'FontName','Arial','FontSize',35,'FontWeight','Bold', 'LineWidth', 4);

hold on

plot(abs(Efzp_pol(501,450:550)./max(abs(Efzp_pol(501,450:550)))),'linewidth',3, 'color','red'),set(gca,'FontName','Arial','FontSize',35,'FontWeight','Bold', 'LineWidth', 4);

axis tight, legend ('BFZP', 'PCFZP'),legend("Position", [0.15544,0.69987,0.0959,0.18245]);
